# Support To Rural India’s Public Education System (STRIPES2) and impact on numeracy and literacy scores: A cluster randomized trial in rural villages of Madhya Pradesh, India

**DOI:** 10.1371/journal.pone.0330203

**Published:** 2025-09-12

**Authors:** Ila Fazzio, Siddharudha Shivalli, Nicholas Magill, Diana Elbourne, Suzanne Keddie, Dropti Sharma, Sajjan Singh Shekhawat, Arjun Agarwal, Rukmini Banerji, Sridevi Karnati, Harshavardhan Reddy, Tony Brady, Piotr Gawron, Pei-Tseng Jenny Hsieh, Alex Eble, Peter Boone, Chris Frost

**Affiliations:** 1 Effective Intervention, London, United Kingdom; 2 London School of Hygiene and Tropical Medicine, London, United Kingdom; 3 Pratham Education Foundation, New Delhi, India; 4 GH Training and Consulting, Hyderabad, Telangana, India; 5 Sealed Envelope, London, United Kingdom; 6 National Foundation for Educational Research, London, United Kingdom; 7 Teachers College Columbia University, New York, United States of America; Northeastern University, UNITED STATES OF AMERICA

## Abstract

**Introduction:**

Rates of primary school enrolment have improved in India, but levels of learning achievement remain low. In the Support To Rural India’s Public Education System (STRIPES) trial, a para-instructor intervention improved numeracy and literacy levels in Telangana, India (2008−10). The STRIPES2 trial was designed to assess whether a similar intervention in a younger cohort of children would have similar effects in Satna and Maihar districts of Madhya Pradesh, India, and be cost-effective.

**Methods:**

In this Madhya Pradesh cluster-randomized controlled trial, 196 villages (clusters) were randomized to receive either a health (CHAMPION2: community health promotion and medical provision and impact on neonates) or education (STRIPES2) intervention. Villages receiving the health intervention were controls for the education intervention and *vice versa*. For children newly enrolled in primary school, the STRIPES2 intervention comprised before/after-school classes (2 hours per day, 6 days a week) given by trained para-instructors from the local community, frequent monitoring, and engagement with caregivers to motivate children, delivered by the Pratham Education Foundation. STRIPES2 activities had to be suspended twice for around ten and a half months, and some components of the intervention modified due to the COVID-19 pandemic. The period of the trial was extended with the primary outcome (a composite literacy and numeracy score of Early Grade Reading and Mathematics Assessments) assessed around 30 months after classes started.

**Results:**

Composite test scores were significantly higher in the intervention arm (98 villages; 3054 children) than in the control arm (98 villages; 3275 children) at the end of the trial. The mean difference on a percentage point scale was 14.17; 95% CI 11.36 to 16.97; p < 0.001, equating to a 0.58 (95% CI 0.47 to 0.71) standard deviation difference. The cost per child per 0.1 SD increase in composite test score was INR 2476 (US$33.5).

**Conclusion:**

Despite COVID-19 interruptions and disruptions, STRIPES2 resulted in a major improvement in children’s literacy and numeracy. However, the cost of achieving such benefits was substantial.

## Introduction

In common with many other low- and middle-income countries (LMICs), India has witnessed a massive expansion in school enrolment over the last 20 years, and yet many students finish primary education without the foundational literacy and numeracy skills that would be expected as indicated by their grade and national curriculums [1,2]. More than half of children in LMICs, who have finished, or are close to finishing, their primary education have not acquired the basic skills to read and comprehend a short story appropriate for their age [3–6]. In Madhya Pradesh, India, according to ASER (Annual Status of Education Report) 2018 [5], the overall school enrolment of children between six and fourteen years old in rural villages was very high (96%), with about 72% of them enrolled in public schools in 2017–18 [5]. Yet, the results of the ASER reading tests showed that only 41.6% of children in grade 5 (10–11 years old) were able to read the short story (with familiar words and a simple sentence construction that is typical of Indian Grade 2 textbooks). Among the Grade 3 children (7, 8 and 9 years old), only about 10.4% of those enrolled in public schools were able to read the short story. In mathematics, ASER 2018 data showed that only about 8.5% of grade 3 students in public schools in rural Madhya Pradesh could perform at least 2-digit by 2-digit subtraction with borrowing (this being expected of children who finish 2^nd^ grade in most states). This low reading and mathematics attainment of students despite high school enrolment can be partly attributed to weak engagement, support, and monitoring of teachers. There is some evidence that teachers frequently do not attend classes or are not engaged in teaching even when present in the classroom [7]. Moreover, in public schools in Madhya Pradesh (as in all India states), teachers are instructed to complete the syllabus for each grade, regardless of students’ learning outcomes and ability to understand the content of what is being taught. As a result, slow performing students do not have a chance to catch up on what is being taught and frequently end up showing a very poor performance even in basic skills.

Studies aiming to raise students’ learning, followed by several reviews [8–10], have provided frameworks for comparing interventions and recommending the best ones in terms of years of learning and cost benefits, so governments and organizations can make informed investment to improve the quality of education. In these reviews, remedial instruction taught by community instructors with an improved pedagogy to match teaching to students’ learning level is cited as one of the most effective strategies for primary school age children [6,8,9,11,12]. Remedial instruction targets the foundational skills that a child needs to master. Its core idea is that if a child has not acquired the foundational skills in literacy and numeracy, it is not possible to assimilate more complex concepts and skills.

Many of the strategies aimed at addressing the poor quality of education fall short of enhancing children’s learning. This inadequacy is mainly due to their focus on interventions that were limited to providing facilities, materials, or school access, rather than focusing on improving the child’s learning experience [8–10,13–15].

Studies from Chile and various parts of India reported that extra teaching by tutors or volunteers from the local community improved literacy and numeracy skills among early primary grade children [16–18]. For instance, offering remedial classes to third and fourth graders in public schools located in Mumbai and Vadodara, India, led to an increase of 0.28 standard deviations in overall test scores for literacy and numeracy in the second year [17]. Another study from Uttar Pradesh, India, demonstrated a large positive impact in both language (Hindi) and mathematics of children in Grades 3–5 who consistently attended learning camps facilitated by community volunteers for 40 days where they were grouped and taught according to their learning levels for 1.5 hours per day [11]. Similarly, a tutoring initiative spanning three months and targeting low performing Grade 4 students in Chile resulted in an important improvement in their reading abilities [16].

The STRIPES trial [18] in Telangana, India (from 2008−10) evaluated the effectiveness of an intervention that provided 18 months of supplementary, remedial teaching and learning materials (and an additional “kit” of materials for girls) to children in Grades 2–4 at baseline. The primary outcome was a composite of language and numeracy test scores, revealing significantly higher scores in the intervention arm (107 villages; 2364 children) compared to the control arm (106 villages; 2014 children) at the end of the trial (mean difference on a percentage scale 15.8; 95% CI 13.1 to 18.6; p = 0.001; 0.75 standard deviation (SD) difference). The cost per 0.1 SD increase in composite test score per child was INR 382.97 (£4.45, $7.13). The STRIPES trial thus provided evidence that supplementary teaching, when implemented in remote rural areas, can significantly enhance numeracy and literacy skills at a reasonable cost. Given the scarcity of evidence from previous trials, there is potential to adapt and expand the STRIPES trial to ascertain the generalizability of its findings across diverse settings.

Building on STRIPES, the STRIPES2 trial was conducted to assess whether structured supplementary classes [19] in early grades had a similar effect on the literacy and numeracy of primary school age children in rural villages of Madhya Pradesh, and if it was cost-effective.

Despite the STRIPES and STRIPES2 interventions being designed and implemented by two different organizations (the Naandi Foundation in Telangana and Pratham in Madhya Pradesh) the core approach remained the same: providing supplementary classes led by para-educators recruited from local communities [19].

## Methods

### Study design, setting and participants

The CHAMPION2/STRIPES2 (Community Health Promotion And Medical Provision and Impact On Neonates, and Support To Rural India’s Public Education System and impact on numeracy and literacy scores) trial was a cluster-randomized trial conducted in rural villages in the Satna and Maihar districts of Madhya Pradesh. At the time the trial was conducted the Maihar district was part of the Satna district. Full details of the STRIPES2 trial design and planned statistical analysis can be found in the CHAMPION2/STRIPES2 protocol paper [20], protocol update [21] and statistical analysis plan [22]. In brief, 196 villages were randomized, with 98 receiving a package of interventions to improve literacy and numeracy (STRIPES2) and 98 (CHAMPION2) receiving a package of maternal and newborn interventions. The villages receiving the CHAMPION2 intervention acted as controls for the STRIPES2 trial and vice-versa. The reason this design was adopted was (as with the original STRIPES and CHAMPION trials) to create a positive presence in all the villages in both trial arms, thereby encouraging STRIPES2 control children to give us data and attend the midline and endline tests even though they were not receiving any educational intervention. The CHAMPION2 intervention was primarily aimed at improving outcomes in pregnancy, whereas the STRIPES2 intervention aimed to improve educational standards in primary school-age children. We accordingly anticipated that the CHAMPION2 intervention would not materially impact on outcomes in STRIPES2 and vice-versa.

The trial was conducted in Satna district, Madhya Pradesh, India. Satna district is further divided into 10 *tehsils* (sub-districts). Three *tehsils* (*Birsinghpur, Majhgawan* and *Raghurajnagar*) were excluded due to difficult access (forest area), risk of violent robbery, and being an urban sub-district. The remaining seven *tehsils* comprised 1263 villages (68% of all villages in Satna), with a population of 1,441,930 [23]. From these we selected the 484 villages that (i) were considered ‘rural’ with a population less than 2500 inhabitants (ii) had at least 120 children under the age of 6 years and (iii) were accessible by road. An algorithm was written to select a maximally sized subset of these villages such that (iv) each village center was at least 5 km away from the nearest Community Health Centre (CHC) and (v) there was a minimum of 3 km between village centers to avoid contamination (buffer zones). The requirement that villages should have at least 120 children under the age of 6 was included because this gave an expectation of 20 children in each academic year and therefore a good chance of there being at least 15 children eligible for the intervention. In the event that (vi) fewer than 15 eligible children were identified at enumeration (or that villages were considered to be urban rather than rural) villages were not randomized.

In STRIPES2, the target group was children born between 16 June 2010 and 15 June 2013, whose caregivers were planning to enroll them in Grade 1 for the first time in the 2018–2019 school year, and resident in the trial villages. Because of delays in getting the trial started, enumeration took place twice (July 2017 and April 2019), so eligible children who were missed the first time had a second chance to participate in the trial.

All data collection and related research activities for the STRIPES2 trial were organized by independent teams recruited, trained and monitored by GH Training and Consultancy (GHTC), India.

This trial employed multiple tiers of consent: village and individual level consent from the caregiver on behalf of the child. Agreement to approach eligible villages was first obtained from the village heads (*Sarpanchs*). In the trial villages, consent was obtained from the village after the trial had been presented in a meeting with village elders representing all the castes and village residents. Consent was given orally by the elders during a village meeting, and the *Sarpanchs* of the villages signed (or gave their thumbprint) in a document that described the trial. During the enumeration survey, GHTC interviewers read a simple standard consent explanation of the trial in Hindi (local language) and caregivers of eligible children were offered the chance to participate, giving signed or thumbprint consent if they wished to. This trial’s protocol [20] and its amendment [21] were approved by the Ethics Committees of L V PRASAD Eye Institute, Hyderabad, India (LEC 02–16−008) and London School of Hygiene and Tropical Medicine (LSHTM Ethics Ref: 10482). We also obtained the approvals from the Indian Council of Medical Research, New Delhi and the Government of Madhya Pradesh to conduct this trial in Satna district. The trial complies with the Declaration of Helsinki, local laws, and the International Conference on Harmonization Good Clinical Practice (ICH-GCP).

### Intervention design

The STRIPES2 intervention aimed at building foundational literacy and numeracy skills for Grade 1 and 2 children in a holistic manner, combining multiple interventions to promote learning outside and inside the child’s home. Caregivers (especially mothers) had a crucial role in engaging with their children’s learning. Also, its content extended beyond academic learning, as it included activities to develop children’s social and emotional skills. The approach involved a combination of individual, small group, and large group activities, incorporating play-based teaching methods that included: storytelling, reading, playing, singing, coloring, drawing, and role-playing. This approach aims to help make learning enjoyable and engage children in a manner that aligns with their natural instincts.

The STRIPES2 intervention design was based on Pratham’s experience across multiple states in India, and especially in Madhya Pradesh [11]. Pratham has been working in Madhya Pradesh with Grade 1 and 2 children since 2005, indirectly supporting projects that involved training and fostering community volunteers to interact with local schools’ teachers and mothers to engage in children’s academic, emotional and physical learning through the development of workbooks for Grades 1 and 2. However, this was the first time that Pratham was working directly in the trial area (Satna and Maihar districts).

Over the years, Pratham has worked closely with the state government to provide primary school teachers with innovative teaching methods and curricula, aiming to enhance children’s proficiency in core subjects. With a focus on Grades 1 and 2, programs in partnership with the government have addressed essential competencies in language and mathematics. Teacher training, utilizing Pratham’s “teaching-learning” and “teaching-at-the-right level” methods, have been central to these initiatives, overseen by both government and Pratham personnel. In 2013, Pratham partnered with the Madhya Pradesh government in selecting, training and sometimes hiring education coordinators to provide on-site support to teachers, enhance instructional materials and practices, ensure effective curriculum implementation, address challenges in the classroom (such as diversity in learning levels etc.) and improve overall quality in primary education. The design of the teaching component of the STRIPES2 intervention was based on Pratham’s innovative [18,24] activity-based content and curriculum package developed for Grades 1–2 that focuses on improving foundational literacy and numeracy (FLN) whilst fostering other developmental areas such as physical, social, and emotional growth. It follows a phased approach, starting with a warm-up phase to prepare children from diverse backgrounds (see Manual for Instructors/Teachers: Warm Up – Phase I Language & Math Class 1 & 2, in S1 Appendix), followed by instructional phases that strengthen literacy and numeracy skills [18,24].

The key components of Pratham’s method are:

- Simple Testing Tools: One-on-one oral tools and worksheets are used to track progress, with assessments conducted at baseline and end-line stages.
- Daily free play (10–15 minutes), group socio-emotional activities like storytelling and role-playing, and physical tasks such as running and jumping (see examples of games and activities in the *Manmauji Ganit* Booklet – S2 Appendix).
- Activity-Based Instruction: Activities adapted to individual learning levels and encourage gradual improvement in reading and arithmetic.
- Contextual and Low-Cost Materials: Locally relevant materials are used, including stories, flashcards, and print-rich environments to support language development. Context-specific materials and culturally adapted rhymes are included as optional elements. To ensure effective implementation, teachers are trained with hands-on practice, supported by activity booklets and a structured two-hour lesson plan framework.
- Focus Domains: Emphasis is placed on language (oracy, phonological awareness, vocabulary, fluency, comprehension, writing) and mathematics (pre-mathematics concepts, numbers and operations, measurement, geometry, data handling)

The para-educators, called Pratham instructors (PIs), were female residents in the intervention villages. All had completed 12^th^ Grade and passed a written exam during the selection process. By hiring female teachers, Pratham also intended to empower local women. PIs received a 6-day training by Trainers (Manual for Master Trainers – Grades 1 and 2 in S3 Appendix)

PIs were responsible for conducting classes either before or after school hours, maintaining contact with families, and engaging mothers in the learning process. They were overseen by a team of full-time cluster leaders (CLs), each CL being responsible for approximately 10–12 villages. CLs provided continuous monitoring, training, and mentoring support to PIs. Additionally, the CLs played a crucial role in facilitating the engagement of PIs with the local community, assisting them in talking to parents about the importance of these before/after school classes and finding adequate places in the village to hold the classes.

Before the launch of the program, Pratham teams piloted the activities and materials for these before/after school classes in rural villages of Bhopal district, Madhya Pradesh. In November 2019, Pratham started the 3-month preparatory phase for students’ readiness. The initial 2–3 weeks period was a warm-up phase and focused on nurturing attendance (see S1 Appendix). During that stage PIs worked on mobilizing families to send their children to the classes, motivating children to come on time to classes and getting them used to sitting for long activities. PIs carried out a series of activities such as playing, singing, coloring, and drawing, to make children feel at ease within the school and class so that they were able to interact comfortably and independently with the instructors and their peers (see Activity Book *Manmauji Ganit* – S2 Appendix).

After the warm-up phase, the STRIPES2 teaching-learning approach was implemented in three phases: readiness (phase I) and instructional (phases II and III). The readiness phase (for 4 weeks) included simple, interesting, and well-designed activities focusing on a preparatory stage, aiming to familiarize children with the classroom environment, develop emergent literacy and numeracy skills, and promote overall growth in various developmental areas. The emphasis on diverse activities and the teacher’s active role in understanding individual needs contribute to a comprehensive approach to early childhood education. Each of the instructional phases II and III were implemented for about 2–3 months and aimed to strengthen children’s literacy (Hindi) and numeracy learning.

In addition to teaching, the PIs were actively involved in maintaining frequent communication with caregivers through door-to-door interactions (*home visits)* and fortnightly meetings with caregivers (*caregivers’ meetings*). During these *home visits* and *caregivers’ meetings*, the PIs engaged with caregivers to discuss children’s learning progress, offer suggestions for fun activities that could support the learning process and investigate whether caregivers were conducting these activities (reading and mathematics), and check whether the activities were appropriate to the child’s learning level.

In March 2020, about 4 months after the intervention had started in the communities, the government of India declared a nationwide lockdown due to the COVID-19 pandemic. As a result, the intervention activities were first suspended and then resumed with adaptations. Between August and November 2020, the program had to be limited to calls and text messages by the PIs. The initial text messages were focused on health precautions and then shifted to focus on basic language and mathematics. These activities were adapted from Pratham’s national campaign the “*Karona:thodi Masti, Thodi Padhai*” (Do it: A little fun, a little study) [25]. It is important to highlight that these activities were not intended as formal teaching but rather as a means of promoting caregivers’ engagement and sparking children’s interest. In contrast, the instructor-led model that was adopted as soon as COVID-restrictions were lifted involved focused, structured learning for two hours daily [25]. Moreover, PIs also conducted daily follow-up calls to ensure the seamless delivery of the content. These calls played a crucial role in building a rapport with the parents and ensuring their active participation in the activities. Despite the changes made during the pandemic, the education program remained mainly instructor-led, not caregiver-led.

During the lockdown period, the relief schemes (like distribution of activity books and text messaging) provided by the government were designed and implemented uniformly, ensuring similar access and support in both trial arms.

**Fig 1:**
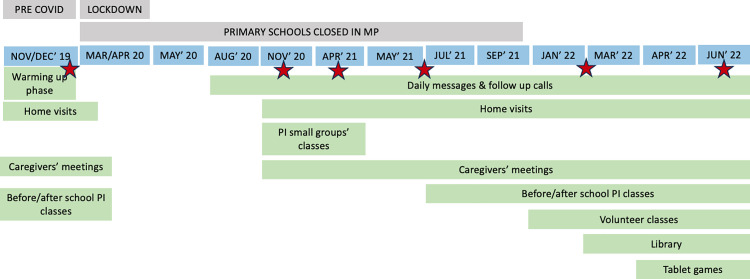
Timeline of the STRIPES2 trial activities in Madhya Pradesh, India.

As COVID-19 restrictions eased (November 2020), the subsequent phase involved setting up small group interactions with 2–5 children (*small group classes*) (Fig 1) to understand learning levels of children after a prolonged break and prepare them to resume classroom activities. In parallel, PIs restarted *home visits* to actively engage with children and their caregivers/family members (both individually and in small groups), during which they talked with caregivers/family members and children about queries or concerns. The frequency of these visits and group activities was related to the availability and willingness of parents and the village head, as well as the number of children that each PI was responsible for. In parallel with these activities, Pratham teams conducted six ASER-like assessments throughout the trial period to keep track of how children’s learning was improving. These are represented in Fig 1 as red stars (December 2019, November 2020, April and August 2021, February and June 2022).

Around July 2021, the intervention team, with the help of the local community, identified suitable class locations that allowed for social distancing, so that PIs could restart teaching the before/after school classes. Additionally, PIs were trained to discuss the risks associated with COVID-19, ways to reduce its transmission and sensitize caregivers to avoid sending their children to classes if anyone at home had symptoms.

From January 2022 onwards, a group of 163 young volunteers started conducting 20- to 40-minute-long fun activities with groups of 4–5 children on a regular basis (*volunteer classes*). The 163 volunteers engaged in this initiative were primarily young females from the community who had demonstrated a keen interest in working with children on a consistent basis. They had foundational skills in reading and mathematics, enabling them to facilitate learning activities with the children. To mobilize these potential volunteers, a month-long collaborative effort was undertaken by PIs and Community Leaders (CLs). This initiative involved various strategies, including door-to-door meetings and large group gatherings within villages, facilitated with the support of the local *Sarpanch* (village head) and other key community stakeholders.

A month later, the same volunteers were trained in performing (and creating materials for) storytelling (for instance: drawing, painting, and cutting cardboards to create characters and scenarios; and ways of dramatizing when telling a story). In March 2022, informal libraries were created in the local shops to provide a platform where children’s books could be easily borrowed, encouraging interest in books, and providing material for children to practice reading.

In April 2022, volunteers began using tablet games to reinforce and support learning through fun activities in sixty-five of the intervention arm villages. These sessions, conducted daily for 25–30 minutes with groups of 4–5 children, were offered to all enrolled children but targeted especially at children who were struggling to recognize and interpret the sounds associated with syllables (which is essential for reading and writing) and number recognition.

Table 1 presents a list of the components of Pratham’s intervention, indicating whether each was considered core or optional. Although each component could not be implemented simultaneously in all villages, Pratham teams followed a consistent process of implementation for each of them except the formal libraries.

**Table 1:** Core and optional components of Pratham’s intervention.

Components of Pratham’s intervention	Core	Optional
A warm-up phase (2–3 weeks) aimed at nurturing attendance by engaging families and motivating children to come to classes	√	
Daily free play in groups (storytelling, physical activities, role-playing)	√	
Activities adapted to individual learning levels (Play and Activity-Based Learning)	√	
Learning materials that are cheap and locally relevant	√	
Structured two-hour lesson planning	√	
Simple ASER-like tests that aimed at capturing foundational skills like phonological awareness, vocabulary knowledge, listening comprehension, early writing skills, mathematical patterns, classification, sequences, one-to-one correspondence, quantity comparison, etc.	√	
Hands-on-PI (teacher) training	√	
Home visits	√	
Caregivers’ meetings (every two weeks)	√	
Text messages		√
Follow-up calls to families		√
Tablet games		√
Fun activities with small groups		√
Informal libraries		√

### Outcomes

The primary outcome was the arithmetic mean of the child’s scores on EGRA (Early Grade Reading Assessment) and EGMA (Early Grade Mathematics Assessment) tests [26,27]. We chose to use EGRA and EGMA because of our positive experiences applying them in similar contexts (as they are sensitive in measuring small differences in ability among children who have very low levels of learning), and because they are widely used tests for assessing early grade literacy and numeracy.

Secondary outcomes included separate scores for literacy and numeracy; caregivers’ engagement in their child’s learning; enrolment in school; caregiver’s report of school attendance; and the cost effectiveness of the intervention. Owing to the interruptions caused by the COVID-19 pandemic, the STRIPES2 intervention finished later than had initially been planned, meaning that the intervention (and the final testing from July-September 2022) was carried out in older children than would otherwise have been the case.

### Sample size and randomization

The sample size calculation was primarily driven by the CHAMPION2 (health) intervention (details published in the protocol and update) [20,21]. Originally, the intention was to randomize 300 villages, which would give over 90% statistical power to detect a difference of 0.25 standard deviations in mean standardized test scores for STRIPES2. In the first STRIPES trial (Telangana), [8] the estimated effect was a 0.75 standard deviation (SD) increase in mean score: however, an effect of smaller magnitude than this would still be important to detect.

After drawing the buffer zones, it turned out that only 204 villages could be selected. These 204 villages had a mean population of 1487 (minimum 558, maximum 2490) and a standard deviation of 505 (equating to a coefficient of variation of 0.34). Estimating the number of children in each school year from the number under the age of six years old, the mean number of children in each school year was 38.3 (minimum 20, maximum 71) with a standard deviation of 13.3 (a coefficient of variation of 0.35). Assuming that 25% of the children would not satisfy the eligibility criteria, the mean number of eligible children per village was estimated as 28.7 with a minimum of 15. Further assuming that i) 95% of the 204 villages would ultimately participate, ii) 60% of the eligible children in these villages would take the test at the end of the trial, iii) an intra-cluster correlation coefficient of 0.23 (as seen in the STRIPES trial [8]) and iv) a coefficient of variation in numbers taking the test by village of 0.35 gave the trial 88% power to detect a difference of 0.25 SD in mean standardized scores between intervention and control villages using a conventional 2-sided statistical significance level of 5%. In fact, 196 of the 204 villages were randomized (6 villages being removed because they were found to be too close to urban areas to be considered rural, and 2 being removed because there were insufficient eligible children).

All children were enumerated and enrolled before randomization. Randomization of clusters was performed by the trial statistician based in London in June 2019 using a random number generator, with stratification by village size (above or below median) and distance to the nearest Community Health Centre or Civil Hospital (above or below median).

### Adherence

We measured adherence through children’s attendance at before/after school classes recorded by PIs, the participation of caregivers in fortnightly meetings and their compliance with reading and mathematics exercises (that were given by PIs for caregivers to practice with their children).

Before/after-school classes were not run in exactly the same way in all of the villages. Partly this was due to COVID-19 concerns and restrictions and the difficulty some children had to reach the place where these classes were run. As a result, there was variability in the number of planned classes per week, the length of these and the size of classes.

For simplicity, we decided to use counts of the numbers of classes i) offered to and ii) attended by each child. We assumed that, had the intervention run as planned, then each child would have been offered 360 classes (6 classes a week for 60 weeks, this corresponding approximately to a 16-month period with allowance for holidays etc.). We refer to this as the ideal number of classes. We calculated the total number of classes that were offered to each child and the total number of classes that each child attended. Where more than 360 classes were offered or attended, we took the number(s) to be 360. We defined adherence at child level as the proportion attended of ideal, proportion offered of ideal, and proportion attended of offered. We also did this at village level by calculating mean proportions within each village.

As well as recording attendance in classes, PIs recorded the attendance of caregivers at caregivers’ group meetings and asked whether they had carried out the language and mathematics activities/exercises with their children. These meetings occurred every two weeks so that caregivers could share experiences and talk about doubts related to their children’s learning. Attendance at these meetings was summarized in an analogous way to attendance in classes with ideal attendance being 24 classes. If the caregiver did not come to the meeting it was assumed that she had not carried out the activities with the child.

### Baseline data collection

Prior to randomization, data were collected on children’s gender, age, whether or not parents were alive and who their primary female and male caregivers were (mother, father, grandmother, grandfather etc.). In addition, data were collected on the caregivers’ religion, caste, literacy and education levels.

### Midline test and survey

GHTC teams conducted the midline tests with trial children about six months after PI classes restarted and a month after schools reopened. These midline ASER-like tests were developed by an expert in educational evaluations using the structure and rules of the main ASER reading and mathematics tools adapted to the context of rural Madhya Pradesh (S4 Appendix). They were conducted in both trial arms between December 2021 and January 2022 with the aim of understanding the learning levels of children soon after the pandemic-long break.

To ensure efficient and timely testing, this midline test and a midline survey were conducted in two separate periods. This minimized the risk of children gaining access to the tools before being tested.

The midline survey was conducted between February and April 2022 with the main caregivers. The aim of doing this survey was to monitor residence and school enrolment of participant children (both before and after the lockdown – academic years of 2019−20 and 2020−21) and gather some information about expenses related to schooling and educational challenges faced by and support given to children a few months after primary schools reopened in Madhya Pradesh.

Data were also collected at the midline survey (or at the endline survey if missed at midline) on the materials that houses were constructed from (whether the materials used for the floor, roof and walls were synthetic or natural) and on whether household members owned a television, radio, motorbike and/or a 4-wheeled vehicle. These variables were used to construct wealth indices.

### Endline tests and survey

The EGRA and EGMA protocols were adapted to the local context by the National Foundation for Educational Research (NFER, [28]) and a board of local language and curriculum experts and primary teachers. Three rounds of workshops were organized to adapt and evaluate the subtasks and the individual items in the test in terms of relevance, accordance to curriculum, and suitability to the local context and language. The validation of the Hindi test instruments included expert review and qualitative trials with a small number of children. The validated EGRA and EGMA protocols were uploaded onto tablets (using the Tangerine platform designed for EGRA and EGMA- https://www.tangerinecentral.org/tangerine to facilitate tests’ application), which provided an opportunity for immediate online uploads of the test scores and monitoring of the data (the English versions of the full test papers are given in S5 Appendix). A quantitative pilot of the assessments was conducted with children from nearby non-trial villages to further trial the test administration process, investigate possible order effects, and inform the assessment design. NFER conducted psychometric analysis (Rasch Model) with the pilot data and the result informed the final revision of the EGRA/EGMA protocols. To minimize the risk of children sharing the test content after they had taken the test, we conducted the test in each trial village over the course of a single day.

GHTC recruited an independent team of test administrators for the Endline test. They were otherwise not involved in the trial and were unaware of the randomization. The tests were administered orally and sequentially by two groups of test administrators (one group each for EGRA and EGMA). Tests were administered in one-on-one sessions to each participant child present in the village on the day of the assessment. High attendance at the tests was ensured by the village level mobilization team (see S6 Appendix for a more detailed description of the process of data collection). Tests were conducted about one month after the intervention teams had stopped the main activities (between 24 July and 19 September 2022).

The NFER team coordinated the process of adaptation, translation, and initial monitoring of the assessment administration.

The endline survey was a brief survey conducted by GHTC between November 2022 and January 2023 with the main caregivers to assess the enrolment of participant children in school (2022–2023 academic year), their reported attendance in school during the two weeks prior to the interview, and caregivers’ engagement with children in reading and mathematics activities at home (estimated hours per week).

### Statistical analysis

The main analysis was conducted according to the intention-to-treat principle. All enumerated children satisfying the eligibility criteria were included in the primary analysis. The primary outcome of the trial was the composite literacy and numeracy test score. Each test score was calculated as a simple arithmetic mean of the percentage of correct answers on each of the subtasks, evenly weighting each task and not accounting for time remaining (for subtasks composed of two parts (a and b), we used a mean of the two results): for a detailed description of the statistical analysis see the Statistical Analysis Plan [22]. Summary statistics were reported at both child level (in the main paper) and at cluster level (in S7 Appendix).

In the primary analysis the composite language and mathematics test scores at follow-up were compared using a linear regression model with the clustered sandwich estimator of variance (allowing for clustering at the level of the village). Covariates were randomization arm and the randomization stratification variables. The primary analyses were conducted using scores calculated on a percentage scale. We also present the comparison as a standardized effect to allow comparison with other studies. No external standard deviation (SD) was available, so the standardized effect was estimated by fitting a linear mixed model with the same covariates as above to the scores on the percentage scale. The linear mixed model included cluster-specific random effects, with the variance of these random effects and the residual variance allowed to differ between randomization arms. The standardized effect was estimated by dividing the estimated adjusted difference in scores by the estimated total SD in the control arm (this being the square root of the sum of the between- and within-cluster variances in the control arm). A nonparametric bootstrap confidence interval (bias corrected and accelerated, 2000 replications at cluster level, stratified by randomization arm) was computed for this standardized effect.

We used similar linear regression models to the main analysis to explore the effect of interactions between baseline factors and the intervention on the primary outcome. These factors were village population size, gender, two wealth indices, caste, female caregiver literacy and male caregiver education (the male caregiver was often not present to read the sentence, meaning many cases were missing). The data used to compute the two wealth indices were collected post-randomization, but it is implausible that the intervention will have impacted on these variables. In addition to these pre-specified analyses, we carried out a *post-hoc* interaction analysis investigating whether the effect on the primary analysis differed according to whether or not there was a pregnancy in the household as recorded in the CHAMPION2 trial, this being done to assess whether household involvement in the CHAMPION2 trial might have impacted on the STRIPES2 primary outcome. Were the CHAMPION2 intervention to have materially impacted on STRIPES2 outcomes it is likely that the most marked effects would be seen in children from households where there was a pregnancy.

For the primary outcome we also conducted a pre-specified per-protocol analysis comparing those with high adherence to all controls. High adherence was defined as attending more than 75% of the ideal number of before/after-school classes. We also utilized an instrumental variables approach to explore how the primary outcome varied with attendance as a percentage of ideal in a *post-hoc* analysis. We adopted the two-stage structural mean model (SMM(G)) approach described by Maracy and Dunn (2011) [29], assuming a linear relationship between attendance and outcome, stratifying by the randomization stratifiers and computing bootstrap 95% confidence intervals (bias corrected and accelerated, 2000 replications at cluster level, stratified by randomization arm) for the whole process.

We estimated the effects of the intervention on reading and mathematics test scores at midline, learning support (hours spent engaging child in reading or writing activities) and number of school days missed in the previous two weeks using analogous linear regression models to those used in the main analysis of the primary outcome. The intervention effect on whether the child was enrolled in school was expressed as an odds ratio with a 95% confidence interval obtained from a GEE model with a binary outcome, a logit link, and a ‘working’ assumption of independence, with robust standard errors to take account of clustering.

In a secondary analysis of the primary outcome, we addressed missing data using multiple imputation by chained equations. We included as auxiliary variables the randomization stratification factors, caste, gender, male and female primary caregiver literacy, the wealth indices, the adherence to intervention variables defined above, the midline test scores, school enrolment at endline, number of school days missed in the two weeks before endline interview, number of hours the caregiver spent engaging child in reading or writing activities post lockdown, caregiver’s report of school attendance, whether or not the child was enrolled in school pre and post the COVID-19 lockdown, school grade at endline, the child’s residence status and the variables quantifying the learning support (and spending) provided by family, school teachers, NGOs and/or private tutors during the time when schools were closed. We performed multiple imputation using the “jomo” package in R [30], which is able to impute clustered data. We carried out imputations separately by trial arm; we used 20 imputations.

In a further secondary analysis, we considered the composite score and the reading test score with one problematic item omitted. Soon after the tests started being administered, the NFER consultant (unaware of randomization status) noticed an issue with a reading comprehension task (first item in subtask 5b), as fewer children than expected were getting that item correct and the result was somewhat inconsistent with individual performances in the rest of the test. The question asked the child what the day/weather was like, after the child had read a related text about it being a windy day. This could have happened because in some parts of India, wind is associated with good weather (because it makes the usually hot days more pleasant), hence quite a number of children answered “good, nice” and sometimes “cool”, or because the question asking about the state of the weather was not accurately translated. Given that this issue was not flagged during the validation or the training, we decided to carry on conducting the tests as they were and conduct a sensitivity analysis omitting the score from EGRA subtask 5b question 1, which was judged to be potentially misleading.

We used a significance level of 0.05 and report 95% confidence intervals. For the interaction tests, claims of different effects in subgroups were only made if there was strong evidence (p < 0.01) of an interaction. We conducted all analyses apart from the multiple imputation in Stata 18 [31]. Full details of analyses are described in the statistical analysis plan [22].

### Cost effectiveness

Cost effectiveness was calculated using actual budget expenditures. In 2020 and 2021, the before/after school classes had to be suspended for 10 and a half months because of COVID-19 restrictions. Although salaries continued to be paid normally for all the staff working for the program, we present the results of cost effectiveness per child both including and excluding the expenses during the period that these classes were not being conducted due to the COVID-19 pandemic.

For cost effectiveness, we assumed the total number of children impacted by the program to be the numbers taking the endline test.

Annual costs were converted to 2021 prices using the Indian GDP deflator, and then adjusted to USD using the average annual Indian Rupees (INR) to US dollars exchange rate.

## Results

**Fig 2:**
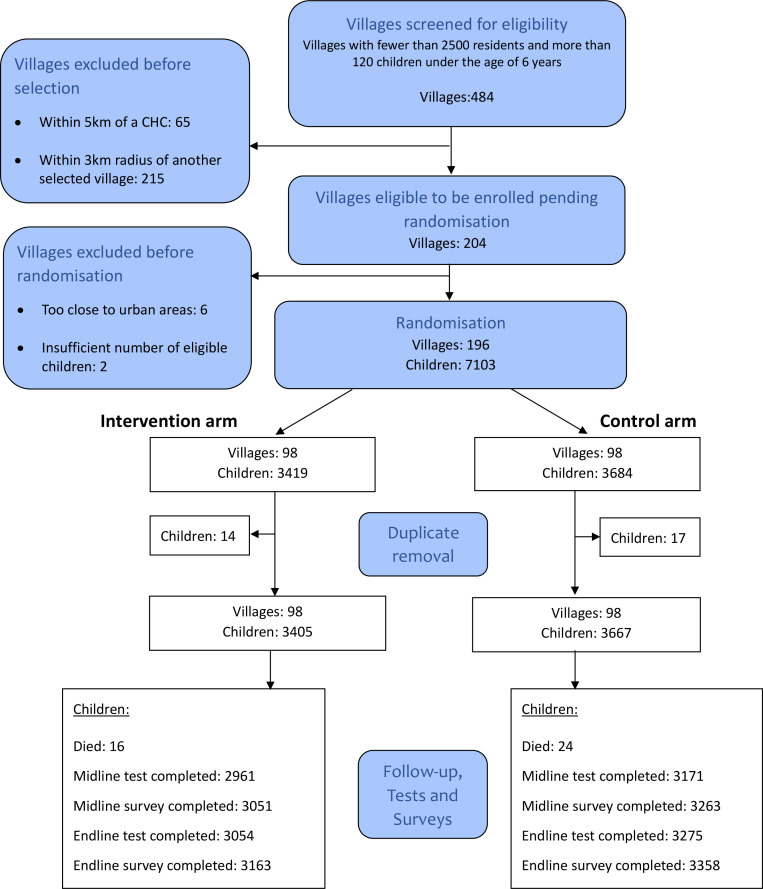
Flow diagram showing the selection of villages and the flow of participants.

The flow diagram in Fig 2 shows the selection of villages and flow of participants throughout the study. At randomization it was thought that there were 7103 children (3419 in the intervention arm, 3684 in the control arm) included in the trial. However, during the course of the trial it was discovered that 31 of the children had been erroneously enrolled twice, leaving 7072 (3405 in the intervention arm, 3667 in the control arm).

3054 out of 3405 (89.7%) children in the intervention arm and 3275 out of 3667 (89.3%) children in the control arm completed the final test. For the Endline survey 3163 of 3405 (92.8%) caregivers in the intervention arm and 3358 of 3667 (91.5%) of caregivers in the control arm were interviewed.

Baseline characteristics of villages and children are shown by trial arm in Tables 2 and 3 respectively. Table 3 and all subsequent tables present results at the child level (cluster level means and standard deviations are given in S7 Appendix). Following recommended best practice [32–34] we do not conduct statistical tests for differences in baseline characteristics, as any differences would necessarily have arisen by chance. Villages in the two arms were very similar in terms of size and distance to the nearest hospital/community health center. Children in the intervention and control arms were similar in all characteristics surveyed at baseline, with the gender ratio, and distributions of family caste, caregivers’ education, caregivers’ literacy, and children’s age all being comparable. In both arms, almost all families were Hindu. Almost all children in both control and intervention arms had biological parents as the main caregivers. There was little missing data for most variables, the exception being male caregiver literacy (assessed by handing over a literacy card to be read aloud), because many fathers were not present at the time of the enrolment interview.

**Table 2:** Baseline village characteristics.

Variable	Intervention arm N = 98	Control arm N = 98
*Village size (total population)*		
Mean (SD)	1504.8 (491.6)	1459.1 (515.3)
Median (IQR)	1384.5 (1138.0, 1967.0)	1371.0 (1063.0, 1894.0)
*Distance (km) to nearest Community Hospital/ Community Health Centre*		
Mean (SD)	12.5 (5.5)	12.6 (5.5)
Median (IQR)	11.3 (8.2, 15.6)	11.4 (8.5, 16.7)

**Table 3:** Baseline child characteristics.

Variable	Intervention arm N = 3405	Control arm N = 3667
*Gender*		
Female	1,641 (48.2%)	1,790 (48.8%)
*Family religion*		
Hindu	3,354 (98.5%)	3,615 (98.6%)
Muslim	26 (0.8%)	14 (0.4%)
Missing	25 (0.7%)	38 (1.0%)
*Family caste*		
Scheduled Caste	723 (21.2%)	821 (22.4%)
Scheduled Tribe	936 (27.5%)	1,102 (30.1%)
Other Backward Caste	1,330 (39.1%)	1,263 (34.4%)
Forward Caste	391 (11.5%)	443 (12.1%)
Missing	25 (0.7%)	38 (1.0%)
*Child’s main female caregiver*		
Biological mother	3,306 (97.1%)	3,561 (97.1%)
Stepmother	8 (0.2%)	9 (0.2%)
Grandmother	31 (0.9%)	37 (1.0%)
Other female family member	10 (0.3%)	8 (0.2%)
Other	35 (1.0%)	45 (1.2%)
No female caregiver	15 (0.4%)	7 (0.2%)
*Child’s main male caregiver*		
Biological father	3,289 (96.6%)	3,528 (96.2%)
Stepfather	5 (0.1%)	7 (0.2%)
Grandfather	26 (0.8%)	33 (0.9%)
Other male family member	14 (0.4%)	16 (0.4%)
Other	43 (1.3%)	55 (1.5%)
No male caregiver	28 (0.8%)	28 (0.8%)
*Female caregiver’s education:*		
No schooling	1,004 (29.5%)	1,107 (30.2%)
Primary	765 (22.5%)	933 (25.4%)
Middle school	900 (26.4%)	939 (25.6%)
High school	497 (14.6%)	465 (12.7%)
Higher secondary	129 (3.8%)	116 (3.2%)
Graduate	48 (1.4%)	39 (1.1%)
Postgraduate	17 (0.5%)	15 (0.4%)
Missing	45 (1.3%)	53 (1.4%)
*Male caregiver’s education:*		
No schooling	472 (13.9%)	606 (16.5%)
Primary	601 (17.7%)	645 (17.6%)
Middle school	987 (29.0%)	1,042 (28.4%)
High school	823 (24.2%)	795 (21.7%)
Higher secondary	280 (8.2%)	323 (8.8%)
Graduate	108 (3.2%)	118 (3.2%)
Postgraduate	55 (1.6%)	29 (0.8%)
Missing	79 (2.3%)	109 (3.0%)
*Child’s age* (years; mean; SD)	6.9 (0.7)	6.9 (0.7)
*Mother alive at baseline*	3,359 (98.6%)	3,629 (99.0%)
*Father alive at baseline*	3,352 (98.4%)	3,603 (98.3%)
*Female caregiver’s literacy:*		
Can’t read	1,486 (43.6%)	1,650 (45.0%)
Can read part of the sentence	582 (17.1%)	649 (17.7%)
Read entire sentence	1,038 (30.5%)	1,014 (27.7%)
Missing	299 (8.8%)	354 (9.7%)
*Male caregiver’s literacy:*		
Can’t read	342 (10.0%)	421 (11.5%)
Can read part of the sentence	236 (6.9%)	266 (7.3%)
Read entire sentence	796 (23.4%)	856 (23.3%)
Missing^#^	2,031 (59.6%)	2,124 (57.9%)

# Many male care givers were not present at the time of the enrolment interview

### Migration and mortality

The participant children’s residence status was verified at midline (March 2022) and endline (December 2022). A high percentage (89.6% in the intervention arm, 89.0% in the control arm) of the primary caregivers completed in the midline survey interviews, with the percentage being slightly higher at the endline survey (92.9% in the intervention arm, 91.6% in the control arm). Table 4 shows the percentage of children who were resident in the village across intervention and control groups in both surveys done during the trial. In both intervention and control arms, very few children were not resident in their village at each of the surveys. In total, 40 children died during follow-up (16 in the intervention arm and 24 in the control arm).

**Table 4:** Children resident in the study villages at midline and endline.

Variable	Intervention arm N = 3405 n (%)	Control arm N = 3667 n (%)
Midline survey		
Yes	3,046 (89.5%)	3,257 (88.8%)
No	5 (0.1%)	6 (0.2%)
Dead	15 (0.4%)	21 (0.6%)
Missing	339 (10.0%)	383 (10.4%)
Endline survey		
Yes	3,150 (92.5%)	3,339 (91.1%)
No	13 (0.4%)	19 (0.5%)
Dead	16 (0.5%)	24 (0.7%)
Missing	226 (6.6%)	285 (7.8%)

### Adherence and attrition

Adherence measurement results can be found in Table 5. Adherence varied markedly between children. In some intervention arm villages, classes ran for up to 23 months (October 2019 to March 2020, January 2021 to April 2021 and July 2021 to July 2022 (inclusive)), longer than the initially planned ideal length of 17 months. However, there were significant disruptions due to the COVID-19 pandemic in many villages, impacting the continuity of classes even during these periods, leading to interruptions and potential gaps in learning for the children. On average 77% of the planned ideal number of classes were offered to children, with the average number of classes attended being 53% of that regarded as ideal. Only 37% of children attended more than 75% of the number of the classes that were regarded as ideal before the trial started. There were no substantial differences in patterns of attendance according to baseline characteristics (Table S4A in S7 Appendix).

**Table 5:** Adherence: Before/after-school classes attended in the intervention arm.

	Attended as a proportion of ideal (N = 3405)	Offered as a proportion of ideal (N = 3405)	Attended as a proportion of offered (N = 3405)
Mean (SD)	0.53 (0.36)	0.77 (0.36)	0.66 (0.29)
0	434 (12.7%)	412* (12.1%)	22 (0.6%)
>0 and ≤25%	616 (18.1%)	129 (3.8%)	326 (9.6%)
>25% and ≤50%	432 (12.7%)	205 (6.0%)	494 (14.5%)
>50% and ≤75%	664 (19.5%)	252 (7.4%)	757 (22.2%)
>75% and <100%	834 (24.5%)	512 (15.0%)	960 (28.2%)
100%	425 (12.5%)	1,895 (55.7%)	434 (12.7%)
Not computed	0 (0%)	0 (0%)	412* (12.1%)

*Attended as a proportion of offered could not be computed where no classes were offered.

In the intervention arm 3054 out of 3405 children (89.7%) contributed the primary outcome as did 3275 out of 3667 (89.3%) children in the control arm.

Table 6 shows attendance at the caregiver’s meetings. 75% of caregivers attended at least one class, but only 32% attended more than 75% of the number regarded as ideal. Amongst those who attended, only a few reported that they had not done the language and mathematics activities.

**Table 6:** Attendance at the intervention arm caregivers’ group meetings.

	Attended as a proportion of ideal (N = 3405)	Did language activity as a proportion of ideal (N = 3405)	Did mathematics activity as a proportion of ideal (N = 3405)
Mean (SD)	0.50 (0.37)	0.45 (0.35)	0.45 (0.35)
0%	838 (24.6%)	868 (25.5%)	868 (25.5%)
>0 and ≤ 25%	323 (9.5%)	411 (12.1%)	407 (12.0%)
>25% and ≤ 50%	419 (12.3%)	492 (14.4%)	513 (15.1%)
>50% and ≤ 75%	742 (21.8%)	788 (23.1%)	774 (22.7%)
>75% and < 100%	886 (26.0%)	714 (21.0%)	718 (21.1%)
100%	197 (5.8%)	132 (3.9%)	125 (3.7%)

### Primary outcome

**Fig 3:**
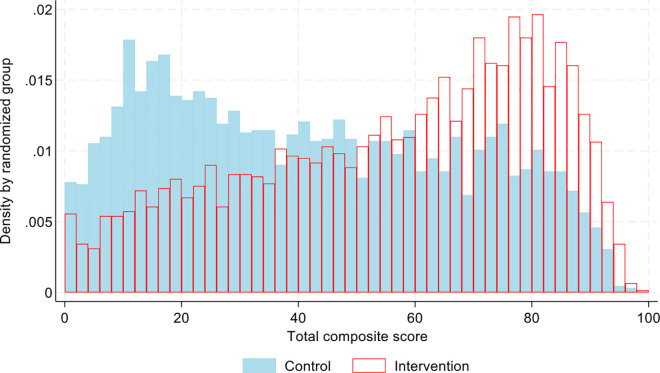
Distribution of child endline test scores by intervention arm.

The primary outcome from the endline test was obtained in 3054 out of 3405 children (89.7%) in the intervention arm and 3275 out of 3667 (89.3%) in the control arm. The primary outcome (composite score) and total scores for the reading (EGRA) and mathematics (EGMA) tests are displayed by randomization arm in Fig 3 with summary statistics shown in Table 7. On average, intervention children scored 14.17 percentage points higher than control children on the tests (95% CI 11.36, 16.97; p < 0.001), adjusting for the randomization stratification factors and including all children who did the test (even those who did not attend classes or attended very few of them). The standardized intention-to-treat estimate was 0.580 (95% CI 0.466 to 0.706). In the same table, we present the results for the total mathematics and reading scores separately. The differences between intervention and control children’s overall mathematics and reading test scores were similar to their difference in the primary outcome and both were highly statistically significant: 13.90 (95% CI 10.91 to 16.88) and 14.44 (95% CI 11.54 to 17.34) percentage points respectively, both p < 0.001. Results without the problematic item 5b were almost identical (Table S5 in S7 Appendix).

**Table 7:** EGRA and EGMA composite and separate total scores.

Variable	Intervention arm N mean (SD)	Control arm N mean (SD)	Difference (95% CI) p-value**
Composite test score*	3,054 56.30 (25.03)	3,275 42.25 (25.74)	14.17 (11.36, 16.97) p < 0.001
Composite test score* by attendance as a proportion of ideal			
0	280 44.01 (28.44)		
> 0 and ≤25%	536 44.14 (27.09)		
> 25% and ≤50%	397 50.46 (25.92)		
> 50% and ≤75%	622 56.65 (22.51)		
> 75% and <100%	806 63.64 (20.20)		
100%	413 71.17 (16.72)		
Reading test score	3,055 57.06 (25.25)	3,288 42.73 (25.95)	14.44 (11.54, 17.34) p < 0.001
Mathematics test score	3,058 55.52 (27.26)	3,282 41.75 (27.86)	13.90 (10.91, 16.88) p < 0.001

*Composite score presented only for children who took both of the tests (EGRA and EGMA)

**Estimated using linear regression with adjustment for the randomization stratification variables and using robust standard errors that take account of clustering.

Baseline characteristics in those children who did and did not take the test and contributed the primary outcome were similar (Table S4B in S7 Appendix) with minor differences being consistent in size and direction across the two trial arms (for example the percentages of children where the biological mother and father were not the primary caregivers were slightly higher when the test result was not taken in both trial arms). In the intervention arm the proportion of children with the primary outcome increased with increasing attendance at the before/after school classes (with 280/434 of those attending no classes contributing the primary outcome compared with 413/425 in those attending 100% of the classes, Tables 5 and 7). Our multiple imputation sensitivity analysis explored the potential for this and other differences between those with and without the primary outcome to introduce bias. With multiple imputation the estimated intervention effect was 14.04 (95% CI 9.17, 18.90; p < 0.001), adjusting for the randomization stratification variables, very similar to the result from the primary complete-case analysis.

### Per protocol analysis and dose-response relationship with class attendance

The per-protocol population was defined as the children enumerated in the intervention villages who attended more than 75% of the ideal number of before/after-school classes. There were 1259 (37%) such children in the intervention arm. The mean composite score was 66.19 (SD 19.42) among intervention children who attended more than 75% of before/after-school classes. The per-protocol estimate was 23.77 (95% CI 20.86, 26.68; p < 0.001) percentage points. In a *post-hoc* analysis we explored how the effect of the intervention varied according to attendance at the classes. The mean composite score in the intervention arm increased with increasing attendance (Table 7) being similar to that in the control arm when no classes were attended and rising to 71.17 when attendance was ideal. Using the structural mean model (SMM(G)) described by Maracy and Dunn [29] it was estimated that each 10 percentage point increase in attendance increased the composite score by 2.63 (95% CI 2.13, 3.16).

### EGRA and EGMA subtasks

Table 8 shows scores for each EGRA subtask, with the average percent correct for all subtasks, and the fluency scores for timed subtasks. Intervention children outperformed control children in reading in all subtasks, and the control-intervention difference was at least 10 percentage points (out of 100) for each. Children in the intervention arm demonstrated higher reading skill mastery across subtasks of all difficulty levels.

**Table 8:** EGRA subtasks and fluency scores.

Subtasks (item No)	Intervention arm N mean (SD)	Control arm N mean (SD)
Letter recognition (1)	3,055 51.80 (23.64)	3,288 40.11 (25.28)
Initial sound recognition (2)	3,055 51.27 (30.01)	3,288 41.93 (28.92)
Invented word reading (3)	3,055 40.24 (24.57)	3,288 28.39 (24.81)
Familiar word reading (4)	3,055 55.64 (35.02)	3,288 35.73 (34.75)
Passage reading (5a)	3,055 63.10 (35.97)	3,288 41.06 (37.77)
Reading comprehension (5b)	3,055 51.11 (35.75)	3,288 31.61 (34.69)
Reading comprehension (5b) – sensitivity analysis	3,055 55.16 (37.90)	3,288 33.92 (37.22)
Listening comprehension (6)	3,055 86.26 (23.24)	3,288 80.27 (27.21)
Letter recognition fluency score*	3,055 51.89 (24.03)	3,288 40.12 (25.45)
Invented word reading fluency score**	3,055 20.28 (12.76)	3,288 14.30 (12.82)
Familiar word reading fluency score^#^	3,055 32.01 (24.13)	3,288 19.92 (21.98)
Passage reading fluency score^##^	3,055 46.95 (33.18)	3,288 29.45 (31.47)

* Letter recognition fluency score – correct letters per minute: it is calculated as the number of correct letters per minute = letters correct × (60/ time elapsed in seconds). If the child did not finish reading the letters before time ran out, the score is the number of correct letters provided.

** Invented word reading fluency score – correct non-words per minute: non-words correct × (60/ time elapsed in seconds).

^#^ Familiar word reading fluency score – correct words per minute: words correct × (60/ time elapsed in seconds).

^##^ Passage reading fluency score – Also called Oral Reading Fluency (ORF), and sometimes referred to as connected text words per minute: number of words read correctly × (60/ time elapsed in seconds).

Analogous results from the EGMA subtask are shown in Table 9. The mean scores for children in the intervention arm are higher for all mathematics tasks, and the differences are broadly similar in magnitude to the ones observed in the reading tasks.

**Table 9:** EGMA subtasks and fluency scores.

Subtasks (item No)	Intervention arm N mean (SD)	Control arm N mean (SD)
Number identification (1)^*^	3,058 70.14 (29.94)	3,282 54.21 (32.54)
Quantitative comparisons (2)	3,058 68.84 (32.61)	3,282 51.73 (36.10)
Missing number (3)	3,058 51.73 (28.07)	3,282 39.25 (26.80)
Addition level 1 (4a)^**^	3,058 52.16 (30.50)	3,282 40.28 (31.59)
Addition level 2^*^ (4b)^***^	3,058 55.58 (38.32)	3,282 37.96 (37.15)
Subtraction level 1 (5a)^#^	3,058 37.67 (23.83)	3,282 27.81 (24.40)
Subtraction level 2^*^ (5b)^##^	3,058 44.98 (37.52)	3,282 28.80 (34.88)
Word problems (6)	3,058 47.20 (30.83)	3,282 37.88 (29.63)
Number identification fluency score	3,058 23.11 (18.29)	3,282 16.38 (16.66)
Addition level 1 fluency score	3,058 10.90 (6.51)	3,282 8.49 (6.68)
Subtraction level 1 fluency score	3,058 7.81 (4.84)	3,282 5.92 (5.16)
Subtraction level 2 (5b)	3,058 44.98 (37.52)	3,282 28.80 (34.88)

* Number identification, subtask 1 - In this subtask, the child is asked to read each number aloud from a grid with 20 numbers (one, two and three-digit). The assessor marks the number of correct numbers read in one minute. If the child does not finish before time runs out, the score is the number of correct numbers provided.

** Addition level 1, subtask 4a - In this subtask, the child has 20 simple addition questions to solve in one minute. If the child hesitates for 5 seconds, the assessor provides the answer and marks item as incorrect. If a child makes 4 successive errors, the assessor stops this subtask and moves to the following subtask.

*** Addition level 2, subtask 4b - This subtask is conducted only with children who answered at least one item correctly in subtask 4a (Addition level 1). Children who do not answer one such item correctly in subtask 4a are given a score of zero on subtask 4b.

# Subtraction level 1, subtask5a - In this subtask, the child has to answer 20 simple subtraction questions in one minute. It has the same rules as those described above for Addition level 1, subtask 4a.

## Subtraction level 2, subtask5b - This subtask is conducted only with children who answered at least one item correctly in subtask 5a (Subtraction level 1). Children who do not answer one such item correctly in subtask 5a are given a score of zero on subtask 5b.

### Subgroup analyses

The results of subgroup analyses of the primary outcome by pre-specified variables of interest based on characteristics of the child and village are shown in Table 10. Numbers of observations, means and standard deviations are given by trial arm for each level of the moderators. As per our statistical analysis plan, we considered male caregiver education, rather than male caregiver literacy, as a potential moderator due to the large amount of missing data for the latter variable. The intervention effect was broadly consistent across subgroups, albeit with a suggestion of larger effects in poorer households. In particular, there was statistically significant evidence at the 1% level of a differential impact of the intervention by wealth and female caregiver literacy. Our results suggest that the positive gain in learning was higher among children from poorer households (as per the materials households are made of – wealth index 1). The children whose female caregivers were less literate also had higher gains compared to children of female caregivers who could read the entire sentence. A smaller difference is seen between the intervention effects in boys and girls, where the positive impact of the intervention seems higher in girls, albeit with the p-value being above the threshold (p = 0.01) we used for these interaction tests. There is also a suggestion of a larger difference in smaller villages: the 95% confidence interval here is wide because this is a completely between cluster comparison. In a *post-hoc* analysis there was no evidence that the intervention effect was different when there was a pregnancy in the household.

**Table 10:** Composite test scores by subgroup, with interaction tests.

Moderator variable Subgroup	Intervention arm N: mean (SD)	Control arm N: mean (SD)	Difference (95% CI)***	Interaction p-value****
Village population				
Below median	1213 54.15 (25.36)	1241 37.36 (25.41)	16.69 (11.66, 21.73)	p = 0.174
Above median	1841 57.72 (24.72)	2034 45.24 (25.49)	12.56 (9.38, 15.75)	
Gender				
Male	1579 56.16 (25.58)	1668 43.76 (25.67)	12.61 (9.54, 15.67)	p = 0.023
Female	1475 56.45 (24.44)	1607 40.69 (25.74)	15.79 (12.60, 18.97)	
Wealth index 1*				
House made of all natural materials	155 55.78 (25.64)	116 39.13 (25.73)	17.21 (10.63, 23.79)	p < 0.001
House made of natural/synthetic materials	2062 54.34 (24.84)	2282 38.20 (25.04)	16.08 (13.09, 19.07)	
House made of all synthetic materials	780 62.90 (23.60)	795 54.92 (23.52)	8.63 (5.12, 12.14)	
Wealth index 2 (items owned)**				
0	1156 50.05 (25.52)	1257 34.88 (24.14)	15.10 (11.66, 18.53)	p = 0.583 (trend test)
1	908 57.31 (23.72)	1006 43.63 (24.89)	13.75 (10.44, 17.05)	
2	637 66.42 (20.79)	656 53.64 (24.43)	13.29 (10.21, 16.38)	
3 or 4	142 71.69 (19.56)	110 58.70 (23.83)	13.31 (7.70, 18.92)	
Caste				
Scheduled Caste	654 56.31 (24.41)	733 42.16 (23.75)	14.21 (10.24, 18.17)	p = 0.411
Scheduled Tribe	851 43.11 (24.33)	974 27.38 (21.14)	15.56 (11.53, 19.60)	
Other Backward Caste	1204 61.57 (23.17)	1158 48.66 (24.61)	13.06 (9.81, 16.31)	
Forward Caste	323 71.06 (18.56)	377 60.34 (23.35)	11.23 (7.09, 15.37)	
Female caregiver literacy				
Can’t read	1370 48.53 (25.41)	1498 33.71 (23.48)	14.90 (11.53, 18.27)	p = 0.010
Can read part of the sentence	526 57.79 (22.82)	594 41.43 (24.60)	16.32 (13.01, 19.63)	
Read entire sentence	917 67.87 (20.45)	877 57.23 (23.33)	10.88 (8.33, 13.44)	
Male caregiver education				
Illiterate	433 41.49 (25.63)	540 28.01 (21.73)	13.16 (8.82, 17.51)	p = 0.263
Primary school	538 47.72 (24.47)	569 32.73 (22.86)	15.16 (11.15, 19.17)	
Middle school	888 55.12 (23.73)	946 40.96 (24.03)	14.29 (11.26, 17.33)	
High school	744 65.16 (21.03)	720 51.35 (24.29)	14.03 (10.80, 17.26)	
Higher secondary school	246 71.09 (19.08)	276 60.09 (22.59)	11.28 (7.20, 15.36)	
Graduate	88 73.87 (19.08)	102 66.78 (20.19)	7.05 (1.11, 12.99)	
Postgraduate	47 76.19 (15.54)	26 70.04 (20.96)	6.50 (−5.54, 18.54)	
Pregnancy in Household (*post-hoc* analysis)				
No	2495 56.71 (25.03)	2707 42.99 (25.56)	13.86 (11.12, 16.59)	p = 0.287
Yes	559 54.49 (25.00)	568 38.73 (26.36)	15.76 (11.35, 20.17)	

*Wealth index 1 was extracted from a categorical measure defined by the materials of the roof, walls, and floor of the child’s home in the last year of the trial. Category 1 is that all materials are natural (e.g., a thatched roof, mud walls, and an earthen floor); category 2 is that some but not all materials are synthetic (e.g., a steel roof, but natural walls and floor); category 3 is that all materials are synthetic (e.g., a steel roof, brick walls, and a tile or concrete floor).

**Wealth index 2 is the number of items (television, radio, motorbike, 4-wheeled vehicle) owned by the household members.

***Estimated using linear regression with an interaction term between moderator and treatment allocation and adjustment for the randomization stratification variables, and using robust standard errors that take account of clustering.

****For moderators with three or more categories p-values are from joint tests of differences amongst all of the categories unless otherwise indicated.

### School enrolment and attendance

School enrolment is presented in Table 11 School enrolment levels were high for both intervention and control children in the academic years of 2019−20, 2021−22, and 2022−23. Differences between the percentage of enrolment between control and intervention arms were small at all three time points, albeit statistically significant at endline (2022−23), the pre-specified hypothesis testing time.

**Table 11:** Children’s enrolment in school.

Variable	Intervention arm N = 3405	Control arm N = 3667	Odds ratio (95% CI) p-value**
Midline – pre lockdown*			
Yes	2,992 (87.9%)	3,115 (84.9%)	
No	59 (1.7%)	148 (4.0%)	
Missing	354 (10.4%)	404 (11.0%)	
Midline – post lockdown*			
Yes	2,955 (86.8%)	3,104 (84.6%)	
No	96 (2.8%)	159 (4.3%)	
Missing	354 (10.4%)	404 (11.0%)	
Endline*			
Yes	3,076 (90.3%)	3,234 (88.2%)	1.38 (1.04, 1.83)
No	84 (2.5%)	121 (3.3%)	p = 0.025
Missing	245 (7.2%)	312 (8.5%)	

*Midline-pre lockdown refers to the academic year of 2019–2020, whereas post lockdown refers to the short period in which schools reopened (between November 2021 and March 2022). These data were collected during the midline survey. Endline refers to the academic year that started in June 2022.

**Odds ratio compared those responding “yes” and “no” to the question about enrolment in school, ignoring missing data. The odds ratio is estimated using a GEE model with a binary outcome, a logit link, and a ‘working’ assumption of independence, with adjustment for the randomization variables, and using robust standard errors that take account of clustering.

At the endline survey, caregivers were asked to report the number of days children had missed in school over the past two weeks (relative to the day of the interview). Attendance reported by the caregivers was similar for both arms (intervention arm mean 2.81 days missed: control arm mean 2.89 days missed: adjusted difference −0.10 days, 95% −0.40, 0.20; p = 0.492, full details in Table S10 in S7 Appendix).

### Learning support

There was no evidence (Table 12) that caregivers in the intervention arm gave more time to support their children with learning-related activities, despite efforts from the intervention teams to engage caregivers (primarily mothers) in their children’s learning (through home visits, telephone messages and caregivers’ meetings).

**Table 12:** Learning support (endline).

Variable	Intervention arm N = 3405	Control arm N = 3667	Difference (95% CI) p-value*
Caregivers’ help with schoolwork			
No	884 (26.0%)	895 (24.4%)	
Yes	2,188 (64.3%)	2,319 (63.2%)	
Missing	333 (9.8%)	453 (12.4%)	
Caregivers’ help with reading or counting activities to promote learning			
No	2,073 (60.9%)	2,274 (62.0%)	
Yes	999 (29.3%)	940 (25.6%)	
Missing	333 (9.8%)	453 (12.4%)	
Hours per week (ignoring missing) of caregivers engaging with child in reading or counting activities to promote learning (mean; SD)	1.25 (2.12)	1.19 (2.20)	0.08 (−0.17, 0.32) p = 0.530

*Estimated using linear regression with adjustment for randomization variables, and using robust standard errors that take account of clustering.

### Midline test and survey results

Table 13 shows the results of the midline tests. In these ASER-like tools, the tasks increase or decrease in complexity depending on whether a child can comfortably solve a given task according to the rules of the test (S4 Appendix). The midline tests’ results show that the differences in basic literacy and numeracy were statistically significantly higher among children in the intervention arm. In mathematics, children in the intervention arm scored significantly higher than those in the control arm. In the most difficult task, subtraction with borrowing, there were 13 percentage points more children in the intervention arm who could solve two operations correctly. The results in the reading test are sizeable, with 39% of the intervention children reading a grade 2 level short story (i.e., fluently and with ease at a good pace and making 3 or fewer mistakes) compared to 23% of the control arm children.

**Table 13:** Midline test results (using ASER-like exam).

Variable	Intervention arm N = 3405: N, mean (SD) or N (%)	Control arm N = 3667: N, mean (SD) or N (%)	Difference (95% CI) p-value*
Mathematics test score (in those taking the test)	2961 2.63 (1.30)	3171 2.04 (1.36)	0.60 (0.45, 0.74) p < 0.001
Beginner level	157 (4.6%)	374 (10.2%)	
Numbers 1–9	595 (17.5%)	1,036 (28.3%)	
Numbers 10–99	522 (15.3%)	559 (15.2%)	
Addition	611 (17.9%)	503 (13.7%)	
Subtraction	1,076 (31.6%)	699 (19.1%)	
Did not take test	444 (13.0%)	496 (13.5%)	
Reading test score (in those taking the test)	2961 2.67 (1.43)	3171 1.86 (1.51)	0.81 (0.65, 0.97) p < 0.001
Beginner level	255 (7.5%)	643 (17.5%)	
Letters	619 (18.2%)	1,118 (30.5%)	
Words	304 (8.9%)	274 (7.5%)	
Paragraph	455 (13.4%)	301 (8.2%)	
Story	1,328 (39.0%)	835 (22.8%)	
Did not take test	444 (13.0%)	496 (13.5%)	

*Estimated using linear regression with adjustment for randomization variables, and using robust standard errors that take account of clustering.

Table 14 shows access to technology, learning materials and help at home during the time schools were closed (due to COVID-19) for children in the intervention and control arms separately, whilst Table 15 shows the expenses related to schooling and learning done by caregivers during the first academic year that schools were closed. There were no large differences in caregivers’ support at home for studying. Most caregivers in both arms were helping their children to study at home. There were not many families who bought new devices in either arm. Most children were studying through textbooks and worksheets (many of them provided by the school) and had very limited access to tablets. The percentage of children with access to a smartphone was slightly higher among intervention children (24%) than control children (17%).

**Table 14:** Support during the COVID-19 schools’ closures (midline).

Variable	Intervention arm N = 3405 mean (SD)	Control arm N = 3667 mean (SD)
Help for home study		
No	116 (3.4%)	339 (9.2%)
Yes	2,933 (86.1%)	2,923 (79.7%)
Don’t know	2 (0.1%)	1 (0.0%)
Missing	354 (10.4%)	404 (11.0%)
Home devices		
Regular phone bought	81 (2.4%)	59 (1.6%)
Smartphone bought	221 (6.5%)	179 (4.9%)
Tablet/computer bought	3 (0.1%)	2 (0.1%)
Access at home to		
Regular phone	445 (13.1%)	198 (5.4%)
Smartphone	804 (23.6%)	619 (16.9%)
Tablet/computer	13 (0.4%)	3 (0.1%)
Educational videos etc.	1,134 (33.3%)	768 (20.9%)
Textbooks or worksheets	2,974 (87.3%)	3,017 (82.3%)
Support from schools		
Learning materials/activities	2,553 (75.0%)	2,461 (67.1%)
Child’s progress/well-being	1,784 (52.4%)	1,418 (38.7%)
Administrative information	1,395 (41.0%)	1,120 (30.5%)

**Table 15:** Adult caregiver spending (INRs) on education between July 2020 and June 2021.

	Intervention arm N = 3405 mean (SD)	Control arm N = 3667 mean (SD)
School materials	637.5 (935.5)	655.9 (998.9)
School fees School fees	1093.7 (2702.8)	1120.1 (3221.6)
Out of school tuition	311.3 (959.5)	412.6 (1150.5)
Other	351.3 (1696.1)	348.2 (1820.1)
Total	2393.8 (4234.7)	2536.8 (5057.4)

Information on spending was missing for 354 children in the intervention arm and 404 children in the control arm.

### Costs and cost-effectiveness

The primary expenditures of the intervention program along with their corresponding percentages are presented in Table 16. Forty-three percent of expenditures were on teaching staff (PIs) and 20% of spending covered cluster supervisors and manager salaries. Although PI salaries accounted for a large portion of the expenses, the net amount they received per month was 4,362 INR, in line with the government’s salary guidelines for half-time, unskilled labor. This amount is substantially lower than what local government teachers earn. For example, a third-grade teacher receives a minimum salary of 25,000 INR per month plus dearness allowance during their two-year probationary period, with the total nearly doubling after probation. There were minor capital costs (laptops and tablets), and these were fully depreciated over the three years of the trial. Since the program had been previously developed by the implementing partner, Pratham, only minor costs related to the development and piloting of the activities and materials for the before/after school classes were included.

**Table 16:** Costs of intervention program by item.

Category	Cost (INR)	Percent of total cost
Pratham Instructor (Part-Time)	25,999,948	43.0%
Program Head/Content Associate/MME Associate/Cluster Leader	11,884,951	19.6%
Training of Pratham personnel	2,860,800	4.7%
Materials for children and caregivers	1,753,319	2.9%
Office Rent	2,691,064	4.4%
Measurement, monitoring, evaluation	3,747,525	6.2%
Staff travel	1,034,063	1.7%
Overhead, equipment and other	10,535,377	17.4%
Total	60,507,047	100.0%

The main components of Pratham’s education program were highly affected by the disruptions caused by COVID-19, thus, in our primary analysis we calculated the cost per child excluding the ten months and a half period in which the before/after school classes could not be conducted. Excluding the two periods of interruption (mid-March to October 2020 and April to June 2021) and converting all the prices to 2021 prices using the India GDP deflator, the cost per child was 13,631 INR (184 USD) and the cost per 0.1 SD improvement in learning was 2,476 INR (33.5 USD). Equivalently, the intervention yielded a gain of 0.299 per $100 spent.

Using the same price conversion, and accounting for the period PIs could not conduct before/after school classes due to COVID-19 restrictions, the cost per child in 2021 values would be 20,213 INR (279 USD), and the cost per 0.1 SD improvement would be 3744 INR or 50.66 USD (a gain of 0.197 SD per $100 spent).

## Discussion

In this study we addressed the potential for improving foundation skills in reading and mathematics among children in their first years at primary school in rural villages of Madhya Pradesh, India. We showed that this strategy was very successful, with children in the intervention arm scoring materially better in all reading and mathematics subtasks of EGRA and EGMA tests. In the composite score, the difference was 14.17 percentage points and highly statistically significant (95% CI 11.36, 16.97; p < 0.001).

The main components of our intervention were: (i) supplementary classes (either before or after school) provided by a female para-educator (PI) from the community (ii) involvement of caregivers in their children’s education (through proposed activities via mobile text messages, home visits by para-educators and biweekly meetings), and (iii) implementation of teaching-learning methods and materials together with frequent training of PIs.

Supplementary teaching with para-instructors and volunteers have shown large effects: increasing literacy and numeracy among primary-school age children in India, The Gambia, China and Chile [11,17,18,24,35,36]. Experiences in India and The Gambia showed that para-teachers (or in some cases volunteers) who receive regular monitoring, coaching and are trained on structured lessons are highly effective [11,17,24]. This is likely to be because para-instructors, like contract-teachers, feel highly motivated due to their short-period contracts and tend to be more open-minded about new educational methods [17,24,37]. On the other hand, implementing regular extra classes outside school can be challenging, especially with young children in rural settings. In fact, this was one of the limitations of our study, as discussed below.

The second important strategy of our intervention was the frequent and in-depth interaction between PIs and the main caregiver. The main purpose of these interactions was to promote an environment at home to boost learning through simple activities tailored to the child’s learning level and get regular feedback on the child’s progress. This was done through daily mobile messages, weekly home visits and biweekly group meetings. Evidence shows that the home learning environment is important to foster children’s literacy and numeracy development [38–40]. A study targeting mothers in rural India, found that mathematics skills of young children as well as other aspects of home learning environment improved when mothers (some illiterate) were encouraged and guided to help with children’s learning [38]. Parents’ involvement in their children’s education became even more important when face-to-face contact was restricted due to the COVID-19 pandemic. In fact, exchanging phone messages became the backbone of the ongoing interactions in several programs implemented by Pratham during 2020 [41]. Our results suggest that caregivers in the control and intervention arms spent similar amounts of time engaging with children while studying at home (Table 12). However, this does not imply that encouraging and guiding caregiver-child interactions has no effect on learning outcomes. The impact may lie in the quality of the engagement rather than the amount of time spent with the child.

A key aspect of the intervention was the pedagogical approach of “teaching-learning method”, which is designed specifically for early education (Grades 1 and 2). This method goes beyond the curriculum content and emphasizes also the child’s emotional, social, and physical development. The PIs began classes by discussing with the children their recent experiences and the emotions associated with them. After this, the children participated in physical activities and engaged in group tasks to develop their social skills.

Important methodological strengths of the study are its randomized design, large size, careful implementation, and thorough data collection (with a rigorous follow-up of the trial children done by independent teams) (see S6 Appendix). The high proportion of randomized children taking the endline test (close to 90% in both arms) is a major strength. The fact that this proportion was very similar in the two arms (as was data collection in general) suggests that simultaneously carrying out the STRIPES2 and CHAMPION2 trials was successful in encouraging STRIPES2 control children and their families to contribute data.

It is theoretically possible that CHAMPION2’s monthly health promotion activities, such as participatory women’s groups, may have raised awareness around maternal and child health, hygiene, or caregiving practices at the community level. Because all STRIPES2 control villages received the CHAMPION2 intervention this cannot be investigated empirically, but we believe that due to the focus (pregnancy and neonatal care) and low frequency of these activities, it is very unlikely that they influenced parents’ behaviour related to early childhood education or cognitive development. It is also theoretically possible that children in those households where a woman became pregnant and received CHAMPION2 services benefited from improved caregiver knowledge, more caregiver free time and reduced stress. To investigate this possibility, we carried out a (post-hoc) analysis investigating the extent to which the effect of the STRIPES2 intervention differed according to whether or not the child’s household had active participation in CHAMPION2 through a household member becoming pregnant. The estimated interaction effect was small and not statistically significant, suggesting that the simultaneous implementation of the CHAMPION2 intervention in the STRIPES2 control arm did not materially bias results.

We had low attrition rates, and they were very similar in the control and intervention arms. Our primary outcome (EGRA and EGMA composite test score) was missing for about 10% of the children in the intervention (351/3405) and 11% in the control arms (392/3667). The results of the sensitivity analysis using multiple imputation and the primary analysis were very similar, with the estimated intervention effect being around 14 percentage points in both analyses.

Interestingly, our subgroup analysis (Table 10) reveals that the intervention has a somewhat larger impact on children coming from poorer families and those with female caregivers with lower literacy levels. Similarly, a randomized controlled trial that offered community-based preschools in Mozambique found larger effects on primary school enrolment and learning outcomes for children from poorer households [42].

The study has a few key limitations. It was not possible to blind the participants due to the nature of intervention. However, outcome assessors who administered the EGRA and EGMA tests were blinded. Secondly, we do not know if the effect of the intervention persisted after the intervention was completed as our study did not continue to conduct further follow-up.

The supply of Pratham Instructors (PIs) was a major challenge. PIs should ideally be from the local area to ensure a deeper understanding of the community’s needs and culture. Throughout the trial many PIs married and were absent due to maternity leave, which significantly disrupted class schedules, and finding a substitute for these PIs proved to be a complex process. Moreover, this significantly added to the cost of the intervention.

Another limitation is the relatively low adherence to the intervention. Just 37% of children in the intervention villages attended 75% or more of the ideal number of before/after-school classes (our definition of high adherence, as defined prior to the start of the statistical analysis). Many children could not come to the classes because the venue where these before/after-school classes were conducted was too far from their houses. Even though the Pratham team had access to a detailed map with the location of all participants’ households (prepared by GHTC), it was challenging to identify a spot that would be easily accessible by all the participants. In rural Madhya Pradesh, households are typically highly dispersed within villages. Pratham instructors anecdotally reported that exhaustion was also a reason given by some caregivers to explain why some children were not coming to classes. They also reported that in some villages, it was difficult to find a time when all the children could attend the before/after school classes. Our findings suggest that the magnitude of the impact could have been greater with improved adherence to the before/after-school classes. The per-protocol analysis shows that children in the intervention villages who attended more than 75% of before/after-school classes had a mean score of 66.19 (SD 19.42), with a treatment difference that was about ten percentage points higher than that in the intention-to-treat results.

We cannot completely rule out the possibility of spillovers through community meetings or shared resources in some villages, although we believe that this did not occur to any material extent. Such spillovers would tend to reduce the observed effects of our intervention, not increase it. However, Pratham took measures to try and ensure that control group children did not attend classes in intervention villages. Registers of children eligible to attend classes were maintained, and though on occasion additional children were allowed to attend classes, Pratham carried out visits to households to ensure that such children were resident in the intervention villages. Before randomization, we explored the possibility of having a larger distance between villages, but this would have drastically reduced the number of villages in the trial. Thus, the decision to go for 3 km buffer zones was pragmatic.

Finally, the COVID-19 pandemic caused considerable disruption to the original plans. In March 2020, all activities had to be suspended a few months after their launch in the communities because of the imposed severe lockdown due to COVID-19. India experienced “lockdown” and movement restrictions for several months in 2020 and in 2021, and schools remained closed for almost two years. Not all Pratham Instructors had been hired when lockdown was imposed and therefore in about 18 villages the activities started much later. Even when “lockdown” restrictions were loosened, activities such as school-going or attending neighborhood community-based classes did not resume immediately. These interruptions inevitably affected the process of learning.

### Cost-effectiveness

In the STRIPES2 trial, cost per child (13,631 INR, 184 USD) and cost per 0.1 SD improvement (2,476 INR or 33.5 USD) were substantially higher than the original STRIPES trial [18], where the cost per child in 2021 values was 4143 INR (56 USD), and the cost per 0.1 SD improvement was 557 INR or 7.54 USD. Equivalently, STRIPES yielded 1.326 SD per $100 spent, while STRIPES2 yielded only 0.29 SD per $100 spent.

This relatively lower cost-effectiveness observed in the STRIPES2 trial on children’s outcomes can be attributed to several factors. The higher costs primarily reflected substantially higher labor costs, as well as the additional costs added due to an extra effort by the Pratham teams to compensate for the learning losses during the time schools were closed when the contact with children and parents was limited to mobile text messages. These additional costs reflect both a greater outlay of raw resources in the STRIPES2 intervention, and a greater expense of operating in India (holding resource levels constant) today as compared to then, given India’s rapid development and steady inflation over the 13 years that have elapsed since the end of the original study.

The STRIPES2 program design was labor intensive as we needed to hire many Pratham Instructors. This was partly needed because of the trial structure, given that houses in our trial area were spread over a large area and therefore it was challenging to visit all families and ensure that all trial children would come to the before/after-school classes. The overhead costs for management of the trial could be reduced if implemented within the existing educational structures. For that, teachers need to be fully supported by the existing structures, have clear instructions to implement the methods and devote a time slot of the daily class to apply it. As an example of this approach, a randomized evaluation of a teacher incentive program [43] found children in the treatment groups, where teachers received incentives calibrated to 3% of salaries, scored 0.17 and 0.27 SD higher on language and math scores compared with controls respectively. For a teacher earning a salary of 600,000 INR per year, and teaching 20 students, these incentives would cost 1800 INR per student over two years, markedly less expensive than the costs per student reported for STRIPES 2. Another option is to train volunteers to lead the activities in the schools. These two models have proven to be the most effective when scaling up the “Teaching at the Right Level” methods [11]

The STRIPES2 trial was also costlier than a similar project implemented in Gambia (pre-COVID-19). Although the average cost per child (adjusted to endline test takers) in the SCORE trial in Gambia [24] was high, the improvement in test scores was greater, and the cost per 0.1 SD improvement was 25.59 USD, (i.e., 0.389 SD per 100 USD). In rural Gambia, learning levels and learning trajectories are much lower and flatter, respectively, than in India, allowing more room for improvement there. Therefore, the cost-effectiveness will vary also depending on the existing learning levels.

## Conclusion

STRIPES2 results showed that bundled interventions that combine community trained para-instructors, caregivers’ engagement in child’s learning and teaching-at-the-right-level methods can accelerate the end of learning poverty in areas where learning has been low and stagnant for many years. This is consistent with other studies that found that well-structured supplementary education programs can have a large positive impact on learning levels. The approach used in STRIPES2 could be applied in numerous other settings, in India and beyond, which closely resemble our trial area in terms of size, remoteness and level of services provided by the government. Although learning gains were not dramatic relative to the high unit cost which was driven by trial logistics and labor costs, the intervention may be sustainable if the package can be integrated into existing services, either in public schools by existing teachers or in the community by volunteers (unpaid).

## Supporting information

S1 Appendix Manual for instructors/teachers: warm up – phase I language & math class 1 & 2. (PDF)

S2 Appendix *Manmoji Ganit* booklet. (PDF)

S3 Appendix Manual for master trainers – grades 1 and 2. (PDF)

S4 Appendix Midline assessment. (PDF)

S5 Appendix EGRA and EGMA tests. (PDF)

S6 Appendix STRIPES2 data collection process. (PDF)

S7 Appendix Statistical analysis – full results. (PDF)

S1 STRIPES2 Analysis dataset Excel format. (XLSX)

S2 STRIPES2 Analysis dataset Stata format. (DTA)

S3 STRIPES2 Analysis Stata do file. (DO)

S1 File Inclusivity in global research questionnaire answered. (DOCX)
