# Supplementary material for: Support To Rural India’s Public Education System (STRIPES2) and impact on numeracy and literacy scores: A cluster randomized trial in rural villages of Madhya Pradesh, India
Source: PLoS One. 2025 Sep 12;20(9):e0330203. doi: 10.1371/journal.pone.0330203 (PMC12431668; doi:10.1371/journal.pone.0330203)
Supplement: S1 Appendix — (PDF) [file pone.0330203.s001.pdf]

Warm Up

Phase-I

Language & Math

Class 1 & 2

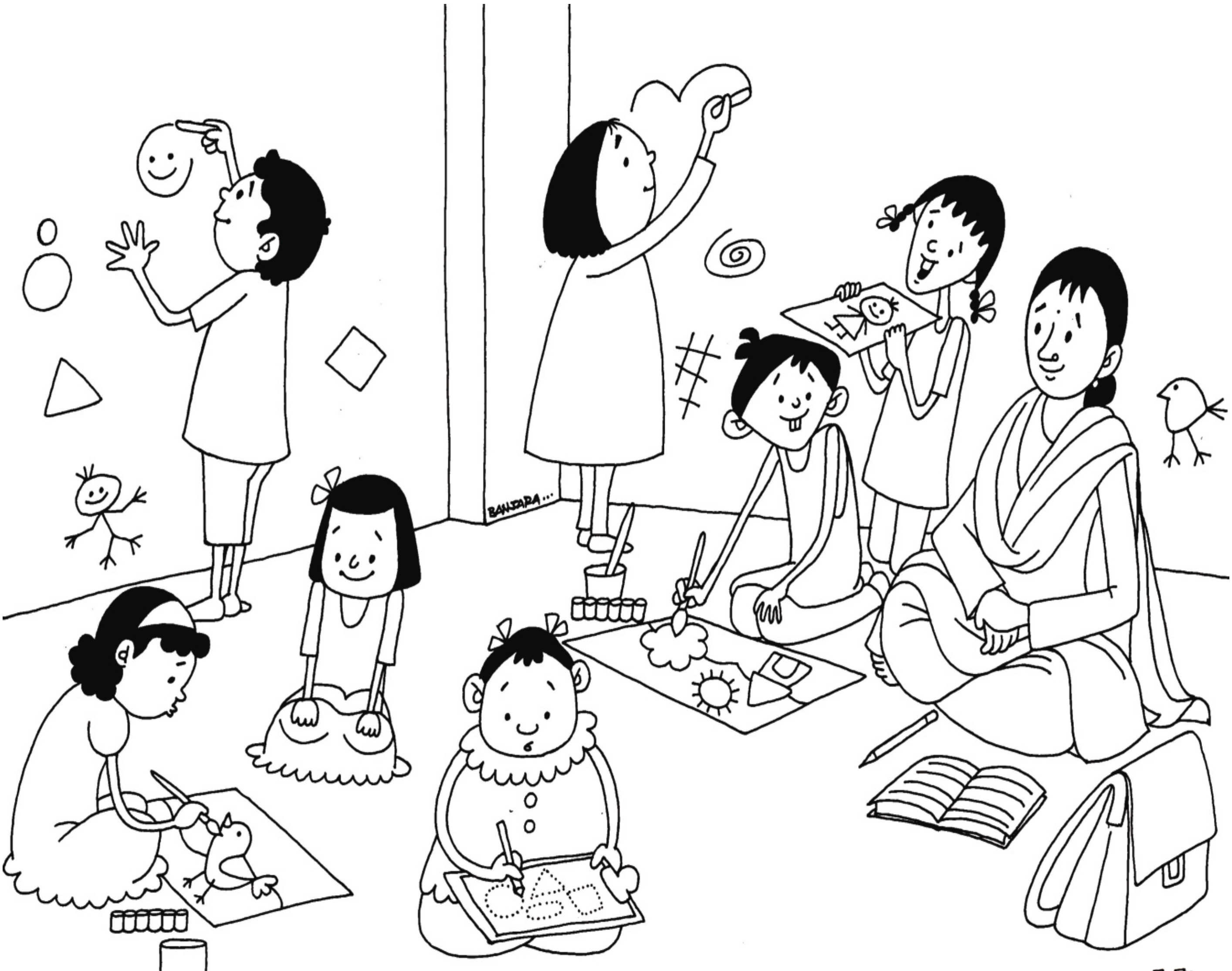

Pratham

Introduction

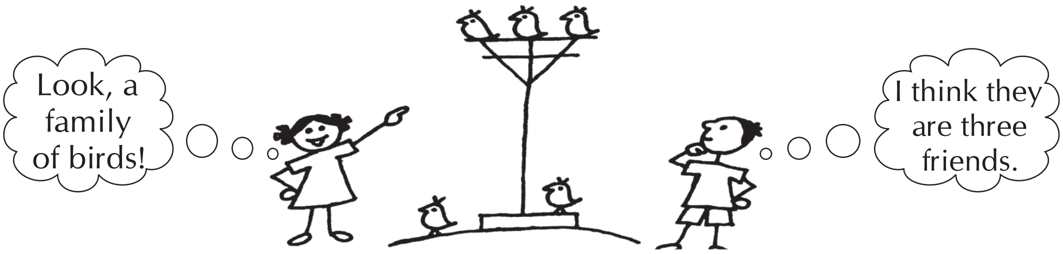

In schools, you will find 5-8 year old children either enrolled in grades 1-2 or about to be enrolled in the same. Often, we expect that by the end of grade 2, these children will be able to obtain basic language and math skills. Similarly, with grade 1, we expect some children to be competent enough to be able to learn reading and writing. Forming groups in class, interacting with other children, expressing themselves as well as listening to other children and teachers are some competencies that can be learnt at Aanganwadis and even at home with someone's help before coming to school. This also includes learning to control small and large muscles. Schools, society and children's homes have a major role in improving the quality of education that children receive. Additionally, learning is closely linked with opportunity and guidance. If children receive ample opportunities and guidance, they can deeply enhance their learning capabilities. Therefore, if they receive additional help from someone at their homes, it can only help improve their learning.

This manual is created keeping this concept in mind. Apart from learning reading and mathematical skills, some additional activities have been provided which will help children in their physical, emotional and social development as well. Do these activities with the children so that they can prepare for self-learning in a better way! Apart from these, include other activities which will help them in learning language and math. Seeing this as 'Warm-up Phase' (Official term) as a mere preparation would be a mistake. 'Self-learning' and 'learning in school' should not be seen as two different concepts. The 'Warm-up Phase' helps us realize that in learning, there is no need to force our experiences on them or to hurry with the process itself. We can also talk about 'what not to do' but for now, it is more important to be alert about 'what to do'.

- As a teacher, we should understand and accept that every child might have a different learning capacity and speed. Each child builds his knowledge in a different way. Therefore, we need to help each child, keeping these nuances in mind.
- Every child brings with him/her a certain kind of linguistic knowledge to class. Additionally, each child's knowledge can differ based on his/her experiences in life.
- We also need to help children understand that 'learning' essentially means enjoyment along with 'understanding meaning'.
- We hope that you will keep these pointers in mind while taking 2 hour classes every day.

Phase-I (Warm Up) - Goals and Daily Plans

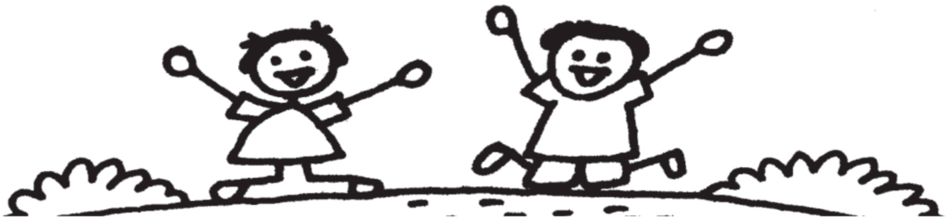

Language

- Introducing the book (Text formats)
- To be able to express themselves freely
- To be able to understand pronunciation of words
- To be able to understand sound symbols of letters
- To be familiar with the school, class and the teacher
- Active participation in teaching-learning process

<table>
<tr><th colspan="4">Activities to be conducted in class</th></tr>
<tr><th>Time</th><th colspan="3">Activities</th></tr>
<tr><td>10 minutes corner</td><td colspan="3">In the beginning of the class, ask the children to play their favorite game in the corner.</td></tr>
<tr><td>5-10 minutes Discussion</td><td colspan="3">Have a daily discussion with the children. The activities for discussion have been given.</td></tr>
<tr><td rowspan="3">15-20 minutes Story related (5-6 days on one story)</td><th>Day 1</th><th>Day 2,3,4,5</th><th>Day 6</th></tr>
<tr><td>• Conversation
• Story Telling
• Discussion- who, what</td><td>• Story telling in their own words.
• Discussion- questions- what, how, etc.
• Following the text with a finger while reading.
• Spoken words- finding, writing and reading.</td><td>• Repetition
• Role-play</td></tr>
<tr><td colspan="3">Base your activities on only one story for 3 days</td></tr>
<tr><td>Activities using a chart (10 minutes)</td><td colspan="3">Letter sound Activity :
• Have activities based on the chart in class every day.</td></tr>
<tr><td>Phonological Game (5 minutes)</td><td colspan="3">• With the help of word from the story, play sound games in class every day.</td></tr>
<tr><td>Letter Recognize game (10 minutes)</td><td colspan="3">• Make the children play letter sound identification games with help of 'Chalo Khelein' Activity Booklet.</td></tr>
<tr><td>Writing (5-10 minutes)</td><td colspan="3">• Daily opportunity to express their idea, understanding or view : Easy-Busy/Drawing Write about story.</td></tr>
</table>

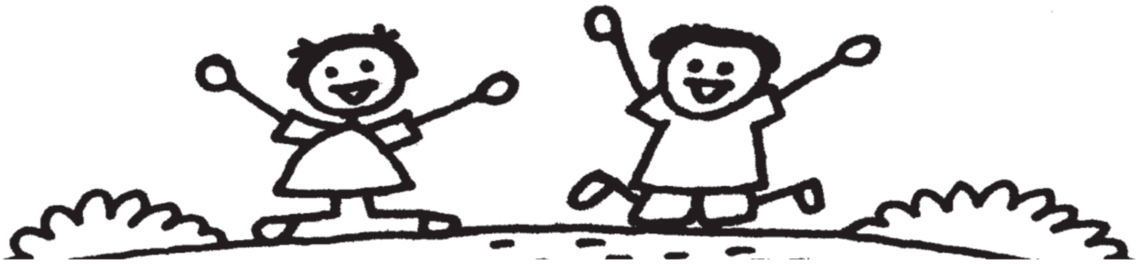

Corner-Free Games: Social, Emotional and Physical development

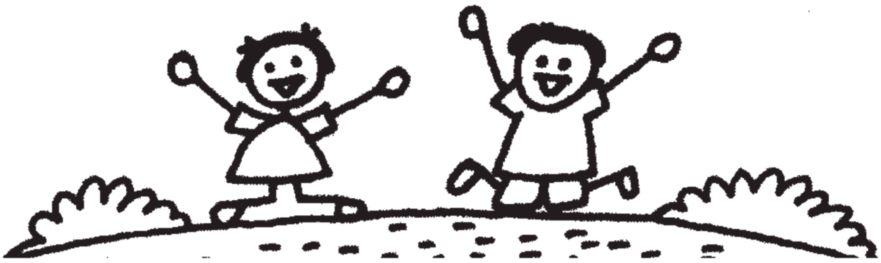

Preparing children for school is very important, but this preparation doesn't mean that they should be taught how to read and write. Focus should be on developing certain behaviors and habits at home, Aanganwadi centers and pre-primary learning centers. for example listening to others, being responsible, being able to hold a pencil properly, playing in a group, being happy, etc. But as a teacher, one should also keep in mind the importance of constantly working on developing these competencies. In class 1, apart from linguistic and mathematical competency, working on physical, social and emotional developments is also necessary. It is because there is a direct correlation between these factors and their ability to learn reading-writing as well as mathematics efficiently. Sometimes, for social, emotional and physical development in children, different kinds of activities need to be organized and other times, during some activities these developments get affected too. Therefore, in class 1 such activities should always be conducted which provide an opportunity for some kind of group or individual task.

Activities for Social and Emotional Development

Social and emotional development plays an important role in the development of the reading and writing skills of children in grades 1 and 2. Therefore, appropriate socio-emotional development activities should be organized with the children in these grades.

children should be given adequate opportunities to develop their socio-emotional skills through various activities/games in groups or pairs. Some examples of such activities are rhymes/poems/songs recitation, playing with sand, discussion, dancing, playing with dolls etc.

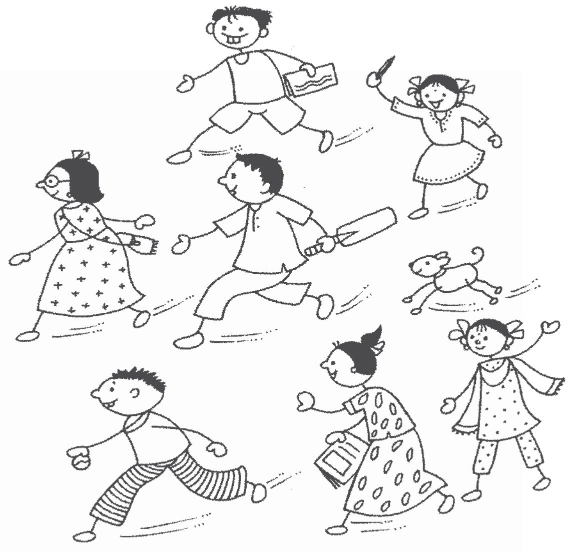

Physical Development

Before coming to grade 1, there should be an adequate development of the motor skills of children for example hand - eye coordination as well as the development of large body muscles. Sometimes, the child does not get enough opportunities to work on these abilities before coming to grade 1. Therefore, it is important to organize various activities like running, jumping, colouring, tearing a page etc to develop motor skills of children in grades 1 and 2.

Corner Free Games: Social, Emotional and Physical Development

How and what to do

In order to develop the social, emotional and physical competencies, provide the children freedom to play in the class before beginning with the language and math lessons. The free play will go on for 10-15 minutes in which children should be encouraged to participate in groups, pairs or even individually.

- Assign four corners in the class. Place different kinds of materials in each corner and allow the children to pick their favorite corner to play in.
- Similarly, for their physical and creative development, encourage them to explore the school ground and share their observations that they observe around them.

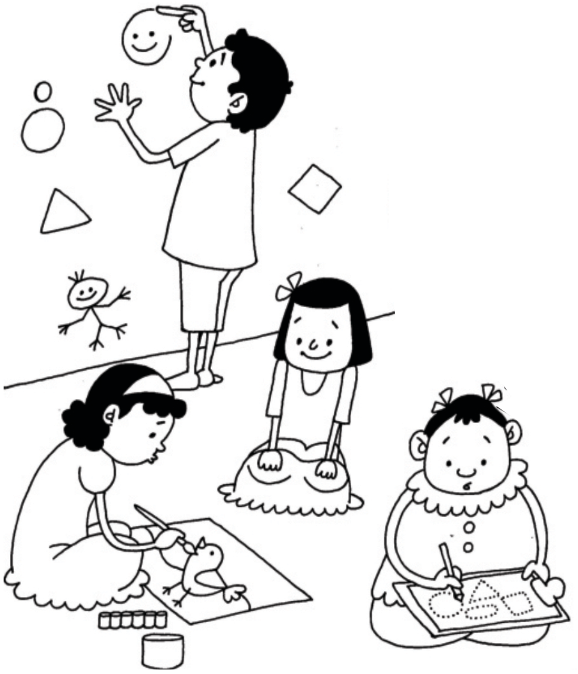

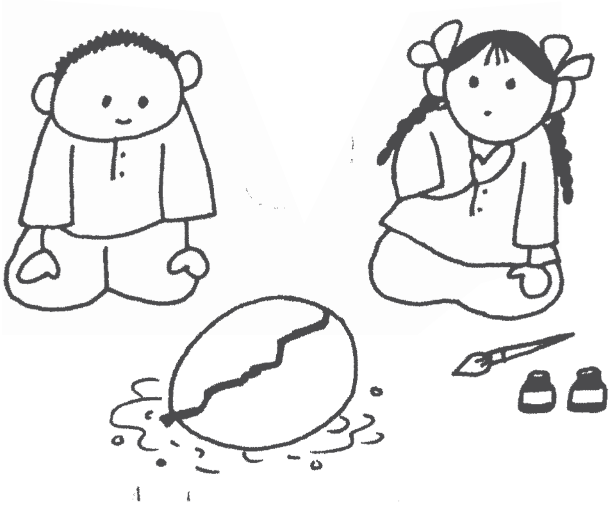

Note: In the class, the various kinds of activities that can be conducted in 'Corner Free Games' are given in the 'Let's Play Something Different' manual in detail. The manual also contains details about activities on physical development and school exploration. Conduct 1-2 activities with the children daily.

Things to remember:

- Don't suggest what the children should or should not play.
- Encourage the children to choose and sit in different corners everyday.
- During their playing time, you can roam around the class and check if they need help with anything.
- It is possible that the children may play by themselves initially but encourage them to participate in groups and pairs.

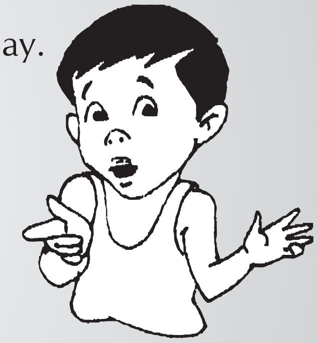

ORACY

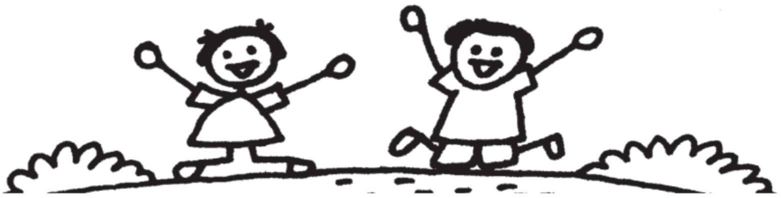

Children who get enrolled in classes 1 and 2 come with the knowledge of their native language. They already know how and when to use their language. Using their vocabulary, children are also able to create a large number of sentences according to their requirement or the situation they are in. This knowledge of how a language works comes from their experiences as well as the conversations that they make. In other words, a child's oral language development is based on his/her experiences. In the initial days, allow children to talk about their experiences in their native language so that they are able to use this language in various contexts very skillfully. By using language in these various contexts and situations, they obtain many experiences. These experiences then help them in oral language development. Therefore, **it is important for the teacher to listen and understand the language that his/her child uses in class** and to also respect it. The important thing is, that **expressing themselves after thinking helps children to learn reading-writing as well.** Hence, coming from different backgrounds, these children come to school with already existing vocabulary and sentence frameworks in their native language and learn a new language in school. Thus, in schools when their spoken language is given respect and they are given the freedom to use it however then working on both their language and learning school's language will become an enjoyable process.

If I had

In a large group, tell the children, **"Let's play a game. I will say something and you tell me if it had been you, what would you do?"**

For example- **"If you are in a garden and suddenly a monkey appears, what would you do?"** Let the children think for sometime then ask them what did they think and what would they have done? Some examples are, **'If you were a king/a queen/', 'I would visit the city'.... 'if it rains today then I/you....'** Etc.

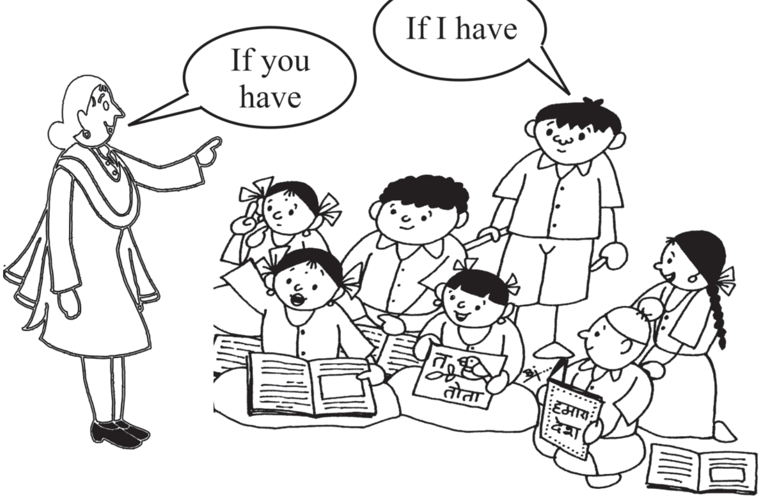

Discussion on a Picture

In a large group, show a picture to the children and talk about it. For example- "**What is there in the picture?**" and "**What is happening?**" Ask the children, "**What do you think what is the story of this picture?**" Give each child a chance to speak. In order to encourage what the children have to say, ask questions like, **"Oh! What do you think happened next?"** This way, while interacting with the children, a whole story can be formed orally.

Discussion on Topic

In a large group, pick any topic and start speaking about it and ask the children to share their thoughts on it too. For example**- 'book'**. First talk about a book yourself, **'A book is colorful'**. Then ask the children to think about the topic and speak.

1 minute talk

Make some small chits and write the name of/draw an object. For example- **Shoe, Bottle, House, Pencil,** etc. Then place the chits in between the children and ask each child to pick one. Ask the children to think about the object for a minute. Then ask them to talk about the object. If the children are facing difficulties in talking, ask the children questions related to the object. For example- **What is it? What does it look like? Where do we get it/find it? How would it be if it were not there?**

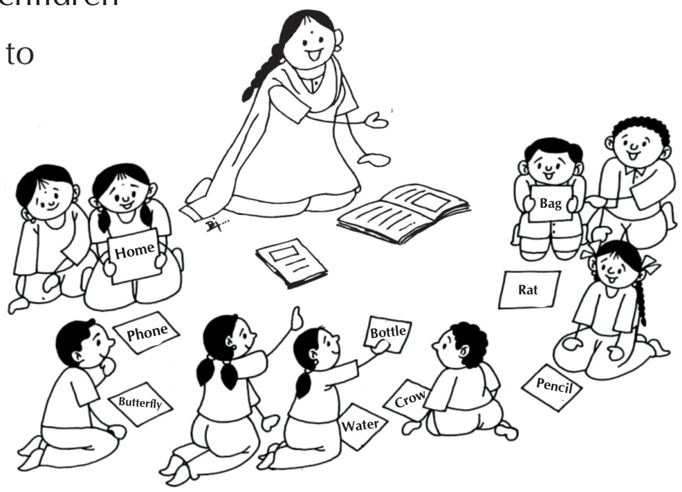

Things to Remember

- Talk about familiar and unfamiliar topics in class with the children daily.
- A discussion is not limited to just asking questions and giving answers. A good discussion includes the children talking about their experiences, and not just answering questions.
- Let the children use their native language during discussions. Listen carefully to what they have to say.
- Sometimes, in order to encourage the children to share their thoughts, pretend as if what they are saying is something you have never heard before or didn't know.
- Often use phrases like, **"Oh!/ What is that?/ How is it done?"** These phrases will encourage children to form answers on their own.

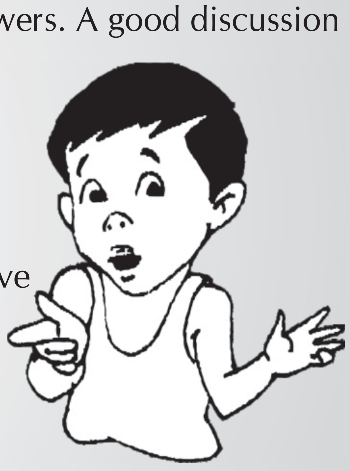

Story Related Activities

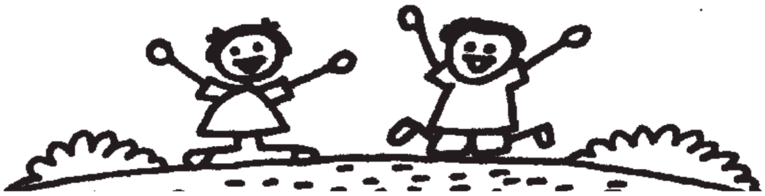

In order to attract children towards reading and writing, the teacher should read the stories from a book in the initial period. Stories help children to develop interest. Using books with the children helps them to relate their learning process with written language as well. Through the medium of a story, children understand that 'reading' is not just reading but understanding as well.

A story's title, pictures and its written language are all very important. Development of reading ability should be done by reading to the children from a book every day. Additionally, discussing things related to the story should be encouraged. With the help of stories, reading ability and understanding develops at a faster pace.

First Day

- Before reading the story, discuss the images and the title of the story. For example for a picture ask questions like- **"Who is there? What are they doing?"** etc. (Talking about the story helps children to guess its plot and also help then to try to relate it with their own lives.)

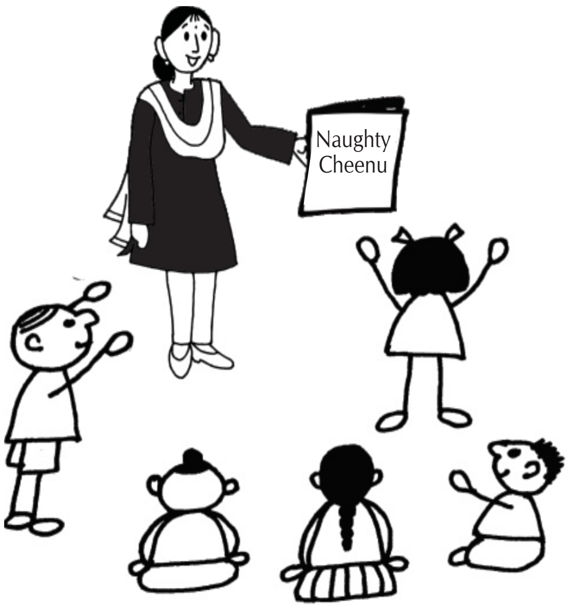

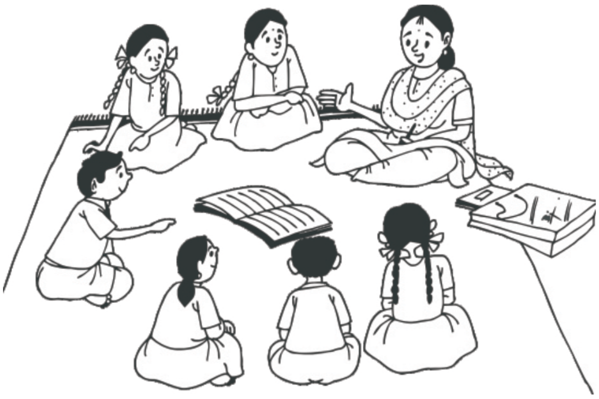

- Read the story in a clear voice with proper intonation. Make sure the children are listening the story carefully and not repeating the words after you.
- After reading the story, discuss about the story with the children, **"Who was there in the story? What happened in the story? Who did what and why?"** etc"

Second, Third, Fourth and Fifth Day

- Ask the children to narrate the stories which was read to them on Day 1 in their own words.
- Sometimes you can also ask them to play roles of the characters from the story.
- Give the children story books/cards. Then ask them to follow the words with their fingers while you read the story.
- Ask **"Who will read now?"** and let 1-2 children read

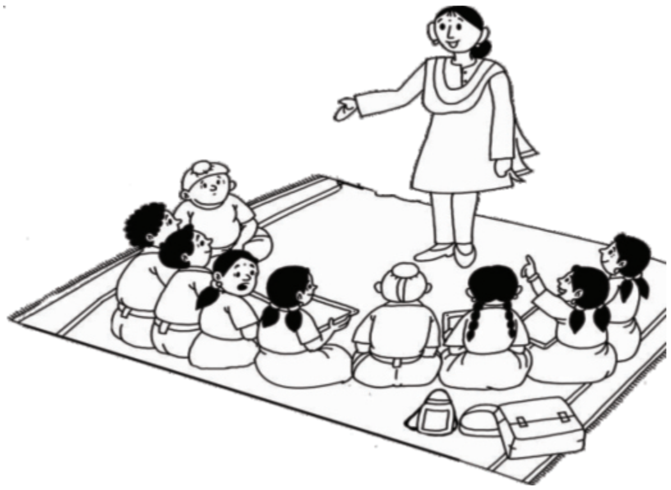

Sixth Day

- Make small groups in the class and prepare the children for role playing. Help the children with preparing dialogues and playing the characters. Conduct the role play activity with a big group.

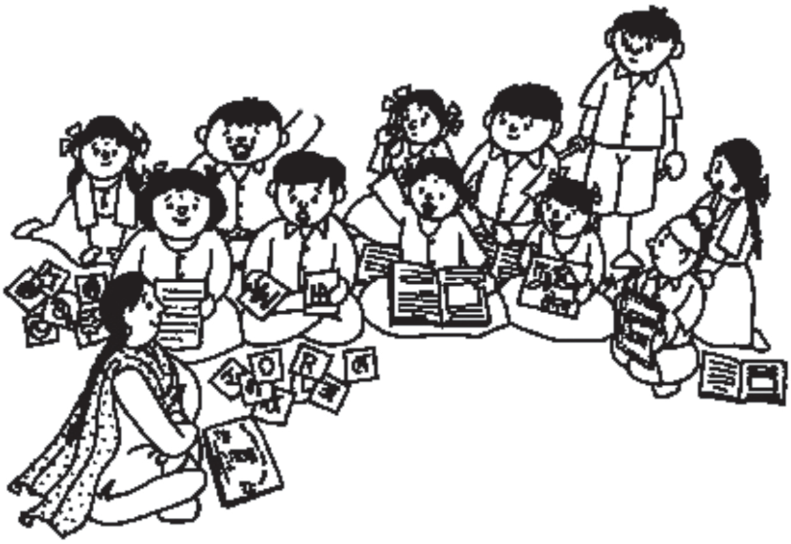

Note: During the story related discussion motivate children to share their experience & opinion.

Phonological Awareness : Sound Game

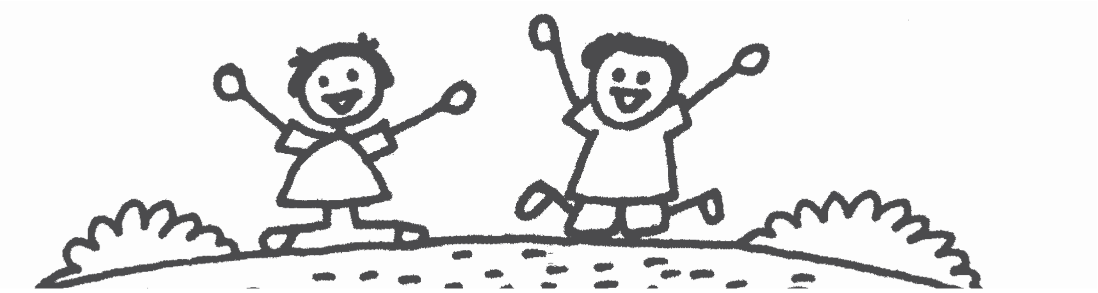

In order to recognize and understand various sounds in words and sentences, conducting sound-related activities becomes important. An understanding of breaking and combining sounds to make words helps in developing reading-writing abilities in a faster way. In order to master the understanding of sound, it is important to conduct different types of games in class. For example- Breaking words into different sound patterns, pronouncing different sounds to form words/sentences, understanding the first and last sound of a word, changing the first or last sound of a word to form a new word or even listening to a sound and guessing what word could it form.

Activities for introducing sounds:

Note: In order to introduce sounds, choose a set of words from a story beforehand.

- Say the word (clearly)
- Ask- How many sounds are there?
- What is the first sound?
- What is the second sound?
- What do we get after combining both the sounds? (With this way, play this game with different words.)

Things to Remember:

- Start with words having only 2 syllables.
- Always play the sound games orally so that the children understand that 'words' are made up of sound symbols.
- Sound games can be played using words from the story.
- Play this game with 3-4 words daily.
- Don't use compound words for this game.

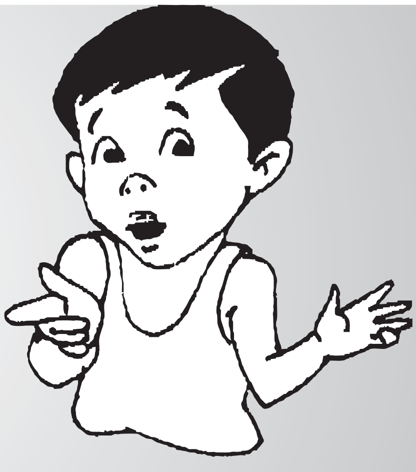

Letter : Chart Activity

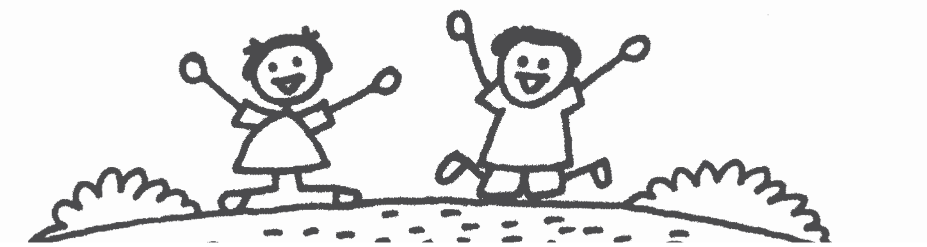

Each sound is also a symbol, it is important to understand in order to develop reading-writing abilities. Good understanding of the coordination of sound symbols makes it easier for children to identify and understand a word. Therefore, out of the given games, make the children play symbol-identification games daily.

Activities with alphabet chart/letter card

- Place your finger on each letter on the Alphabet chart and read it aloud slowly and carefully. Make sure that the children are listening and not repeating after you.
- In between, place your finger on a letter on the alphabet chart and ask the children to call out the sound/name of the letter.
- Give the letter card to each child. Then place your finger on each letter while reading it aloud while the children follow the letter on their cards with their fingers. Make sure they are not repeating the letter after you.
- After reading, ask the children, "Who will read now?" (Make 2-3 children read)
- Give the letter card to the children and read letters randomly. Then ask the children to identify the letter in their cards and write it in their notebooks/on the floor.

Note: After a few days, read out some simple words. The children will find the letters and write the words with it. For example- मदन, घर पर and बल etc.

Letter sound combination

- Write the letters introduced in class in a straight line and ask the children to tell you their name (sound).

For example- म
ख
र

- Add a symbol to the letters and then read. For example-

'म'	'मे'	मेल
'ख'	'खे	खेल'
'र'	'रे'	रेल

An Alphabet chart for the class and alphabet card for each children will be given .

Note: For beginners, conduct activities using the alphabet chart. If the children can recognize letters, then conduct activities with them using 'बारहखड़ी' chart.

Games for Letter-sound Association

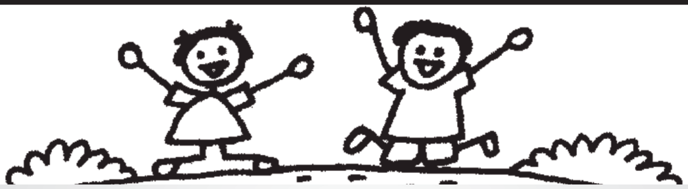

In order to improve the understanding of letters it is important to conduct activites which help in practicing the coordination of sounds and symbols of letters. This means that only learning the letter sounds or only learning how to write letters is not enough. The focus should be on understanding how letters and letter sounds work together, that is, finding the symbol for spoken letter symbol and looking at a symbol and telling the sound it will make.

Letter Identification Activities:

Conduct this activity using 3-4 letters daily.

Finding Letters

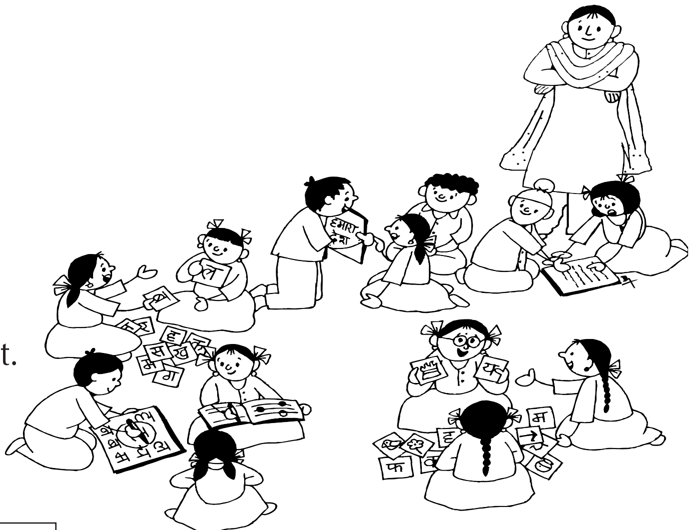

- Show the letter (flash card)
- Tell its name
- Ask the children to find the same letter
- Ask them to write the letter down once they find it.

Letter Jump

- Make the children sit in small groups of 2-3

म	क
र	ल
क	म

ल	र
क	म
र	ल

क	ल
म	र
ल	क

- Draw a box (Show an Below) on the blackboard
- Ask leader to copy same box on the floor.
- Write letters in each box
- Now start playing say the letter out loud
- The children will find the letter and jumping on by finger it.

Things to Remember

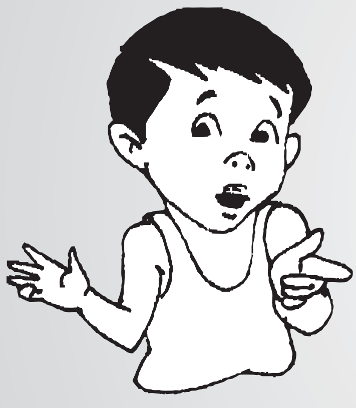

- The letters that are taken for these games can be taken from an order. Sometimes, we can also take letters which are not easy to recognize or which kept appearing in the stories.
- Use the letters used in these games to form words.
- In the initial days do not use letters whose sounds or symbols are almost the same so as to avoid confusion. For example-'ल' and 'ध', 'ट' and 'त', 'च' and 'छ', etc
- The letters used on a day should be revised the next day along with introducing new letters.

Writing

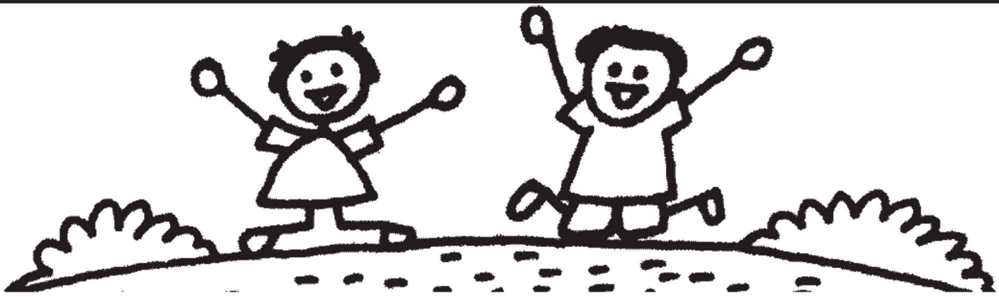

Encouraging children to express their ideas through writing with the help of a sheet of paper and a pencil is an important precursor to writing practice. Initially, the children may write in a way which makes it difficult to understand but a small instruction like, "draw anything" and asking, "What did you make?" makes the children think about the lines they have created and encourages them to use words to express their thoughts. Even though words are made up of letters and sentences are made up of words, writing doesn't mean only being skilled in making symbols and letters. Rather, writing actually means thinking about an idea and presenting it on paper. Often, children are unable to understand that putting into words what they think about an idea is writing only. Here words and sentences made out of symbols and letters play a huge role. Therefore, from the first day itself, ask the children to draw or write anything that they can after they listen to a story, even if they present their thoughts with the help of crooked lines. This activity is very important for the children to express themselves and also helps in developing their writing abilities.

Activities for Writing

Make/Write anything based on the story

After listening to the story and discussing about it, ask the children to draw a picture on basis the story. The picture can be made on notebook or even on the floor. Even if the children make straight or crooked lines, let them. Do ask the children, **"What have you made?"** If some children can write, let them write. Help them if they make mistakes.

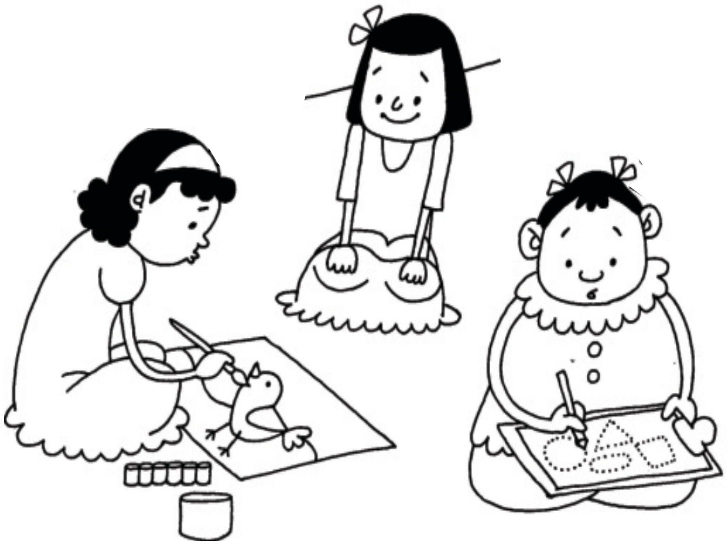

Writing by Looking

In a large group, give each child a chalk and ask, **"Draw on your notebooks or on the floor, what I am drawing on the board."** Write down the letters introduced in the class on the board. The children will try to copy the written letter.

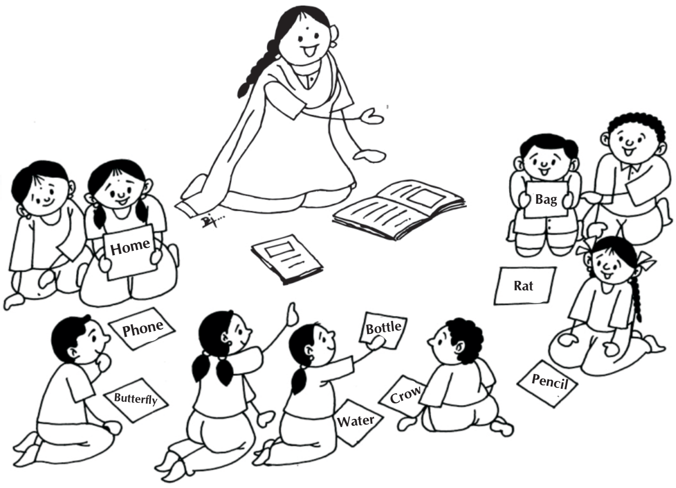

Writing by Listening

In a large group, give each child a chalk and say, "Listen carefully to what I am saying and draw it on your notebook/on the floor." Write down the letters introduced in the class on the board. The children will try to copy the written letter. (During this game, speak each letter clearly and loudly)

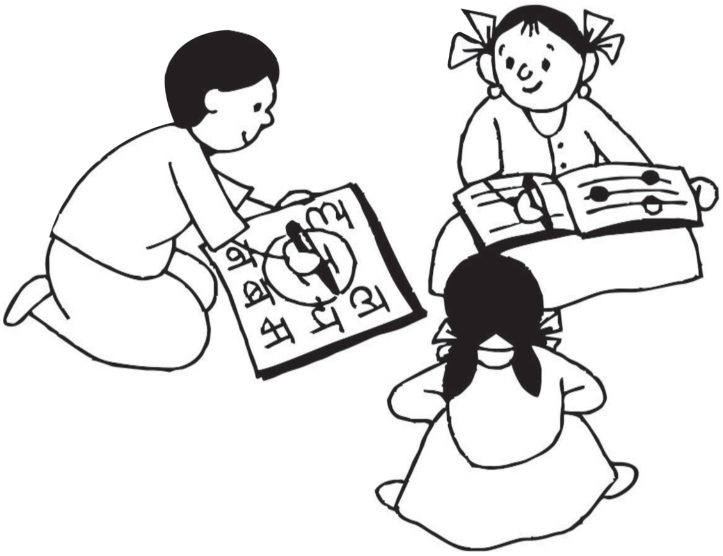

Things to remember:

- In the initial period, the children are only able to draw straight and crooked lines. Let the children express themselves through these lines.
- It is possible that in the beginning some children write the letters incorrectly. In such cases the teacher should help them separately.
- The practice of writing letters by looking at them should not be forced in class.

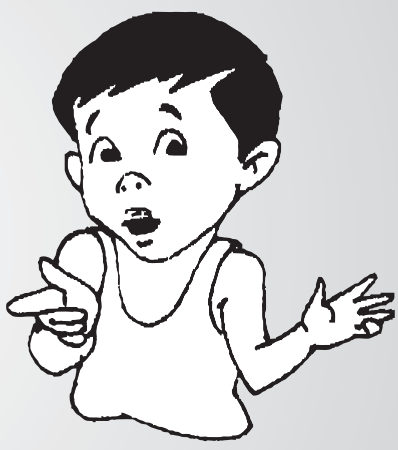

Introduction

Learning is a continuous process which begins from the early year of the children. There are several factors which affects the learning process for example: interest, goal and motivation of a child. It is observed that children have fear of math, instead it is a subject which children can understand easily and apply it to their day-to-day task. Children develop understanding of various concept of math through their immediate surrounding which include their home environment, family, friends any many more. During early year, counting number is a difficult task for children but they have innate understanding to make the estimation of sizes, shapes, distance, thickness, etc. on their own. Children are also capable of understanding different patterns around them. Even though they cannot understand the mathematical concepts such patterns, they still enjoy it. It is important to consider children's experiences while introducing mathematical concept to make the process of learning more enjoyable and easier.

Things to remember in order to teach children

- Normally, children start grasping mathematical concepts by observing the things around them. Concepts like measuring quantities, finding patterns, understanding balance and positions and sharing things equally with their siblings come naturally to them without any special instructions.
- Sometimes, children can be deprived of such experiences and in those cases; it is our job to help them in an effective way.
- Before making the children to read numbers, it is important to make them learn the foundational skills like counting things, their order, and cardinality. This will help the children in understanding the symbols of numbers and the relationship between the numbers and their base tens.
- Encourage the children to present their work after working with their group members to build their confidence and to help them decide what is wrong and what is right.
- In order to make children learn the mathematical concepts easily, it is important to teach these concepts by organizing various activities that will help the children to learn these concepts in an enjoyable way.

First Phase (Warm-up Phase) - Goals and Daily Plans

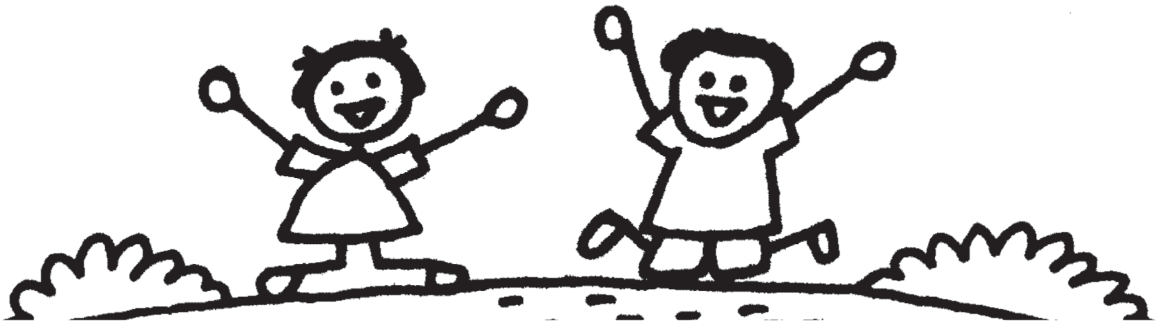

- Understanding mathematical vocabulary like - **less than - more than, far - near, small - big,** etc.
- Introduction to numbers from 1 - 20
- Understanding basic shapes △ ▭ □ ○
- Solving one-digit word problems orally

Activities to be organized every day in the classroom	
**Song-Poem 5 minutes**	Organize a song - poem session in class every day. Start the session and gradually encourage the children to come forward and sing songs - poems on their own.
**Pre-Math 10 minutes**	• Less than - more than, up – down<br>• Introduction to the shapes<br>• Different patterns in shapes<br>• Games with shapes
**Identifying number 15 minutes**	• Reading number chart<br>• Identifying numbers<br>• Bundle and Straw
**Fun Math 10 minutes**	Organize a level-specific activity
**Word-based Games 10 minutes**	Talking, guessing, estimating, etc.
**Physical Games 10 minutes**	Conduct physical games and observation related activities daily.

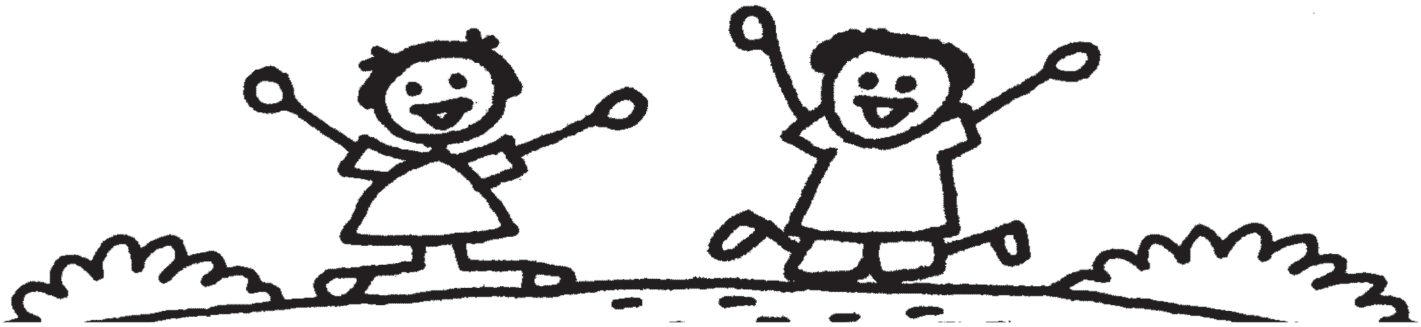

Poems

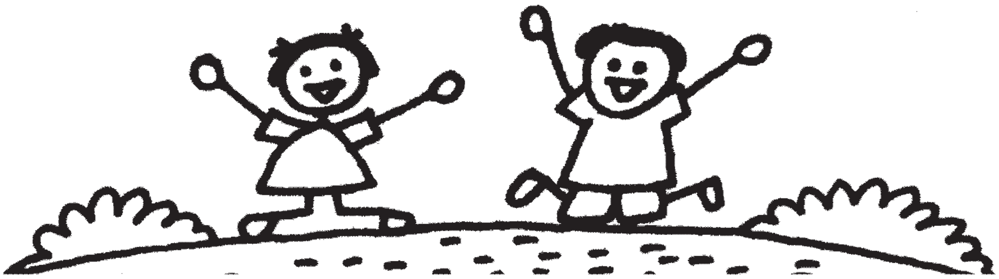

5 little monkeys

Five little monkeys jumping on the bed
One fell off and bumped his head
Mamma called the doctor and the doctor said,
"No more monkeys jumping on the bed".
Jumping on the bed,
Jumping on the bed,
Jumping on the bed
And falling off the bed!

4 little monkeys.....

No little monkeys jumping on the bed
None fell off and bumped his head
Mamma called the doctor and the doctor said,
"Put those monkeys back in the bed".

One Two Buckle my Shoe

One, two,
Buckle my shoe.
Three, four,
Knock at the door.
Five, six,
Pick up sticks.
Seven, eight,
Lay them straight.
Nine, ten,
A big fat hen.

Bus on the wheels

The wheels on the bus go round and round
Round and round, round and round
The wheels on the bus go round and round
All through the town.

The wipers on the bus go "Swish, swish, swish,
Swish, swish, swish, swish, swish, swish"
The wipers on the bus go "Swish, swish, swish"
All through the town.

The door on the bus goes open and shut
Open and shut, open and shut
The door on the bus goes open and shut
All through the town.

The horn on the bus goes "Beep, beep, beep
Beep, beep, beep, beep, beep, beep"
The horn on the bus goes "Beep, beep, beep"
All through the town.

The money on the bus goes "Clink, clink, clink,
Clink, clink, clink, clink, clink, clink"
The money on the bus goes "Clink, clink, clink"
All through the town.

The baby on the bus says, "Wah, wah, wah!
Wah, wah, wah, wah, wah, wah!"
The baby on the bus says, "Wah, wah, wah!"
All through the town.

The people on the bus say, "Shh, shh, shh,
Shh, shh, shh, shh, shh, shh"
The people on the bus say, "Shh, shh, shh"
All through the town.

The wheels on the bus go round and round
Round and round, round and round
The wheels on the bus go round and round
All through the town.

Math

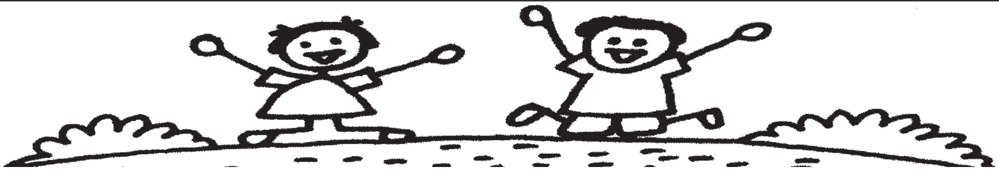

When we look at children aged 5-8 who are developing their pre-math abilities and are moving towards recognizing numbers, we assume how easily do these children combine their understanding of Math with their life and classroom situations. As a teacher, one should think why children in upper primary classes are scared of Math. This might happen because of the lack of balance between understanding of mathematical concepts and their practice.

There are two important things to be considered while teaching Math in grades1 and 2:

- There should be proper balance between Pre-mathematical abilities and the number recognition skills.
- The process of teaching these concepts should be made enjoyable to enhance children' learning.

Pre - Math: Less than/more than, up - down, forwards - backwards, near - far

- Show a picture to the children and ask what is there in the picture. For example - **Where is the boy standing?**, **On what side of the tree is the cow standing?, Who is taller between the two boys?** etc. (Make sure to place your finger on the picture while talking about specific people and objects in it.)
- This activity can also be done with objects present in the classroom. For example - **Where is the roof?, Where is the bulb?, What is there on the table?** etc.

Number Identification : Bundle from straws

- Show a straw to the children and ask them what it is. After some children have responded, tell the children that we will call it a straw from today.
- Show the straws to the children and ask them how many straws are there.
- Ask the children to pick a straw, one by one, count and call out the total number of straws.
- Hold 3-4 straws in your fist and ask the children how many straws are there. After some children have responded, pick one straw at a time and ask them to count the straws like five, six, seven, etc.
- Distribute straws to children. Ask them to count their straws, locate that number on the 'Number Chart' and to write the same number either on the floor or in their notebooks.
- Make a bundle with the help of 10 straws and ask the children that we will always make a bundle when we have 10 straws and it will be called as a 'Bundle'.
- Give the straws to the children and ask them to make bundles. Help the children to understand the concept of the numbers such as '10', '20', '30', ... ,'90' with the help of straws and bundles.
- After some days, help children to understand the concept of 'more than' and 'less than' with the help of the bundles and straws.
- Ask the children to call out the bundle and the number of straws used to make that bundle. Example: 3 bundles = 30 straws (10 straws per bundle)

Word Problem - Addition and Subtraction

1. Call out a word problem for the children as a story.
2. Ask the children what happened in the beginning and what happened next.
3. Ask the children what do they think what happened and what should be done. Also ask the children if things have become more or less, etc.
4. Ask the children what will be the answer. After some children have responded, ask the children how did they solve it.
5. Similarly ask the children to solve more word problems.

The world of shapes

- Show a circle to the children and ask them what it is.

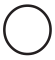

- Ask the children to call out some objects of the same shape.
- Show the circle again to the children and tell them its name.

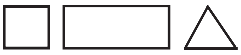

- Similarly discuss a square, a rectangle and a triangle with the children.

Some More activities with Shapes:

- Divide the children into groups and give one set of shapes to each group and ask each group to make something using the shapes.
- Ask each group what they have made using the shapes.
- Organize a discussion about each shape, its side(s), its corner(s) and the things that each group has made.

Activities of Number Chart

1. Call out the numbers from 1-20 on the 'Number Chart'.
2. Call out each number, one at a time, by placing your finger under each word with correct pronunciation and ask the children to look carefully as you read and do not repeat the numbers after you.
3. Ask the children to identify the numbers as you read the numbers on the 'Number Chart' randomly.
4. Read the numbers again and ask the children to repeat the numbers after you on their number chart.
5. Ask the children who will read like you when you have completed reading the numbers (Give an opportunity to 2-3 children to read the numbers).
6. Call out some numbers and ask the children to locate those numbers on their number chart by placing their finger on it, and to write the same number either on the floor or in their notebooks.

Note: Call out the numbers from the 'Number Chart' randomly and ask the children to identify the number and to tell you how many bundles and straws will be required to form that number.

Fun with Math

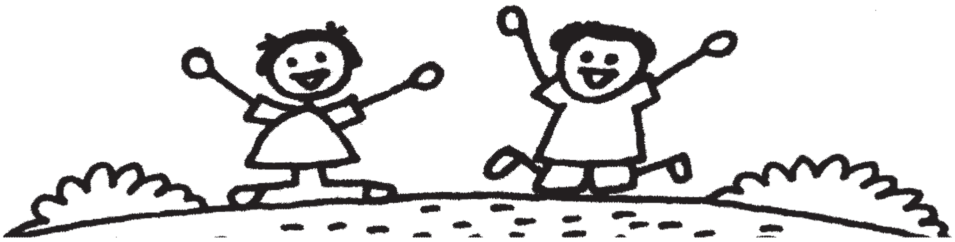

Out of the given activities, conduct any one activity in class every day

Forward- Backwards Counting

Make the children sit in a circle and ask them to start counting. First child says 'one'; second child says 'two' and so on. After one round, ask the children to count backwards. First child says 'ten', second says 'nine' and so on.

Guess and Find the Number

Draw a circle or any shape on the board and make a lot of dots inside it. Ask the children to guess how many dots are there in the shape. After they guess, count the number of dots.

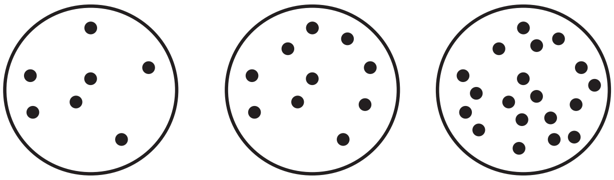

Dot Patterns

Show the flashcards with patterns of dots to the children and ask them how many dots do they see. Start with the basic dots with the children.

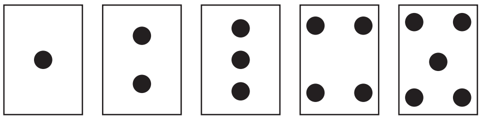

Ask the children to add the basic patterns of dots with the next pattern of dots and to answer the total number of dots.

Shape of Straw

Divide the children into groups. Divide some straws in each group. Ask the children to make different shapes on the floor using the straws. Discuss each shape with the groups.

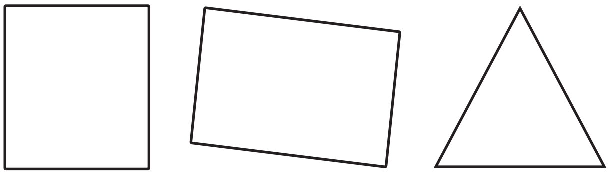

Shapes from shapes

Give children a piece of chalk and ask them to draw any shape. After a child has made a shape, ask her/him to draw another shape inside it. Example: 'Circle', draw a triangle inside the circle.

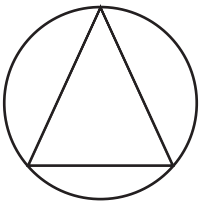

Reading Time

Imaginary and short stories are interesting for the children because it is connected to their lives and environment due to which reading and learning becomes interesting and entertaining. However, it is imperative to have reading practice of poems. The practice of reading written materials of different forms helps in strengthening the ability to read and understand. Hence, various materials of reading like newspaper, magazines, articles, story books, letters etc should be available in the class for children.

What to do in Reading Time:

- Select a 'Reading Corner' somewhere in the class and put various reading materials in it. Introduce the Reading Corner to the children in the beginning itself.
- Encourage the children to read material (silent reading) of their choice for 5-10 minutes daily.
- Meanwhile, teacher should also engage in reading and encouraging the children to deliberate among themselves if they don't know the meaning of a word.
- Sometimes, ask the children to read a particular reading material by making announcements like 'Today, we shall read the newspaper.' Once they are done with reading, ask the children to explain what is being conveyed through the reading material.
- Once in a fortnight, organise a small discussion session of 5-10 minutes where children have to tell what they liked amongst their readings and what they didn't. (Teacher should begin with sharing their opinion about the read material.)

Points to be noted:

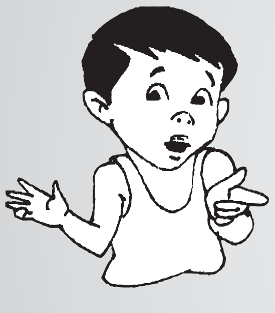

- The books in reading corner should be within the reach of the children.
- Children stay interested if the books in reading corner are changed every month.
- Ask for children's opinion about the books once in a week.
- Reading corner should necessarily have reading materials for different levels of children.

Annexure 1 : Classroom Management

In order to make the process of learning how to read and write more comprehensive, not only is it important to conduct necessary activities but also the way they are conducted. Therefore how should we manage our classroom? We need to focus on this part so that children develop in a better way. In order to meet this goal, all activities in the class should be conducted with large groups, small groups and even with individual children.

With Large Groups:

Story related activities or any other activity can be demonstrated in class so that the children can be familiarized with the activity and when working in small groups, can do it easily. Some examples are **'Story Reading', 'placing your finger on a chart and reading', 'discussion before writing and role-plays'.**

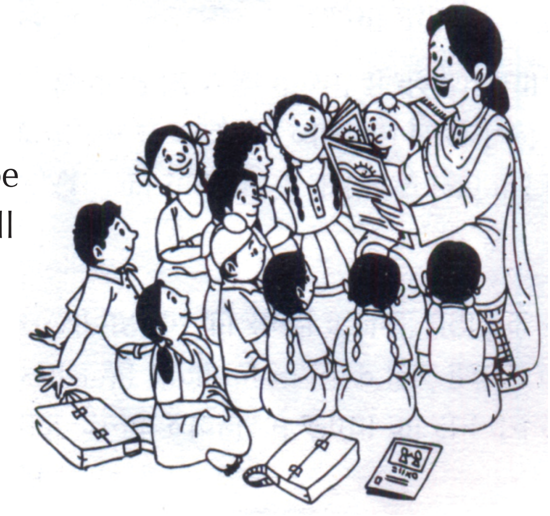

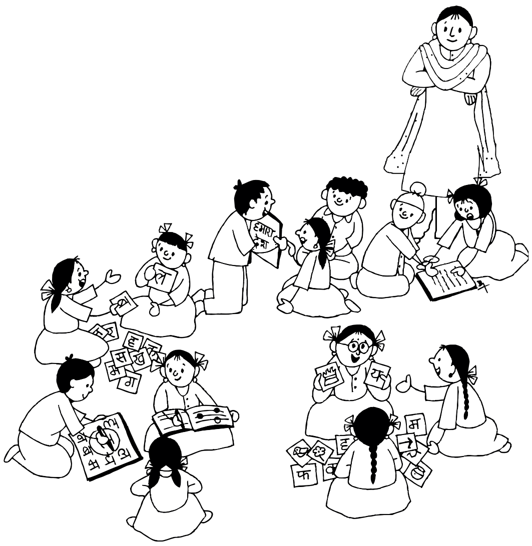

With Small Groups:

In small groups, all the children get to participate in level-specific activities in a given time period. Additionally, the children also get the chance to interact and discuss their ideas with each other. When children are working in small groups, the teacher can sit with them to help them if they face difficulties. Activities like **Level-Specific Games, Question making, Role-play etc** can be conducted in small groups.

With Individuals/Self:

In order to make sure that each child is able to fully understand an activity, it is important that he/she is provided with the opportunity to practice the activity in class at an individual level. Activities include- **Writing, Mathematical games, etc.**

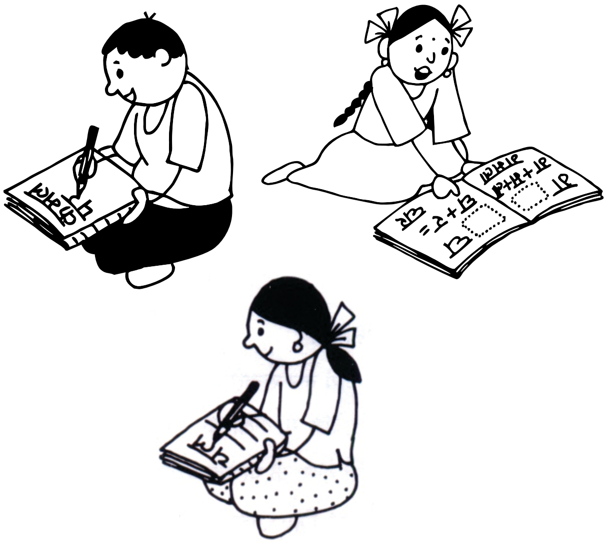

Annexure 2 : Introduction With TLM

What and How?

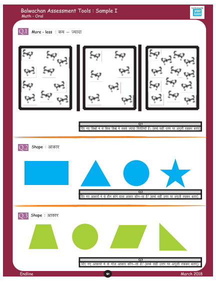

Testing Tool Kit: This testing tool is created in order to assess reading levels of children and to create level-specific activities and groups in class. It contains assessment tools as well as grading formats which will be used to assess language and mathematical abilities of your children. (Every teacher will have one testing tool kit.)

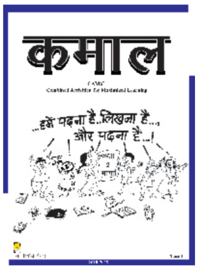

CaMaL Manual: This instruction manual contains the procedure for every activity that is to be conducted in class as well as the target of the activity. Teachers can use it to manage their classes efficiently. (Every teacher will have one manual.)

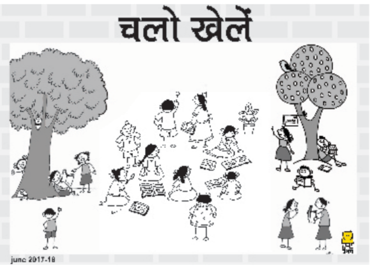

Let's Play: This book contains a variety of games that can be played in the class. Lanugage teaching and learning games have been divided into four sections- Sound games, letter games, word building and word bank games. (Every teacher will have one book.)

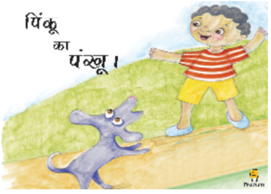

Stories: Different kinds of activites related to the stories will be conducted with children from class 1-2. Each class will be provided a set of stories according to class size.

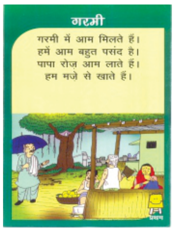

Reading cards: These cards have simple sentences with 5-6 words. These will help in building fluency. Therefore these cards can be given to the children for them to take them home. (Every class will have one set of Reading Cards.)

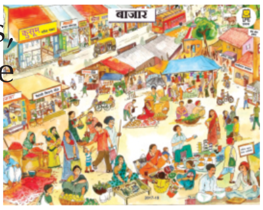

Picture Cards: These cards can be used to have various kinds of discussions, Word Bank activities and writing practice in class. (Every class will have one set of Picture Cards.)

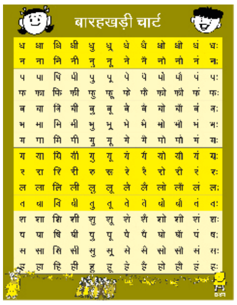

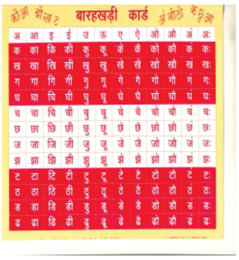

Barahkhadi Chart: Daily reading of the Barahkhadi chart and conducting activities based on it will help the children connect sounds and letters together to better understand the connection between sounds and symbols. Additionally it also helps them to develop their reading speed. (Every class will have one Barahkhadi chard and every child will have one Barahkhadi card.)

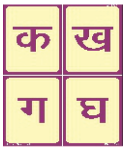

Letter Flash Cards: These cards will help in conducting various activities which will help in understanding the coordination between sounds and symbols.

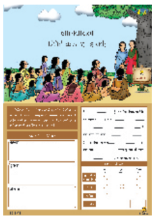

Report Card: Each child in the class will have a report card. The report will have information regarding skill levels in math and language. The report card will be shared with the child's parents/guardians.

Annexure 3 : Become Friends with your children and also try this!

Sometimes for effective organization of teaching and learning activities, one should organise songs, poetry, discussion and listening to children' problems sessions. Given below are some activities which will help in classroom management:

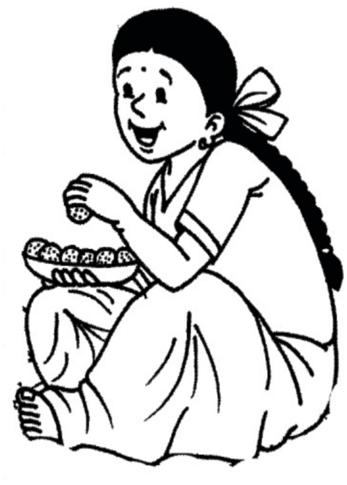

- Call children by their names and use 'आप' to address them.
- Help the children form large and small groups in the beginning and gradually encourage them to form groups on their own.
- Seek permission before entering or leaving the classroom, make sure the class is neat and clean and keep things in their assigned place.
- Before the class begins, pick up waste paper material along with the children.
- Discuss with children the importance of standing in a line and walking in a line. For example: Going to the assembly, coming back to the class after the assembly and going out for lunch during break.
- Discuss the importance of washing hands before and after every meal and after using the toilet .

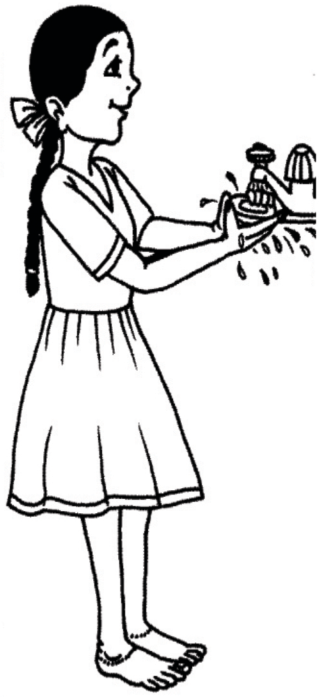

- Encourage the children to take bath, comb their hair everyday and wear clean clothes. Clap for the children who come clean and tidy to school.
- Discuss with the children the importance of listening to others and expressing their thoughts clearly, to wait for their turn and raise their hand before answering a question.
- Discuss the classroom rules with the children.
- If a child violates any of the classroom rules, do not scold her/him but try to discuss the problem and find a solution.
