# Supplementary material for: Support To Rural India’s Public Education System (STRIPES2) and impact on numeracy and literacy scores: A cluster randomized trial in rural villages of Madhya Pradesh, India
Source: PLoS One. 2025 Sep 12;20(9):e0330203. doi: 10.1371/journal.pone.0330203 (PMC12431668; doi:10.1371/journal.pone.0330203)
Supplement: S2 Appendix — (PDF) [file pone.0330203.s002.pdf]

Manmauji Ganit

Pratham

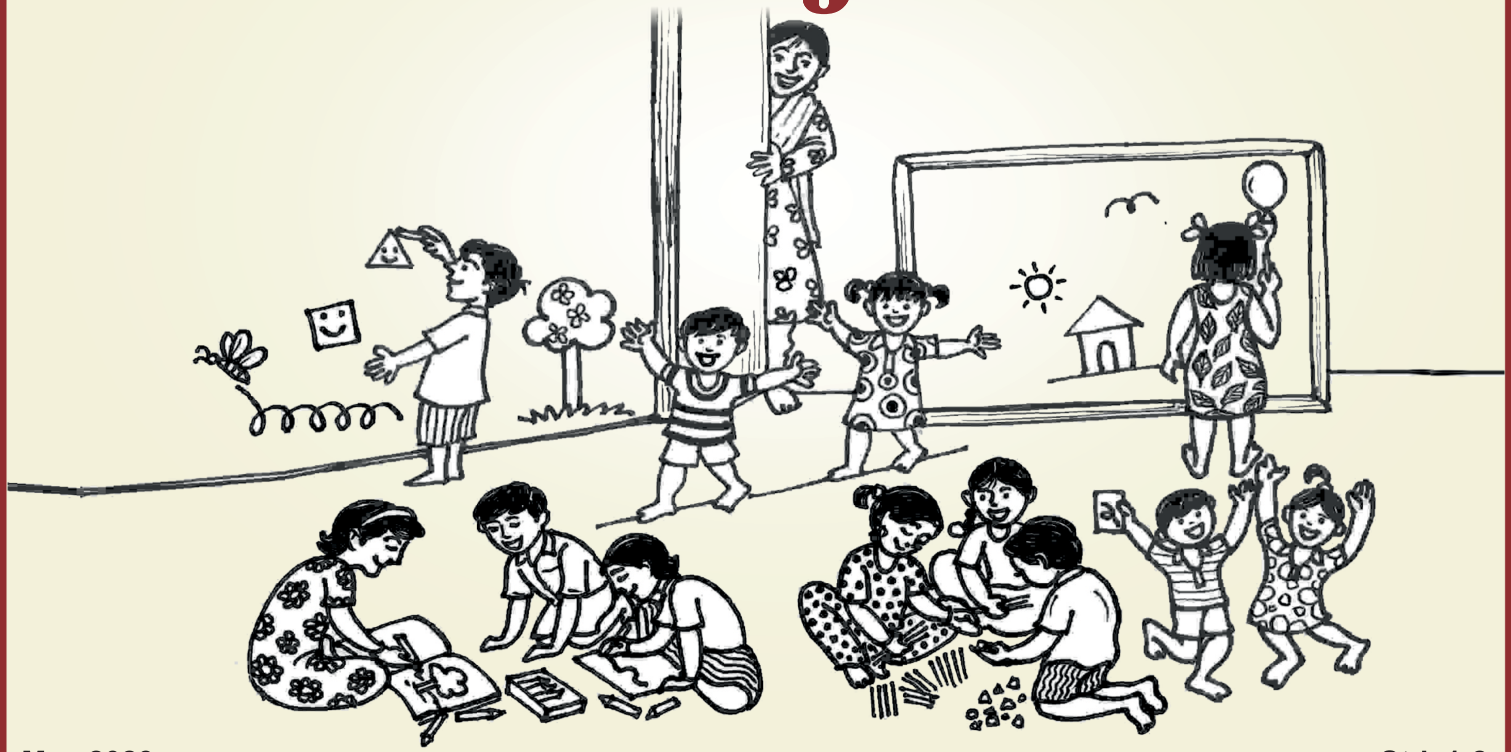

May 2023

Std. 1-2

Forward Pattern

Classroom Arrangement: In a big group

Material: Nothing

Process: Oral

Tell the children that let's play a game. All children listen carefully and tell what will come next?

- Speak the pattern of objects/colors comfortably, such as red-blue, red-blue, red.
- Ask the children, what will come next?
- Say the next pattern for practice, such as a bowl – a glass, a bowl – a glass….
- Similarly, do the activity of oral pattern.
- It is necessary to use picture cards along with solid objects to understand and make patterns.
- Where possible, use English vocabulary yourself and the children, such as - the pattern of 'circle' is visible in it.

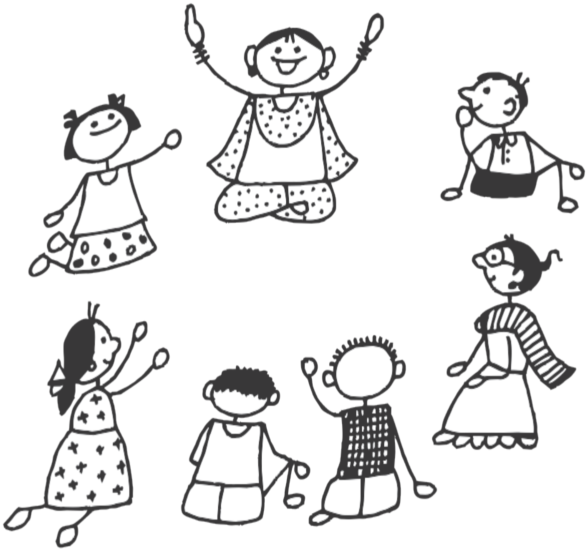

Forward the Pattern (With Objects)

Classroom Arrangement: In a big group & small group

Material: Some solid objects.

Process: With solid objects.

- Take some similar types of items, like utensils, bowls & spoon etc (quantity of each item should be 3-4 pieces)
- Now arrange these items in any patterns, like- 1 bowl- 1 spoon,1 bowl-1 spoon,1 bowl… (Apply the pattern while speaking to the children and then stop).
- Now ask any child to complete the pattern.
- Encourage each child to make a pattern with whatever they have.
- Make the initial patterns simple & use arrangement by using only two items.

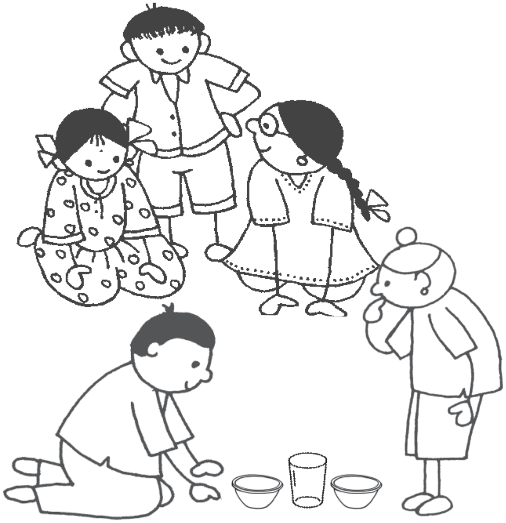

Where the pattern appears

Classroom Arrangement: Small group.

Material: Nothing

Process: With solid objects

- Ask them to sit in a group.
- Give a piece of cloth to each group.
- Ask the children to find the pattern of the piece of cloth.
- Give the group time to find the pattern in the piece of cloth.
- Each group will talk about the pattern one after another.
- Ask children to bring a small piece of old cloth from their home one day before.
- Where possible, use English vocabulary yourself and the children, such as - the pattern of 'circle' is ‘same’ in it.

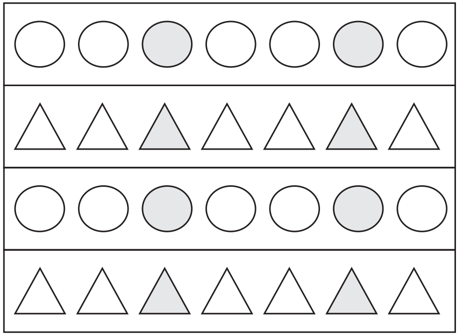

Pattern (with numbers)

Classroom Arrangement: In a big group

Material: Copy, pencil

Process: Written

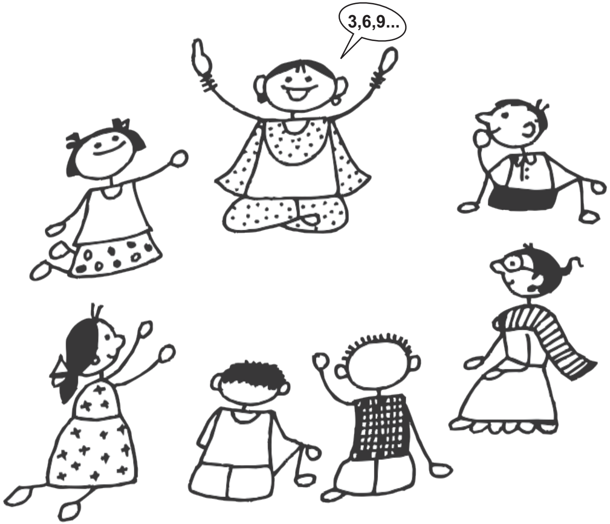

- Tell children - today we will play a new game.
- Then say a pattern of numbers to children, such as 3, 6, 9, 12....
- Talk to understand the pattern, 6 after 3 and what will come after 6... This means 3 is being added every time.
- Now ask children to continue it.
- Give other similar patterns to children to do.
- It is essential to talk about the number pattern again and again.
- While doing the number pattern, in the beginning make the children do the number pattern according to their level.

Classification

Classroom Arrangement: In a big group

Material: Copy, pencil

Process: With solid object

- Sit in a small group.
- Give each group some mixed things, such as gram, chickpeas, gram and small seeds, etc.
- Now ask children to sort the given items and separate them.
- Talk to children, which item needs to be kept separately and why?
- Do this activity with a picture flash card along with a solid object.
- Ask them to classify the shapes on the basis of smaller, bigger and equal sides.
- Use English vocabulary/sentences wherever possible, like side, square etc.

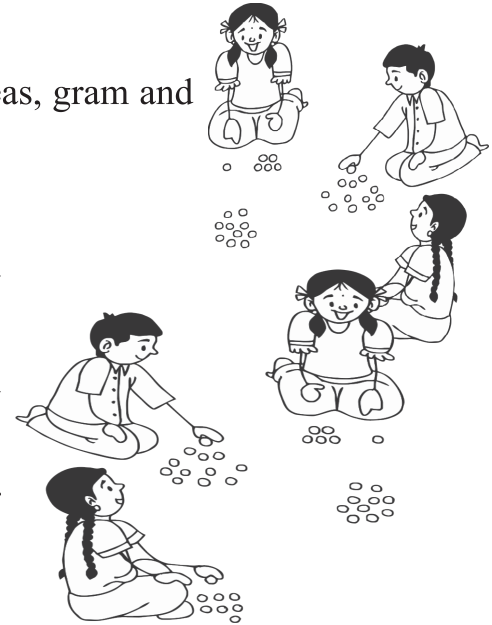

Come, Let's separate

Classroom Arrangement: Small Group

Material: Different types of Objects

Process: With Solid Objects

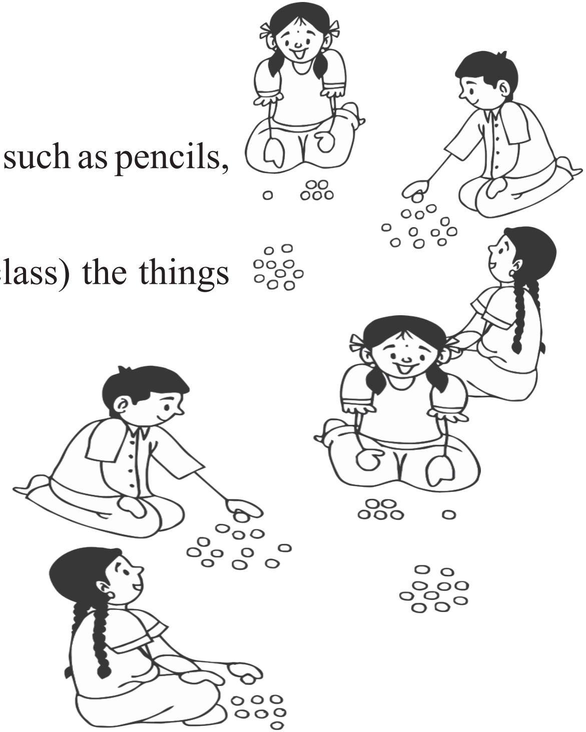

- Ask the children to sit in small groups.
- Ask each group to collect different types of objects, such as pencils, erasers, leaves, pebbles, etc.
- Ask each group to separate (by colour, shape or class) the things they collect in their group.
- At the end, do the same activity with picture cards like picture/flash cards of birds, vehicles, animals and food items.
- Similarly, play this game with other objects/things, like mixing large grains (Rajma and Chana) and ask them to separate them.
-

Come, Let's separate

Classroom Arrangement: Small Group

Material: Different types of Objects

Process: With Solid Objects

- Ask the children to sit in small groups.
- Ask each group to collect different types of objects, such as pencils, erasers, leaves, pebbles, etc.
- Ask each group to separate (by colour, shape or class) the things they collect in their group.
- At the end, do the same activity with picture cards like picture/flash cards of birds, vehicles, animals and food items.
- Similarly, play this game with other objects/things, like mixing large grains (Rajma and Chana) and ask them to separate them.

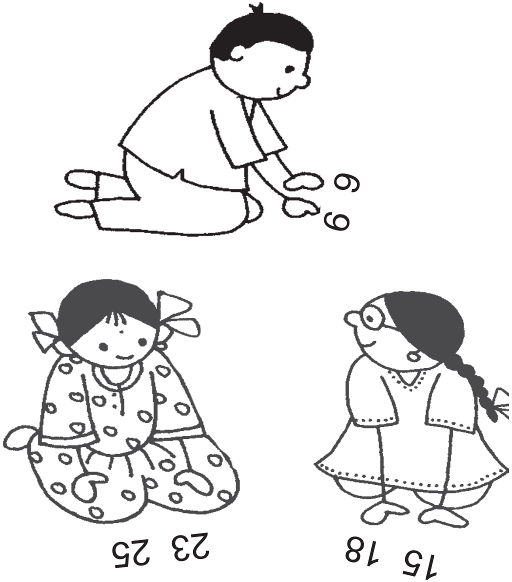

More or Less (With numbers)

Classroom Arrangement: Small and big group- Individual

Material: Nothing

Process: Oral and Written

- Ask the children, let's play a new game, I will say two numbers, you have to find them and write them.
- Then say any two numbers like 6-9/15-18/23-25 etc.
- Ask the children which of these numbers is less or more.
- Similarly change the number and ask
- Say the numbers according to the level of the children.
- Children write numbers by finding them from the number cards.

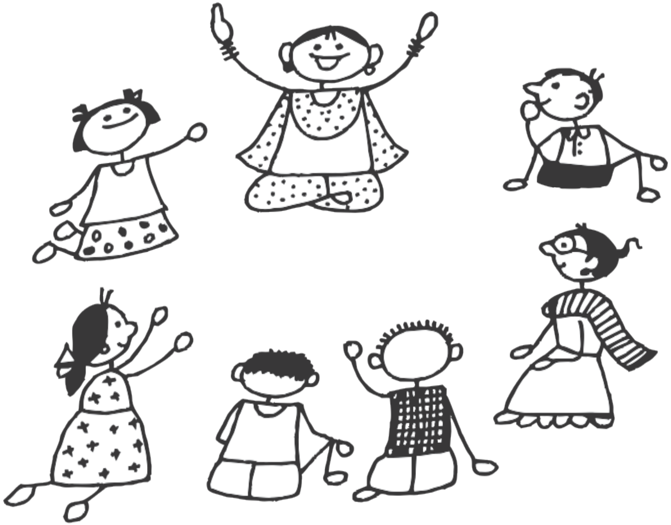

Difference/Similarities in shapes

Classroom Arrangement: Small group

Material: Various shapes (Set of shapes for each group)

Process: Oral and Written

- Show any two shapes and ask the children what is the difference between these two shapes?
- Discuss the differences and similarities between the two shapes on the basis of angles, sides, colours etc.
- Now give two shapes to each group. Discuss and write the similarities or differences between the given shapes in each group.
- Now go to each group, ask for differences or similarities in shapes.
- Make sure to talk about the side, name, corner etc. in difference and similarity.

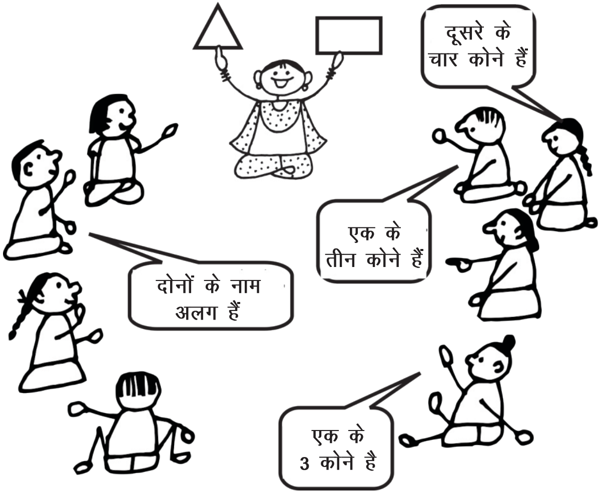

What did I make

Classroom Arrangement: Big group

Material: Nothing

Process: Oral and Written

- Ask them to sit in a large circle.
- Ask the children to draw any shape they want in the air and the rest of the children identify it.
- Ask all the children what shape they make?
- Similarly, ask the children to draw and identify shapes in the air.
- This activity can also be done in pairs, with one child drawing the shape on the back of the other.
- If possible, use as much English vocabulary for yourself and the child as well, such as This will be a 'square'.

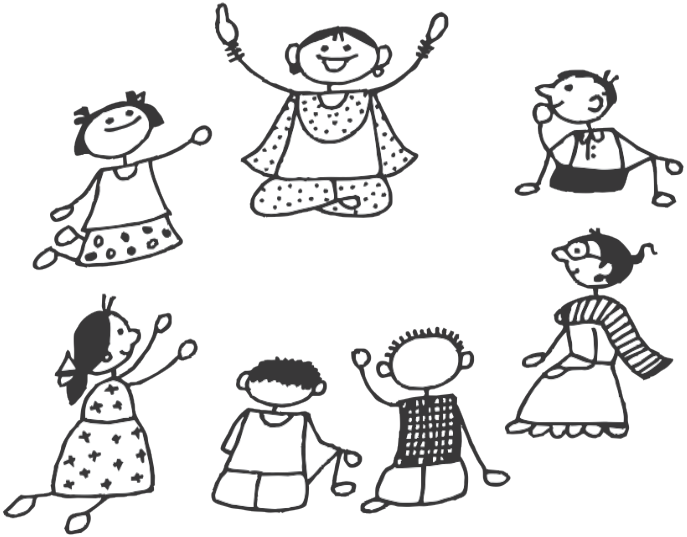

What I made

Classroom Arrangement: In a big group

Material: Nothing

Process: Oral and Written

- Ask to sit in large circles.
- Tell children to draw any shape they like in the air and let the rest of the children recognize it.
- Ask all children what type of shape they made?
- Similarly, ask children to draw and identify shapes in the air.
- This activity can also be done in pairs, in which one child creates shapes on the back of another child.
- Where possible, yourself and children should use as much English vocabulary as possible, such as it would be a 'square'.

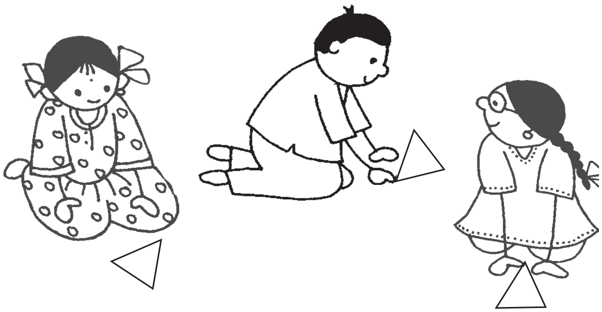

Size from objects

Classroom Arrangement: In big group/individual

Material: Objects from around them

Process: With Solid objects

- Everyone, sit in a circle.
- Tell children that I will say a shape and you will make that from things around you, such as a house/shape, rectangle/square/hexagon etc.
- Continue the game like this.
- Where possible, yourself and children must use English vocabulary/sentences, such as- Make a 'square'.

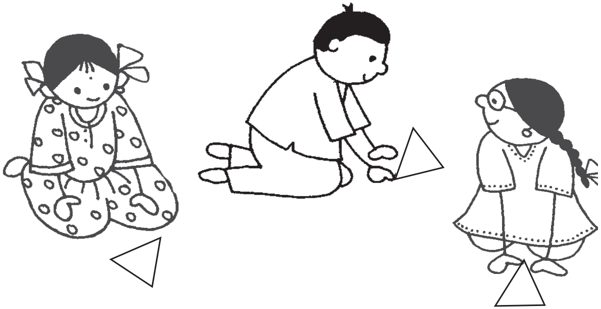

Distribute equally

Classroom Arrangement: Big group/individual

Material: Copy, pencil/chalk

Process: Written

- Everyone sits in a circle.
- Tell the children, the shape which I will say, you will draw it on your copy/ground.
- Then ask children to make the shape into two parts/pieces from its middle.
- Continue the game with other shapes.
- Wherever possible, yourself and children must use English vocabulary/sentences, such as- divide 'square' into 'two' parts.

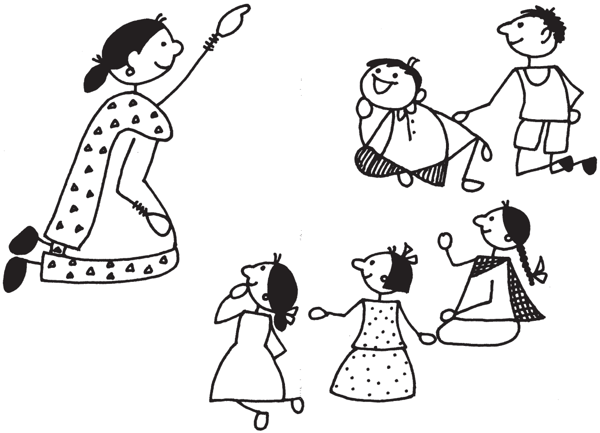

I will say and you will Count

Classroom Arrangement: Individual

Material: Stick/ pieces of chalk/pebble

Process: With solid Objects

- Tell children that we will play a game.
- Give sticks/pebbles/pieces of chalk to all the children.
- I will say a number, you count the same number of sticks/pebbles/chalk pieces.
- Say a number, ex-6, children will count 6 sticks and tell.
- After a few days children can also be asked to write numbers/digits.
- Use English vocabulary/sentences yourself and the child where possible, such as- count to 'five'.

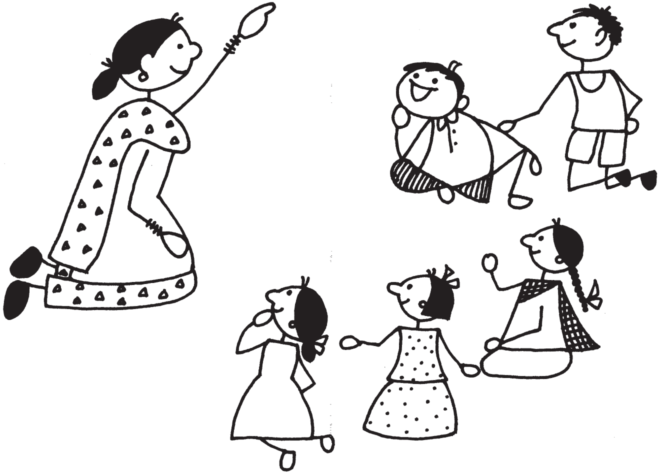

Brother will say how many

Classroom Arrangement: In a big group

Material: Nothing

Process: Written

- All the children stand in a circle and you stand in the middle.Tell children we will clap and sing a song. I will say how many brothers, how many and while roaming in the circle - you will say as many as you want.
- When you say a number, such as 3, all the children will form groups of 3. Children whose group is more or less than 3 should sit down.
- Continue playing like this.
- Keep in mind that while forming small groups, the children should not be drawn with each other.
- Where possible, children and themselves should use English vocabulary/sentences, such as- If I say 'three', then you make a 'group' of 'three'.

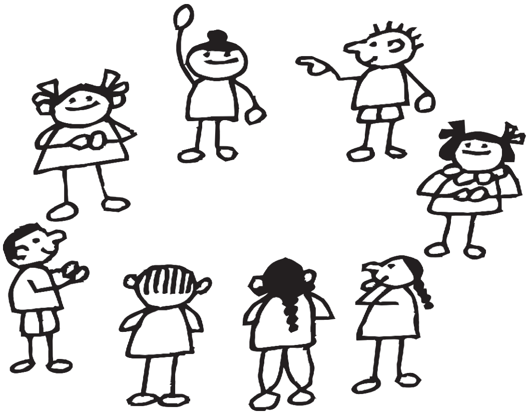

Descending order in one minute

Classroom Arrangement: In a small group

Material: Number Cards

Process: Oral

- Sit in small groups.
- Now make some cards and write a number on them.
- Children from any group will come and pick up a card. After reading the number written on the card, stand in the line in such a way that the numbers are in decreasing order.
- Give each group a chance to play the game in turn in the same way.
- Use English vocabulary/sentences by yourself and the child where possible, such as- 'five' is 'bigger' 'number' than 'two'.
-

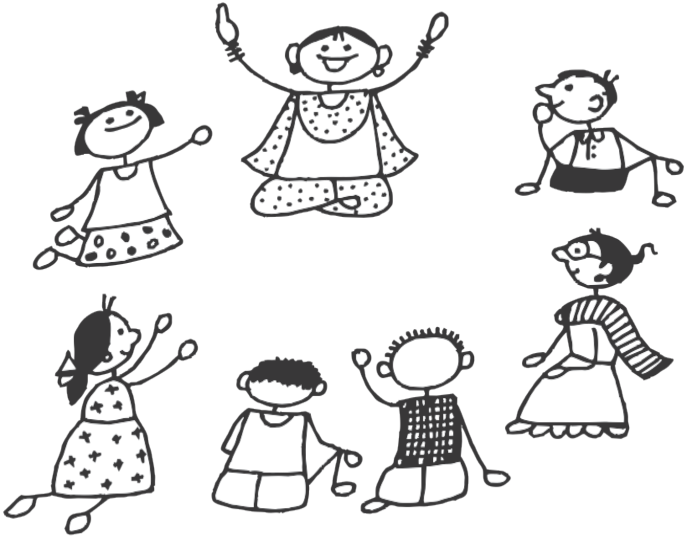

How many bundles and how many sticks

Classroom Arrangement: Individual

Material: Nothing

Process: Oral

- Give sticks to children.
- Children build a house of bundles and sticks.
- Count bundles and sticks and find from the number card.
- Ask children to say the number in bundles and sticks, example- 25, children will say two bundles and five sticks.
- Make sure the children change sticks in between.
- Be sure to keep sticks in your hand as well.

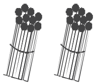

दहाई	इकाई
बंडल	तीली
2	5

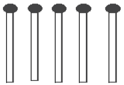

Cross the Line

Classroom Arrangement: In big/small groups

Material: Nothing

Process: Oral

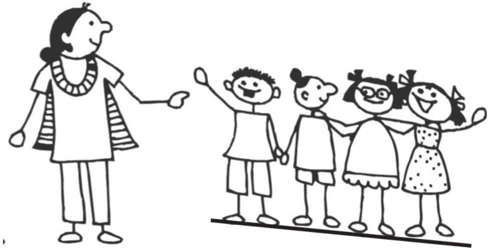

- Draw a straight line on the floor. Make the children stand on one side of the line facing in the same direction.
- Now tell the children that you are going to say something, if they agree to it, they are going to cross the line, if they do not agree with it, then they must stand at the line itself.
- Now, you have to say 'cross this line if you feel that adding one and one makes it three?'
- It's on the children now to decide whether they have to cross the line or not.The child who commits a mistake shall sit (who crosses the line when the children were not supposed to cross the line or does not cross the line when they were meant to cross the line).
- Play this game unless there is one child left at the end.

- You can play this game with different shapes/patterns/classifications too.
- Wherever possible try to use English vocabulary/sentences yourself and ask students to use them too.
