# Supplementary material for: Support To Rural India’s Public Education System (STRIPES2) and impact on numeracy and literacy scores: A cluster randomized trial in rural villages of Madhya Pradesh, India
Source: PLoS One. 2025 Sep 12;20(9):e0330203. doi: 10.1371/journal.pone.0330203 (PMC12431668; doi:10.1371/journal.pone.0330203)
Supplement: S3 Appendix — (PDF) [file pone.0330203.s003.pdf]

Manual for Master Trainers

Grades 1&2

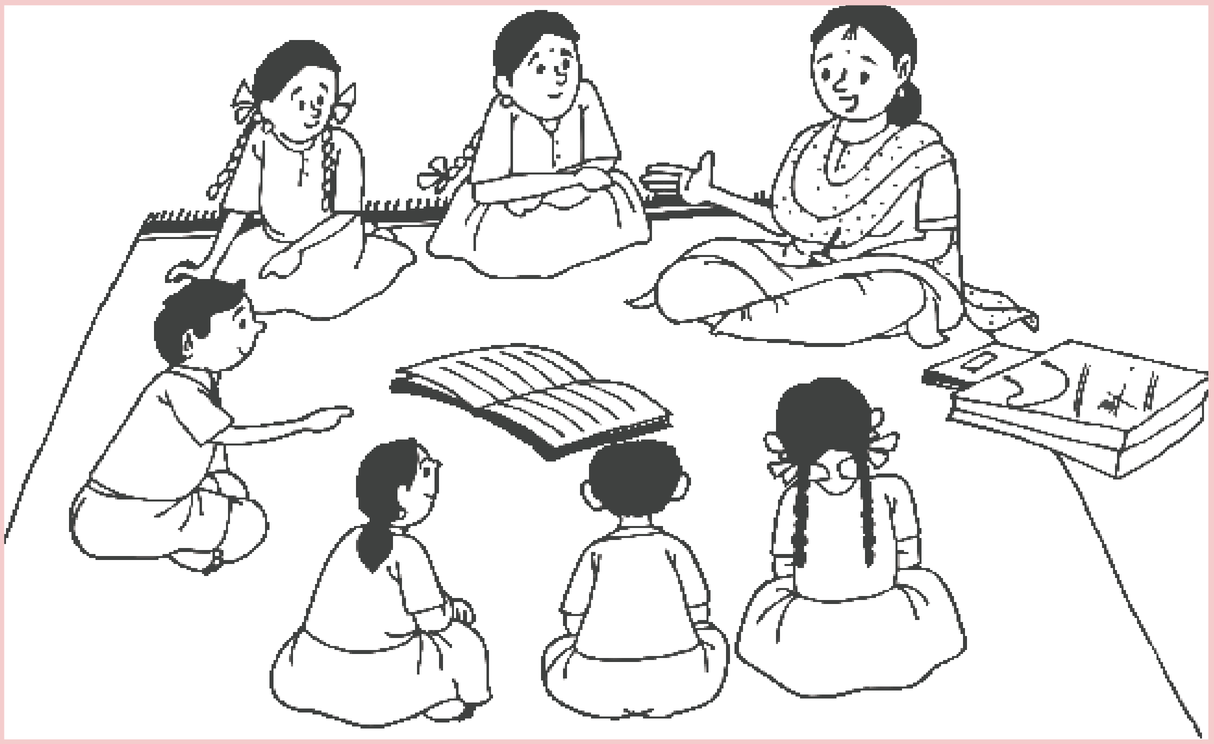

NIPUN BHARAT PLEDGE

Let us join hands to ensure a conducive learning environment enabling all children to achieve foundational skills.

We pledge to make the school a place of joyful and experiential learning where children can use their language freely, ask questions freely, play freely, and where every child is respected.

Let us make the school as well as the home, a place for developing lifelong skills for reading with comprehension, writing with purpose and understanding numeracy, in every child that they can apply in their everyday life situations.

Let us strive to make education meaningful and joyful for each child of our country and make every child NIPUN.

PREFACE

The Early Years are critical years for the development of a child. It is a time when brain development is at its peak, and children respond rapidly to stimulation and input. The development of foundational skills in this period have been closely associated with improved learning outcomes, including aspects such as better school readiness, and enhanced retention in primary grades. Poverty, malnutrition and the lack of adequate resources can severely impede development at this stage, resulting in persistent challenges later in life.

Across India, there is renewed focus on early years as a pivotal stage for child development. Policy documents recommend that focused intervention in this period can go a long way in equipping children with the necessary skills required to be able to cope with grade-level curriculum as they progress to higher grades.

60 Days course for grade 1-2 teacher of project SALT (Supporting Andhra's Learning Transformation) is aimed at strengthening the foundations of overall development of children, in terms of physical, language, cognitive and socio-emotional skills, along with creative and aesthetic appreciation before the children are expected to deal with an expanded and extended curriculum in grade 3.

In order to achieve the mentioned in the children of age 3-8, the teacher's role becomes very important. To do so, teachers have to make effective plans for the classroom and use the best strategies to deliver them to their foundational stage learner. Apart from pedagogical skills, a teacher must have the skill of mobilizing the community and parents to contribute to and take ownership of their child's learning. Therefore, building the capacity of the teacher and providing extensive support becomes an integral part of any program/project.

The main goal of Mission FLN initiated by NIPUN Bharat is to enable all children across the country to acquire the ability to read, write and do arithmetic by the end of Class 3 by promoting foundational literacy and numeracy by the year 2026-27.

This trainer module is developed in alignment with the NIPUN document and an attempt has been made to keep all the above-mentioned points in mind while preparing the training manual. We hope that you will take care of all the points given in the module while conducting the 6 days training of the teachers.

GLOSSARY

Alphabet Knowledge: Alphabet knowledge is the recognition of letters as distinct symbols that have specific names and specific sounds associated with them.

Anecdotal Record: An anecdotal record is a detailed descriptive narrative recorded after a specific behavior or interaction occurs. Anecdotal records inform teachers as they plan learning experiences, provide information to families, and give insights into identifying possible developmental delays.

Assessment for learning: This is an approach to teaching and learning that creates feedback which is then used to improve students' performance.

Balanced Approach: Where teachers follow what is appropriate for their classroom and where every child learns in a joyful and stress-free manner, by taking the best of multiple approaches.

Competency: Competencies are statements that specify what children will know, be able to do, or be able to demonstrate when they have completed or participated in a course or program.

Culture: The Oxford Dictionary defines Culture as - the arts and other manifestations of human intellectual achievement regarded collectively.

Classification: involves putting together things that have some characteristics in common.

Data Handling: Data refers to information in a raw form that is collected from various sources. Data handling includes collecting, representing, analyzing and interpreting data.

Early literacy: Early Literacy is what children know about reading and writing before they read or write.

Ethnicity: Oxford reference states that - it is a term for the ethnic group to which people belong. Usually, it refers to group identity based on culture, religion, traditions, and customs.

First Generation Learners: It refers to the students who are the first in their entire generation to go to school and receive an education or whose parents have attended the formal education system only up till primary level of schooling.

Formative assessment: Formative assessment refers to a wide variety of methods that teachers use in the classroom to conduct in-process evaluations of student comprehension, learning needs, and academic progress during a lesson, unit, or course.

Foundational Numeracy: The ability to read and write and perform basic operations with numbers.

NIPUN BHARAT: National Initiative for Proficiency in Reading with Understanding and Numeracy

Fine motor skills: The coordination of small muscles in movements usually involves coordination of hands and fingers with eyes.

Gross motor skills: Gross motor skills are the abilities required to control the large muscles of the body for walking, running, sitting, crawling, and other activities.

Holistic development: Development of intellectual, mental, physical, emotional, and social abilities.

Learning outcomes: Learning outcomes are statements that describe the knowledge, skills, and attitudes that students should acquire by the end of a particular assignment, class, course, or program, and help students understand why that knowledge and those skills will be useful to them.

Life skills: Life skills are defined as a set of abilities, attitudes, and socio-emotional competencies that enable individuals to learn, make informed decisions, and exercise rights to lead a healthy and productive life and subsequently become agents of change.

Literacy: Literacy is the ability to identify, understand, interpret, create, communicate and compute, using printed and written materials associated with varying contexts. Literacy involves a continuum of learning in enabling individuals to achieve their goals, to develop their knowledge and potential, and to participate fully in their community and wider society (UNESCO, 2004; 2017).

Measurement: Involves dealing with quantities involving the use of numbers.

Multilingual Class: This is a class where learners have a variety of first languages.

Numeracy: Numeracy refers to the ability to use mathematical understanding and skills to solve problems and develop a critical viewpoint with appropriate reasoning.

Phonics instruction: This focuses on the relationship of the sounds in spoken words and their associated letters and groups of letters as they appear in print.

Phonological awareness: It is the ability to recognize and work with sounds in spoken language.

Picture reading/Talk: Children can be shown sceneries of a particular event, place, story like a fair/mela, zoo, circus, etc. Children can then be engaged in conversations involving observations (What is happening in the picture?), reasoning (Why do you think so?), prediction (where do you think the girl is going?). Children can also put the events shown in the picture in a sequence and narrate them.

Problem Solving: Problem-solving is the act of defining a problem; determining the cause of the problem; identifying, prioritizing, selecting an appropriate solution from amongst alternatives; and implementing the solution.

Read-aloud: Read-aloud is a practice where teachers, parents, and caregivers pick up an engaging story from a book and read it out. Variations in pitch, tone, pace, volume, pauses, eye contact, questions, and comments make for a fluent and enjoyable delivery. Reading aloud engages the creativity and imagination of children along with increasing their attention span and ability to focus.

Pre-number concepts: Before children start counting objects or develop an understanding of numbers, they need to be able to classify, order, and set up one-to-one correspondences to some extent. Since these skills are preliminary to the understanding of numbers, they are called Pre-number concepts.

Patterns: A pattern is an arrangement, order, sequence, or repetition.

Phonics approach: Children are taught by introducing letters first and then gradually building a correlation with their sound.

Rubric: A rubric is an assessment tool that clearly indicates achievement criteria across all the components of any kind of student work, from oral to written or to visual. It can be used for marking assignments, class participation, personal-social qualities or giving overall grades.

Shared reading: In shared reading, the teacher holds one big book up and the whole class reads from the same book. The book has large font, illustrations, and simple text so that all children can participate in reading. The teacher reads the text while children join when they recognize a word from illustrations, sight word, or memory. Children start developing the concept of print.

Seriation: involves ordering a set of objects according to some rule.

Shapes and Spatial Understanding: Spatial understanding is the area of mathematics that involves shape, size, space, position, direction, and movement.

Sensory and Perceptual Development: Development of the five senses through visual, auditory, and kinesthetic experiences.

Spiral learning: Spiral learning means learning a concept gradually and repeatedly, reinforcing concepts over time, rather than trying to master a subject all at once.

Vocabulary: Developing knowledge of a wide range of words and word meanings.

Whole language approach: Children should be immersed in print rich, literate environments, use authentic children's literature and exposed to lots of opportunities to read and write. Teachers should model how they themselves read and write. Children will be given opportunities to try to express their thinking by experimenting with drawings, scribbling, and invented spellings.

INDEX

S. No.	Topic	Page No.
1	Training: Session Plan for Grade 1-2 Teachers	9
2	Day 1: Understanding the age of 3-9 years	32
3	Day 2: Phase-wise transition & teaching Math in Grades 1-2	59
4	Day 3: Teaching Language in Grades 1 & 2	78
5	Day 3: Other aspects of learning environment	105
6	English as a Second Language	108
7	Community and Parental Involvement	112
8	Assessment in Early Grades	117

TRAINING SESSION PLAN

Training: Session Plan For Grade 1 and 2 Teachers					
Session Time	Session & Lead by	Objective	Duration	Process: Step by Step process for Conducting Session	Material Required
**DAY 1**					
10:00 am - 11:30 am	Inauguration & Introduction	Share the workshop's agenda and objectives with the participants	30 minutes	**Inauguration**<br>Give a background of the program, present a quick framework for how the workshop will be organized, and share the workshop's objectives.	PPT slides 3-4
		Introduce the participants and the facilitators with each other		**Introduction**<br>-Facilitator provides the attendance sheet and asks participants to sign it.<br>-Facilitator welcomes everyone to the training. The facilitator introduces themselves and the co-facilitator.<br>-Facilitator gives instructions on how the introduction activity will be conducted:<br>-Co-facilitator will play music for 7-8 seconds while participants will move around the room randomly. When the music stops, participants will form groups of 5 with people standing near them and introduce themselves by<br>a. Telling their name<br>b. Telling their school's name<br>c. Telling their district's name	

				Repeat this process 2-3 times	
	Setting ground rules for the 6-day training	Identify and establish some rules to ensure that the workshop is productive and its goals are met.	20 minutes	-Co-facilitator to put 1 chart on the wall with the heading 'ground rules' and then give sticky notes to every participant<br>-Ask participants to write rules on sticky notes and paste them on the chart<br>-Co-facilitator must reiterate these rules, discuss any rules that are missing and set a few expectations for the workshop.	- PPT slide 5<br>- A chart labeled 'Ground Rules'<br>- Sticky notes
	Quiz: Baseline	Understand participants' knowledge of the topics to be covered in the workshop before it begins to ascertain what do they know	20 minutes	Facilitator will give the following instructions:<br>-We will open the link shared on the WhatsApp group.<br>-Everyone will attempt the quiz individually without discussing it with anyone.<br>-Co-facilitator will share the link on the WhatsApp group as well as display the slide with the link.<br>-Ask participants to complete the quiz in the given time.	UNICEF Module 1: Video 11<br><br>PPT slides 15-16
	Overview of the certificate course	Discuss the course broadly and share the objectives of SALT	20 minutes	- Facilitator briefs participants about project 'SALT'.<br>- Facilitator delivers an overview of the course including a focus on participant roles and responsibilities with respect to the 60-day certificate course.	PPT slides 7-11
	**Tea break +Energiser**				

11:30 am - 2:00 pm	Brain Development in ages 3-8	Understand brain development and the impact of a positive and stimulating environment on the development of the brain	40 minutes	- Facilitator asks participants about the characteristics of brain development from ages 3 through 8 years (co-facilitator writes appropriate points on the board).<br>- Facilitator plays a video and asks participants to note down important points.	UNICEF Module 1: Video 6, video 8 and video 9<br><br>PPT slides 12-14
	Early Childhood Care and Education (ECCE)	Understand the landscape of ECCE and its importance as a foundational stage	20 minutes	-Facilitator writes 'ECCE' on the board and ask participants to tell its meaning.<br>-Co-facilitator writes the full form of ECCE (early childhood care & education) and facilitator concludes by sharing the definition written on the slide.<br>-Facilitator asks the meaning of care and education for children between the ages of 3 and 8 years<br>- Facilitator to briefly talk about connection between brain development, ECCE and policy, mentioning it will get further covered in 1 pm session.	urse Module<br><br>PT slides 15-1

	Holistic Developme nt: Key Developme ntal Areas & Developme ntally Appropriat e Activities and its connection with the 3 to 8 years continuum	-understand what is meant by holistic development and developmentall y appropriate activities	40 minutes	-Facilitator shows the video<br>-Facilitator asks participants 'What is holistic development and what are some examples of activities that facilitate holistic development? (Co-facilitator will write all the developmental domains on the board.)<br>-After that facilitator discusses the meaning of each domain and a sample activity for each.<br>-Facilitator organizes the participants into 8 groups and provide a chart to each group.<br>-Facilitator gives a specific development domain to each group and ask them to write the list of activities related to that domain (10 minutes). For e.g., Group 1 will get physical domain and write activities for it.<br>-Each group pastes their charts on the wall (5 minutes)<br>-Facilitator concludes the session by summarising the each developmentally-appropriate activity and its relation with the 3 to 8 year age group (10 minutes)	F Module 2: v<br><br>PT slides 17-1

	Transition in Early Years & Stage wise progress recommended by policies:	Understand the transition in ages 3-8 in light of new national education policies	50 minutes	-Facilitator explains the importance of transition period from ages 3 to 8 and its connection with policy with the help of the PPT<br>-Facilitator explains stage-wise progression using the PPT (using example of any one domain)<br>-Facilitator gives participants the opportunity to ask and answer questions<br>-Facilitator will give the reading assignment to the participants from NIPUN; the co-facilitator will call out a few names and those participants will share the key takeaways from the reading.<br>-Facilitator discusses the challenges faced wehn Grade 1 children lack pre-school exposure; the co-facilitator will write the relevant points on the board.<br>-Conclude by mentioning that the best way to deal with the challenges is by 3 months of school readiness.	-PPT slides 19-23
2:00 pm - 3:00 pm	**LUNCH**				

3:00 pm - 4:00 pm	How do children learn?	Understand how children learn	20 minutes	-Facilitator to ask participants- If the brain developed in a rapid and tremendous way then how does a child/brain learn?<br>-Facilitators ask the participants to watch a UNICEF video Module 2: Video 8 'How Children Learn?' and not down the key point while watching it. (2-3 minutes)<br>-Brief discussion on each of the principles of how children learn in depth. (20 mins)<br>-Conclude by showing the slide "7 key principle of learning" (Connect with NCF & Nipun)	PPT slide 24 UNICEF Video : Module 1: Video 8(Intro)
	Working with grades 1-2: phase-wise transition in one academic year and its objective:	Understand the objectives of working with early grades and the transition from one phase to another	20 minutes	-Facilitator will explain the phase-wise transition in grade 1 and overall objective of 1-year long engagement with children.<br>-Once the facilitator explain the year long plan then the questions from participants can be asked and address by facilitated	PPT slide 25
	Readiness Phase and Vidya Pravesh	Understand the objectives of the readiness phase and the vidya pravesh guidelines	20 minutes	-Facilitator and participants brainstorm the objectives of the school readiness phase and how to implement it.<br>-Facilitator provides the school readiness manual in small groups and ask them to read specific pages.	-PPT slides 26-28<br>-Phase 1 manual : page on framework of school readiness classroom process

4:00 pm - 5:30 pm	School Readiness: Week-wise planner and classroom transaction	Understand how to conduct a school readiness class with children	30 minutes	-Facilitator will explain the week wise plan and classroom transaction to group<br>-Later on facilitator demonstrates a complete day's lesson plan for week 1 of school readiness	-Material provided in the trainer kit<br>-PPT slides 29
				**Tea break**	
	Hands-on practice for classroom process of school readiness :	Understand how to provide hands-on practice to the participants for school readiness activities	60 minutes	-Facilitator provides the school readiness manual in small groups and ask them to read specific pages (week wise allocation)<br>-Facilitator asks participants to practice the activities in small groups according to the assigned week (20 minutes for small group practice and preparation).<br>-Four selected groups conduct the demo class (7 minutes demonstration and 2-3 minutes for feedback for each group)	- Slide on group wise distribution of week wise planner for demo and small group practice<br>-Play kit and classroom teaching material<br><br>PPT slides 30, 31 and 32
5:30 pm - 6:00 pm	**Concluding the school readiness phase**				
	**Reflection**				
**END OF DAY 1**					
**DAY 2**					
**Before starting the session on day 2 , the facilitator & co-facilitator will create four corners in the hall. At the time of entry, the facilitator treats the group as children and asks them to play in the corners. Welcome the participants at the entry. (10 mins)**					

10:00 am - 10:30 am	Attendance & Prayer	Warm up session	10 minutes	-Participants sign on the attendance sheet.	PPT slide 37
	Recap	Recall the topics covered on the previous day<br>Set the agenda for the day	10 minutes	-Participants sit in a circlular shape and share their learnings/reflections from the previous day. The facilitator makes sure that every broad topic discussed the previous day is covered.	PPT slide 38
10:30 am - 11:00 am	Free play	Understand the benefits of free play for achieving developmental goals and how to organize it in the class	30 minutes	**Big group (30 mins)**<br>-Discussion on the steps of organizing free play by setting the four learning corners along with the points to remember<br>-Discussion on what materials were put in each corner and the reasons for doing so<br>-Discussion on how to create materials if the class doesn't have the required materials<br>-Discussion on the welcome activity (chart) as well as the attendance process (putting their names in a bowl)<br><br>-Facilitator will show UNICEF's video on free play.	-UNICEF Module 10: Video 4<br>-material to set 4 corners in the class<br>-slide on free play based on manual page<br>-Domain-wise material to set up the learning corners<br>PPT slides 39 and 40
	**Tea break + Energiser**				

11:00 to 11:50	What is mathematics, and what are the key components of mathematics?	-Understand what is teaching Maths in early grades and what are its key components<br>-Understanding the policy recommendations of teaching Maths<br>-Understand the classroom process to be followed for Maths :	50 minutes	-Co-facilitator divides the whiteboard into two columns. Do not name any column.<br>-Facilitator asks 'What is teaching Maths in early grades?' (take 6-7 responses)<br>-Every response that is an example of 'What is teaching Maths in early grades?' should be written in the first column. Facilitator asks 'What are the key components of Maths?'; Take a few responses till you complete all the components of Maths. Co-facilitator lists all the components of Maths on the board.	PPT slides 41 and 42
	Non linear approach of teaching mathematics in early grades recommended by Policy docs: NCF and NIPUN			-Facilitator to show the PPT slide for daily classroom process framework and discuss in detail.<br>-Facilitator to tell the manual page number on which framework of daily class process is given.<br>-Give a brief example of an activity for each component using the Phase II manual and connection with Grade 1-2 textbook	-phase II manual page PPT slide 43
11:50 to 12:50	Pre-math : Understanding the components:	Understand the components of pre-math	20 minutes	-Facilitator asks "What do we understand by pre-math?" (Take 2-3 responses). Co-facilitator to write all the responses on the whiteboard.	-Phase II & III manual of mathematics

				-Facilitator asks “Which components are covered under Pre-Math?”<br>-Facilitator explains each component (not more than 2 minutes) using the PPT and gives an example for each.	-Activity booklet: Manmoji ganit PPT slides 44 and 45
	Pre-math: Demonstration:	Understand the activities to be conducted for pre-math development	40 minutes	-Distribute the maths activity booklet in the small groups.<br>-Inform the participants that they need to prepare demonstrations of each component of pre-math using the activities given in the booklet (20 minutes).<br>-In the end, one group will demonstrate all the components to big group.<br>-The facilitator, co-facilitator, and the participants will share the feedback on the demonstration.	-Slides on math classroom process<br>-Phase II & III manual of mathematics<br>-Activity booklet: Manmoji ganit PPT slide 46
12:50 to 2:00	Number sense: Hands on practice of conducting various types of activities -Katta Pullalu :	Understand and conduct number recognition activities using tili-bundle	40 minutes	Katta Pullalu :<br>-Facilitator demonstrates the number recognition activity using tili-bundle<br>-Facilitator asks participants to recall the step by step process followed for reading the number chart and the co-facilitator will write the steps on the board.<br>-Participants practice in small group followed by demonstration before the big group.	-Number chart & copies of number cards<br>- Manual & Activity booklet: Manmoji ganit PPT slide 47

	Number sense: Hands on practice of conducting activity using number chart -Number Chart Reading :	Understand and conduct number recognition activities using the number chart	30 minutes	Chart Reading:<br>-Facilitator demonstrates the number recognition activity using the number chart<br>-Facilitator asks participants to recall the step by step process followed for reading the number chart and the co-facilitator will write the steps on the board.<br>-Participants practice in small group followed by demonstration before the big group.<br>-In the end facilitator will connect number recongnition activty to grade 1-2 workbook to practice the same by children	-Number chart & copies of number cards<br>- Manual & Activity booklet: Manmoji ganit PPT slide 48
2:00-3:00				**LUNCH**	
3:00-4:30	Games: Level-wise activity	Understand and conduct activities for number recognition	40 minutes	**Big group**<br>Facilitator to ask "What are the different Maths levels of children in the classroom?"<br>Discussion on different levels in mathematics and appropriate tasks for each levelled group (Beginner and 0-9, 10-50, 51-99).<br>Co-facilitator lists all the tasks discussed on board<br><br>**Small groups**<br>Ask the participants to look at the level-wise activities from the booklet in their smaller groups and practice 1-2 activities for each level	PPT slide 49 Activity booklet: Manmoji ganit

				Facilitator and co-facilitator will give feedback in small groups.	
	Word problem: Addition and subtraction Hands on practice of doing word problem with grades 1-2 children:	Understand how to solve word problems related to simple operations: addition and subtraction	40 minutes	-Facilitator demonstrates on word problem (example of word problem can be taken from the grade 1-2 math textbook)<br>-Facilitator discusses with the participants and the co-facilitator writes the steps on the board<br>-Participants practice in small groups followed by demonstration before the whole group<br>-In the end participants to write word problems (2 on addition and 2 on subtraction)	- sticky notes for writing word problems. PPT slide 50 Activity booklet: Manmoji ganit
	**Tea break + Energiser**				
4:30-5:30	Measurement & estimation in early grades	Understand measurement and estimation	45 Minutes	-Facilitator to discuss the main components of measurement<br>-Facilitator demonstrates on measurement and estimation activity to group<br>-Facilitator discusses with the participants and the co-facilitator writes the steps on the board<br>-Participants practice in small groups followed by demonstration before the whole group<br>-In the end, facilitator to give example of using grade 1-2 workbook to practice the same by children at home)	PPT slides 51 and 52

	Concluding teaching Math in grades 1-2	Revise the main components of teaching Math in the early grades and how to include them in the daily lessons	15 minutes	-Facilitator to discuss the math classroom process with group and make one day lesson plan for math class<br>-Facilitator to demonstrate the daily classroom process of math	PPT slide 53
5:30- 6:00	**Orientation on formats and training manuals**<br>**Writing Assignment: provide the two-fold blank card. Ask the KRPs to fill the sheet by writing 2 addition and 2 subtraction word problems.**				
**END OF DAY 2**					
**DAY 3**					
10:00-10:30	Attendance & Prayer	Warm up session	15 minutes	-Participants sign on the attendance sheet.<br>-Participants sit in a circular shape and share their learnings/reflections from the previous day. The facilitator makes sure that every broad topic discussed the previous day is covered.	PPT slides 57 and 58
	Recap	Recall the topics covered on the previous day<br>Set the agenda for the day	15 minutes		
10:30 to 11:30	Teaching Language in Grades 1&2 & Connection with Policy Documents : NCF & NIPUN	````	60 minutes	-Discussion on the components of language development (with a focus on reading development in early years and comprehension) connection with write up.<br>-Facilitator discusses the components for teaching reading with the participants and the co-facilitator writes the points on the board	- Page from manual of language development in early grades<br>-Watch the video from 7:00 to 8:45

				-Facilitator shows the slides of the daily classroom process for language and literacy development aligned with NIPUN Bharat's recommendation of teaching first language<br>-Using the ppt slide facilitator to conclude by showing the daily classroom framework for language aligned with grade 1 langauge textbook	PPT slides 59, 60, 61 and 62
	**Tea break + Energiser**				
11:30 to 2:00	Kaburulu (informal talk): Demo on Kabrulu in big group and hands-on practice of Kabrulu in small groups along with its step by step process :	Understand the skills that enhance oral language development	60 minutes	-Facilitator discusses and demonstrates the 5 different types of Kabrulu (informal talk) before the whole group and the co-facilitator writes them in the day-wise grid<br>-Participants practice all five types of Kabrulu (informal talk) in small groups with the help of the Chalo Khelen booklet<br>-Demonstration on one type of Kabrulu (informal talk) by each small group before the big group (picture, topic for informal talk should be from textbook)	PPT<br>- Phase 2 language manual page on "Baatchit/Kabrulu (informal talk)"<br><br>PPT slides 63 and 64
	Story related activities: Demo on story in big group and hands on	Understand the role of story in language development and how to translate this	60 minutes	-Facilitator discusses and demonstrates the story-related activities for the 5 days before the small group practice and the co-facilitator shows them in the day-wise grid (use the textbook story for the demonstration)	-PPT slides 65-69<br>- Phase 2 language manual page on "story

	practice of story related activities in small groups:	into classroom practice		-Facilitator discusses the similarities as well as differences between the steps followed on each of the 5 days<br>-Participants practice the same in small group followed by demonstration by a group before the whole group	related activity"
	Phonological Awareness: Demo on phonological awareness in big group and hands on practice of phonological awarenss in small groups : **Lead by Pandu**	Understand how to develop phonological awareness	30 minutes	**Big group (15 minutes)**<br>-Facilitator to demonstrate on activity for phonological awareness (word should be taken from the story)<br>-Facilitator asked participants "how does the phonological awareness game help children to learn language?<br>-Co-facilitators to write point comes from participation<br>-Facilitator to conclude by telling that phonological awareness skill is important to develop the reading and writing skill in the child. It helps children to<br>-understand the connection between sound and script and also develop metalinguistic awareness. Therefore this kind activities are important, now can you please tell me steps<br>-Co-facilitators pull the slide on phonological awareness while facilitator discuss in group<br>**Small Group 15 minutes**	-PPT slides 70-72<br>- Phase 2 language manual page on "Phonological awareness and its rationale"<br>-Section on phonological games in Chalo khelein for small group practice

				- After discussion, ask participants to practice the phonological awareness activity in small group (activities mentioned in chalo khelein booklet)<br>-Demonstration by the participants in a big group (by selective 1 group)	
2:00-3:00				**LUNCH**	
3:00- 4:30	Developing decoding skill: Guninthalu	Understand how to conduct different types of decoding activities in the classroom as per the need of children or phase	30 minutes	- Facilitator demonstrates Guninthalu reading activities.<br>- Co-facilitator writes down the steps as the facilitator discusses the activities.<br>**Small group**<br>- Participants to practice in small groups.<br>- Facilitator and co-facilitator move around to observe the groups practicing.<br>- Facilitator chooses one group to demonstrate before the whole group.	-PPT slides 73, 74 and 75<br>- Phase 2 language manual page on "Phonological awareness and its rationale"<br>-Section on Guninthalu in Chalo Khelain for small group practice
	Game: Level-wise activities to develop decoding skills:	Understand how to identify different levels in a language classroom and thereafter	30 minutes	- Facilitator discusses different levels in language and level appropriate tasks for them.<br>- Co-Facilitator lists the level appropriate tasks on the board.<br>**Small group**	-PPT slides 76 and 77<br>-copies of the manual and chalo khelain

		allocate level appropriate tasks		- Facilitator provides each group the Language Activity Booklet and asks them to practice some activities.<br>- Facilitator moves around and observes the small group practice.	booklet according to the number of groups
	Writing development:	Understand how to conduct the guided writing activity	30 minutes	- Facilitator shows the video of guided writing and then demonstrates the same.<br>- Co-facilitator writes the steps while facilitator discusses and demonstrates.<br>**Small group**<br>- Participants practice guided writing process in small groups.<br>- Facilitator and co-facilitator move around the classroom and observe.<br>- Facilitator concludes by listing other writing activities mentioned in Chalo Khelein Booklet.	-PPT slides 78, 79 and 80
	**Tea break + Energiser (10 minutes)**				
4:40 - 5.30	Textbook Transaction: Telugu, Math & English :	Understand how to use a textbook as resource material to develop foundational literacy and numeracy	30 minutes	- Facilitator asks the participants about the uses of a textbook for developing a one-day session plan on a given subject.<br>**Small Groups**<br>- Facilitator assigns each group a subject book (English, Telugu, or Math).<br>- Each group gives a presentation.<br>**Big Group**	-grade 1-2 textbooks & workbook of each subject: English, Telugu & math<br>-A4 size paper for

				- Facilitator gives feedback and covers any points not presented by the groups.	all the participants<br>PPT slides 81 and 82
	Material for grades 1-2 :	Understand how to identify and list the required material for classroom transaction	20 minutes	-Facilitator will explain the subject-wise material requirement<br>- In small groups, identify materials for English, Maths and Telugu	PPT slide 83
5.30 - 6.00	**Reflection & Writing Assignment: ask the KRPs to fill the sheet given yesterday with a story, 2 paragraphs, and 2 situations for Kabrulu (informal talk).**				
**END OF DAY 3**					
**DAY 4**					
10:00-10:30	Attendance & Prayer	Warm up session	15 minutes	-Participants sign on the attendance sheet.<br>-Participants sit in a circlular shape and share their learnings/reflections from the previous day. The facilitator makes sure that every broad topic discussed the previous day is covered.	PPT slides 86 and 87
	Recap	Recall the topics covered on the previous day<br>Set the agenda for the day	15 minutes		
10:30 to 11:30	Other Aspects of learning environment	-understand how positive teacher behavior looks like<br>-understand how to build a positive	30 minutes	**Small groups (15 minutes)**<br>- Distribute Phase III manual: pages on classroom environment, reading slot, etc.<br>- Each small group is assigned a group leader. The Facilitator gives each group leader a topic for their group.	Phase 3 Manual: pages on positive teacher behaviour, positive classroom environme

		classroom environment -discuss the strategies to build a reading habit in children -discuss how a print-rich classroom looks like		- Each group discusses a different component and list out 3 strategies for the same<br>**Big group (15 minutes)**<br>- The 4 groups present their ideas and the facilitator will be provide feedback.<br>- Facilitator discusses any strategies that were not covered by the groups and then summarizes the topics to conclude.	nt, building a reading habit, and a print-rich classroom<br><br>PPT slide 88
	Introduction to multi-grade, multilevel, and multilingual classrooms	Understand strategies for teaching a multi-grade, multilevel & multilingual classroom	30 minutes	-Facilitator to discuss the importance of the mother language (Telugu) to teaching second language (English) or school language (Telugu) in the classroom<br>-Facilitator to make groups to discuss strategies required to better handle multi lingual (different language), multi grade and muti level classrooms	- PPT slides 89-92<br>- Chart Paper
Tea & Energizer					

11:30 to 2.00	Second Language Learning: Exposure to English through visual and oral vocabulary building	Understand how to use posters and basic phrases to introduce and expand English vocabulary	2 hours	- Facilitator asks participants to list down some common greetings, expressions, and commands that can be used with and by the children.<br>- Facilitator and participants discuss different situations during which the expressions and commands can be used and the importance of repetition and practice to help a class learn these phrases.<br>**Poster**<br>- Facilitator shows the three posters to be used in the lessons.<br>- Facilitator explains and demonstrates the 3 activities to be used as per the PPT.<br>**Small groups**<br>- Facilitator assigns each group a poster.<br>- Groups demonstrate the classroom process for using a poster.<br>- Facilitator provides feedback and error correction to each group.	- PPT slides 93-96<br>- Posters
	Understanding the educational ecosystem and different stakeholder	Understand the position of potential stakeholers in the community, and their roles and contribution	30 minutes	- Facilitator discusses the importance of parents and the community in a child's learning.<br>- Facilitator highlights topics such as 'where a child's learning begins' and 'the involvement of people in the immediate environment'.	- Mother engagement booklet<br>- Phase II manual

	s in the community	towards learning		- Facilitator to quiz participants on the key takeaways from the session.	
2:00- 3:00	**LUNCH**				
3:00 to 4:10	Different strategies for parents involvement: Organizing Readiness Mela	Understand how to organize a readiness mela and their importance as a tool for communication and community involvement in a child's learning	50 minutes	- Facilitator shows the video on readiness mela and discusses the key steps from it.<br>- Facilitator provides a report card to participants and asks them to discuss domain wise activities, processes, and required domain wise material, which are central to a child being observed during the mela.<br>- Facilitator selects a group of participants to demonstrate the mela in big group.	-Video on Readiness mela<br>-Report cards (20 copies)<br>-PPT slides 97-99
	Different strategies for parental involvement:<br>1. Formation of mothers groups<br>2. Parents' meetings,<br>3. Sharing idea cards	Understand how to use different strategies to mobilize community stakeholders towards learning	20 minutes	- Facilitator asks 'how can parents be reached systematically for their involvement in their child's learning?' (co-facilitator writes responses on the board).<br>- Facilitator discusses the formation of mother groups, how frequently they meet, how to conduct parent meetings and more.<br>- Facilitator to conclude using PPT slide.	-PPT slides 100 and 101<br>- Mother engagement booklet

4:10 to 5.30	Assessment in Early Grades	Understand the meaning and importance of assessment	20 minutes	- Facilitator asks participants to "write a word on what comes to your mind about assessment" in their notebooks.<br>- Facilitator writes 5 - 7 words on the whiteboard after taking inputs from participants.<br>- Facilitator asks participants 'what is the meaning of assessments?'<br>Video showcasing and discussion<br>- Facilitator plays the UNICEF video and presents the PPT on Assessment.<br>- Facilitator summarizes the purpose and importance of assessments.(Slide 4, 5 and 6)	Assessment PPT, UNICEF Video (Module 13A Video 4)
	Assessments and Policy	Understand how to link concepts such as 'competencies' and 'learning outcomes' with policy and understand how they are relevant to assessments	20 minutes	- Facilitator starts the session by explaining assessments as per NCF for Foundational Stage (Slide 8).<br>- Facilitator asks the participants about their understanding of word competencies and learning outcomes.<br>- Facilitator explains the concepts of Curricular Goals, Competencies and Learning Outcomes (Slide 9, 10).<br>- Facilitator closes the discussion by going through the summary points mentioned on the PPT.<br>- Facilitator concludes by conducting a quick 3 question quiz on the topics covered.	Assessment PPT, Set of NCF documents on assessments

	**Tea break + Energiser**				
	Understanding and Communication of Learning Levels	Understand how to guage a child's progess and the benefits of communicating the same to parents and other community stakeholders	30 minutes	- Facilitator discusses the importance of understanding and communicating a child's progress with the participants.<br>**Small group**<br>- Facilitator distributes case studies to each group.<br>- The groups prepare a 5 minute and present how they would communicate the case studies they have been given (this exercise will be given 10 minutes).<br>**Big Group**<br>- Facilitator summarizes the session by discussing the 'do's and don'ts' of communication of learning levels and progress.	Set of report cards, case studies printouts
	Post Training Quiz	Understand and gauge the knowledge of participants after the training	10 minutes	Facilitator will give the following instructions:<br>- Open the link shared on the WhatsApp group.<br>- Everyone will attempt the quiz individually without discussing it with anyone.<br>- Co-facilitator will share the link on the WhatsApp group as well as display the slide with the link.<br>- Ask participants to complete the quiz in the given time.	Quiz, PPT
5.30 to 6.00	**Reflection, allocating sessions for Day 4 (for demos)**				
**END OF DAY 4**					

DAY 1

UNDERSTANDING THE AGE OF 3-9 YEARS

Day 1: Session 1
6-day training for teaching Grade 1-2 children

The first session of the first day is divided into four main sections. The total duration of this session is 1 hour 30 minutes. This session will cover the topics mentioned below:

1.1 Inauguration and Introduction
1.2 Setting ground rules for the 4-day session.
1.3 Pre-Quiz
1.4 Overview of the SALT project & 60 days course for Grade 1-2 teacher

1.1 Inauguration & Introduction	
**Pre work**	● Set up a registration/attendance table at the entry of each training hall/room: make sure that attendance should be done before the session starts.<br>● Setting up the projector, relevant ppt on screen and required materials: stationery and printouts of required material in required quantity.<br>● Co-facilitator must be ready with music which is going to be played for the introduction round.
**Objective**	Familiarization with 6 days training and introduction to each other
**Total Duration**	30 minutes
**Process of Facilitation**	**Introduction**<br>● The facilitator provides the attendance sheet and asks participants to sign on the attendance sheet.<br>● The facilitator welcomes everyone to the course. The facilitator introduces themselves and the co-facilitator.<br>● Facilitator gives instructions on how the introduction activity will be conducted: Co-facilitator will play music for 7-8 seconds while participants will move around the room randomly. When music is paused, participants will form groups of 5 with people standing near them and introduce themselves by<br>a. Telling their name and,<br>b. telling their school.<br>● Repeat this process 2-3 times.<br>**Inauguration**<br>● Participants sit in a big group (circle) and the facilitator will inaugurate the 6 days training using the PPT.
**Classroom arrangement**	Big group
**Material Required**	PPT slides 3-4, audio file

Note:
Co-facilitator should make use of the attendance sheet to collect the contact numbers of all the participants. By the end of this session, a WhatsApp group with all the participants should be formed.

1.2 Setting ground rules for the 6-day session	
**Objective**	To bring the all participants on common ground where everyone agrees on a set of rules for the session and have common understanding of the 6-day workshop
**Total Duration**	20 minutes
**Process to Facilitation**	**Rule setting**<br>• Co-facilitator will put 1 chart on wall with the heading 'ground rules' and then give sticky notes to every participant.<br>• Ask participant to write rules on sticky note and paste it on the chart.<br>• Facilitator must reiterate that these rules would be followed by each person in the room including the facilitator as well
**Classroom Arrangement**	Big group
**Material**	PPT slide 5, chart paper and double-sided tape, sticky notes (3-4 different colors), markers

Note: *Co-facilitator makes sure that chart is pasted on the wall before or during the conversation.*

1.3 Quiz: Baseline	
**Objective**	Assessing participants' understanding of the content to be covered in the 6-day session
**Total Duration**	20 minutes
**Process to Facilitation**	• Facilitator will give the following instructions:<br>-We will open the link shared on the WhatsApp group formed in Session 1.<br>-We will attempt the quiz by ourselves, without discussing it with anyone.<br>• Co-facilitator will share the link on the WhatsApp group as well as pull out the link slide.<br>• Ask participants to open and complete the quiz in the given time.
**Classroom Arrangement**	Big group
**Material**	PPT slide 6, quiz link

1.4 Overview of the certificate course	
**Objective**	Understand the course overview and objective of SALT
**Total Duration**	20 minutes

**Process to Facilitation**	Lead facilitator will brief about the project 'SALT' and course overview with respect to their roles and responsibilities for the 60 days certificate course using the slides
**Classroom Arrangement**	Big group
**Material**	PPT slide 7-11

For Facilitator: Understanding of Age 3-8 Years

Early Grades refer to children who are in grades 1&2. They are usually in the age group of six to eight. Though the National Education Policy 2020 recommends that the age of entry into grade 1 should be six years, currently children in some states of India also enter grade 1 at age 5. These grades are the first two years of a child in the **formal school** system, as they transition from preschool or Anganwadi to school. The first two school years of a child are not only important for the development of the brain but for **overall holistic development** as well. As a teacher you can help the child to develop to their full potential by providing a nurturing and stimulating environment in class. We will learn more about how this can be done in Chapter 3 of this module.

These years lay the foundations for **early literacy and numeracy**. This prepares them for the coming school years so they can perform and understand concepts later. ASER 2022[1] reports that only 20.5% children in grade 3 are able to at least read a simple Grade II level text and only 17.6 % children in grade 3 are able to do simple subtraction (2 digit subtraction with borrow). As a result, if children are not able to read simple text and do subtraction, they may not be able to grasp the higher-level text and concepts or cope with the syllabus expectations of grade 3.

Day 1: Session 2

Understanding Children in the Ages of 3-8 Years

The second session focuses on the understanding of children in the age group of 3-8 years in the light of brain development, factors and how a child learns. In order to understand the foundational stage, this session is divided into 4 main sections. The total duration of this session is 2 hours and 30 minutes. This session will cover the topics mentioned below:

2.1 Brain Development in ages 3-8 years
2.2 ECCE: Understanding of age 3-8 years with respect to early childhood care and education.
2.3 Holistic Development: Key Developmental Areas
2.4 Transition in early years and stage wise progression recommended by polices.

[1] http://img.asercentre.org/docs/ASER%202022%20report%20pdfs/All%20India%20documents/aserreport2022.pdf pages 64 & 65.

**2.1 Brain Development in ages 3-8**	
**Pre work**	Co-facilitator must be ready with the UNICEF videos which are going to be shown.
**Objective**	Understand brain development, the factors affecting brain development, and the importance of a positive stimulating experiences for development
**Total Duration**	40 minutes
**Process of Facilitation**	**Brain development**<br>● Facilitator will ask the participants what comes in your mind when you hear 'brain development.'<br>● Co-facilitator will write all the words that come up on the whiteboard. Take a maximum of 6-7 responses. (Spend 5-10 minutes of discussion and take response from participants)<br>● Show the UNICEF video on the process of brain development (Mod 1: Video 6) (remind participants to take notes before starting the video). (2-3 minutes)<br>● The facilitator talks about the difference between brain growth in terms of size and brain growth in terms of development and concludes using the PPT.<br>**Factors affecting brain development**<br>● Show the UNICEF video on the factors affecting brain development (Mod 1: Video 8) and the next video on positive stimulating experiences (Mod 1: Video 9)<br>● The facilitator concludes using the PPT.
**Classroom arrangement**	Big group
**Material Required**	PPT slides 12-14, UNICEF Module 1:<br>Video 6, video 8 video 9

For Facilitator: Brain Development

1. Brain Development: A child's brain develops rapidly in the first 6 years of their life. In the early years the brain develops due to growth in the size of neurons and neural connections being formed, which is maximum during the first three years of life. The brain is 95% of its adult size (in terms of volume) when the child reaches the age of six years. This development continues through the remaining childhood and adolescent years. Studies have shown that for children in 3 to 6 years, the most rapid growth is seen in the frontal lobe of the brain. The frontal lobe is involved in planning and organizing new actions, and in maintaining new actions to tasks.

This helps in self-controlling actions like walking, clapping. It also helps to think which helps to further build their intentions. It is responsible for building the overall personality of an individual.

- **Occipital lobe:** *vision*
  This helps the person to see the world around them.

- **Temporal lobe:** *hearing, language processing, memory*
  This is responsible for hearing and listening and learning language. It further builds memory which helps to remember.

- **Parietal lobe:** *registering spatial information, attention, motor control.*
  This helps to understand the size, shape, texture, etc. of objects around a person. Also, it helps to build attention and build better control of physical activities (writing, jumping, etc.).

2. Factors affecting Brain Development
As teachers, it is important for us to understand the factors which might help in the development of the brain. The development of the brain depends on mainly 2 factors:

**Heredity**	**Environment**
Through mother and father (genes)	Experiences of interactions and situations.

Heredity: These are the characteristics/traits acquired through chromosomes of the biological mother and father. This depends on what a child inherits from their parents and is very difficult to change.

Environment: The immediate environment of the child which is the home, school, *Anganwadi* and any other place where the child spends the maximum time affects the development of the child's brain. Deprived environments like no children around, less food, issues within family, no interaction, no response from caretaker or any other challenging situation leads to depressed (or less) brain activity. Due to this the child might not be able to go through the proper brain development process. In conclusion, everything impacts the development of the brain from what they see, hear, smell, touch, the people they interact with and the language they are exposed to. However, it is important to note that early intervention by providing a stimulating environment can help the brain to reorganize neural connections and can reverse the adverse outcomes on brain development. Therefore, the role of the teacher in providing this stimulating environment in the early years becomes extremely crucial.

2.2 ECCE: Understanding of age 3-8 years with respect to Early Childhood Care and Education

**Pre work**	Co-facilitator must be ready with videos which are going to be shown.

**Objective**	Understand ECCE landscape and its importance as foundational stage
**Total Duration**	20 minutes
**Process of Facilitation**	***ECCE***<br>● The facilitator will write the word “ECCE” on board and ask participants to tell the meaning.<br>● Co-facilitator will write the definition of ECCE-early childhood care & education.<br>● Facilitators ask the meaning of care and education in the age of 3-8 years and will conclude using the PPT slide
**Classroom arrangement**	Big group
**Material Required**	-Whiteboard & markers,<br>- PPT slides 15-16<br>-UNICEF course Module 1: Video 11

For Facilitator: Early Childhood Care & Education

ECCE is defined as the care and education of children from birth to six years. The first six years of a child’s life are truly critical and lay the foundation for lifelong well-being, and overall growth and development across all dimensions- physical, cognitive, and socio-emotional. The aim of ECCE is to facilitate optimum development of a child's full potential and lay the foundation of all around development and lifelong learning. Parents and home are primarily responsible for a child's well-being, however, ECCE centers, *Anganwadi* and the community must work together to meet the following objectives.

- Ensure each child is valued, respected, feels safe and secure and develops a positive self-concept.
- Enable a sound foundation for physical and motor development of each child.
- Imbibe good nutrition routines, health habits, hygiene practices and self-help skills.
- Enable children for effective communication and foster both receptive and expressive language.
- Promote development and integration of the senses.
- Stimulate intellectual curiosity and develop conceptual understanding of the world around by providing opportunities to explore, investigate and experiment.
- Enhance development of social skills, social competence and emotional well being
- Develop a sense of aesthetic appreciation and stimulate creative learning processes.
- Imbibe culturally and developmentally appropriate behavior and core human values of respect and love for fellow human beings.
- Enable a smooth transition from home to the ECCE center to formal schooling.
- Enhance scope for overall personality development.

ECCE ideally consists of flexible, multi-faceted, multi-level, play-based, activity-based, and inquiry-based learning, comprising of physical, social, emotional, and emergent literacy and numeracy skills (alphabets, languages, numbers, counting, colors, shapes, indoor and outdoor play, puzzles and logical thinking, problem-solving, drawing, painting and other visual art, craft, drama and puppetry, music and movement).

2.3 Holistic Development: Key Developmental Areas & Appropriate Activities	
**Pre work**	Co-facilitator must be ready with videos which are going to be shown.
**Objective**	● Understand key developmental domains for the child and conceptualize with activities.<br>● Understand developmentally appropriate activities and age-appropriate development stages
**Total Duration**	40 minutes
**Process of Facilitation**	***Holistic Development***<br>● The facilitator will write the word “Holistic Development” on board.<br>● Facilitators ask the participants to watch a UNICEF video and ask them to write the key point while watching it. (2-3 minutes)<br>● Conclude by asking 'What is holistic development and developmental area?'<br>***Developmentally appropriate activities***<br>● Divide the group into 8 small groups and provide the chart to each group (5 minutes)<br>● Facilitator will give specific development areas to each group and ask them to write the list of activities (10 minutes). For e.g., group 1 will do physical domain and write activities on it.<br>● Group will paste their respective chart papers on wall (5 minutes)<br>● Facilitator will summarize the developmentally appropriate activities in relation to 3-to-8-year age group (10 minutes).
**Classroom arrangement**	Brainstorming in Big group & task in small group
**Material Required**	Whiteboard & markers,<br>-PPT slides 17-18<br>UNICEF Module 2: video 4

For Facilitator: Holistic Development & Appropriate Activities

All round development: Holistic Development

Early years are the formative years of child development. They form the basis for foundational learning. Early years refers to preschool years and grades 1 & 2. Holistic development in the early years emphasizes different developmental areas i.e., physical, language, cognitive, socio-emotional, aesthetic and creative development of a child. If these domains are developed equally well, in a balanced manner, it ensures all round or holistic development of the child. Encouraging holistic development is crucial to maximize a child's learning experience in a stimulating environment and fostering lifelong learning. It establishes social adaptability alongside emotional intelligence along with developing physical, cognitive, language and aesthetic development comprehensively. It

helps in building social-emotional competence, linguistic proficiency, physical growth, & helps to attain cognitive competencies. It lays the foundation for lifelong learning.

Physical development: The physical domain covers the development of physical changes, which includes growing in size and strength, as well as the development of both gross motor and fine motor skills.

Cognitive Development: 'Cognitive development' can be defined as the development of a child's ability to think and reason. Cognitive abilities associated with memory, reasoning, problem solving, and thinking continue to emerge throughout childhood; it involves gaining and managing knowledge and learning to use it.

Language Development: Language development involves acquiring skills for oracy and literacy. It involves skills for: Speaking; the ability to use language to communicate ideas, thoughts, and feelings, listening; ability to listen to and understand language, reading; ability to read the text with comprehension and writing; ability to express, communicate ideas, thoughts in writing.

Socio-emotional Development: Social and emotional development serves as the foundation for relationships and interactions that give meaning to a child's experiences in the home, at school, and in the larger community. Social-emotional development includes the child's experience, expression, and management of emotions and the ability to establish positive and rewarding relationships with others.[2]

Developmentally Appropriate Activities

Activities that are designed based on the ability and age of children are developmentally appropriate activities. These developmentally appropriate activities should be created for all domains of development. They should also be designed adopting a play based approach. The classroom activities should be comprehensive to achieve the goal of holistic development. Following is the description of developmental domains and sample activities pertaining to each domain.

Activities for Physical Development

This is an important domain especially in the early years. All activities may require components of the physical domain, but specific activities should be conducted in the classroom to enhance skills for fine and gross motor development. Gross motor development refers to the movement of large muscles in the body such as arms and legs. Fine motor development refers to the movement of small muscles such as fingers, wrists and hand-eye coordination. Following are the activities for gross and fine motor development:

- **Gross Motor Development:** Activities for gross motor development include throwing, catching, skipping, running & jumping. Games for walking may include walking on a straight line, walking on a zig-zag path, crossing the obstacles and lemon race.
- **Fine Motor Development:** It involves the skills for holding and grasping using coordinated eye-hand movement. Activities for fine motor development include drawing, coloring, paper folding, and beading. For example, making paper almirah using two folds or making a paper boat using multiple folds.

Note: These activities should be conducted from easy to difficult. These activities should be conducted according to the developmentally appropriate level of children. Scan the QR code to watch the activity.

Activities for Cognitive Development

Cognition is an important feature of human beings and many decisions we make in our day-to-day life about choosing to do something or refusing to do something involve cognitive processes. It allows the ability to think

[2] Cohen and others 2005

and reason. There are a range of competencies to develop cognitive skills in early grades. Given below are some of the cognitive skills that develop:

- Pre-math: Activities for developing pre-math through skills of pattern, comparison, classification, shapes. The nature of these activities is from simple to complex, for example, pattern activity can be done orally, using concrete objects and then written patterns in form of shapes, numbers can be followed. Scan the code to watch the activity.
- Problem solving: Problem-solving is a self-directed, cognitive-behavioral process in which an individual seeks to identify or discover solutions for problems that arise throughout everyday life (D'Zurilla, Nezu, & Maydeu-Olivares, 2004). Activities can include solving puzzles which can range from solving 3-4 pieces of puzzle to 7-8 pieces-puzzle and solving day-to-day life situations.
- Number Sense: There can be activities to develop mathematical concepts pertaining to number recognition, operations and more. The learning of the concept should range from simple to complex, for example, concepts related to number understanding such as big/small number, before/after number, expanded form and more can be done with 1-digit numbers and then gradually move to 2-digit numbers.

Activities for Language Development

Children develop language skills innately. Children acquire skills of speaking and listening through their immediate surroundings. Skills for literacy; reading with comprehension and writing are learnt in the early years. The development of language and literacy in the early years requires a wide range of abilities. Following are the activities for developing language skills:

- Phonological skills help in reading and writing. Activities for developing phonological skills involve identification of phonic sounds, segmentation of two-three syllable words, writing the segmented sounds and forming the words.
- To enhance reading comprehension, children should be provided enough opportunity to read stories and simple paragraph reading cards.

Note: *More activities for enhancing language & literacy development will be shared through later chapters in the course module.*

Activities for Socio-emotional Development

Classroom integration of socio-emotional activities is crucial. Socio-emotional development refers to the ability of the child to understand and express his/her own emotions as well as relate and understand emotions felt by others in the immediate surroundings.

- Socio-emotional skills develop through children playing together, interacting, participating in classroom activities, working in groups, expressing themselves. These skills can be carved out through a variety of activities such as giving situations to the children and asking them how they will respond to it. Cross cutting and separating activities are important to develop these skills.
- Facilitators can hold discussions on the situation provided. For example, 'How do you feel when your parents scold you?'. Each child should be given the opportunity to respond and be allowed to express themselves.

2.4 Transition in Early Years & Stage wise progress recommended by policies	
**Pre work**	Co-facilitator must be ready with videos & slides which are going to be shown.
**Objective**	Understand transition in early years & stage-wise progress recommended by policies
**Total Duration**	50 minutes
**Process of Facilitation**	● Using the PPT slides, facilitator will explain the importance of the transition period from age 3-8 and its connection with policy. New structure and continuum of age 3-8 years.<br>● Facilitator will explain stage wise progression taking of one example of competency (using example of any one domain)<br>● Opportunity for question/answer<br>● Provide reading for assignment to the participants from NIPUN. Participants to tell important takeaways (Co-facilitator to call out few names to share the takeaway)<br>● Facilitator will conclude through an open discussion on the challenges faced with Grade 1 children if they are not coming from preprimary (Co-facilitator will write selective and appropriate points on the board). Facilitator will emphasize that the best way to deal with the challenges is dedicating 3 months to school readiness.
**Classroom arrangement**	Big Group
**Wait Material Required**	PPT slides 19-23

For Facilitator: Transition in Early Years

Building the right foundations in the early years can lead to a substantial improvement in the life chances of an individual and enable a more productive journey ahead. The early years must be viewed as a continuum that begins before school and continues into the first two years of schooling (Grades 1-2). By undertaking age-and stage-appropriate interventions with children in their early years, one can ensure strong foundational skills and prepare children for the expectations in school and beyond.

With prolonged school closure, schooling and learning both have been disrupted. Apart from ensuring enrollment and attendance, all school systems face a major challenge - building a strong base for learning in classes 1 & 2. Children who are going to enter classes 1 & 2 will have had no exposure to early education in the past year. Targeted activities need to be done to help their transition into formal school. The National Education Policy 2020 also puts high priority on achieving universal foundational learning in early grades. Hence the proposed program will fit well under the overall objective of NEP 2020.

Before any ‘at-grade’ teaching can begin as the new academic year begins, it is imperative that the gaps previously mentioned are addressed in a systematic manner. Hence, **a focused learning program for a duration**

of 2-3 months Std. 1-2 is recommended. The core elements, objective and sample framework have been detailed in the following sections. The proposed strategy has been divided into three phases. These are:

- **Phase 1** will aim to make children ready for school through direct input in class and active parental support at home.
- **Phase 2** will aim to impart all-round development and foundational skills to children over a 2–3-month long period. Along with classroom teaching this phase will aim to make children ready for school through active parental support at home.
- **Phase 3** After Phase 2, it can be decided on how to move forward with grade-based activities.

Linkage with three developmental goals for foundational stage: NEP 2022 & NIPUN

The NEP 2020 has focused on the holistic development of the child. There are different domains of development like physical and motor development, socio-emotional development, literacy and numeracy development, cognitive development, spiritual and moral development, art and aesthetic development which are interrelated and interdependent. All these domains have been subsumed into three developmental goals[3] as per NIPUN Bharat:

1. Developmental Goal 1: Children Maintain Good Health and Well-being.
2. Developmental Goal 2: Children Become Effective Communicators
3. Developmental Goal 3: Children become involved learners and connect with their immediate environment.

Moreover, key competencies and concepts of each goal have been highlighted and these competencies have been drawn from the documents 'Pre-school Curriculum' and 'Learning Outcomes' developed by the NCERT. Likewise, the perspectives of mathematical thinking, design learning etc. as envisaged in the NEP 2020 has been taken care of.

Day 1: Session 3

Implementation Structure

Third session of day 1 designed to help the trainees to understand the objective, structure and phase wise transition of one-year long duration intervention with children of grade 1-2. The session will be completed by covering three major components. This session will cover the mentioned topics.

3.1 How do children learn.
3.2 Working with grades 1-2: phase-wise transition in one academic year and its objective.
3.3 Readiness Phase and Vidya Parvesh

[3] Nipun Bharat 2021, page 157, para 7.1

3.1 How do children learn	
**Pre work**	Co-facilitator must be ready with videos which are to be shown.
**Objective**	Understanding how children learn
**Total Duration**	20 minutes
**Process of Facilitation**	● Facilitator will ask participants - If the brain develops in a rapid way then how does a child/brain learn?<br>● Facilitators ask the participants to watch a UNICEF video Module 2: Video 8 'How Children Learn?' and note down the key points while watching it<br>● Brief discussion on each of the principles of how children learn in depth<br>● Conclude by showing the slide “7 key principles of learning”
**Classroom arrangement**	Big group
**Material Required**	● Whiteboard & markers<br>● PPT slide 24<br>● UNICEF Video: Module 1: Video 8(Intro)

For Facilitator: How do children learn

While dealing with children it is important to understand human behavior. There have been various philosophers and theorists who have come up with their theories. Studies have taken the whole lifespan approach while determining the theories of development.

Cognitive Development Theory – Jean Piaget: Piaget laid down the four stages of cognitive development for children. According to this theory two processes are involved: organization and adaptation. One organizes their experiences and observations to make sense of what is going on around us. Additionally, we adapt to adjust to new and changing demands of the world around us. According to Piaget there are four stages as follows[4]:

Socio-cultural Cognitive Theory – Lev Vygotsky

Vygotsky focused on how culture and social interaction play a major role in cognitive development. One can’t separate social and cultural activities from a child’s development. According to him, language, mathematical systems and memory strategies are all inventions of society which a child is learning. For cognitive development, it is important they interact with more skilled adults and peers, in order to learn, adapt and be successful.

Information-Processing Theory

[4] Santrock, pg. 25 (fig 1.12)

The information-processing theory focuses on how individuals manipulate, monitor and strategize information. According to this, there are no set stages. However, humans learn by slowly increasing their capacity for processing information. This helps them later to have complex knowledge and skills. Thinking is information processing, where one perceives, encodes, represents, store, and retrieve information.

Operant Conditioning – B.F. Skinner
Operant conditioning depends on changes because of certain behavior. **Behavior** refers to the child's activities and changes refer to the result which is **reward or punishment** based on the act/ behavior of the child. For example, a child is often rewarded by clapping in class when they recite the poem nicely/ getting a treat from parents when they score better marks. Consequently, there will be a change in the behavior of the child where they will be more responsible to study/learn to get these rewards (result).

Social Cognitive Theory – Albert Bandura
Social cognitive theory highlights that key factors in development are behavior, environment, and cognition[5]. It focuses on how cognitive processes are linked with environment and behavior. According to Bandura, learning occurs through observing what others do. This is why people acquire different behaviors, thoughts, and feelings by observing others.

Ethological Theory
Ethology stresses that behavior is strongly influenced by biology and is characterized by critical/sensitive periods (time frames where certain experiences mothers as soon as they are hatched. Lorenz in his experiment separated eggs laid by one goose in a group of two. One group was kept with a goose and another with Lorenz. As soon as the hatching happened the group with the goose followed the mother goose. For the second group, where Lorenz was present the babies followed him. Soon, he put the set of two goslings in one box. Mother Goose and mother Lorenz stood in different directions, and each group went to their 'mother'. Lorenz called this process as imprinting that is rapid (very fast) innate learning which involves attachment to the first moving object seen.

Bowley emphasized attachment to caregivers over the first few years of life. This attachment has important consequences throughout life span. If the child has positive and secure attachment, they will develop positively in childhood and adulthood.

Ecological Theory – Urie Bronfenbrenner
Ecology refers to studying the relationship between living things and surroundings. According to this theory, a child develops in a complex system of relationships. These systems get affected by multiple levels of the surrounding environment. There are four systems according to this theory[6].

Theoretical Perspectives on Child's Learning: There have been various views on how children learn or how one needs to create such an enriching environment for them to learn.

Western Perspectives[7]

Jean Jacques Rousseau
- Believed that children should be allowed to express themselves freely.
- In early years, socio-emotional adjustments are more important than mugging up information.
- Teacher should help them explore, so that they learn on own *(free play)*

Johann Pestalozzi

[5] Santrock, pg. 27 (fig 1.13)
[6] Santrock, pg. 29 (fig 1.14)
[7] Foundations of Human Development: A Lifespan Approach (pg68)

- Education should be based on natural development, self-discovery, a child's interest, and own experience.
- Teaching methods should fit individual needs of each child

Fredrich Froebel
- Emphasized on group activities and social development.
- Play is central to knowing child's inner thoughts and social world

John Dewey
- Believed that realities of everyday life should be basis of classroom activities *(kitchen set, doctor set)*
- Play activities have potential to build problem solving abilities.
- Teacher's role is to encourage social skills by planning, organizing, and providing opportunities

Maria Montessori
- Montessori activities were organized for individuals, and children were allowed to choose how, and for how long, they wanted to use the material

Jean Piaget
- Believed that children construct their own knowledge, and they should be given freedom to play, experiment and participate in guided learning activities

Lev Vygotsky
- Believed that children learn best with help of others

Indian Perspectives[8]

Tarabai Modak
- Gave importance to socio-cultural approach to the education of children, and created learning centers within spaces in the community

Gijubhai Badheka
- Believed that children learn in interactive environments, and created material on parent engagement (*Mata Pita se Batcheet*)
- Created awareness of the multiplicity of caregivers, who need to work together in socialization of child

In conclusion, during early years children learn through play-based activities. They learn to think, remember, and solve problems. For example, playing 'house-house' helps children to create stories and learn about the roles of family members. They learn best when they are given a free and healthy environment to experiment, succeed-fail, learn and hence, develop to their full potential. Each child should be treated as an individual who learns at their own pace, which should be appreciated by the teachers.

Principles on How Children Learn: There are seven principles on how children learn in early years.

1. Children learn by doing.
2. Children learn from concrete experiences.
3. Learning for children should follow a sequence of simple to complex.
4. Learning for children from what they already know
5. Children learn through play.
6. The social context of children influences their learning.
7. Children learn through everyday activities.

[8] Foundations of Human Development: A Lifespan Approach (pg68)

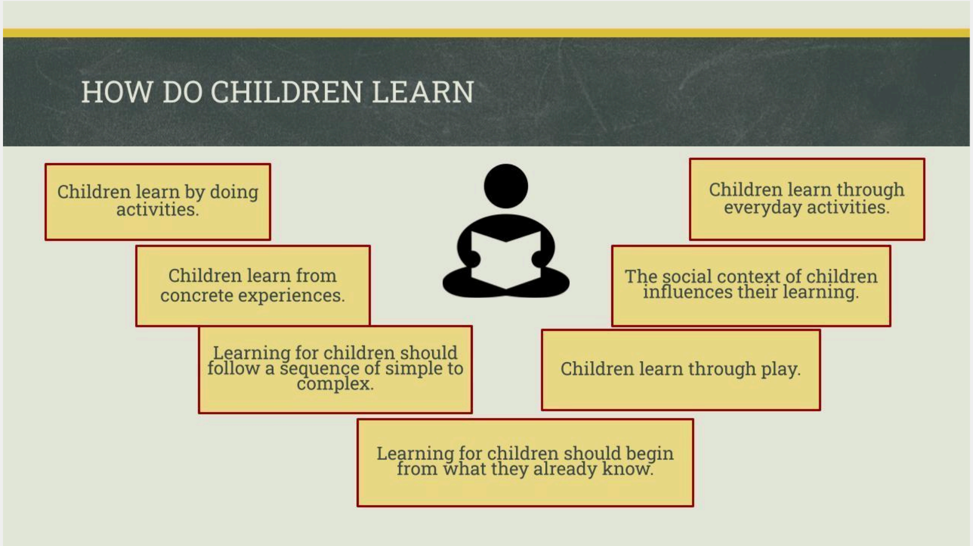

3.2 Phase-wise transition in one academic year and its objective	
**Pre work**	Co-facilitator keeps slides which are going to be shown ready.
**Objective**	Understand the objective of working with early grades and its phase wise transition in one academic year
**Total Duration**	20 minutes
**Process of Facilitation**	• Facilitator to discuss the objective of working with grade 1-2 children in a yearlong model.<br>• Co-facilitator can write 1-2 participants response.<br>• Facilitator to explain the objective of the program using the objective slide.<br>• Facilitator to explain the phase wise transition in one year cycle to achieve the same
**Classroom arrangement**	Big Group
**Material Required**	-PPT slide 25

For Facilitator: School Readiness classroom transition

Significance of school readiness in early years: Generally, the period of birth to age of 9 years is referred to as the early years[9]. It's a crucial age, as the growth, development, and learning start before birth and continues through the lifespan. It means the period is not only important for brain development but also for developing the foundational skills of the child for lifelong learning. Therefore, it is crucial to provide appropriate input, exposure, and opportunity, in order to build a solid and broad foundation[10] for lifelong learning and wellbeing. The age of 3 to 9 years should ensure the holistic[11] development of a child's social, emotional, cognitive, and physical skills. This requires appropriate inputs delivered through a play-based teaching approach to develop the foundation skill for lifelong learning.

Research proved that the first 8 years of the children are crucial from the point of growth, development, and learning. Therefore, the National Education Policy emphasizes on (a) transition and linkage between pre-primary to primary till grade 3 in the light of continuum of age 3-9 years. (b) the significance of Foundational Literacy & Numeracy (FLN) for children's overall development and learning. It has accorded the highest priority to the universalization of FLN[12] for all children by the end of grade 3 by 2027. Given this policy push several state governments have launched or are launching their own FLN missions as well as focusing (a) the pre-primary, whether running into school premises or in Anganwadi center (b) 3-month readiness focus intervention in the starting of grade 1 (c) building the capacity of Anganwadi workers and grade 1 teachers through Nishtha.

> "Children who enter school without having mastered specific skills or little or not ECCE experiences, primary curriculum should include child initiated as well as teacher supported activities, and should emphasize hands-on, integrated learning to boost their school readiness," NIPUN Bharat 2021, pg. 169

Meaning of school readiness: No matter what you call it? Whether you're calling 'Getting Ready for school', 'Warm up phase', 'School preparation' or 'School Readiness', the meaning as well as an objective of this is to help children to prepare for school and lifelong learning. School readiness[13] (official term) means smooth and comprehensive transition from home to preprimary and preprimary to school. The term school readiness is often misunderstood by parents, teachers, and school as a period of preparation for developing literacy and numeracy skills in the child. Though, this must be considered as the period of growth, development, and learning. Seeing this as school readiness as a mere preparation would be a mistake. 'Self-learning' and 'learning in school' should not be seen as two different concepts. The school readiness indicates to us to realize that in learning, there is no need to force other experiences on children or not to accelerate with the process itself.

> "Children, schools and families are considered ready when they have gained the competencies and skills required to interface with the other dimensions and support smooth transition of children from home to Early Childhood Care and Education (ECCE) center and subsequently to primary school". NIPUN, pg. 25.

Importance of school readiness: In schools, you will find 5–8-year-old children either enrolled in grades 1 or about to be enrolled in the same. Often, we expect that by the end of grade 2, these children will be able to obtain

---

[9]NIPUN Bharat, pg. 60, para 2.1, that talks about the need for foundational skills development in the early years (age group 3-9).

[10] Foundation for lifelong learning refers to the ability to read and write, and to perform basic operations with numbers, is a necessary foundation and indispensable prerequisite for all future schooling and lifelong learning.

[11]Holistic development means that education should not focus only on cognitive skill development rather focus on building individual character and well-rounded individuals having 21st century key skills.

[12]The Mission will focus on children of age group of 3 to 9 years including preschool to grade 3, children prior to age 5 and before entering to class I will be attending Balvatika in Anganwadis or primary schools having preprimary sections (NIPUN 2021)

[13] UNICEF, 2012. School Readiness: A conceptual Framework

basic language and math skills. Similarly, at the entering of grade 1, we expect children to work in big or small groups, interacting with other children, expressing themselves as well as listening to other children and understanding the academic instructions. Unfortunately, three different types of children can find in preprimary/enter in the grade 1: (a) children who are directly enrolled in grade 1 (b) children coming from Anganwadi whether they get quality preprimary education or not and (c) children coming from low-cost private preprimary setting. In order to make children ready for formal schooling, focus and specific input is required here. Therefore, facilitators have a good understanding of the school readiness aspect and its importance as well as being able to conduct the appropriate activities in their respective classrooms to help children to acquire the required skills.

Readiness of children for school: Ready children[14] for school indicates achieving those skills that help children to deal & cope with future schooling and lifelong learning. For example:

(a) Working in small groups or actively participating in classroom activities opens a window for children to use different sources to learn as well as provide a platform where children can learn from others too.

(b) Having a tripod grip on crayon wax while coloring a picture or drawing a picture will help children to write on any surface. Although this skill come under the physical development but help to language development

(c) Paying attention while listening to instruction and responding accordingly plays a crucial role in learning at an individual level.

Readiness of school for children: Readiness of school[15]

- Schools should welcome all children and accept the individual differences and different pace of learning.
- Schools should understand children develop holistically and at different rates.
- The content and delivery system should be aligned with their needs.
- Teachers should be aware and have the skill of teaching these children.
- School should provide specific time and duration for grade 1 children where they learn and practice their prerequisite skills.
- Schools may use various practices to bridge the cultural divide between home and school. The divide is greatest for children whose home language is not the same as the language of school instruction.

Readiness of family for children: Readiness of family[16]

This indicates the behavior, involvement and attitude of family and parents in their child's learning. Families & parents should

- Be aware of their role in a child's learning and development.
- Spend some time on a regular basis with their child.
- Provide encouraging, supportive, and rich environments for their children to learn comprehensively.
- Apart from taking care of and feeding their child, by telling a story, interacting with their child, and engaging them in simple educational activities, parents boost their child's learning in a holistic manner.

[14] Nipun Bharat 2021, page 167, under the paragraph 8.1.d
[15] Nipun Bharat 2021, page 167, under the paragraph 8.1.e
[16] Nipun Bharat 2021, page 167, para 8.1.f

3.3 Readiness Phase and Vidya Pravesh	
**Pre work**	Co-facilitator keeps the slides which are going to be shown ready.
**Objective**	Understand the objectives of the readiness phase and the Vidya Pravesh guidelines
**Total Duration**	20 minutes
	• Facilitators and participants brainstorm the objectives of the school readiness phase and discuss the similarity with Vidya Pravesh guidelines.<br>• Facilitator provides the school readiness manual in small groups and asks them to read the manual page and list down the<br>• Facilitator will conclude the session using the PPT slide
**Classroom arrangement**	Big Group
**Material Required**	-PPT slides 26-28

For Facilitator: School Readiness

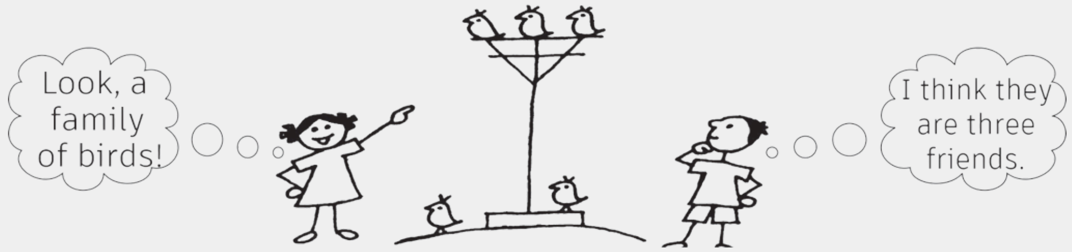

In schools, you will find 5-8 year old children either enrolled in grades 1-2 or about to be enrolled in the same. Often, we expect that by the end of grade 2, these children will be able to obtain basic language and math skills. Similarly, with grade 1, we expect some children to be competent enough to be able to learn reading and writing. Forming groups in class, interacting with other children, expressing themselves as well as listening to other children and teachers are some competencies that can be learnt at Anganwadis and even at home with someone's help before coming to school. This also includes learning to control small and large muscles. Schools, society and children's homes have a major role in improving the quality of education that children receive. Additionally, learning is closely linked with opportunity and guidance. If children receive ample opportunities and guidance, they can deeply enhance their learning capabilities. Therefore, if they receive additional help from someone at their homes, it can only help improve their learning.

This manual is created keeping this concept in mind. Apart from learning reading and mathematical skills, some additional activities have been provided which will help children in their physical, emotional and social development as well. Do these activities with the children so that they can prepare

for self-learning in a better way! Apart from these, include other activities which will help them in learning language and math. Seeing this as 'Warm-up Phase' (Official term) as a mere preparation would be a mistake. 'Self-learning' and 'learning in school' should not be seen as two different concepts. The 'Warm-up Phase' helps us realize that in learning, there is no need to force our experiences on them or to hurry with the process itself. We can also talk about 'what not to do' but for now, it is more important to be alert about 'what to do'. As a teacher, we should understand and accept that every child might have a different learning capacity and speed. Each child builds his knowledge in a different way. Therefore, we need to help each child, keeping these nuances in mind. Every child brings with him/her a certain kind of linguistic knowledge to class. Additionally, each child's knowledge can differ based on his/her experiences in life. We also need to help children understand that 'learning' essentially means enjoyment along with 'understanding meaning'. We hope that you will keep these pointers in mind while taking 2 hour classes every day.

For Facilitator: From the School Readiness Manual (Preface)

The National Education Policy 2022 focuses on basic literacy and mathematical skills of children. Emphasis on strengthening and strengthening, including pre-primary and the initial 3 years of primary education are described, highlighting that early years of children should be focused on the field of education and children should learn to read and write. As we all know that the last 2 years due to the COVID-19 pandemic, schools were closed. Due to which all the children who took admission in class 1 and 2 this year have been deprived of school experiences. Therefore, when schools reopen this year, we will need to reemphasize interventions around enrollment, interest in school along with strengthening foundational skills apart from attendance as these might just be some of the core challenges schools will face on reopening. So this year, we should work with grades 1 and 2 in a step-by-step manner. There are 3 steps that we can follow - the first stage is school readiness, followed by the second stage in which we will focus on foundational literacy and mathematical skills. Lastly, a strong initial understanding and use of textbooks will be a part of the third phase. Emphasis will be on acquiring mastery in skills at each level. A readiness package is prepared considering the all-round development and school readiness of children. During the initial days of class operations this year, along with grade 1, the Government of Himachal Pradesh has prepared a play-based school readiness package for all children from grade 2 to grade 5. This package covers learning needs of children and experiences of pre-primary schools. In addition, this school readiness package will also help teachers provide a fear-free and joyful environment for children to attend school. Keeping above mentioned points in mind, with detailed fun activities related to school readiness of children, tips on how teachers, parents and community help children together are also suggested. We hope you will conduct school readiness activities with children according to the given instructional manual.

Day 1: Session 4
**Phase I implementation: School Readiness**

The fourth session of the first day is divided into three main sections. The total duration of this session is 1 hour 45 minutes. This session will cover the topics mentioned below:

4.1 School Readiness: Week-wise planner and classroom transaction
4.2 Hands-on practice for classroom process of school readiness
4.3 Concluding the School Readiness phase.

4.1 School Readiness: Week-wise planner and classroom transaction	
**Objective**	Understand how to conduct a school readiness class with children
**Total Duration**	30 minutes
**Process of Facilitation**	• The facilitator will inform the participants that they're going to demonstrate 1 full day readiness class.<br>• The facilitator will demonstrate the 1 full day lesson plan from Week 1 planner of school readiness.<br>• Discussion on what the participants observed during the demonstration. *(Exemplar participant response- participants should be able to tell the activities that were done, the sequence in which they were done, and the domain to which the particular activity belongs)*
**Classroom arrangement**	Big group
**Material Required**	• Material provided in the trainer kit.<br>• PPT slide 29

Note:
During the demonstration, the facilitator will act like a teacher while considering the participants as children in their class.

4.2 Hands-on practice for classroom process of school readiness	
**Objective**	Provide hands-on practice to the participants for school readiness activities
**Total Duration**	60 minutes

**Process of Facilitation**	**Small groups**<br>● Facilitator to provide school readiness manual in the small groups and ask them to read specific pages (week wise allocation as described in the PPT)<br>● Participants to practice the activities in small group according to the assigned week (20 minutes for small group practice and preparation)<br><br>**Big group**<br>● Selected 3 groups will conduct the demo class and will be given quick feedback one by one.
**Classroom arrangement**	Small groups, big group
**Material Required**	● PPT slide 30<br>● Specific pages from the school readiness manual<br>● Play kit and classroom teaching material

Week 1_Planner

Area of Domain	Activity	Day 1	Day 2	Day 3	Day 4	Day 5
Socio-emotional Development	Free Play 10 Minutes	Children to do free play activity in the beginning of class.				
	Aao Bole (Let's Speak) 10 Minutes	To talk to children about their- name, age, etc	To talk to children about their- name, age, etc	To talk to children about their family-number of members, who does what etc	To talk to children about their school-things they like etc	To talk to children about their friends-number of friends, things they like about them etc
Language Development	Sune Sunayein (Story telling and retelling) 20 Minutes	"To work on a story for two days. Storytelling by teacher and retelling by children on first day. Storytelling by teacher and retelling by children followed by asking simple questions and role play by children. Note: At least 30 days story should be done following the above mentioned process. Later one reading with putting finger on each word by teacher and children both must be introduced."				
	Sune Pehchane (Listen and identity) 10 Minutes	Distinguish between sounds of bangles, pebbles/stone	Distinguish between sounds of bangles, anklet, pebbles/stone	Distinguish between sounds of different animals	Distinguish between sounds of different vehicles	Distinguish between sounds- laughing, angry, crying, shouting
	Akshar Khel (Letter game) 10 Minutes	Ek aneк- to tell the words starting with letter 'म' letter like - machli, matar	Ek aneк- to tell the words starting with letter 'क' letter like - kamal, kachua	Patta palat game- with letters 'म' and 'क'	Letter jump (akshar kood) game with letters- 'क' 'म' 'ल' 'ब' 'र'	Letter jump (akshar kood) game with letters- 'क' 'म' 'ल' 'ब' 'र' 'ह'
	Aao Bnanayein (Let's make) 10 Minutes	To colour the picture from book	Children to draw picture of their choice and tell about the drawn picture	To draw anything from book/story books and tell what did they draw and why	Draw anything and colour	Draw anything and write about it
	Aao gayein (Let's sing) 10 Minutes	Ek mota hathi- in big group	Ek mota hathi- individually by few children	Billi mausi- in big group	Billi mausi- individually by few children	Children to recite their favourite story turnwise
Cognitive Development	Aao Soche (Let's think) 10 Minutes	Show circle shape to children, ask them to identity objects of same shape from the surroundind and count them	Show circle shape and make pattern: small-big	Show rectangle shape to children, ask them to identify objects of same shape from the surrounding and count them	Put circle and rectangle shapes in order from big to small	To find similarity and differences in the shapes- circle, rectangle
		Classification from the objects around- seeds, leaves, twigs etc	Oral pattern- 1 flower 2 leaves, 1 flower 2 leaves, 1 flower 2 leaves	What's inside the bag? Touch and identify them	Oral pattern- 2 pencil 3 eraser, 2 pencil 3 eraser, 2 pencil 3 eraser	Classification on the basis of shape, colour
	Aao Gine (Let's count) 10 Minutes	Number chart activity (only 1-9)<br>• Teachers to read 1 line from the chart<br>• Teacher to ask numbers randomly<br>• Teacher and children will read together (putting finger on each number)		• Ask 2-3 children to read the same line<br>• Teacher to say any number and children to find in there number card and write		
		• Activity on estimation and approximation in number (only 1-9) for example 'snap and clap'				
	Aao khlein (Let's play) 10 Minutes	Jumping on numbers	Making circles and count	Counting tilli and writing the number	Jumping on numbers	Making circles and count
	Aao hal karien 10 Minutes	To do oral word probem of simple addition and subtraction upto 1 digits				
Physical development	Khel 10 Minutes	Walking on straight line	Jumping inside and outside circle	Paper folding- making fan	Walking on zig-zac line	Making cicle with clay/sand

Week 2_Planner

Area of Domain	Activity	Day 1	Day 2	Day 3	Day 4	Day 5
Socio-emotional Development	Free Play 10 Minutes	Children to do free play activity in the beginning of class.				
	Aao Bole (Let's Speak) 10 Minutes	To talk to children about their favourite game	To talk to children about the process of playing their favourite game	On situation- how do they feel when...	To talk on picture	On situation- if I (happy, sad, angry)
Language Development	Sune Sunayein (Story telling and retelling) 20 Minutes	To step wise work on a story for five days. Discussion on title, story reading by teacher, discussion on question, story reading by children, finding-writing-reading words.				
	Sune Pehchane (Listen and identity) 10 Minutes	Distinguish between sounds of different things	Distinguish between sounds of animals	To tell number of sounds in a word (2 unit)	To tell number of sounds in a word (2 unit)	To tell number of sounds in a word (3 unit)
	Akshar Khel (Letter game) 10 Minutes	To make the letter same as I make	Ek anek- to tell the words starting with letter 'ख' letter like - khargosh, khat	Find the letter game with taught letters	I'm saying, you write the words	Making words out of letters
	Aao Banayein (Let's make) 10 Minutes	Children to draw picture of their choice and tell about the drawn picture	Draw from the story read	I'll say and you draw	To draw on a worksheet	Draw anything of their choice and write about it
	Aao gayein (Let's sing) 10 Minutes	Bandar ped par baitha hai- in big group	Ek mota hathi- individually by few children	Main to so rhi thi- in big group	Main to so rhi thi- individually by few children	Children to recite their favourite story turnwise
Cognitive Development	Aao Soche (Let's think) 10 Minutes	Classification of circle and triangle	Sequencing of circle and trianlge shape (biggest, bigger, big, small....)	Pattern from - circle and triangle	Show rectangle shape to children, ask them to identity objects of same shape from the surrounding and count them	To find similarity and differences in the shapes- circle, rectangle, rectangle
		Comparison of 2-3 shapes based on the size	Classification of fruits and vegetables	Pattern from objects- pencils, rubber...	Comparison- light and heavy	Classification of objects on the basis of color/size
	Aao Gine (Let's count) 10 Minutes	Number chart activity (only 1-9)<br>• Teachers to read 1 line from the chart<br>• Teacher to ask numbers randomly<br>• Teacher and children will read together (putting finger on each number)<br>• Ask 2-3 children to read the same line<br>• Teacher to say any number and children to find in there number card and write				
		• Activity on estimation and approximation in number (only 1-9) for example 'snap and clap'				
	Aao khlein (Let's play) 10 Minutes	Sequencing of numbers with number cards	To play true and false activity with numbers	To play true and false activity with addition and subtraction of numbers	Making circles equivalent to the number told	To find and write the asked number
	Aao hal karien 10 Minutes	To do oral word problem of simple addition and subtraction up to 2 digits				
Physical development	Khel 10 Minutes	Playing with elastic rope	Paper folding- ship making	Frog jump	To make circles out of paper	Tipi-tipi-tap

Week 3_Planner

Area of Domain	Activity	Day 1	Day 2	Day 3	Day 4	Day 5
Socio-emotional Development	Free Play<br>10 Minutes	Children to do free play activity in the beginning of class.				
	Aao Bole (Let's Speak)<br>10 Minutes	To talk to children on the objects- like about toys; how do they look like, where do you them from...	To talk to children on food item	To talk to children on festival	On situation- if suddenly there is short circuit and electricity is gone	On situation- what you would have done if...
Language Development	Sune Sunayein (Story telling and retelling)<br>20 Minutes	To step wise work on a story for five days. Discussion on title, story reading by teacher, discussion on question, story reading by children, finding-writing-reading words.				
	Sune Pehchane (Listen and identity)<br>10 Minutes	To tell number of sounds in a word (3 unit)- katori, mausam	To tell number of sounds in a word (2 unit)	To tell number of sounds in a word (2 unit)	To tell number of sounds in a word (2 unit)	To tell number of sounds in a word (3 unit)
	Akshar Khel (Letter game)<br>10 Minutes	To make words of same sounding words (milte-julte shabd)	To write word on any said letter	Making words out of letters	Letter jumping games	Patta palat game
	Aao Bnanayein (Let's make)<br>10 Minutes	Draw from the story read	To write letter by copying	Listen and write letter	Listen and write	Draw anything of their choice and write about it
	Aao gayein (Let's sing)<br>10 Minutes	Aaloo kachalu- in big group	Aaloo kachalu- individually by few children	Twinkle twinkle- in big group	Twinkle twinkle- individually by few children	Children to recite their favourite story turnwise
Cognitive Development	Aao Soche (Let's think)<br>10 Minutes	Show square shape to children, ask them to identity objects of same shape from the surrounding and count them	To show square shape and ask the children about its name. To do sequencing activity with the square shape	Pattern from - circle and triangle	Show rectangle shape to children, ask them to identity objects of same shape from the surroundind and count them	To find similarity and differences in the shapes- circle, rectangle, triangle and square
		Classification of circle, rectangle, triangle on the basis of colours, corners, sides and other characteristics	Classification of birds and animals	Pattern from objects- flower, leaves...	To tell: Up-down, Right-Left	To tell: Long-short
	Aao Gine (Let's count)<br>10 Minutes	Number chart activity (only 1-9)<br>• Teachers to read 1 line from the chart<br>• Teacher to ask numbers randomly<br>• Teacher and children will read together (putting finger on each number)		• Ask 2-3 children to read the same line<br>• Teacher to say any number and children to find in there number card and write		
		• Activity on estimation and approximation in number (only 1-9) for example 'snap and clap'				
	Aao khlein (Let's play)<br>10 Minutes	Kitne bhai kitne	To make bundle with tilli	Stapu	Kitne bhai kitne	To make bundle with tilli
	Aao hal karien<br>10 Minutes	To do oral word problem of simple addition and subtraction up to 2 digits				
Physical development	Khel<br>10 Minutes	To walk while keeping book on head	Nishanebaaz- target activity	To decorate wall and floor	Posham pa bhai posham pa	Paper folding- dog making

Week 4_Planner

Area of Domain	Activity	Day 1	Day 2	Day 3	Day 4	Day 5
Socio-emotional Development	Free Play 10 Minutes	Children to do free play activity in the beginning of class.				
	Aao Bole (Let's Speak) 10 Minutes	To talk on a picture-description about the picture	On situation- if anyone ask you for your favourite toy	To talk on the object- its physical characteristics, composition	On situation- how will you feel if someone will scold	Children to talk about themselve
Language Development	Sune Sunayein (Story telling and retelling) 20 Minutes	To step wise work on a story for five days. Discussion on title, story reading by teacher, discussion on question, story reading by children, finding-writing-reading words.				
	Sune Pehchane (Listen and identity) 10 Minutes	To tell number of sounds in a word (3 unit)- katori, mausam	To tell number of sounds in a word (2 unit)	To tell number of sounds in a word (2 unit)	To tell number of sounds in a word (2 unit)	To tell number of sounds in a word (3 unit)
	Akshar Khel (Letter game) 10 Minutes	To write said letter	To say words starting from the said letters	Making words out of letters	To play vocabulary building activities	Word game- singular/plural
	Aao Bnanayein (Let's make) 10 Minutes	Draw from the story read	To write anything from their choice	Draw and write about the read story	I'll say, you draw and write about it	Draw anything of their choice and write about it
	Aao gayein (Let's sing) 10 Minutes	Ek thi raja ki beti- in big group	Ek thi raja ki beti- individually by few children	Jonny,Jonny Yes, Papa! - in big group	Jonny,Jonny Yes, Papa!- individually by few children	Children to recite their favourite story turnwise
Cognitive Development	Aao Soche (Let's think) 10 Minutes	To make figure out of shapes	Pattern from circle, triangle and square (2-3 patterns)	Sequencing of circle, triangle and square (Big, small, smaller, smallest)	To make interesting figure out of shapes	To find similarity and differences in the shapes- circle, rectangle, triangle and square
		Sorting of the objects present nearby- seeds, leaves, branches...	Classification of land and water transport	Pattern from the objects- flowers, leaves etc.	Game: Left-right feet	Game: Near-Far object
	Aao Gine (Let's count) 10 Minutes	Number chart activity (only 1-9)<br>• Teachers to read 1 line from the chart<br>• Teacher to ask numbers randomly<br>• Teacher and children will read together (putting finger on each number)		• Ask 2-3 children to read the same line<br>• Teacher to say any number and children to find in there number card and write		
		• Activity on estimation and approximation in number (only 1-9) for example 'snap and clap'				
	Aao khlein (Let's play) 10 Minutes	Stapu game	Jump on number	To make bundle out of tilli	Count the tilli and write the numbers	Kitne bhai kitne
	Aao hal karien 10 Minutes	To do oral word probem of simple addition and subtraction upto 2 digits				
Physical development	Khel 10 Minutes	Free Dance	To make necklace with beads	To draw and colour it	Animals walk	To make toys out of clay

4.3 Concluding the School Readiness Phase	
**Objective**	Conclude the readiness phase sessions
**Total Duration**	10-15 minutes
**Process of Facilitation**	• Facilitator will conclude the school readiness phase by asking the following questions:<br>○ How long does the school readiness phase last?<br>○ How many hours to give for school readiness activities every day?<br>○ Which developmental domains get covered in the activities?<br>○ What do we do on the 6th day?
**Classroom arrangement**	Big group
**Material Required**	PPT slide 31

Assignment	
Reading assignment on Free Play and Maths	
**Material Required**	PPT slide 33

Day 1: Reflection	
**Total Duration**	30 mins
**Process of Facilitation**	• Facilitator will ask "What is one new thing that you learnt today?"<br>• Each participant will share one new thing that they learnt today.
**Classroom arrangement**	Big group
**Material Required**	PPT slide 34

DAY 2

TEACHING MATH IN GRADES 1-2

Day 2: Session 0
**Free play: Four Learning Corners**

Before the day's session starts, all participants will be encouraged to engage with the four learning corners.

**Objective**	Understand how to conduct free play in practice class
**Total Duration**	10 minutes
**Process of Facilitation**	● The facilitator & co-facilitator will create the four corners for free play in the training hall.<br>● The facilitator will treat the group as children.<br>● Before entering the room, the facilitator will ask each participant to play at the corner of their choice.<br>● Each participant will be asked to put their bags in one place and play at the four learning corners.<br>● After 10 mins, ask the participants to keep all the material used back at their places neatly.
**Classroom arrangement**	Small groups
**Material Required**	Appropriate material for four different corners (handmade, printed etc.)

Day 2: Session 1
**Attendance, Prayer and Recap**

The first session of the second day is divided into two main sections. The total duration of this session is 30 minutes. This session will cover the topics mentioned below:
1.1 Attendance and Prayer
1.2 Recap

1.1 Attendance and Prayer	
**Pre work**	Keep the attendance sheet ready.
**Objective**	Mark the attendance and sing a prayer before starting the day
**Total Duration**	10 minutes
**Process of Facilitation**	● The facilitator will provide the attendance sheet and ask participants to sign on the attendance sheet.<br>● The group assigned will conduct the prayer.
**Classroom arrangement**	Big group
**Material Required**	Attendance sheet, PPT slide 37

1.2 Recap	
**Objective**	Recall the topics covered on the previous day
**Total Duration**	10 minutes
**Process of Facilitation**	● The group assigned to lead the recap session will lead it.<br>● Participants will sit in a big circle and each participant from the assigned group will share their learnings from the previous day.<br>● The facilitator will make sure that every broad topic discussed on the previous day should get covered.<br>● The group assigned with leading the rules session will reiterate the rules.
**Classroom arrangement**	Big group
**Material Required**	PPT slide 38

Day 2: Session 2

Free Play

The second session of the second day will include a discussion on the four corners of free play. The total duration of this session is 30 minutes.

**Objective**	Understand the benefits of free play for achieving developmental goals
**Total Duration**	30 minutes
**Process of Facilitation**	**Big group**<br>● Discussion on the steps of doing free play and the things to remember.<br>○ Discussion about what was kept at each learning corner and why we need to keep those things there.<br>○ Discussion about the kind of materials they can use to make the materials if they don't have it.<br>○ Discussion about the welcome activity (chart) as well as the attendance process (putting their names in a bowl)<br>● The facilitator will show the UNICEF video on free play.
**Classroom arrangement**	Big group, small groups
**Material Required**	● Material to set up the learning corners.<br>● UNICEF module 10: Video 4<br>● Pages on free play from the school readiness manual (Pages 8 and 9)<br>● PPT slides 39 and 40

For Facilitator: Free play (Beginning of class)

Before coming to school, it is necessary to prepare children for school. But this preparation does not mean that they should be taught to read and write, but by this we mean to inculcate those habits, behaviors and those needs which should be worked upon in the Anganwadi center or pre-primary schools at home. Like listening to others, taking responsibility for one's own things, holding a pencil properly, playing in a group, being happy etc. But by being a teacher, it should also be understood that continuous work should be done on all these habits, behavior and abilities.

In other words, in Class 1, along with language and math skills, physical and socio and emotional skills development should also be worked on because this has a direct effect on their learning of reading, writing and mathematical skills. Sometimes different activities have to be done for their fine/gross motor skills and socio-emotional development in children. These activities have a great impact on developing these skills.

Therefore, some activities should be done daily in class 1 in such a way that children get opportunities to work in groups or individually.

Socio-Emotional Development Activities

Socio- emotional development also contributes to the process of learning to read and write for children of classes 1 and 2, therefore appropriate activities for socio-emotional development should be done with the children of classes 1 and 2 that are taking admission in the school.

Children should be given enough opportunities to play games and activities related to socio-emotional development in groups and pairs. Such as playing in groups, poetry, songs, clay/sand games, conversation, dancing and playing with dolls etc.

Physical Development

Before starting class 1, children should have a strong development of small muscles i.e., hand eye coordination and ability to balance on large muscles of their body. Sometimes children do not get enough opportunities/experience to work on these abilities before they come to Class 1, which is why children in class 1 and 2 are taught to run and jump for strong development and coordination on small and large muscles. It is necessary to do games and activities like paper tearing and coloring etc.

What And How to Do

Give opportunities to the children to have free play time before the daily language and math lessons to develop the above-mentioned abilities. Daily free play time should be done in class for 10 to 15 minutes in which children should be given the opportunity to play their favorite games in groups, pairs and individually.

- Make four corners in the class. Keep different types of material in each corner and let the children play in the corner of their own choice.
- Similarly, for physical (large muscles) and creative development, encourage them to walk around the school grounds and express themselves by observing different things.

Note: For making "Free play corner" in the class, what kind of activities can be done by them? The details of the activities related to physical development and school tour are also given in the booklet. Do some activities every day in the class with the children.

Keep in Mind

- Avoid telling children what they should play with.
- Children can play individually initially but encourage them to play with others and in groups.
- During this time, you can roam around and see if anyone needs your help.
- After a few days, let the children sit in different corners and play.

Day 2: Session 3

Teaching Mathematics in Grades 1 and 2

After the children complete the school readiness phase, we will transition into Phase II. In Phase II, we will start with teaching Math's to achieve the FLN goals by the end of Grade 2.

The third session of the second day is divided into two main sections. The total duration of this session is 50 minutes. This session will cover the topics mentioned below:

3.1 What is teaching Mathematics in the early grades, and what are the key components of Mathematics?
3.2 Framework of the Math classroom process

**3.1 What is Mathematics, and what are the key components of Mathematics?**	
**Objective**	Understand what is teaching Math's in the early grades and what are the key components of Math's
**Total Duration**	30 minutes
**Process of Facilitation**	• Co-facilitator divides the whiteboard into two columns. Do not name any column.<br>• Facilitator asks, 'What is teaching Math's in the early grades and why is it important?' (Take 6-7 responses)<br>• Every response that is a characteristic of 'What is teaching Math's in the early grades' should be written in the first column.<br>• The facilitator asks, 'What are the key components of Math's?'; Take a few responses till you complete all the components of Math's.<br>• Co-facilitator lists all the components of Math's on the board.
**Classroom arrangement**	Big group
**Material Required**	PPT slides 41 and 42

**3.2 Framework of the Math classroom process**	
**Objective**	Understand the classroom process to be followed for Math's
**Total Duration**	20 minutes
**Process of Facilitation**	• Facilitator to show the PPT slide for math framework and discuss in detail.<br>• Facilitator to tell the manual page number on which framework of daily class process is given.<br>• Give a brief example of an activity for each step in the process using the Phase II manual.
**Classroom arrangement**	Big group
**Material Required**	PPT slide 43, page from the Phase II manual titled 'Daily Classroom Plan'

For Facilitator: Teaching Math’s in the Early Grades
In terms of learning, it is often said that interest in a particular task affects the learning process. When we talk about interest in Math, it is often observed that many students do not show interest in Math, but Math is one subject that attracts students towards itself very easily. Students start to understand and apply various concepts of Math in their everyday life with the help of things present in their immediate surroundings even before they start coming to school. Even though they are not capable of counting things yet, they can still manage to estimate their sizes, shapes, distance, thickness, etc. on their own. Additionally, students are also capable of understanding different patterns present in things around them. Even though they cannot understand the mathematical concepts behind such patterns, they still enjoy it. If students' experiences are taken up in class as a means to introduce mathematical concepts to them, the process of learning will become more enjoyable and easier for them. Sometimes, students can be deprived of such experiences and in those cases; it is our job to help them in an effective way.<br><br>**Things to remember in order to teach students.**<br>● Before making the students read numbers, it is important to make them learn the foundational skills like counting things, their order, and cardinality. This will help the students to understand the symbols of numbers and the relationship between the numbers and their base tent.<br>● Encourage the students to present their work after working with their group members to build their confidence and to help them decide what is wrong and what is right.<br>● In order to make children learn mathematical concepts easily, it is important to teach these concepts by organizing various activities that will help the students to learn these concepts in an enjoyable way.<br>● Normally, students start grasping mathematical concepts by observing the things around them. Concepts like measuring quantities, finding patterns, understanding balance and positions and sharing things equally with their siblings come naturally to them without any special instructions.

Day 2: Session 4
**Pre-math**

The fourth session of the second day is divided into two main sections. The total duration of this session is 1 hour. This session will cover the topics mentioned below:

4.1 Pre-math: Understanding of the components of pre-math.
4.2 Pre-math: Demo

4.1 Pre-math: Understanding the components of pre-math	
**Objective**	Understand the components of pre-math
**Total Duration**	20 minutes
**Process of Facilitation**	● Facilitator to ask, “What do we understand by pre-math?” (Take 2-3 responses). Co-facilitator to write all the responses on the whiteboard.<br>● Facilitator to ask, “Which components are covered under Pre-Math?”

	(Exemplar participant response- Shapes, Patterns, Directions, Classification, Comparison)<br>● Briefly explain each component using the PPT slides and give an example for each without taking more than 2 mins to explain each component.
**Classroom arrangement**	Big group
**Material Required**	PPT slides 44 and 45, pages from Phase II & III manual of Mathematics titled 'Pre-math related activities'

**4.2 Pre-math: Demo**	
**Pre work**	Printouts of the activity booklet
**Objective**	Understand the activities to conduct for pre-math development
**Total Duration**	40 minutes
**Process of Facilitation**	● Facilitator to conduct the quick demo on each component (Shapes, Patterns, Directions, Classification, Comparison) of Pre-Math.<br><br>*Note: Each activity should be done from a simple to complex manner.*<br><br>● Distribute the activity booklet in the small groups.<br>● Inform the participants that they need to do a demo of each component of pre-math using the activities given in the booklet. One group would be selected in the end to present the demo for all the components of pre-Math in the big group.<br>● Let the participants prepare for 20 mins.<br>● Feedback by facilitator and co-facilitator in small groups.<br><br>**Big group**<br>● Demo of each component by one group in front of the big group<br>● Feedback on the demo
**Classroom arrangement**	Big group
**Material Required**	PPT slide 46, Pages from Math Activity booklet

For Facilitator: From the Math Activity Booklet

Forward the Pattern (With Objects)

Classroom Arrangement: In a big group & small group

Material: Some solid objects.

Process: With solid objects.

- Take some similar types of items, like utensils, bowls & spoon etc (quantity of each item should be 3-4 pieces)
- Now arrange these items in any patterns, like- 1 bowl- 1 spoon,1 bowl-1 spoon,1 bowl… (Apply the pattern while speaking to the children and then stop).
- Now ask any child to complete the pattern.
- Encourage each child to make a pattern with whatever they have.
- Make the initial patterns simple & use arrangement by using only two items.

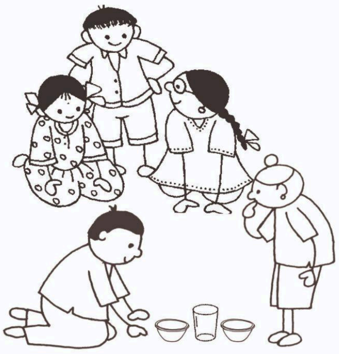

Classification

Classroom Arrangement: In a big group

Material: Copy, pencil

Process: With solid object

- Sit in a small group.
- Give each group some mixed things, such as gram, chickpeas, gram and small seeds, etc.
- Now ask children to sort the given items and separate them.
- Talk to children, which item needs to be kept separately and why?
- Do this activity with a picture flash card along with a solid object.
- Ask them to classify the shapes on the basis of smaller, bigger and equal sides.
- Use English vocabulary/sentences wherever possible, like side, square etc.

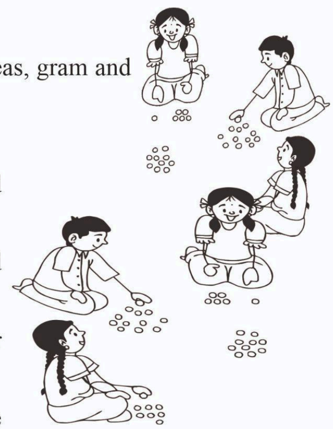

What did I make

Classroom Arrangement: Big group

Material: Nothing

Process: Oral and Written

- Ask them to sit in a large circle.
- Ask the children to draw any shape they want in the air and the rest of the children identify it.
- Ask all the children what shape they make?
- Similarly, ask the children to draw and identify shapes in the air.
- This activity can also be done in pairs, with one child drawing the shape on the back of the other.
- If possible, use as much English vocabulary for yourself and the child as well, such as This will be a 'square'.

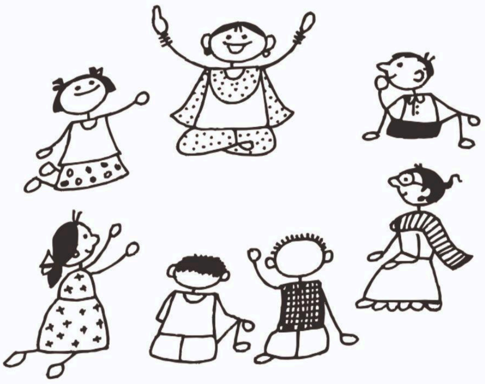

Distribute equally

Classroom Arrangement: Big group/individual

Material: Copy, pencil/chalk

Process: Written

- Everyone sits in a circle.
- Tell the children, the shape which I will say, you will draw it on your copy/ground.
- Then ask children to make the shape into two parts/pieces from its middle.
- Continue the game with other shapes.
- Wherever possible, yourself and children must use English vocabulary/sentences, such as- divide 'square' into 'two' parts.

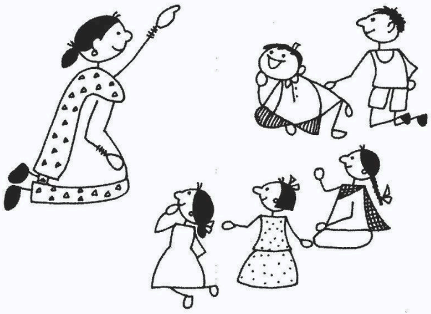

For Facilitator: Pre-math
When we look at students aged 5-8 who are developing their pre-math abilities and are moving towards recognizing numbers, we assume how easily do these students combine their understanding of Math with their life and classroom situations. As a teacher, one should think why students in upper primary classes are scared of Math. This might happen because of the lack of balance between understanding of mathematical concepts and their practice.<br>There are two important things to be considered while teaching Math in grades 1 and 2:<br>● There should be proper balance between pre-mathematical abilities and the number recognition skills.<br>● The process of teaching these concepts should be made enjoyable to enhance students. learning.

Day 2: Session 5
Number Sense

The fifth session of the second day is divided into two main sections. The total duration of this session is 1 hour 10 minutes. This session will cover the topics mentioned below:

5.1 Number Sense: Katta Pullalu
5.2 Number Sense: Number Chart Reading

5.1 Number Sense: Katta Pullalu	
**Pre work**	Keep tillis and a few bundles ready. Keep the printouts of the number chart and the number cards ready.
**Objective**	Understand and conduct Katta Pullalu activity for number recognition
**Total Duration**	40 minutes
**Process of Facilitation**	**Big group**<br>● The facilitator will show a certain number of tillis, and each participant will guess the number of tillis that the facilitator is holding in their hand.<br>● Now, let each participant take a few tillis.<br>● Then, the facilitator and participants will count the number of sticks they have.<br>● Count forward and then backward.<br>● Use this activity to introduce 0.<br>● The facilitator will do a quick recap of the steps.<br>● Next, ask them to find that number on the number chart as well and write it in their notebooks.<br>*Note:*

	Introduce bundling following the given below process only when children are comfortable with numbers up to 9.<br><br>**Big group**<br>● Next, explain the concept of the Katta Pullalu house (Katta Pullalu house).<br>● Count with the participants using the same in the big group.<br><br>**Small groups**<br>● Ask the participants to practice in small groups.<br>● Facilitator and co-facilitator will give feedback in small groups.
**Classroom arrangement**	Big group, small groups, individually, number chart for participants
**Material Required**	PPT slide 47, tillis, rubber bands, number cards

**5.2 Number Sense: Number Chart Reading**	
**Pre work**	Keep the printouts of the number chart and the number cards ready.
**Objective**	Understand and conduct activities for number recognition
**Total Duration**	30 minutes
**Process of Facilitation**	**Big group**<br>● Use the number chart to count to 10 (use different strategies to count, recognize numbers, etc.)<br>● Then ask the participants to count with you.<br>● Ask all the participants to find a number in their charts and write it in their notebooks. The facilitator would roam around the room to help whichever participant needs help.<br><br>**Small groups**<br>● Ask the participants to practice in small groups.<br>● Facilitator and co-facilitator will give feedback in small groups.
**Classroom arrangement**	Big group, small groups
**Material Required**	PPT slide 48, number chart, number cards

For Facilitator: Number Sense
Students begin to understand the quantity of things very early on, but they are. unaware about its correct quantity and the process of measuring it. Students should be. encouraged to count in the beginning and they should also be explained this process which. will help them to understand the relationship between numbers and the patterns of these numbers. It is important to build foundational skills from the beginning.<br><br>**[17]Numbers and Number Operations:**<br>● Cardinal numbers are used to measure and communicate the size of a group of objects.<br>● Ordinal numbers are used to describe the position of an object when they are arranged in a specific order.<br>● Nominal numbers are used as nouns/labels only, that is to identify or label objects in a group or individually; they do not have an actual value or position.<br>● One-to-one correspondence: involves matching or pairing of objects.

Day 2: Session 6
**Level-wise Activity and Word Problems**

In order to strengthen the understanding of numbers, organize number-related games in the class every day. (During this period, those students who have acquired the understanding of the numbers, give them the number card). The sixth session of the second day is divided into two main sections. The total duration of this session is 1 hour 20 minutes. This session will cover the topics mentioned below:

6.1 Level-wise Activity
6.2 Word Problems

6.1 Level-wise Activity	
**Objective**	Understand and conduct activities for number recognition
**Total Duration**	40 minutes
**Process of Facilitation**	**Big group**<br>● Facilitator to ask, "What are the different Math's levels of children in the classroom?"<br>● Discussion on different levels in mathematics and appropriate tasks for each level group (Beginner and 0-9, 10-50, 51-99).<br>● Co-facilitator lists all the tasks discussed on board.<br><br>**Small groups**<br>● Ask the participants to look at the level-wise activities from the booklet in their smaller groups and practice 1-2 activities for each level.<br>● Facilitator and co-facilitator will give feedback in small groups.

[17] Number and Number Operation: NIPUN, glossary

**Classroom arrangement**	Big group, small groups
**Material Required**	PPT slide 49, Math Activity booklet

For Facilitator: From the Math Activity Booklet

Classroom Arrangement: In a big group

Material: Nothing

Process: Written

- All the children stand in a circle and you stand in the middle.Tell children we will clap and sing a song. I will say how many brothers, how many and while roaming in the circle - you will say as many as you want.
- When you say a number, such as 3, all the children will form groups of 3. Children whose group is more or less than 3 should sit down.
- Continue playing like this.
- Keep in mind that while forming small groups, the children should not be drawn with each other.
- Where possible, children and themselves should use English vocabulary/sentences, such as- If I say 'three', then you make a 'group' of 'three'.

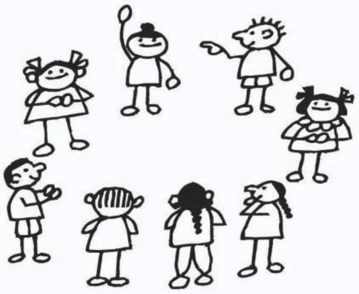

I will say and you will Count

Classroom Arrangement: Individual

Material: Stick/ pieces of chalk/pebble

Process: With solid Objects

- Tell children that we will play a game.
- Give sticks/pebbles/pieces of chalk to all the children.
- I will say a number, you count the same number of sticks/pebbles/chalk pieces.
- Say a number, ex-6, children will count 6 sticks and tell.
- After a few days children can also be asked to write numbers/digits.
- Use English vocabulary/sentences yourself and the child where possible, such as- count to 'five'.

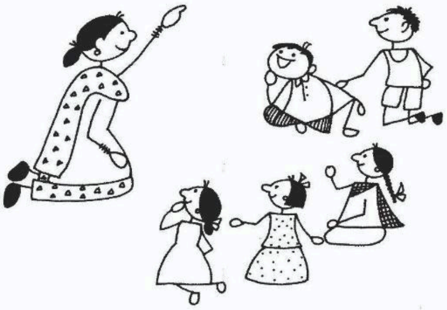

How many bundles and how many sticks

Classroom Arrangement: Individual

Material: Nothing

Process: Oral

- Give sticks to children.
- Children build a house of bundles and sticks.
- Count bundles and sticks and find from the number card.
- Ask children to say the number in bundles and sticks, example- 25, children will say two bundles and five sticks.
- Make sure the children change sticks in between.
- Be sure to keep sticks in your hand as well.

Tens	Ones
Bunch	Stick
2	5

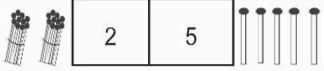

6.2 Word Problems	
**Objective**	Understand how to solve word problems related to addition and subtraction
**Total Duration**	40 minutes
**Process of Facilitation**	**Big group**<br>● Broadly discuss the approach to solving word problems in class. Understanding what is happening in the problem is the first step before understanding how to do the Math involved in the problem.<br>**Steps**<br>● Narrate a word problem like a story (using only 1-2 sentences).<br>● Ask the participants "What is being asked in the problem?"<br>● Now ask the following questions: "What do you think needs to be done? Are things getting more or less?"<br>● Once the participants answer, ask them: "How did you arrive at this answer?"<br>● Now, the facilitator and the participants will solve the problem using the tilis on the floor. The facilitator uses the set of questions asked above to arrive at the answer.<br>● Similarly, discuss more such problems.<br><br>***Note:*** *Solve at least one word problem in the class every day.*

	**Small groups**<br>● Let the participants practice using the activity booklet in small groups.<br>● Facilitator and co-facilitator will give feedback in small groups.
**Classroom arrangement**	Big group
**Material Required**	PPT slide 50, Math Activity booklet

For Facilitator: From the Math Activity Booklet

Cross the Line

Classroom Arrangement: In big/small groups

Material: Nothing

Process: Oral

- Draw a straight line on the floor. Make the children stand on one side of the line facing in the same direction.
- Now tell the children that you are going to say something, if they agree to it, they are going to cross the line, if they do not agree with it, then they must stand at the line itself.
- Now, you have to say 'cross this line if you feel that adding one and one makes it three?'

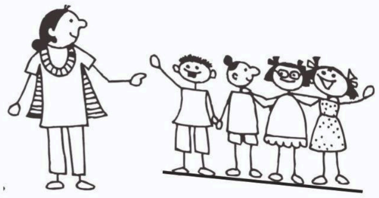

- It's on the children now to decide whether they have to cross the line or not.The child who commits a mistake shall sit (who crosses the line when the children were not supposed to cross the line or does not cross the line when they were meant to cross the line).
- Play this game unless there is one child left at the end.

- You can play this game with different shapes/patterns/classifications too.
- Wherever possible try to use English vocabulary/sentences yourself and ask students to use them too.

For Facilitator: Word Problems

The objective of word problems is to develop in students an understanding of the process of solving questions as well as situational problems in students. Discuss the process of solving a word problem with the students. Ask the students to think and verify the answer. Understand the question with the help of the given picture. Then ask the students to solve the question formally in their notebooks. The teacher should also discuss the process of solving the question with the students. After solving the problem, verify the answers.
Some things to keep in while discussing word problems:

- Students are able to solve questions based on addition and subtraction orally but have difficulty solving the same in a formal manner because they are not familiar with the process. Ensure that the students

understand the process to solve a question instead of memorizing it. It is important for the students to understand and solve a question orally in different ways.

- After the students start to understand the questions, give more questions to them, organize discussions and ask the students to make frames and solve the questions using bundles and straws. Help the students if they face any difficulty.
- Discuss the different ways to solve a problem.
- Tell the students that if we add the numbers at ten places, we should be able to guess.
  the correct total from just that. For example, if we have to add the numbers '36' and '25', we first add the digits at the tens place in the given numbers such as 3+2=5. Then, we can guess that the total should be a number that will be greater than 50.

Day 2: Session 7
**Measurement and Conclusion of the Math classroom process**

The last session of Day 2 is divided into two main sections which will be covered in 60 minutes. This session will cover the topics mentioned below:

7.1 Measurement
7.2 Concluding teaching Math’s in grades 1-2

2.1 Measurement & Estimation	
**Objective**	Understand measurement and estimation and the activities related to be conducted for them
**Total Duration**	45 minutes
**Process of Facilitation**	**Big group**<br>● Facilitator to demonstrate the 5 quick activities on weight, capacity, length, height and distance as energizers in the big group.<br>● Facilitator to ask, “How many activities did we do?”<br>● Discuss the activities that were done and the process that was followed.<br>● At the same time co-facilitator will create a table with the 5 main concepts of measurement and explain the same using the ppt slide<br><br>**Small groups**<br>● Facilitator to ask the participants to open the measurement section in the math activity booklet and ask them to practice for demo in small groups.<br><br>**Big group**<br>● Demo of any one game.
**Classroom arrangement**	Big group
**Material Required**	PPT slides 51 and 52

For Facilitator: Measurement
Measurement is not only a mathematical concept but is an important skill that one.<br>uses every day in life. We come across many situations in our life which require us to measure different things using different units.<br>● Before discussing the standard measurement units, it is important for a teacher to discuss the non-standard units such as hand span, foot span, other objects around them etc. with children and encourage them to use these units for measuring things in their immediate environment.<br>● Once the child has understood how we use non-standard units, standard units can be introduced gradually. For example, meter to measure clothes, liter to measure milk and oil, kilograms for other things like fruits and vegetables, pulses and cereals, etc. Apart from these units, other units such as, centimeter, milliliter, etc. can be introduced gradually.<br>● Encourage students to observe the things present in their surroundings and find out what unit of measurement is appropriate to measure a particular object.

**2.2 Concluding teaching Math's in grades 1-2**	
**Objective**	Revise the main components of teaching Math's in the early grades and how to include them in the daily lessons
**Total Duration**	15 minutes
**Process of Facilitation**	● Facilitator asks 3-4 questions one by one about the different components of Math's.<br>● Participants to write the answer.<br>● Facilitator to conclude the session by discussing the answers to the questions in the big group.
**Classroom arrangement**	Big group and individually
**Material Required**	PPT slide 53

Assignment: Word Problem	
- Distribute an A5 sized sheet. Fold it into two. - On page 1, write ‘Word Problem’ on the top. - On page 2, write ‘Paragraph’ on the top. - On page 3, write ‘Story’ on the top. - On page 4, write ‘Gupshup’ on the top. Make two sub-headings of ‘Situation’ and ‘Incident’. - Ask them to write 2 addition and 2 subtraction word problems on page 1.	
**Material Required**	PPT slide 54

Day 2: Reflection	
**Total Duration**	30 mins
**Process of Facilitation**	- Facilitator will ask “Which activity did you like the most today?” - Each participant will share the activity that they liked the most.
**Classroom arrangement**	Big group
**Material Required**	PPT slide 55

DAY 3

TEACHING LANGUAGE IN GRADES 1 & 2

Day 3: Session 1
Attendance, Prayer & Recap

The first session of the first day is divided into two main sections. The total duration of this session is 30 minutes. This session will cover the mentioned below topics.

1.1 Attendance & Prayer
1.2 Recap

1.1 Attendance & Prayer	
**Pre work:**	• Assign the responsibility to the group to get the sign of every participant on the attendance sheet. They will make sure that attendance should be made before the session starts.<br>• Setting up the projector, relevant PPT on screen and required materials: stationery and print out of required material in required quantity.
**Objective**	Familiarizing with 6 days training and introduction to each other
**Total Duration**	15 minutes
**Process of Facilitation**	***Attendance***<br>• Facilitator provides the attendance sheet and asks Participants to sign on the attendance sheet.<br>**Recap**<br>• The group assigned to lead the recap session will lead it.<br>• Participants will sit in a big circle and each participant from the assigned group would share their learnings from the previous day.
**Classroom arrangement**	Big group
**Material Required**	PPT slide 57

1.2 Recap	
**Objective**	• Recall the topics covered on the previous day.<br>• Set the agenda for the day
**Total Duration**	15 minutes

**Process of Facilitation**	● The group assigned to lead the recap session will lead it.<br>● Participants will sit in a big circle and each participant from the assigned group will share their learnings from the previous day.<br>● The facilitator will make sure that every broad topic discussed on the previous day should get covered.<br>● The group assigned to lead the rules session will reiterate the rules.<br>● Facilitator will read and discuss the agenda for the day.
**Material Required**	PPT slide 58

**Day 3: Session 2**
**Teaching Language in Early Grades**

Day 3, session 2 focuses on teaching language in early grades (grade 1 & 2). This session will cover the main component of language teaching to young children and its connection with policy documents. Total duration of 60 minutes. This session will cover the mentioned below topics.

2.1 Teaching Language in Grades 1&2 & Connection with Policy Documents: NCF & NIPUN

**2.1 Teaching Language in Grades 1&2 & Connection with Policy Documents: NCF & NIPUN**	
**Objective**	Understand the components of language & how the components converted to daily classroom process for language in the light of policy document (NIPUN & NCF)
**Total Duration**	60 minutes
**Process of Facilitation**	**20 minutes**<br>● Discussion on the components of language development (with a focus on reading development in early years and comprehension)<br>● The facilitator discusses the components for teaching reading with the participants and the co-facilitator writes the points on the board.<br>**20 minutes**<br>● Facilitator shows the slides of the daily classroom process for language and literacy development aligned with NIPUN Bharat's recommendation of teaching first language.<br>● The facilitator will conclude by showing the framework of the daily classroom process and sharing the further plan of working on each component of language.

Classroom arrangement	Big group
Material Required	• Page from manual of language development in early grades<br>• Slide on transaction of language and literacy learning component.<br>• NISHTHA module 10 "Pedagogy of teaching language. Watch the video from time 7:00 to 8:45<br>• PPT slides 59, 60, 61 and 62

For Facilitator: Reading, An Initial Snapshot

Reading is our foremost tool to make sense of the world around us. We read sign boards, we read newspapers, we read books, we read texts that accompany news items on the television channels, we may also read for pleasure. But have we ever tried to look into what goes into reading? Here, it is suggested that you spend some time on thinking through this process of language that we all learn called "Reading". It is important to think what is exactly happening when you are reading this sentence or this paragraph? Let us list it out:

- We are putting together the letters to sound out what we call 'words' but we are doing it so automatically that we do not anymore see the individual existence of letters.
- We may even get stuck in the above process if we see a word that is difficult to read. We may want to sound its constituent parts gradually.
- Well, the above two are not the only ways we may want to read a word. If we know the meaning of the word, we may find reading it easy. Or,
- We may also read the whole sentence first and then try and guess the meaning of the individual word from the overall meaning of the sentence.
- We may read a bit and then pause and think about what we have read.

So, in reading we are almost simultaneously trying to deal with first, the script that represents a language and second, the meaning of what we are reading. Can we now say: Reading is deciphering script to create meaning?

So, now that we all want to learn to read, what could be the best way to teach us "Reading"?

Well, we may first learn all the consonants and the vowel sounds accurately. Then we may learn to put together the consonant and vowel sounds together to make syllables. Then we may put the syllables together to form words. Then we may put words together to make small sentences. Then we gradually read bigger sentences. And so on. Someday we will read the huge fat books that many scholars read.

This is a way to read, no doubt. The real question is: is this alone the best way to read? Let's examine when we learn to read words from letters, we are immediately dealing with a unit of language that brings some meaning to us. What are words? Imagine seeing a tree. We are not just seeing a shape or an object. We are also associating feelings like 'shelter' with it, 'greenery' with it. It is this link that we establish with what we see, enables us to give this experience of ours a 'name' and this 'name' is called a 'word' in language.

Similarly, the consonants and vowels that are shaped to us, when put together to form a word, suddenly brings a chunk of meaning to us. In this way, the word when used in a sentence may bring altogether a new meaning to us

than the mere individual word meaning.
So, if this ‘meaning making’ is a fundamental factor that helps us to read and it is right there with us from when we learn to put together words, can we use it to ‘read’ better? After all, reading is to make meaning. Without being able to make meaning out of a text, would you consider yourself a reader? No, for sure. But the method of reading we had earlier discussed, that first teaches us consonants and vowels and then words and then sentences, does not use the ‘meaning’ aspect very specifically. It thinks meaning will one day happen on its own and by itself.

Let us again think of another way of learning to read. This time we learn to read with the meaning making skill we have, without learning letters first. We are told stories; those stories are written in very simple language which we see. Then by seeing those stories repeatedly, we come to know certain words and their shapes. However, we haven’t been able to break the words into their constituent parts. So, we see the words as a whole unit of script. We gradually see more words and learn them. However, we have not learnt to join letters and create words ourselves. Gradually, we are told, we will do so.

The above may also be a way to learn to read. But the question still remains: is this the best way to read? The question remains primarily because, in both the reading methods, only some parts of the whole of what we need to read effectively are emphasized. Each method will help us read, no doubt. But there is also a lot that each method may need to take from the other.

These two methods have their own belief systems and underlying research. The first approach is more of a skill-based approach and called the Phonics method. Theorists may define the ‘phonics’ as:

“Phonics is an understanding that there is a predictable relationship between phonemes (the sounds of spoken language) and graphemes (the letters and spellings that represent those sounds in written language)” illinoisearlylearning.org/chat/marks/glossary.htm
The second method that we talked about is more “meaning based” and is called the Whole language approach. This method is formally defined as:

Whole language is an approach to literacy education that emphasizes natural development of literacy competency. Immersion in real literature and daily writing is favored over explicit teaching of basic reading skills. Skills instruction occurs in wholly committed whole-language classrooms on an ‘as- needed’ basis only, and then only in the context of reading and writing, rather than as a focal point of instruction. (Presley and Rankin, J. 1994. More about Whole language methods of reading instruction for students at risk for early reading failure. Learning disabilities, research and practice).

Learning to sound out letters and then encoding them into words is an important early reading skill. The ultimate goal of reading is good comprehension. But in order to understand what one is reading, he must be able to do it correctly and quickly, almost automatically, without stumbling over words. Phonics facilitate this process. Moreover, in early reading instruction in Indian languages, one must take the advantage of the near perfect fit of pronunciation and script.

Although it may be argued by some whole language enthusiasts that letter level recognition of words is a lower-level skill of reading, than recognizing words through their meaning; nevertheless, the skill is important, especially as mentioned in Indian languages, where the sound and the symbol are in sync with each other. It helps us to deal with even unfamiliar words, words that are not there in our vocabulary as children.

It has also been seen that in whole language classrooms children are immersed in story books and this helps children in improving both comprehension and writing, also there is gradual sophistication of language usage. (Fietelson, Kita, & Goldstein, 1996; Morrow 1992). Whole language instruction increases children’s understanding about the nature of reading and writing and stimulates them to do things that are literate. (Graham

& Morris, 1994).

In effect, it is seen that both these skills go hand in hand in reading development. And one thod, when coupled with the best of the other, gives a more comprehensive result. There are quite a few scholars who have suggested a balance in reading instruction in the early grades. (Adams, 1990; Chall, 1983).

Teachers, teaching reading in Indian classrooms, need to understand the increased need for comprehension strategy building in children. Especially because, as mentioned, proficiency in the near perfect fit of letter symbols and their sounds in Indian languages often make teachers think that decoding alone is reading.

So, how does a balanced approach to literacy look like, when active in a classroom teaching Indian languages? Keeping both the approaches to reading we just discussed in brief, we may summarize the most essential elements as follows:

- Lots of books and printed materials in the classroom which children get to read on a daily basis.
- Storytelling, story reading from a book, shared reading, guided reading where children and teachers are engaged in the process together.
- Good reading behavior is modeled.
- There is a lot of discussion around a text or around an activity.
- There are instructions in letter sound correspondences, both in the context of a story and in a de-contextualized manner.
- There is word- building games, word recognition games, and word meaning games.
- There is invented writing in classrooms.

But here is another point to stop and think about. Readers in the early grades will be exposed gradually to first, different types of texts and second, different levels of texts. What kind of reading strategy will help students comprehend the different types and levels of texts?

There are various types of texts. A narrative (story) has characters, a beginning, a complexity of plots within the story that gives direction to its flow, a solution and an end. A text from the EVS textbooks may not necessarily have a similar pattern. It will have an introduction, gradual unfolding of concepts, lots of technical vocabulary that will denote concepts etc.

Good comprehension teaches us to deal with different texts differently. And this is a skill in active comprehension building. Good comprehension relies on quite a few things for effective comprehension. They are namely, fluency in word recognition, activated background knowledge about the text being read, thinking about the text from various angles as it is being read,
vocabulary skills.

Interestingly, the more students engage with texts through various angles, the more are the various layers of meaning/ interpretation that a single text can accommodate. Such a sophisticated text reading enhances what we call cognition. Cognition may be defined as the process of ‘perceiving, thinking, reasoning and analyzing” (www.chop.edu/consumer/ jsp/division/ generic.jsp).

Enhanced cognition results mostly in refined expression. This is because reading strategies for comprehension and expressions of thought, oral and written, share some common features of development.

The moment readers can delve deeper into the meaning of the text and various kinds of texts, they start getting more conscious about language usages, more words, metaphors, information etc. The organization of thought that one requires mentally to retain these higher levels of understanding, are also the ones that are required for representing thoughts in oral or written form. So much so, some researchers think that critical thinking in Reading

and writing are likely to be reciprocal processes, commonly known as critical reading and revision in writing (Fitzgerald, 1989).

Thus, when we start seeing Reading as a process, there are so many dimensions that appear in front of us. One must be able to know the script well, must know how to create meaning out of the text effectively. And clearly, the above two-fold characteristic gets more and more refined as our comprehension increases and we get to read many types of texts. However, this is just a snapshot.

Day 3: Session 3

Hands on practice of language components

Day 3 session 3 will continue with teaching language in early grade. This session will cover three main components of language. Therefore, the session is divided into three main sections which will be covered in 2 hours 60 minutes duration. This session will cover the mentioned below topics.

3.1 Kaburlu (informal talk)
3.2 Story related activity
3.3 Phonological Awareness

**3.1 Oral Language Development:** Kaburlu (informal talk)	
**Objective**	Understand the skills that enhance oral language development
**Total Duration**	60 minutes
**Process of Facilitation**	**10 minutes**<br>• Facilitator to ask participant "meaning of oral language development" and the co-facilitator will write all the points on the board.<br>• Facilitator to summarize the discussion by saying "Oral language development means developing listening speaking skills in the child because oral language development plays a crucial role in learning to read in early years. Children bring their listening & speaking skill/learning at the time of entry of grade 1 which means children learn language before coming to school. They learn more than 8000 thousand words of word vocabulary and can form an infinite number of sentences using it.<br>**15 minutes**<br>• Facilitator to discuss types of activity for oral language development and Co-facilitators to write the types of Gupshup in day wise grid while facilitator discuss in group.<br>• The facilitator concludes using the slide.<br>• Quickly Facilitator will demonstrate all 5 different types of Gupshup.<br>**15 minutes**<br>• Participants to practice all five types of Gupshup in small group with the help of Language Activity Booklet<br>**10 minutes**

	● Demo on one type of Gupshup by each group in a big group.
**Classroom arrangement**	Big group and small group
**Material Required**	● Slide on types of Gupshup followed the step-by-step process and its things to remember.<br>● Phase 2 language manual page on "Baatchit/Gupshup"<br>● PPT slides 63 and 64

For Facilitator: Language Activity Booklet

If I Had

Classroom Arrangement : Big groups

Material: Nothing

Process: Nothing

- In a large group, tell the children, "Let's play a game. I will say something and you tell me if it had been you, what would you do?" For example- "If you are in a garden and suddenly a monkey appears, what would you do?"

- Let the children think for
- Sometime then ask them what did they think and what would they have done?

Some examples are, 'If you were a king/a queen/', 'I would visit the city'.... 'if it rains today then I/you....' Etc.

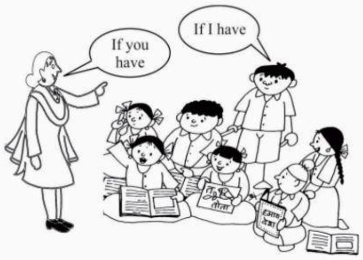

1 minute talk

Classroom Arrangement : Big groups

Material: Nothing

Process: Nothing

- Make some small chits and write the name of/draw an object. For example- Shoe, Bottle, House, Pencil, etc.

- Then place the chits in between the children and ask each child to pick one. Ask the children to think about the object for a minute.
- Then ask them to talk about the object. If the children are facing difficulties in talking, ask the children questions related to the object.

For example- What is it? What does it look like? Where do we get it/find it? How would it be if it were not there?

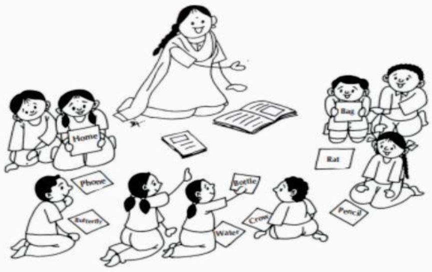

For Facilitator: Kaburlu (informal talk)

Children who get enrolled in classes 1 and 2 come with the knowledge of their native language. They already know how and when to use their language. Using their vocabulary, children are also able to create a large number of sentences according to their requirement or the situation they are in. This knowledge of how a language works comes from their experiences as well as the conversations that they make. In other words, a child's oral language development is based on his/her experiences. In the initial days, children talk about their experiences in their native language, and they are able to use this language in various contexts very skillfully. By using language in these various contexts and situations, they obtain many experiences. These experiences then help them in oral language development. Therefore, it is important for the teacher to listen and understand the language that his/her student uses in class and to also respect it. The important thing is that expressing themselves after thinking helps children to learn reading-writing as well.

Hence, coming from different backgrounds, these children come to school with already existing vocabulary and sentence frameworks in their native language and learn a new language in school. Thus, in schools when their spoken language is given respect and they are given the freedom to use it however they want, then shortening the distance between their language and learning school's language will become an enjoyable process.

- Talk about familiar and unfamiliar topics in class with the children daily.
- A discussion is not limited to just asking questions and giving answers.
- A good discussion includes the children talking about their experiences, and not just answering questions.
- Let the children use their native language during discussions.
- Listen carefully to what they have to say. Sometimes, in order to encourage the children to share their thoughts, pretend as if what they are saying is something you have never heard before or didn't know.
- Often use phrases like, "Oh! / What is that? / How is it done?"
- These phrases will encourage children to form answers in their language.

Example of activity related to informal talk.

1. If I had:
In a large group, tell the children, "Let's play a game. I will say something, and you tell me if it had been you, what would you do?" For example- "If you were in a garden and suddenly a monkey appeared, what would you do?" Let the children think for some time then ask them what they did?
think and what would they have done? Some examples are,
'If you were a king/a queen/', 'I would visit the city'.... 'If it
rains today then I/you....' Etc.

2. Discussion on a Picture
In a large group, show a picture to the children and talk about it. For example- "What is there in the picture?" and "What is happening?" Ask the children, "What do you think the story is hidden in the picture?" Give each child a chance to speak. In order to encourage what the children have to say, ask questions like, "Oh! What do you think happened next?" This way, while interacting with the children, a whole story can be formed orally.

3. Talk for one minute: Describe object
Make some small chits and write the name of/draw an object. For example- Shoe, Bottle, House, Pencils, etc. Then place the chits in between the students and ask each child to pick one. Ask the children to think

about the object for a minute. Then ask them to talk about the object. If the children are facing difficulties in talking, ask the children questions related to the object. For example- What is it? What does it look like? Where do we get it/find it? How would it be if it were not there?<br>***Note: These are sample activities, many other activities have been mentioned in the language activity booklet. Please refer that to practice in small groups and conduct with children.***

**3.2 Story-Related Activity**	
**Objective**	Understand the role of story in language development and how to translate this into classroom practice
**Total Duration**	60 minutes
**Process of Facilitation**	**25 minutes**<br>● Facilitators ask the participants to pick a story and read with voice modulation (1-2 participants)<br>● Then facilitator ask participants to talk about the best way of reading story for children with prosody<br>● Co-facilitator will write 2-3 responses and then the facilitator asks to tell the importance of reading story for language development.<br>● After that facilitator to demonstrate on story related activities for first days and next day<br>● Co-facilitators show the day wise grid for story related activity.<br>**15 minutes**<br>● Facilitator to discuss the similarity as well as difference of story related steps followed in 5 days.<br>● Facilitator will Participants to practice the same in small group.<br>●<br>**10 minutes**<br>● Demo on one type of Gupshup by each group in a big group.
**Classroom arrangement**	Big group and small group
**Material Required**	● Slide on types of story related activities followed the step-by-step process and its things to remember.<br>● Phase 2 language manual page on story related activity<br>● PPT slides 65-69

For Facilitator: From the Language Activity Booklet

Story-related

Carry out activities at least 3 times on one story within a week. In this way, 2 stories can be covered in a week. After narrating the story, ask questions related to the story every week. Carry out the following activities given below on every story –

Day 1

- Discussion on the name of the story
- Narrate the story with expressions and voice modulation.
- Discussion on the story – what happened, who did what etc.
- Use your finger to point and narrate the story so that the children listen and pay attention.

Day 2 to Day 5

- Ask the children to tell the story in their own words.
- Use your finger to point and narrate the story so that the children listen and pay attention.
- Ask the children to read.
- Discussion on the story – why, what, how and what if you were a character from the story etc.
- Ask the children to tell the story in their own words.
- Discussion on the story – meanings of different words and making sentences with those words
- Use your finger to point and narrate the story so that the children listen and pay attention.
- Ask the children to use their finger to point and read the story

- Use the textbook to narrate the story every day
- Every child should have a textbook with them.
- Ask different questions related to the story during the discussion every day.

For Facilitator: Story-Related Activity

In order to attract students towards reading and writing, the teacher should read the stories from a book in the initial period. Stories will be a great help. Using a lot of books with the students helps them to relate their learning process with written language as well. Through the medium of a story, students understand that 'reading' is not just reading but understanding as well.

A story's title, pictures and its written language are all very important. Development of reading ability should be done by reading to the students from a book every day. Additionally, discussing things related to the story should be encouraged. With the help of stories, reading ability and understanding develops at a faster pace. Ren to form answers in their language.

Example of activity related to story.

- Before reading the story, discuss the images and the title of the story. For example, for a picture ask questions like- "Who is there? What are they doing?" etc. (Talking about the story helps children to guess its plot and also helps them to try to relate it with their own lives).
- Read the story in a clear voice with proper intonation. Make sure the children are listening to the story carefully and not repeating the words after you.
- After reading the story, discuss the story with the children, "Who was there in the story? What happened

in the story? Who did what and why? Also ask questions using 'How' and 'Why'(sometimes these questions don't have just one answer. Every child can answer according to his/her understanding.) Listen to their answers carefully. Don't think that an answer is 'right' or 'wrong'.

- Ask the children to narrate the stories which were read to them on Day 1 in their own words.
- Give the children story books/cards. Then ask them to follow the words with their fingers while you read the story. After reading the story, ask "Who will read now?" and let 2-3 children read the story, one at a time. Next day ask the children to narrate the same story in their own words.
- Every day, pick some words from the story and the children have to write it down. (Make sure you tell the page number to the children) Also make sure that the children have written down the correct words. This way, pick 4-5 words each day and ask the children to write.

Do follow the points given below.

- Having an ideal reading of the story. Discussing questions related to the story.
- Encouraging children to try and read the story.
- While reading the story, discuss the important points in the story. Difficult words, synonyms, antonyms, finishing incomplete stories, etc.
- Reading dialogues of characters and acting out scenes.
- When you give the stories to children who cannot read with the required speed, try to make sure that they are able to connect their letter-sound knowledge with the written text.
- Encourage children to share their experiences related to the story. This way, the children are able to easily understand that the stories are connected with their lives.
- Each child should have his/her own story book.
- During the word finding activity, pick words which are fun and have been used frequently in the story.

Note: These are sample activities, many other activities have been mentioned in the language activity booklet. Please refer to that to practice in small groups and conduct with children.

<table>
<tr><th colspan="2">3.3 Phonological Awareness</th></tr>
<tr><td>Objective</td><td>Understand the importance and activity of phonological awareness to develop the reading- writing skill in the child.</td></tr>
<tr><td>Total Duration</td><td>30 minutes</td></tr>
<tr><td>Process of Facilitation</td><td>Big group (15 minutes)
● Facilitator to demonstrate an activity for phonological awareness.
● The facilitator asked participants "how does the phonological awareness game help children to learn language?
● Co-facilitators to write point comes from participation.
● Facilitator to conclude by saying that phonological awareness skill is important to develop reading and writing skill in the child. It helps children to -understand the connection between sound and script and develop metalinguistic awareness. Therefore, these kinds of activities are important, now can you please tell me steps.
● Co-facilitators pull the slide on phonological awareness while facilitators discuss in a group.
Small Group 15 minutes
● After discussion, ask participants to practice the phonological awareness activity in small group (activities mentioned in Chalo khelein booklet)
● Demonstration by the participants in a big group (by selective 1 group)</td></tr>
<tr><td>Classroom arrangement</td><td>Big group and small group</td></tr>
<tr><td>Material Required</td><td>● Slide on types of Importance followed the step-by-step process and its things to remember.
● Phase 2 language manual page on "Phonological Awareness"
● Chalo khelain activity booklet
● PPT slides 70-72</td></tr>
</table>

For Facilitator: From the Language Activity Booklet

Say - How many sounds?

Classroom Arrangement : Small/Large group.
Material: Nothing
Process: Oral & written

- Sit in a large group.
- Say any one word. Like 'water'.
- How many sounds are there in this word?
- What was the first sound?
- Like this, say other words, break the words. In total how many sounds were there in the first, second and last words? Ask.

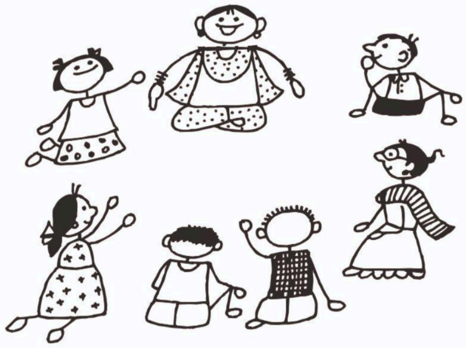

Let's try this too

- In the beginning say only words which have 2 sounds, when children identify with 2 units of words then ask to apply 3-4 units words
- Play this game with English words also- like how many sounds are there in the word-'water"?

Change the last sound

Classroom Arrangement : Small/Large group.
Material: Nothing
Process: Oral & written

- Sit in a group.
- Speak any one word, like- 'Nani'.
- Ask the children, how many sounds are there, first, last and which one?
- Now ask the children, what will happen if 'na' is replaced by 'pa'?
- Similarly, say the word, break it first into its first and second sounds. Change the last sound and ask them to make a new word.

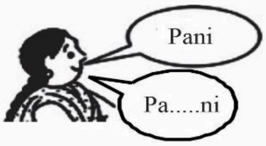

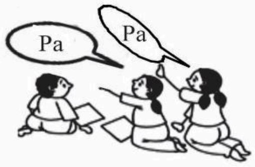

Let's try this too

- In the beginning, speak only 2-syllable words, when children become familiar with the sounds of 2-unit words, then use 3-4-unit words.
- Play this game with English words also like- What will happen if 'p' is used in place of 't' in cat and mat?

For Facilitator: Phonological Awareness

To recognize and understand various sounds in words and sentences, conducting sound-related activities becomes important. An understanding of breaking and combining sounds to make words helps in developing reading-writing abilities in a faster way. In order to master the understanding of sound, it is important to conduct different types of games in class. For example- Breaking words into different sound patterns, pronouncing different sounds to form words/sentences, understanding the first and last sound of a word, changing the first or last sound of a word to form a new word or even listening to a sound and guessing what word could it form.

Things to Remember

- Start with words having only 2 syllables.
- Always play the sound games orally first so that the children understand that 'words' are made up of sound symbols.
- Sound games can be played with words taken from a story.
- Play this game with 3-4 words daily.
- Don't use compound words for this game.

Note: These are sample activities, many other activities have been mentioned in the language activity booklet. Please refer to that to practice in small groups and conduct with children.

Day 3: Session 4

Continue with hands on practice of language components

Session 4 of day 3 continues with decoding skills and will discuss the developing writing skill in the child and its activity. This session is divided into 3 main sections and will cover it in the next four covers in the next 1 hour 30 minutes time duration. This session will cover the mentioned below topics.

4.1 Decoding: Guninthalu activity to develop decoding skill.
4.2 Game: Level-wise activities to develop decoding skills.
4.3 Writing: Tool of expression

4.1 Decoding: Guninthalu Chart Reading activity to develop decoding skill

Pre work	Keep the Guninthalu chart for the facilitator and the copies of Guninthalu cards ready for participants. .
**Objective**	Understand the role of Guninthalu in reading-writing development and activities related to it
**Total Duration**	30 minutes
**Process of Facilitation**	**15 minutes**<br>● Discuss the importance of Guninthalu "how can Guninthalu help children to read and write? (5 minutes)<br>● Conclude the discussion with "infinite number of words and sentences can be formed and read if the child understands the Guninthalu pattern and is familiar with it.<br>● Now the facilitator will do the Guninthalu chart reading demo followed with the steps given in manual.<br>● Then ask the participants to write the steps in their notebook.<br>**15 minutes**<br>● Ask the participants to practice the same in small groups.<br>● Facilitator and co-facilitator will observe the small group activity and help them to do correctly whenever is required.<br>● One demo from a selective group.
**Classroom arrangement**	Big group, small groups
**Material Required**	PPT slides 73, 74 and 75, Guninthalu chart, cards and manual

For Facilitator: Decoding

In order to perfect the understanding of letters it is important to conduct activities which help in practicing the coordination of sounds and symbols of letters. This means that only learning the letter sounds or only learning how to write letters is not enough. The focus should be on understanding how letters and letter sounds work together, that is, finding the symbol for a spoken letter symbol and looking at a symbol and telling the sound it will make.

Each sound is also a symbol, it is important to understand this to develop reading-writing abilities. Good understanding of the coordination of sound symbols makes it easier for children to identify and understand a word. Therefore, out of the given games, make the children play symbol-identification games daily.

Things to Remember

- The letters that are taken for these games can be taken from an order. Sometimes, we can also take letters which are not easy to recognize, or which keep appearing in the stories.
- Use the letters used in these games to form words.
- In the initial days. do not use letters whose sounds or symbols are almost the same so as to avoid confusion. For example- ^y** and ^/k*, ^V* and ^r*, ^p* and ^N*, etc.
- The letters used on a day should be revised the next day along with introducing new letters.

Note: These are sample activities, many other activities have been mentioned in the language activity booklet. Please refer to that to practice in small groups and conduct with children.

4.2 Game: Level-wise activities to develop decoding skills	
**Objective**	Understand how to identify different levels in a language classroom and thereafter allocate level appropriate tasks
**Total Duration**	30 minutes
**Process of Facilitation**	**15 minutes**<br>● Discussion on different levels in language and appropriate task for them<br>● Facilitator quickly gives a demo of conducting level wise game and discusses the step need to be followed.<br>● Co-facilitators pull the slide on level wise game while facilitator discuss in group.<br>**15 minutes**<br>● Participants to provide the Language activity booklet and ask them to practice in small group.<br>● Facilitator will move around and observe the small group practice help them whenever is required
**Classroom arrangement**	Big group and small group
**Material Required**	● Slide on types of Importance followed the step-by-step process and its things to remember.<br>● Phase 2 language manual page on "level wise game"<br>● Chalo khelain activity booklet<br>● PPT slides 76 and 77

For Facilitator: From the Language Activity Booklet

Find letters

Classroom Arrangement : Small groups

Material: Barakhadi card, chalk/pencil

Process: Oral and written

- Sit in small groups of 4-5.
- Write any one letter on the blackboard and ask the children to say this is "M" and they have to now find the letter "M" in their textbook or card.
- Once the children find the letter, ask them to write that letter on the floor/slate/wall.
- After repeating the above activity for 4-5 letters, tell the children, "I will say one letter and you will point your finger and tell me where have you written that letter?"
- All the children will make 2-unit words with the above letters.

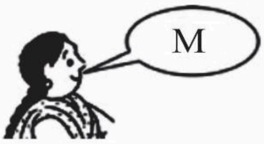

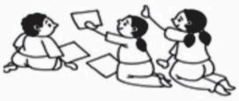

Let's try this too

- You can try this activity even with letters in English. Wherever possible encourage the children to use English vocabulary and use the vocabulary yourself as well. For e.g., find "M" and then write in notebook/wall.

Jump to letter

Classroom Arrangement : Small/Large group

Material: Nothing

Process: Oral and written

- Draw circles on the ground as in point 3.4.
- Write different letters in each circle, like – M, T, N, L
- Ask the children to stand outside the circle.
- Say a letter such as L and have all the children jump into the circle with the correct letter.

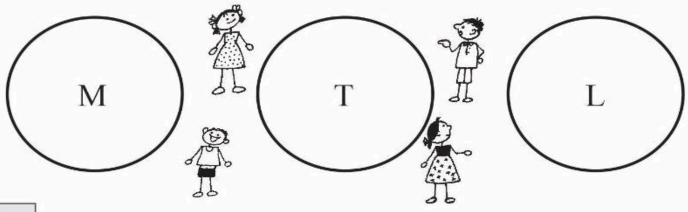

Let's try this too

- Don't play this game with more than 3-4 letters a day.
- This game can also be played for letter recognition of English
- Use English vocabulary/sentences wherever possible, e.g., jump on the letter "M".

New Words

Classroom Arrangement : Individual

Material: Barahkhadi chart, chalk

Process: Written

- Write some units of the Barahkhadi chart on the board like - 'ki', 'ma', etc.
- Make 1 or 2-letter words for children using the Barahkhadi like 'Kima', 'mali', etc.
- Then ask the children to make new words using the same chart.
- Once done, ask the children to read the words they have made.

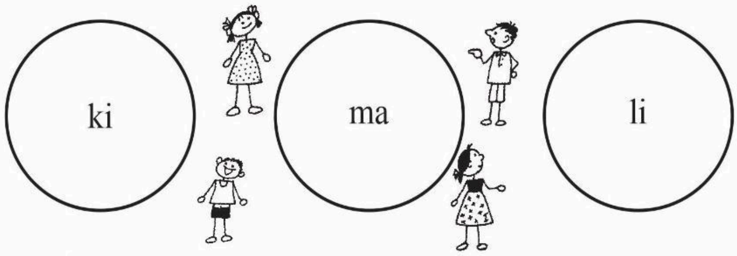

Let's try this too

- Ask children to read whatever words they have made.
- Wherever possible, try to use English sentences/vocabulary.

I Say, You Do

Classroom Arrangement : Small/Large group

Material: Nothing

Process: Oral and written

- Stand in a large group.
- Ask children to do as you say.
- Now say any action word like laughing, crying, etc.
- Now ask the children to act as per the action said.
- After this, ask children to say action words and ask other children to act on the same.

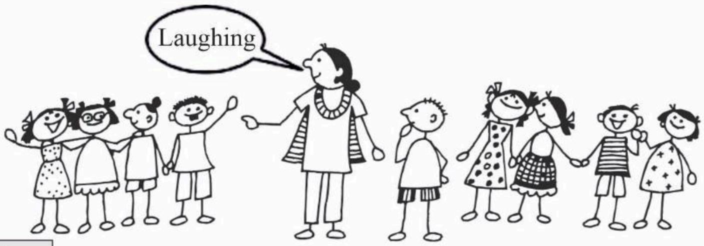

Let's try this too

- Try to play this game with English action words.
- Wherever possible, use English sentences/vocabulary.

Antakshari of Words

Classroom Arrangement : Small Groups

Material: Nothing

Process: Oral and written

- Make 4-5 small groups.
- Say a word like 'Mona'
- Ask the first group to identify the last sound of the word - that is 'na'.
- Now, the second group has to say a word starting with 'na'.
- Just like this, ask the groups to say words one by one and write them down.

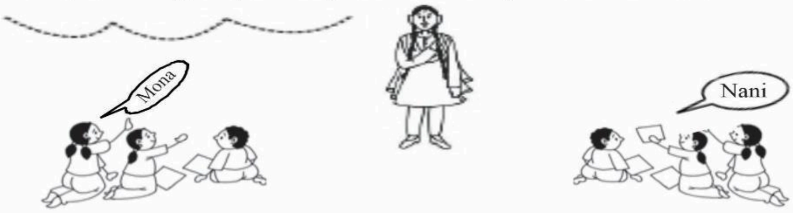

Let's try this too

- Try this game with English words too.

4.3 Writing: tool for expression	
**Pre work**	Keep the Guninthalu chart for the facilitator and the copies of Guninthalu cards ready for participants. .
**Objective**	Understand the role of Guninthalu in reading-writing development and activities related to it
**Total Duration**	30 minutes
**Process of Facilitation**	**15 minutes**<br>● Facilitator shows the video of guided writing and then demonstrates the same.<br>● Co-facilitator writes the steps while the facilitator discusses and demonstrates.<br>● Then ask the participants to recall the steps before the practice in small groups.<br>**15 minutes**<br>● Ask the participants to practice in small groups.<br>● Facilitator and co-facilitator will observe the small group activity and help them to do correctly whenever is required.<br>● One demo from a selective group.
**Classroom arrangement**	Big group, small groups
**Material Required**	PPT slides 78, 79 and 80, Guninthalu chart, Guninthalu cards

For Facilitator: From the Language Activity Booklet

Come on, let's make something

Classroom Arrangement : Individual

Material: Copy, Pencil/Color

Process: Written

- Sit in a large group
- Tell the children, today we will make anything we like on the floor.
- Now tell the children to draw any picture that comes to mind.
- Go to each child and ask what he/she has made
- Go to the child and do praise and applaud them on what they have made.

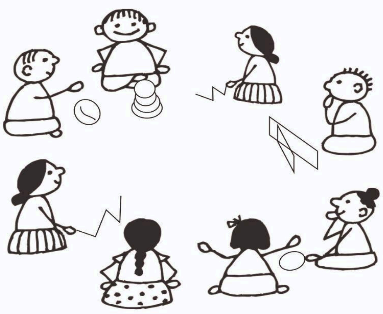

Let's try this too

- Write down whatever the children have told you.
- In the beginning, move the children in a criss-cross line and then do applaud them.

Guided Writing

Classroom Arrangement : Individual

Material: Barakhadi Chart

Process: Written

- Sit in a large group
- After conversation is done, ask the children to repeat a sentence from the conversation that happened during the chat.
- Now ask them to break down the sentences into words.
- Draw as many lines as the words on the board . The children should also draw the same number of lines on the floor/copy.
- Now break each word into sounds
- Find and write down the sounds on the Barakhadi chart,

Let's try this too

- During the guided writing, try to make patterns for writing sentences by breaking down the words and sentences into sounds.
- The understanding of writing should be developed in the children so that the children themselves start writing.
- Ask the children who can write to sit in a group and write.

For Facilitator: Writing Development

Encouraging students to express their ideas with the help of a sheet of paper and a pencil is an important precursor to writing practice. Initially, the students may write in a way which makes it difficult to understand but a small instruction like, "draw anything" and asking, "What did you make?" makes the students think about the lines they have created and encourages them to use words to express their thoughts. Even though words are made up of letters and sentences are made up of words, writing doesn't mean only being skilled in making symbols and letters. Rather, writing actually means thinking about an idea and presenting it on paper. Often, children are unable to understand that putting into words what they think about an idea is writing only. Here words and sentences made out of symbols and letters play a huge role. Therefore, from the first day itself, ask the students to draw or write anything that they can after they listen to a story, even if they present their thoughts with the help of crooked lines. This activity is very important for the students to express themselves and also helps in developing their writing abilities.

Things to Remember

- In the initial period, the children are only able to draw straight and crooked lines. Let the children express themselves through these lines.
- It is possible that in the beginning some children write the letters incorrectly. In such cases the teacher should help them separately.
- The practice of writing letters by looking at them should not be forced in class.

Note: These are sample activities, many other activities have been mentioned in the language activity booklet. Please refer to that to practice in small groups and conduct with children.

Day 3: Session 5

Textbook transaction & Uses of Material

The fifth session of the fifth day is divided into two main sections. The total duration of this session is 60 minutes. This session will cover the topics mentioned below:

5.1 Textbook Transaction: Telugu, Math & English
5.2 Material for grades 1-2

5.1 Textbook Transaction: Developing Lesson Plan	
**Objective**	Understand how to use a textbook as resource material to develop foundational literacy and numeracy
**Total Duration**	30 minutes
**Process of Facilitation**	**Big group**<br>● Facilitator to show and discuss the templates of the Telugu and the Math's lesson plan with the participants.<br>● The facilitator asks the participants about the uses of a textbook for developing a one-day lesson plan on a given subject.<br>**Small Groups**<br>● Facilitator assigns each group member a subject book (English, Telugu, or Math).<br>● Each group member exchanges their plan within their group member to review.<br>**Big Group**<br>● Facilitator moves around and gives feedback and covers any points not presented by the groups.
**Classroom arrangement**	Big group, small groups
**Material**	PPT slides 81 and 82

5.2 Material for Grades 1-2	
**Objective**	Understand the need of materials in each class
**Total Duration**	20 minutes
**Process of Facilitation**	● Facilitator to divide the board into 3 rows/columns.<br>● Write the name of one subject in each column.<br>● Now, ask the participants to list out the material required for each subject.

Classroom arrangement	• Big group
**Material**	PPT slide 83

**Day 3: Reflection**	
**Total Duration**	5-10 mins
**Process of Facilitation**	• Facilitator will ask "Which activity from today do you think children will enjoy the most?"<br>• Each participant will share one activity they think children will enjoy the most.
**Classroom arrangement**	Big group
**Material Required**	PPT slide 84

DAY 4

ENGLISH AND ASSESSMENT FOR EARLY GRADES

Day 4: Session 1
Attendance Recap

The first session of the fourth day is divided into two main sections. The total duration of this session is 30 minutes. This session will cover the mentioned below topics.

1.1 Attendance & Prayer
1.2 Recap

1.1 Attendance and Prayer	
**Pre work**	Keep the attendance sheet ready.
**Objective**	Mark the attendance and sing a prayer before starting the day
**Total Duration**	15 minutes
**Process of Facilitation**	• The facilitator will provide the attendance sheet and ask participants to sign on the attendance sheet.<br>• The group assigned will conduct the prayer.
**Classroom arrangement**	Big group
**Material Required**	Attendance sheet, PPT slide 86

1.2 Recap and Agenda for the Day	
**Objective**	• Recall the topics covered on the previous day.<br>• Set the agenda for the day
**Total Duration**	15 minutes
**Process of Facilitation**	• The group assigned to lead the recap session will lead it.<br>• Participants will sit in a big circle and each participant from the assigned group will share their learnings from the previous day.<br>• Facilitator makes sure that every broad topic discussed on the previous day should get covered.<br>• The group assigned with leading the rules session will reiterate the rules.<br>• Facilitator reads and discusses the agenda for the day.
**Classroom arrangement**	Big group

**Material Required**	PPT slide 87

**Day 4: Session 2**
**Other Aspects and Introduction to multi-grade, multilevel and multilingual classrooms**

The third session of the fourth day is divided into two main sections. The total duration of this session is 1 hour. This session will cover the topics mentioned below:

2.1 Other Aspects
2.2 Introduction to multi-grade, multilevel, multilingual classrooms

**2.1 Other Aspects**	
**Pre work**	Keep the printouts of the phase III manual ready.
**Objective**	• understand how positive teacher behavior looks like<br>• understand how to build a positive classroom environment.<br>• discuss the strategies to build reading habits in children.<br>• discuss how a print-rich classroom looks like
**Total Duration**	30 minutes
**Process of Facilitation**	**Small groups**<br>• Distribute Phase III manual: pages on classroom environment, reading slot, etc.<br>• Assign one component to each group. Each group will list out strategies for the component assigned to them (positive teacher behavior, positive classroom environment, building a reading habit and a print-rich classroom).<br><br>**Big group**<br>• 4 groups will present their ideas and will be given feedback.<br>• Facilitator will conclude the session by summarizing it.
**Classroom arrangement**	Small groups, big group
**Material Required**	Phase III manual: pages on positive teacher behavior, positive classroom environment, building a reading habit, and print-rich classroom.<br>PPT slide 88

For Facilitator: Other Aspects for Learning Environment

Reading time
Imaginary and short stories are interesting for the children as well as connected to their lives and environment due to which reading and learning becomes simple and entertaining in the initial days only. However, it is imperative to have reading practice of informational and various types of articles like letters, travelogs, autobiography and poems. The practice of reading written materials of different forms helps in strengthening the ability to read and understand. Hence, various materials for reading like newspapers, magazines, articles, story books, letters etc. should be available in the class for students.

The books in the reading corner should be within the reach of the children. Children stay interested if the books in the reading corner are changed every month. Ask for children's opinions about the books once a week. Reading corner should necessarily have reading materials for different levels of children.

Teacher Behavior & classroom environment

- Call students by their names and use ^Vik* to address them.
- Help the students form large and small groups in the beginning and gradually encourage them to form groups on their own.
- Seek permission before entering or leaving the classroom, make sure the class is neat and clean and keep things in their assigned place.
- Discuss with students the importance of standing in a line and walking in a line. For example: Going to the assembly, coming back to the class after the assembly and going out for lunch during break.
- Before the class begins, pick up wastepaper material along with the students.
- Discuss the importance of washing hands before and after every meal and after using the toilet.
- Encourage the students to take baths, comb their hair every day and wear clean clothes. Clap for the students who come clean and tidy to school.
- Discuss with the students the importance of listening to others and expressing their thoughts clearly, to wait for their turn and raise their hand before answering a question.
- Discuss the classroom rules with the students.
- If a student violates any of the classroom rules, do not scold her/him but try to discuss the problem and find a solution.

Note: These are sample activities, many other activities have been mentioned in the language manual and activity booklet. Please refer to that to practice in small groups and conduct with children.

2.2 Introduction to multi-grade, multilevel, multilingual classrooms

**Objective**	Strategies to manage multi-grade, multilevel and multilingual classrooms
**Total Duration**	30 minutes

**Process of Facilitation**	**Big group**<br>● Facilitator to ask, "What are the kind of languages you have heard/ you can expect to hear in a Grade 1-2 classroom here?"<br>● Co-facilitator to write the languages on the board (4-5 responses)<br>● Then the facilitator asked, "What are the different levels that would be found in a Grade 1-2 classroom?"<br>● Co-facilitator to write the different levels on the board (4-5 responses<br><br>**Small groups**<br>● Facilitator shares one of the following situations related to a multilingual classroom in each group (NIPUN):<br>a. Possible Situation 1: Child's language is different from the school language, and textbooks.<br>b. Possible situation 2: Child's home language is a combination of two or more languages, and the language of the school is different.<br>c. Possible Situation 3: Two different grade children sit together and single teacher is responsible to teach them.<br>d. Possible Situation 4: Different level of children in one grade<br><br>Each group lists down the different strategies they will use in the classroom to manage the specific situation on a chart paper.<br><br>**Big group**<br><br>● Each group lists down the different strategies they will use in the classroom to manage the specific situation on a chart paper.<br>● Facilitators ask groups to exchange the charts with another group.<br>● The group will read other charts and give them the stars (1 to 5) and can also add any points which are relevant.
**Classroom arrangement**	Small groups, big group
**Material Required**	PPT slides 89-92

For Facilitator: Multilingual classrooms

Multilingual in India

- Multilingualism is a rich source for the classroom in order to learn language. It not only brings diversity to the classroom but also gives a child a sense of self while practicing language.
- The languages of children might enter the classroom but will get switched as soon as the child learns the mainstream language and it can lead to monolingualism.
- When we use translanguaging, many times the languages lose their identity to the mainstream language.
- The linguistic flexibility should be maintained in the classroom even if the major language is the primary one. Multilingualism is complex. Everybody is multilingual. In our everyday practice we are either trans or multi language.
- Translanguaging is natural. We are wired for language. We employ grammatical structures to use or speak a language. It is spontaneous whereas multi - language is political because it is deliberate.
- We don't separate language from the classroom. We start a conversation in consideration with the language they bring in the classroom with all the lexical structures to communicate with each other. This helps a child to learn other languages as well.
- The languages remain in the hierarchy in India. The power gap is so huge. When we use translanguaging as a pedagogy, it can be used as a tool for assimilation into the mainstream language.
- Natural use of your repertoire of language should be there for best expression in the classroom.

Strategies for multilingual class: To develop the second language in child.

- Content and input both focus on oral language development in the second language at the early stage with the help of a variety of activities, stories, poems, rhymes and language games.
- The instruction or input in a second language must be comprehensible for children by using expression, body language or use of bilingual approach or maybe picture.
- In the initial months or stages building the vocabulary of a second language with the help of simple conversation, picture, rhymes and word wall or games plays a crucial role in second language acquisition.
- Provide a learning environment in which children can confidently express themselves in their own language.

Note: These are sample activities, many other activities have been mentioned in the language manual and activity booklet. Please refer to that to practice in small groups and conduct with children.

Multi Grade & Multilevel

One teacher for classes 1 and 2.

It is possible that in all schools, there will be at least one teacher available for children of classes 1 and 2 for teaching. In such a situation, it can be easy to teach both classes together because the level of reading and teaching of children in classes 1 and 2 will generally look the same. Therefore, the task of teaching both classes together can be done this year.

One teacher for class 1 to 5

There might be some schools where there will be only one teacher available for children from classes 1 to 5. This means that the responsibility of the entire school and all the classes will be on one teacher. It can be a little challenging to work with all the children in such a situation, but we have found that teachers often create one group for classes 1 and 2, and another group for classes 3 to 5. Following this plan, it becomes easier to teach both groups by allocating dedicated time to each. Therefore, in this situation, create two groups and teach one group before lunch and the other group after lunch. After teaching a group, give them some tasks to do independently. Additionally, make sure to appoint some children as leaders of small groups who can assist other children in your absence.

Some Special Children

As teachers, we all know that sometimes in our class, there are 1 or 2 children who are a bit different from the others. They may behave or learn differently. We can observe some special things about them, such as:

- Slow learning pace
- Not participating well in the activities
- Very low attention span
- Doing something of own or keep on playing something.
- Always being silent, not talking to anyone.

These are the special children who may require some special teaching methods and behavior. Therefore, when working with these children, it is important to pay attention to certain things:

- Interact with these children in the classroom just like the other children.
- Do not seat them separately or treat them differently in the classroom.
- Include them in class activities just like the other children.
- It is necessary to be patient when working with these children.
- Avoid comparing them with other children as much as possible.
- Regular communication with the parents of these children is equally important as with the other children.

Day 4: Session 3

Second Language Learning

The third session of the fourth day is divided into two main sections. The total duration of this session is 1 hour 30 minutes. This session will cover the topics mentioned below:

3.1 Second Language Learning: Exposure to English through visual and oral vocabulary building

3.2 Understanding of educational ecosystem and different stakeholders.

For Facilitator: Strengthen community and family engagement in early learning practices

The community is an important pillar to ensure that children's environment outside school (at home, in group spaces) is conducive to their growth and development. There are a range of actors in the child's vicinity who have the potential to influence their trajectories through their interactions. The role of various community members, including parents, SMCs, Panchayats and local youth, needs to be identified and demonstrated, such that it complements what is happening in the classroom. Involving the community with a view to create greater ownership over children's learning will also create a strong "demand" to improve functioning of anganwadis and schools and call for greater engagement and communication with teachers, thus increasing accountability of the schools and its administration. Limited awareness among communities of policies and structures put into place by the state as well as objectives of the local anganwadi/ school system need to be tackled such that a collaborative effort can be made.

Moreover, in the current reality where students have not attended school in over a year and will continue remote learning, involvement of family members, especially mothers, becomes very important. The NEP 2020 also notes the importance of community engagement.

The Anganwadi system has strong community connections. Mothers' meetings are conducted on a weekly basis. ECCE days are conducted on a monthly basis. Adolescent girls are engaged for a range of services. Home visits are also a key focus area. This understanding needs to be brought into the classroom setting in schools. It should be ensured that the continuum approach provides for the continuation of these community initiatives even once the child is in school.

In order to achieve this end goal, the Consortium intends to strengthen community involvement through direct engagement with local initiatives and training of schoolteachers and school administrators to successfully include stakeholders of the community into a proper structure of collaboration and accountability. Finally, a large-scale awareness and advocacy movement is required to create greater understanding between community members and teachers and workers.

The key outputs to be delivered by the Consortium under this component are given below.

1. **Community mapping to identify stakeholders and local networks and initiatives:** Region specific community stakeholder mapping will be conducted to create a database. Existing local initiatives will also be mapped, which can be supplemented through the Consortium's efforts. Additionally, information related to access to devices (smartphones and regular phones) and internet penetration will also be recorded in the mapping exercise. The Consortium will conduct extensive conversations with parents, especially mothers, as well as other family members and community youth.

   Through the above efforts, the Consortium will collate a region-wide data and further analyze pre-existing initiatives currently being implemented in the communities. Focus will be put on policies and practices in aspirational districts as well.

   The final step in the community mapping exercise would be to utilize the data thus collected to identify pertinent stakeholders within the community who may be leveraged to drive large scale change within the community. This would enable the government to understand the functioning and practices of local actors and consequently chart out an effective community engagement strategy.

2. **<u>Creating a community-school engagement strategy</u>:** Driving constructive activities with regard to children's learning in communities, Anganwadis as well as schools will strengthen the learning continuum and foster a connection between the community and the schooling system. Given the current situation, the Consortium envisions implementing remote learning activities as well as in-person ones and more importantly creating a balance between the two.

   A. <u>In the case schools reopen:</u> For more than a year, students have not attended school physically. Engagement activities held in communities called "school readiness fairs" would enable communication and collaboration amongst AWWs, teachers and mothers. These fairs will have components of capacity building of mothers where they will be trained to assist and complement the learning of their children with simple activities at home. Simple activities will be conducted in such fairs such as narrating a story, walking on a straight or zig-zag line. The observations of the child's activities will be documented through Progress Cards which will be handed to the mothers. This progress card will serve as a useful tool to discuss children's developmental outcomes with parents and teachers.

   B. **<u>During closure of schools:</u>** Focus will be more on remote learning materials as well as engagement material for key stakeholders (SMC members, parents, youth) in the form of audio content, audio-visual content, as well as print content. These resources will be disseminated based on the most effective mass-media platforms such as Radio, TV, phone calls etc. to maximize reach. Through the community mapping exercise, the teams on the ground will have the data regarding access to devices and internet connectivity. In areas where said access is limited, other remote learning activities such as a "loudspeaker activity" may be conducted.

   Through such activities, the Consortium expects to establish an effective system to capture the feedback loop between parents and teachers. WhatsApp groups between parents and teachers/workers will be created to share photos and videos of learning activities and other relevant information. Monthly meetings will also be organized to ensure that there is regular communication around learning and children's progress. Moreover, community activities will be organized using low-cost, local resources easily available in the community. One example is the creation of community-based mothers' groups to create a culture of sharing thoughts and ideas, engaging in discussions and helping each other tackle common challenges. Mothers of children in the catchment area of the Anganwadi centers and schools will be grouped by neighborhood. Workers and teachers can periodically engage with these mothers' groups and introduce them to simple activities which can be conducted with children.

   In order to successfully implement the above-mentioned activities, the consortium will train the three main groups of stakeholders involved, i.e., workers, teachers and administrators, community members especially the mothers, and school management committee members.

3. **<u>Creating and launching a scalable advocacy campaign closely involving community stakeholders</u>:**

   The Consortium also seeks to conduct periodic community events to engage local stakeholders at large, including government officials, Anganwadi workers, schoolteachers, parents and children. Examples of these initiatives include:

   A. Attendance Drive: To augur increased attendance rates across the districts of the state. This will be a combination of home visits and meetings at schools.
   B. Enrollment Drive: To mobilize and encourage more parents to enroll their children into schools, especially those who are already enrolled in Anganwadis. This will be particularly important in a post-COVID scenario given that children have been outside the school system for over a year.
   C. School Readiness Fairs: Community based assessment fairs will be organized which are attended by children who are about to enter Std. 1, along with their mothers. Stalls will be set

up by local community volunteers, for assessing developmental competencies through a variety of fun activities, in the presence of the mothers. Examples of activities to be assessed in these fairs include walking on a straight line, classifying and matching, coloring, narrating a story, counting using objects etc. The observations of the child's abilities will be documented through Report Cards which will be handed over to the mothers. Materials such as books, number and letter charts and worksheets will also be provided to the mothers to conduct activities with their children at home. After a period of about 20-30 days, another Fair will be organized to capture any improvement in the children's developmental abilities. Other events, where young mothers and family members can demonstrate their learnings with children as a result of the intervention will also be conducted in the community.

D. Reactivation Drive: Finally, efforts will be concentrated to strategize solution models to make the School Management Committees more involved in students' learnings and outcomes which would include encouraging greater accountability and initiative within them.

The advocacy and awareness campaign will include mass mobilization of stakeholders at the district level and will be trickled down to the sub-district level - cluster, block, school, and particular department - through volunteers.

**3.1 Second Language Learning: Exposure to English through visual and oral vocabulary building**	
**Objective**	Understand how to use posters and basic phrases to introduce and expand English vocabulary
**Total Duration**	2 hours
**Process of Facilitation**	• The facilitator asks participants to list down some common greetings, expressions, and commands that can be used with and by the children.<br>• Facilitator goes through the list of common greetings, expressions, and commands listed out in the English PDF<br>• Facilitator and participants discuss different situations during which the expressions and commands can be used.<br>**Posters**<br>**Big Group**<br>• Facilitator shows the three posters to be used in the lessons.<br>• Facilitator explains and demonstrates the 3 activities to be used.<br>○ The first being reviewing familiar vocabulary by asking individuals to name things or objects that they recognize in the scene depicted in the poster.<br>○ The second is to teach new vocabulary, the selection for which is done by knowing what the class already knows as is covered in the first poster activity.<br>○ The third is a comprehension check, which is done by asking various questions relating to both new and familiar vocabulary.<br>**Small Groups**<br>• Facilitator splits group into smaller groups and asks small groups to demonstrate the three activities that need to be covered in the poster

	classroom process
**Classroom arrangement**	Big group, small groups
**Material Required**	PPT slides 93-96, Posters

**3.2 Understanding of educational ecosystem and different stakeholders**	
**Objective**	• understand the potential stakeholders in the community.<br>• understand their roles and contribution in learning
**Total Duration**	30 minutes
**Process of Facilitation**	• Facilitator to ask, 'Where does a child start his/her learning first?' *(Exemplar participant response: home)*<br>• Facilitator to ask, "Who are the people in the child's immediate environment?"<br>• Take 6-7 responses. List down the responses on the whiteboard to discuss all the stakeholders in the child's learning journey.<br>• Facilitator to ask, "How does each stakeholder impact the child?"<br>• Make a segue to one of the most important stakeholders, that is, the parents and more importantly, the primary caregiver. The next session can begin from there.
**Classroom arrangement**	Big group

Day 4: Session 4

Different Strategies for Parents Involvement

The fourth session of the fourth day is divided into two main sections. The total duration of this session is 1 hour 10 minutes. This session will cover the topics mentioned below:

4.1 Different strategies of parent's involvement: Organizing Readiness Mela
4.2 Different Strategies for Parental Involvement: Formation of mother's groups, Parents' meetings, Sharing idea cards.

**4.1 Different strategies of parent's involvement: Organizing Readiness Mela**	
**Objective**	Organize the readiness mela and understand its importance
**Total Duration**	50 minutes

**Process of Facilitation**	**Big group**<br>● Facilitator will ask “Why is parental investment important for a child's holistic development?”<br>● Facilitator will ask “Who are the primary caregivers for most children?”<br>● Facilitator will show the video on readiness mela & discuss the key elements (objective and rationale of conducting the mela /material required for the mela, when and how to conduct the mela)<br><br>**Small groups**<br>● Provide the report card in small groups and ask the participants to first discuss the domain-wise activities, process and the material required domain-wise on which a child is being observed during the mela.<br>● One group to demonstrate the mela in big group
**Classroom arrangement**	Big group, small groups
**Material Required**	Report card, PPT slides 97-99, Video on [School Readiness Mela]

**4.2 Different Strategies for Parental Involvement: Formation of mother’s groups, Parents’ meetings, Sharing idea cards**	
**Objective**	Understand as well as use different strategies to mobilize different stakeholders
**Total Duration**	20 minutes
**Process of Facilitation**	**Big group**<br>● Facilitator to discuss the mother group formation, frequency of their meeting, and conducting parent meetings.<br>● Discuss the points written on the PPT.<br><br>**Small groups**<br>● Prepare a role play on mother engagement (inviting mothers in classrooms, doing activities with them, and explaining activities for the next week to them).<br><br>**Big group**<br>● Big group demo by one selected group<br>● Feedback on the demo
**Classroom arrangement**	Big group
**Material Required**	PPT slides 100 and 101, material needed for role play

Let's play – with household items (Maths)

Objective To make children recognise numbers and basic shapes using household items.

Count with Utensils

- Take any kind of utensils. Example – Spoons.
- Tell the children to count the utensils given.
- Ask them to write down what they have counted.

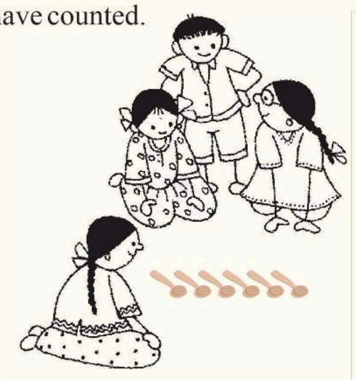

Find out and say

- Have a conversation with the children.
- Tell them a name of a colour and then show them.
- Then tell the children to look for clothes of the same colour in the house.

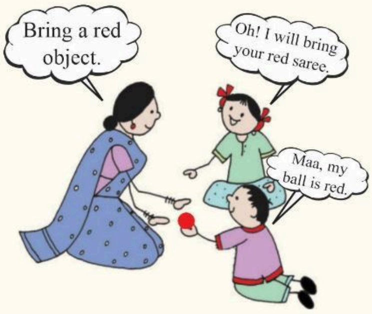

Circle, Square, Triangle

- Show objects available in the house such as- lunch box, bowl, plate, kite, bed, wall, bangle, triangular paratha, etc. Now ask the child about the shape of the object.
- Now ask the child to draw similar shapes using a pencil or chalk.

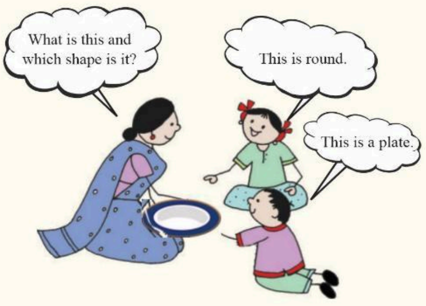

Count how many

- Ask the child to count and bring the objects present in the house. For example, "Bring a few potatoes from the kitchen".
- Count how many potatoes are there.
- The mother can ask the child to count the utensils, clothes, toys, vegetables, etc. available in the house in a similar manner.

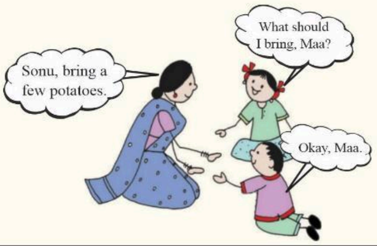

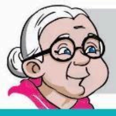

Small but important note: These activities are a few examples. Mothers can try and think of other activities and do them at home.

Creative playing

Objective To develop fine motor skills in children

Impression

- Collect pieces of lady's finger, onion, potato etc.
- Tell the child to make an impression of the pieces on paper.
- Ask them to make any kind of picture by stamping, For example – Trees, Butterflies etc

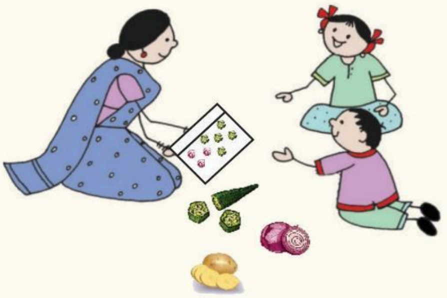

Hand printing

- With some soil or haldi , make a paste in a container
- Ask the child to put handprints on the newspaper or any paper with paste.

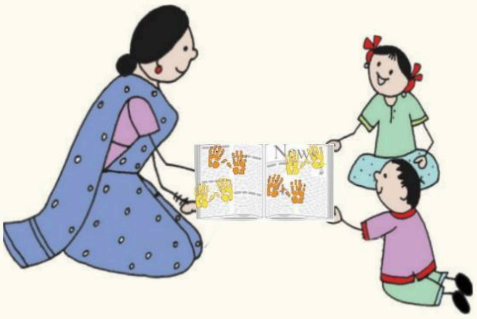

Objective To make children recognise numbers and basic shapes using household items.

Classifying

- Combine two or three types of pulses.
- Ask the child to separate different types of pulses.

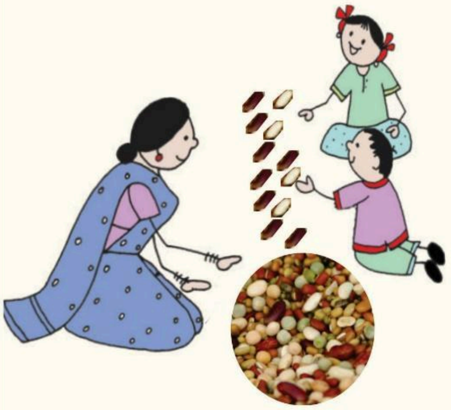

Grouping by colour

- Ask the child to collect things based on their colour
- Examples:
- Yellow- Mango, lemon, banana, clothes etc.
- Green – Okra, Capsicum, Cucumber, Bangles, Clothes.

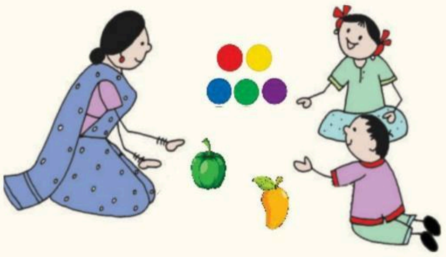

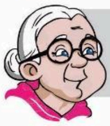

Small but important note: These activities are a few examples. Mothers can try and think of other activities and do them at home.

Some Other Activities

Objective Cognitive and emotional development of the child

Decorating the Kitchen

Ask the child to decorate the kitchen with toys.
If a kitchen set is not available in the house then decorate the kitchen with the utensils in the kitchen.
The parent should tell about the uses of different types of utensils to the child.

Role Play

- Objective of role play:
- 1. Role play on any subject makes the children emotionally attached to that subject and they develop creativity, reasoning ability and helps increase their confidence as well.
- 2. The ability to work together in a group increases.
- 3. You can see a change in the child's behaviour as well.
- How can mothers conduct role play?
- Involve all the members present in the house in the role play. Make sure that everyone participates during the role play.
- Let the child choose the character they want to be and have a discussion about the dialogue.
- Then practice speaking and presenting those dialogues.

Note: You can use masks based on the character. Also, you can wear clothes based on the character as well.

Use of 'Sorry' and 'Please'

- Aman(to Didi): Didi, give me your pen.
- Mummy(to Aman): Aman, whenever you ask for anything from anyone, say "Please".
- Aman(to Didi): Didi, please give me your pen.

Didi (to Mother): Mother, Aman broke my pen.
Mother (to Aman): Aman, whenever someone is harmed by mistake, we should say "Sorry" to them.
Aman (to Didi): Sorry Didi.

Note: Similarly, practice some other easy greetings like – Good morning, Good afternoon, Good evening, Good night etc.

Teach this to children as well

- Exercising regularly
- Washing hands
- Brushing twice a day
- Shower everyday
- Keeping nails short
- Share when eating and playing
- To help others
- Organize your things
- Wait your turn

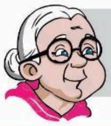

Small but important note: These activities are a few examples. Mothers can try and think of other activities and do them at home.

Day 4: Session 5
**Assessments and Post Quiz**

The fifth session of the fourth day is divided into two main sections. The total duration of this session is 1 hour 20 minutes. This session will cover the topics mentioned below:

5.1 Assessments in Early Grades
5.2 Assessments and Policy
5.3 Understanding and Communication Learning Levels

**5.1 Assessments in Early Grades**	
**Objective**	Understand the meaning and importance of assessments in early grades
**Duration**	20 minutes
**Method**	In a big group
**Process for facilitation**	Mind Mapping in Big group<br><br>1. Facilitator will ask the participants to "write a word on what comes to your mind about assessment" in their notebooks.<br>2. Facilitator to write 5 - 7 words on the whiteboard after taking the inputs from participants.<br>3. Facilitator to ask the participants 'what is the meaning of assessments?'<br><br>Video showcasing and discussion.<br>1. Facilitator to show the UNICEF video and PPT on Assessment.<br>2. Facilitator will summarize the assessment and purpose of the assessment. (Slide 4, 5 and 6)
**Material Required**	UNICEF<br>Module 13A Video 4 Assessment PPT

5.2 Assessments and Policy	
**Objective**	Understand to link concepts such as 'competencies' and 'learning outcomes' with policy and to be able to understand how they are relevant to assessments
**Duration**	20 minutes
**Method**	big group
**Process for facilitation**	Small group<br>1. Facilitator will start the session by explaining assessments as per NCF for Foundational Stage (Slide 8)<br>2. Facilitator will go on to explain the concepts of Curricular Goals, Competencies and Learning Outcomes (Slide 9, 10)<br>3. Facilitator will close the discussion by concluding the summary points mentioned on PPT
**Material Required**	Assessment PPT

5.3 Understanding and Communication of Learning Levels	
**Objective**	Understand the learning levels and how to communicate child's levels to parents and interpret it
**Duration**	30 minutes
**Method**	Small group
**Process for facilitation**	**Small group**<br>• Facilitator to discuss the importance of understanding and communication of learning levels with the participants.<br>• Facilitator to create new 4 groups. Facilitator to distribute the case studies & data format to the groups and each group to prepare a 5-minute role play on it/prepare a presentation on it. Gove 10 minutes for this exercise.<br>• Each group will come and present their case studies (presentation or a role play).<br>• Facilitator to summarize the session by discussing 'do's and don'ts' of the communication of learning levels

**Material Required**	Set of child report card and formats, case studies printouts

**5.4 Post Training Quiz**	
**Objective**	Assessing participants' understanding of the content to be covered in the 6-day session
**Total Duration**	10 minutes
**Process of Facilitation**	• Facilitator to give the following instructions:<br>-We will open the link shared on the WhatsApp group formed in Session 1.<br>-We will attempt the quiz by ourselves, without discussing it with anyone.<br>• Co-facilitator will share the link on the WhatsApp group as well as pull out the link slide.<br>• Ask participants to open and complete the quiz in the given time.
**Classroom Arrangement**	Big group
**Material**	PPT slide 102, quiz link

**Day 4: Reflection**	
**Total Duration**	30 mins
**Process of Facilitation**	• Facilitator will ask "What is one thing that I appreciate about any one person in this group?"<br>• Facilitator to allocate the sessions for Day 5 demos to each group
**Classroom arrangement**	Big group
**Material Required**	PPT slide 103
