# Supplementary material for: Support To Rural India’s Public Education System (STRIPES2) and impact on numeracy and literacy scores: A cluster randomized trial in rural villages of Madhya Pradesh, India
Source: PLoS One. 2025 Sep 12;20(9):e0330203. doi: 10.1371/journal.pone.0330203 (PMC12431668; doi:10.1371/journal.pone.0330203)
Supplement: S4 Appendix — (PDF) [file pone.0330203.s004.pdf]

Appendix 4 - Midline Assessment

General instructions

It is particularly important to be in the right frame of mind while assessing children. We are not going to the village/household to conduct any examination. We want to find out what children can do comfortably in terms of reading and numeracy. Therefore, it is important that you follow the guidelines given below while assessing children:

- **Relaxed environment for the child:** Establish a relaxed environment by having a friendly conversation with the child before you start assessing the child. For example, ask the child about her favourite game/sport, food, friend, festival, story, song; whether she has been to a fair and what did she enjoy the most in it, etc. When you feel that the child is comfortable, show her the tool and tell her that the tool has simple activities you would like her to participate in and that it is not an exam or test. Make sure that you and the child are seated at the same level, i.e., if you are sitting on a chair, then the child should also be seated on a chair. Try not to administer the assessment process while standing.
- **No pressure on the child from others:** Often family members and neighbours gather around to watch how the child is performing. This can make the child nervous. The surveyor should make sure this does not happen.
- **Encouragement and patience with the child:** Encourage the child by appreciating the effort she is making. Be patient with her while she is reading or solving numeracy problems. Give the child ample time to read, think and solve. Do not hurry her.

Ensure that you are following all COVID-19 precautionary measures. It is important to follow all guidelines related to COVID-19 when you are interacting with the people around you and testing the child. Maintain social distancing (a minimum of 6 feet distance) from others and wear a mask always covering your nose and mouth. Sanitise your hands every time you enter or leave a household. However, be sensitive while doing this – it should not offend the people around you. Explain to them that you are following these guidelines to ensure both your own and their safety.

Rules to be followed during the assessment:

1. All children will be assessed on reading and numeracy. We know that younger children will not be able to read much or solve numeracy problems but still follow the same process for all children to keep the process uniform.
2. Only one child should be assessed at a time.
3. The assessment booklet should be in the hands of the child throughout the assessment process.
4. Ensure that you use the same sample for the reading and numeracy assessment for a child.
5. For both reading and numeracy assessment, ensure that you mark the highest level of the child.
6. It is essential that every child's assent is taken before beginning the assessment process. Take the child's consent by following the process given below. Read slowly and clearly only what is written in the box.

- Good morning. My name is ________ and I live in _________ .
- We are going to play some reading and numeracy games. I am going to ask you to read out aloud some letters, words, and stories. I will also ask you to solve some simple Math questions.
- This is not a test, and it will not affect your grade at school.
- You do not have to participate if you do not wish to.
- Do you want to participate? Are you ready to get started?

Midline Reading Assessment

How to assess reading

Level	Assessment instructions	Grading instructions
**PARAGRAPH**	**START THE READING ASSESSMENT AT THE PARAGRAPH LEVEL**<br><br>Ask the child to read the paragraph. Listen carefully to how she reads.	The child is at the **'Paragraph Level'** if the child:<br>• Reads the paragraph like she is reading sentences, rather than a string of words.<br>• Reads the paragraph fluently and with ease, even if she is reading slowly.<br>• Reads the full paragraph with 3 or less than 3 mistakes.<br>*If the child reads the same word incorrectly more than once, it will be considered as only one mistake*<br><br>**If the child can read the paragraph, then ask her to read the story.**<br><br>**If the child is not at 'Paragraph Level' then ask her to read words.**
**STORY**	Ask the child to read the story. Listen carefully to how she reads.	The child is at the **'Story Level'** if the child:<br>• Reads the story like she is reading sentences, rather than a string of words.<br>• Reads the story fluently and with ease, even if she is reading slowly.<br>• Reads the full story with 3 or less than 3 mistakes.<br>*If the child reads the same word incorrectly more than once, it will be considered as only one mistake.*<br><br>**If the child can read the story, then mark her at 'Story Level'.**<br><br>**If the child is not at 'Story Level' then mark her at 'Paragraph Level'.**
**WORDS**	Ask the child to read words from the list. If the child does not start reading, then point out words one by one for her to read.	The child is at the **'World Level'** if the child reads at least 4 out of the 5 words correctly.<br><br>**If the child is at 'Word Level', then ask her to try to read the paragraph again and then follow the instructions for paragraph level testing.**<br><br>**If the child can correctly read at least 4 out of 5 words but is still struggling with the paragraph, then mark her at 'Word Level'.**<br><br>**If the child is not at 'Word Level' (cannot correctly read at least 4 out of 5 words), then ask her to read letters.**

**LETTERS**	Ask the child to read letters from the list. If the child does not start reading, then point out letters one by one for her to read.	The child is at the **'Letter Level'** if the child reads at least 4 out of the 5 letters correctly.<br><br>**If the child is at 'Letter Level', then ask her to try to read the words again and then follow the instructions for word level testing.**<br><br>**If the child can correctly read at least 4 out of 5 letters but is still struggling with the words, then mark the child at 'Letter Level'.**<br><br>**If the child is not at 'Letter Level' (cannot correctly read at least 4 out of 5 letters), then mark the child at 'Beginner Level'.**

MARK THE CHILD ONLY AT THE HIGHEST LEVEL SHE CAN REACH IN THE READING ASSESSMENT.

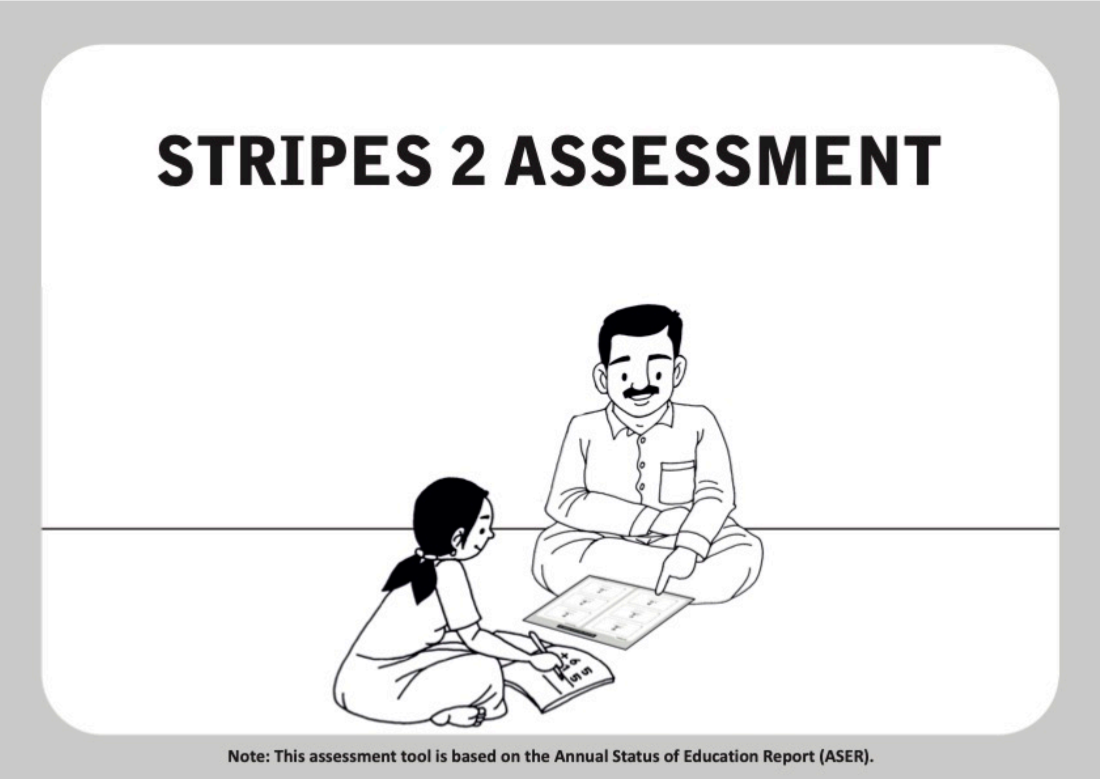

Reading Assessment SAMPLE (1)

Paragraph

रोहन के पास एक हाथी है।
हाथी की सूंड लम्बी है।
वह फल खाता है।
रोज़ सबको खेल दिखाता है।

Story

राजू और सीमा मित्र थे। एक दिन वे दोनों नदी के किनारे खेल रहे थे। खेलते-खेलते अचानक राजू का पैर फिसल गया। वह नदी में गिर गया। सीमा जल्दी से एक रस्सी लाई और उसे राजू की तरफ फेंका। राजू ने रस्सी को ज़ोर से पकड़ा। सीमा ने उसे खींचकर बाहर निकाल लिया। फिर वे अपने घर लौट गए।

①

Reading Assessment SAMPLE (1)

Words

हाथी खुश

बैल

मौसी जेब

Letters

च र

प

न ग

②

Midline Numeracy Assessment

How to assess numeracy

Level	Assessment instructions	Grading instructions
**ADDITION**	**START THE NUMERACY ASSESSMENT AT THE ADDITION LEVEL**<br><br>The child is required to solve 2 addition problems correctly. Show the child the first 2 addition problems one-by-one and ask her to solve the problems. Even if the first addition problem is solved incorrectly, ask the child to solve the second addition problem.<br><br>**If the child solves first 2 addition problems correctly, then the child is at 'Addition Level'.**<br><br>**If the child solves both the problems incorrectly, then the child is not at 'Addition Level'.**<br><br>**If the child solves 1 problem correctly and 1 problem incorrectly, then ask the child to solve the third addition problem.**	The child is at **'Addition Level'** if the child solves 2 addition problems correctly. **Now ask the child to solve subtraction problems and follow the instructions for subtraction level testing.**<br><br>**If the child cannot do 2 addition problems correctly, then ask the child to recognize numbers from 10-99.**
**SUBTRACTION**	The child is required to solve 2 subtraction problems correctly. Show the child the first 2 subtraction problems one-by-one and ask her to solve the problems. Even if the first subtraction problem is solved incorrectly, ask the child to solve the second subtraction problem.<br><br>**If the child solves first 2 subtraction problems correctly, then the child is at 'Subtraction Level'.**<br><br>**If the child solves both the problems incorrectly, then the child is not at 'Subtraction Level'.**<br><br>**If the child solves 1 problem correct and 1 problem incorrectly, then ask the child to solve the third subtraction problem.**	Mark the child at **'Subtraction Level'** if the child solves 2 subtraction problems correctly.<br><br>**If the child cannot do 2 subtraction problems correctly, then mark the child at 'Addition Level'.**
**NUMBER RECOGNITION (10-99)**	Ask the child to recognize numbers 10-99 from the list. If the child does not start recognizing, then point out numbers one by one for her to identify.	Mark the child at the **'Number Recognition (10-99) Level'** if the child recognizes at least 4 out of the 5 numbers correctly.

		**If the child is not at 'Number Recognition (10-99) Level' (cannot correctly recognize at least 4 out of 5 numbers), then ask her to recognize numbers from 1-9.**
**NUMBER RECOGNITION (1-9)**	Ask the child to recognize numbers 1-9 from the list. If the child does not start recognizing, then point out numbers one by one for her to identify.	Mark the child at the **'Number Recognition (1-9) Level'** if the child recognizes at least 4 out of the 5 numbers correctly.<br><br>**If the child is not at 'Number Recognition (1-9) Level' (cannot correctly recognize at least 4 out of 5 numbers), then mark the child at the 'Beginner Level'.**

MARK THE CHILD ONLY AT THE HIGHEST LEVEL SHE CAN REACH IN THE NUMERACY ASSESSMENT.

Numeracy Assessment SAMPLE (1)

Addition	Subtraction
62 + 29	64 − 17
56 + 17	78 − 49
36 + 25	32 − 15

3

Numeracy Assessment SAMPLE (1)

Numbers (10-99)

18 79

60

93 35

Numbers (1-9)

5 1

6

3 9

4
