# Supplementary material for: Support To Rural India’s Public Education System (STRIPES2) and impact on numeracy and literacy scores: A cluster randomized trial in rural villages of Madhya Pradesh, India
Source: PLoS One. 2025 Sep 12;20(9):e0330203. doi: 10.1371/journal.pone.0330203 (PMC12431668; doi:10.1371/journal.pone.0330203)
Supplement: S5 Appendix — (PDF) [file pone.0330203.s005.pdf]

Appendix 5 - EGRA and EGMA tests

EGRA test - English version

General Instructions

It is important to establish a playful and relaxed relationship with the child that will be assessed through an initial talk on topics of interest to the child (see example below). The child should perceive the assessment more as a game rather than an evaluation. It is important that you do not deviate from the guidelines and ONLY read aloud the text in **bold**, slowly and clearly, so that the child can understand the exercises.

If the child does not respond to any of the following questions, do not force, remain friendly, and continue with the exercise.<br>**Good morning/afternoon. My name is ________ and I work for Effective Intervention. And you, what's your name?** [wait until the child responds] **Who do you have in your family?** [wait until the child responds] **Very nice**<br><br>**How is your family?** [wait until the child responds] **Good!**<br>**When I am not at work, I like to ___________. And you? What do you most enjoy doing when you are not at school?** [wait until the child responds] **Very nice**<br>**When I was a kid, I used to play with my friends during summer holidays. What did you do during summer holidays?** [wait until the child responds] **Very nice**

Verbal Consent

- **Let me tell you why I am here today. I am working with a project of Effective Intervention. We came today to your school to do an exercise to help us better understand how children learn how to read and do mathematics, and you were chosen to help us.**
- **We would like to ask for your help. But you do not have to take part if you do not want to.**
- **We are going to do some reading and mathematics exercise. I am going to ask you to read letters, words and a short story out loud. Then you will go to my friend/colleague sitting at the other side** (point to the direction of the EGMA enumerator)**, and he/she will ask you to identify numbers, do some calculations and solve a few problems.**
- **Sometimes I will use a timer to time how long it takes you to complete some of the tasks.**
- **This is NOT a test and it will not affect your grade at school.**
- **Can we start?** [wait until the child responds]

If the oral consent is obtained, please tick: ☐
If the oral consent is not obtained, please make a note on the student list. ☐

Subtask 1. Letter Sound Identification	Page 1	60

Here is a page with many Hindi letters. Please read as many letters as you can.

For example, [Point to "__"] **this letter is "__".**

Let's practice. [Point to "__"] **Tell me what letter this is.**
✓ [If the child read "__", say] **Very good, this letter is "__".**
× [If the child did not read "__", say] **This letter is "__".**

[Point to "__"]. **Now let's try another one. Tell me what letter this is.**
✓ [If the child read "__", say] **Very good, this letter is ."___"**
× [If the child did not read "__", say] **This letter is "___".**

Have you understood? [wait until the child replies]

When I say "start", start here [point to the first letter]**, and read through the page** [sweep finger across first line]. **I will use this timer and will tell you when to stop. Point to each letter and read out loud the letter. Read as fast and the best you can. If there is a letter you can't read, move to the next one.**

Put your finger on the first letter [make sure the child does so]. **Are you ready?** [wait until the child responds] **You can start.**

(/) Mark any incorrect words with a slash (/).
(ø) Mark self-correction as correct.
(⅃) Mark the final word read with a bracket (⅃).

Examples: : **क ट ब**

1	2	3	4	5	6	7	8	9	10	
ग	ह	न	क	र	म	स	ल	अ	व	(10)
उ	त	च	घ	ड	ई	ख	ब	ए	क	(20)
र	द	म	श	य	फ	आ	क	ह	**अं**	(30)
अ	ब	ह	स	न	क	**ज्ञ**	प	ए	र	(40)
भ	स	क	ट	र	ओ	ल	च	म	ग	(50)
ठ	ढ़	र	स	थ	उ	च	त	न	ल	(60)
न	र	च	प	क	त	**त्र**	ड़	ह	ध	(70)
ऊ	द	क	ड	म	ह	ड़	ज	र	य	(80)
व	थ	छ	प	ह	न	थ	झ	य	च	(90)
ब	ष	औ	र	ण	ज	क	फ	ढ़	ड	(100)

Start the timer when the child reads the first letter. Stop the timer when the child reads the last letter.

If the child hesitates for 3 seconds, point to the next letter and say "Continue". Mark the letter you read as incorrect.

When the timer reaches 0, say "stop."

If the child does not provide a single correct response on the first line, say "Thank you!", discontinue this subtask, check the box at the bottom, and go on to the next subtask.

Thank you, let's move to the next exercise.

Subtask 2: Initial Sound Discrimination	🕮 ✗	⏱ ✗

In this exercise, you will listen to the Hindi words that I read. I will read three words and one of them starts with a different sound. I will read TWICE. Tell me which one starts with a different sound.**

For example:
"____", "____", "____"; "___", "___", "___" **which one starts with a different sound?**
✓ [If the child answered "____", say] **Very good, "____" starts with a different sound.**
✗ [If the child did not answer "hot", say] "___", "___", "___". **"____" starts with a different sound than "cat" and "car".**

Now let's try again:
"____", "____", "____"; "_____", "____", "____", which one starts with a different sound?
✓ [If the child answered "____", say] **Very good, "_____" starts with a different sound.**
✗ [If the child did not answer "____", say] **"____", "____", "____". "____" starts with a different sound than "_____" and "______".**

Did you understand? [wait until the child responds] **Are you ready?** [wait until the child responds] **Let's start.**

✍ (✓) 1 = Correct
(✓) 0 = Incorrect
(✓) . = No answer

☞ If the child does not provide an answer in the first 5 items, say "Thank you!", discontinue this subtask, check the box at the bottom, and go on to the next subtask.

➲ If the child hesitates for 5 seconds, provide the answer. Mark the item that you provided answer as "no response".

	…… which one starts with a different sound?			Correct answer	Correct	Incorrect	No response
1	कल	दिन	कम	दिन	1	0	.
2	पत्ती	सब्ज़ी	सफेद	पत्ती	1	0	.
3	गरम	गगन	शाम	शाम	1	0	.
4	खाओ	नाम	खेलो	नाम	1	0	.
5	महक	बड़े	बहुत	महक	1	0	.
6	आठ	जल्दी	आप	जल्दी	1	0	.
7	बिल्ली	पपीता	पीली	बिल्ली	1	0	.
8	नहीं	अगस्त	नमक	अगस्त	1	0	.
9	हल	हफ़्ता	छोटा	छोटा	1	0	.
10	ठीक	किताब	ठंडा	किताब	1	0	.

Thank you, let's move to the next exercise.

Subtask 3. Nonword Reading	🕮 Page 2	⏱ 60

In this sheet there are some made-up words. Read as many words as you can.

For example: [Point to the word "_____"], **this made up word is "_____"**.

Let's practice. [Point to the word "_____"] **Read this word.**

✓ [If the child answered "____", say] **Very good, this made up word is "_____".**
× [If the child did not answer "____", say] **This made up word is "____".**

[Point to the word "mab"] **Now let's try another one. Read this word.**

✓ [If the child answered "mab", say] **Very good, this made up word is "mab"**.
× [If the child did not answer "mab", say] **This made up word is "mab".**

When I say "start", start here [point to the first word]**, and read through the page** [sweep finger across first line]. **I will use this timer and will tell you when to stop. Point to each word and read out loud. Read as fast and the best you can. If there is one word you can't read, move to the next one. Put your finger in the first word [**make sure the child does so]**. Are you ready?** [wait until the child responds] **You can start.**

Start the timer when the child reads the first word. Stop the timer when the child reads the last word.

➲ If the child hesitates for 3 seconds, point to the next word and say "Continue". Mark the word that you provided as incorrect.

✋ When the timer reaches 0, say "stop."

✍ (/) Mark any incorrect words with a slash (/).
(ø) Mark self-corrections as correct.
(⅃) Mark the final word read with a bracket (⅃).

✋ If the child does not provide a single correct response in the first line (5 words), say "Thank you!" and discontinue this subtask.

Examples: **द्प** **डिफ** **मब**

1	2	3	4	5	
नब	चम	पक	कज	कजी	(5)
टब	कूम	नक	लाप	कोहा	(10)
आबा	मस	हेल	सग	रोप	(15)
हुबा	अलक	केसा	दरख	ताजक	(20)
टकप	समल	मतक	कंजप	टेसा	(25)
अनप	ओचला	गरग	साबी	हज्ञ	(30)
ठकच	कमेला	तहद	सदस	बेदाका	(35)
पलच	सेकम	औजकी	अचपक	अंकप	(40)
नखेद	सदेभ	यकम	पनक	हवला	(45)
चपक	दमक	लमस	डीघू	मचद	(50)

Thank you, let's move to the next task.

Subtask 4. Familiar Word Reading	🕮 Page 3	⏱ 60 सेकेंड

In this sheet, there are some Hindi words. Read as many words as you can. Do not spell the words, but read them.

For example, [Point to the word “____”] **this word is “___”.**

Let’s practice. [Point to the word “____”]. **Read this word.**

✓ [If the child answered “____”, say] **Very good, the word is “____”.**
✗ [If the child did not answer ”____”, say] **This word is “____”.**

Now let’s try another one. [Point to the word “____”]

✓ [If the child answered ”top”, say] **Very good, the word is “top”.**
✗ [If the child did not answer ”top”, say] **This word is “top”.**

When I say “start”, start here [point to the first word]**, and read through the page** [sweep finger across first line]. **I will use this timer and will tell you when to stop. Point to each word and read out loud. Read as fast and the best you can. If there is one word you can’t read, move to the next one. Put your finger on the first word [**make sure the child does so]. **Are you ready? You can start.**

✍ (/) Mark any incorrect words with a slash (/).
(ø) Mark self-corrections as incorrect.
(⅃) Mark the final word read with a bracket (⅃).

Example: **टोपी** **हाथी** **हरा**

1	2	3	4	5	
जल	दिन	तीन	बड़ी	हम	(5)
बस	गाय	रुको	रोना	नल	(10)
लंबा	अच्छा	लोग	यहाँ	उनकी	(15)
लाओ	मेरे	होने	बीच	बड़े	(20)
प्रति	साथ	मैंने	हाथ	वर्ष	(25)
सुबह	कब	खेलो	समय	खाना	(30)
पीना	तरह	रात	सभी	दूसरे	(35)
क्यों	घर	आज	मुझे	भाई	(40)
अभ्यास	लिये	इसलिए	अपने	चम्मच	(45)
आसान	सुंदर	हँसना	मीठा	छोटा	(50)

Start the timer when the child reads the first word. Stop the timer when the child reads the last word.

➲ If the child hesitates for 3 seconds, point to the next word and say “Continue”. Mark the word that you provided as incorrect.

✋ When the timer reaches 0, say “stop.”

✋ if the child does not provide a single correct response on the first line (5 words), say “Thank you!”, discontinue this subtask, and go on to the next su

b task.

Thank you, let’s move to the next task.

Subtask 5a: Passage Reading

Page 5.

Show to the children the page of the stimulus booklet while you read the instructions.

- **Here is a short story. I would like that you read this story aloud, quickly but carefully. I will use this timer and will tell you when to begin and when to stop. If there is a word that you cannot read, go to the next one. When you finish, I will ask you some questions about the story. Ready?** [wait until the child replies] **You can start.**

(/) Mark any incorrect words with a slash (/).
(ø) Mark self-corrections as correct.
(]) Mark the final word read with a bracket (]).

It was a windy day.	8
A girl called Aarti was flying a red kite.	20
The kite got stuck high in a tree and she could not reach the kite. She was sad.	35
Suddenly, a big bird flew towards her. Aarti was scared	45
but the bird dropped the kite at her feet, then flew away. “Thank you, big bird!” she shouted.	62

60

Start the timer when the child reads the first word.

If the child hesitates or stops more than 3 seconds on a word, move to the next word and say “Continue”.

When the timer reaches 0, say “stop.”

If the child does not read any word correctly before the boxed word [____], move to the next task.

If the child says “I don’t know”, mark incorrect.

Subtask 5b: Reading Comprehension

When the child finishes reading, <u>REMOVE the passage from the child’s view and ask the first question</u>.

Ask the child only the questions related to the text read. The child should have read the part of the text that corresponds to the question. If a child does not give an answer after 10 seconds, mark “no response” and move to the next question. Do not repeat the questions. Consider all sensible answers the child provides as correct.

- **Now I am going to ask you about the story you just read. Answer the questions the best you can.**

Questions [Answers]	Correct	Incorrect	No response
**What was the weather like?** [windy, a lot of wind]	1	0	.
**What colour was Aarti’s kite?** [Red]	1	0	.
**Why was Aarti sad?** [Because the kite got stuck in a tree/she could not reach the kite.]	1	0	.
**Why was Aarti scared?** [Because of the bird; the bird flew towards her]	1	0	.
**Why did Aarti thank the bird?** [Because the bird helped her/brought back the kite; she was grateful; she had her kite back]	1	0	.

Thank you, let’s move to the next task.

Subtask 6. Listening comprehension				
I am going to read you a short story aloud ONCE and then ask you some questions. Please listen carefully and answer the questions as best as you can. You can answer the questions in whichever language you prefer. Ready? [wait until the child responds] Let's start.				Remove the passage from the child's view.<br>Do not allow the child to look at the passage or the questions.<br>If a child says "I don't know", mark as incorrect.
**At the market, Raju bought spinach and bananas. He put them in his basket. Later at home, Raju was surprised that the bananas were gone and under the spinach was a small, fat, happy monkey!**				
	Correct	Incorrect	No response	
**Where did Raju do his shopping?**<br>[At the market; market]	1	0	.	
**What did Raju buy (***name one thing that Raju buy***)?**<br>[banana, spinach, banana and spinach]	1	0	.	
**Why was the monkey fat and happy?**<br>[It had eaten the bananas]	1	0	.	

Thank you for doing this exercise with me. Now you can __________________.

EGMA test - English version

General Instructions

It is important to establish a playful and relaxed relationship with the child through an initial talk on topics of interest to the child (follow the text in bold below). The child should perceive the assessment more as a game rather than an evaluation. It is important that you ONLY read aloud the text in **bold**, slowly and clearly, so that the child can understand the exercises.

Good morning. My name is ________. And you, what's your name?

In my free time, I like to __________. And you, what do you like to do? [wait until the child responds] **Very nice.**

I like to eat samosas. Samosa tastes even better when it is crispy. And you, what do you like to eat? [wait until the child responds]

Now that you have done some reading games with my colleague, let's do some Maths games. Are you ready? [wait until the child responds] **Let's start.**

Subtask 1. Number identification	🕮 Number sheet 1	⏱ 60 seconds
**On this sheet there are some numbers. When I say "start", start here** [point to the first number]**, and read through the page** [sweep finger across first line].<br><br>**Point to each number and read out loud. I will use this timer and will tell you when to stop. Read as fast and the best you can.**<br><br>**If there is one number you can't read, move to the next one.**<br><br>**Put your finger in the first one** [make sure the child does so]**. Are you ready?** [wait until the child replies]<br><br>**You can start.**		✋When the timer reaches 0, say "stop." | <br>➲If the child hesitates for 5 seconds, point to the next number and say "Go on". Mark the number that you provided as incorrect.
✍(/) = Mark any incorrect number or no response with a slash (/).<br>(⅃) =Mark the final number read with a bracket (⅃).		

**3**	**9**	**0**	**11**	**20**
**33**	**45**	**29**	**37**	**48**
**91**	**66**	**74**	**87**	**54**
**108**	**246**	**587**	**751**	**985**

Thank you, let's move to the next task.

Subtask 2. Number Discrimination (PRACTICE)	Number sheet 2	⏱ ✗
**Look at these numbers. Say which number is bigger** [the child can only be considered correct if he/she "says" the bigger number, pointing is not enough].<br><br>**8 4**<br>✓ **[**If the child answered 8, say] **Well done, 8 is bigger. Let's try another example.**<br><br>✗ [If the child did not answer 8, say] **The bigger number is 8.** [Point to 8] **This is 8.** [Point to 4] **This is 4. 8 is bigger than 4. Let's try another example.**		
**Look at these numbers. Say which number is bigger.**<br>**10 12**<br>✓ [If the child answered 12, say] **Well done, 12 is bigger. Let's continue.**<br><br>✗ [If the child did not answer 12, say] **The bigger number is 12.** [Point to 10] **This is 10.** [Point to 12] **This is 12. 12 is bigger than 10. Let's continue**.		

Subtask 2. Number discrimination (TEST)	Number sheet 3	

Look at these numbers. Say which number is bigger. [repeat for each item]

(✓) 1 = Correct
(✓) 0 = Incorrect or without answer

1	7	5	7		1	0
2	11	24	24		1	0
3	47	34	47		1	0
4	49	56	56		1	0
5	75	73	75		1	0
6	94	78	94		1	0
7	146	153	153		1	0
8	287	534	534		1	0
9	643	634	643		1	0
10	867	965	965		1	0

If the child makes 4 successive errors at any point, say "thank you", discontinue this subtask, mark below and move to the next subtask.

➲If the child hesitates for 5 seconds, point to the next item and say "Go on". Mark the item that you provided the answer for as incorrect.

|

Thank you, let's move to the next task.

<table>
<tr><td>Subtask3. Missing Number (PRACTICE)</td><td>Number sheet 4</td><td></td></tr>
<tr><td colspan="2">
P1 Here are some numbers. 1, 2 and 4, what number goes here [point to the empty box]?
<table><tr><td>1</td><td>2</td><td>(3)</td><td>4</td></tr></table>
✓ [If the child answered 3, say] Well done, it’s 3. Let’s do another one.
✗ [If the child did not answer 3, say] The number 3 goes here. Say the numbers with me [point to each number]. 1, 2, 3 and 4. 3 goes here. Let’s try another one.
P2 Here are some numbers. 5, 10 and 15, what number goes here?
<table><tr><td>5</td><td>10</td><td>15</td><td>(20)</td></tr></table>
✓ [If the child answered 20, say] Well done, it’s 20. Let’s continue.
✗ [If the child did not answer 20, say], The number 20 goes here. Say the numbers with me [point to each number]. 5, 10, 15 and 20. 20 goes here. Let’s continue.
</td><td></td></tr>
</table>

Subtask 3. Missing Number (TEST)	Number sheet 5 and 6	

Here are some numbers [point to the box]**. What number goes here?** [repeat for each item]

(✓) 1 = Correct
(✓) 0 = Incorrect or without answer

If the child makes 4 successive errors at any point, say "thank you", discontinue this subtask, mark below and move to the next subtask.

➲If the child hesitates for 5 seconds, point to the next item and say "Go on". Mark the item that you provided the answer for as incorrect.

Item					Score	
1.	4	5	6	(7)	1	0
2.	13	14	(15)	16	1	0
3.	20	(30)	40	50	1	0
4.	(500)	600	700	800	1	0
5.	2	4	6	(8)	1	0
6.	478	479	(480)	481	1	0
7.	26	(24)	22	20	1	0
8.	60	65	(70)	75	1	0
9.	850	840	830	(820)	1	0
10.	3	8	(13)	18	1	0

Thank you, let's move to the next task.

Subtask 4a. Addition (level 1)	Number sheet 7 and 8	60 seconds

Paper and pencil

In these two pages there are some addition questions [glide hand from top to bottom on the two pages].

I will use the timer and will tell you when to stop.

Say the answer for each question. If you don't know an answer, move to the next question.

If you want, you can use this paper and pencil. Are you ready (wait until the child responds)**? Start here** [point to the first problem].

(✓) 1 = Correct
(✓) 0 = Incorrect or without answer

1	1 + 2 = (3)	1	0		11	7 + 8 = (15)	1	0
2	3 + 2 = (5)	1	0		12	4 + 7 = (11)	1	0
3	6 + 2 = (8)	1	0		13	7 + 6 = (13)	1	0
4	3 + 4 = (7)	1	0		14	9 + 4 = (13)	1	0
5	3 + 3 = (6)	1	0		15	8 + 6 = (14)	1	0
6	8 + 1 = (9)	1	0		16	8 + 8 = (16)	1	0
7	7 + 3 = (10)	1	0		17	9 + 7 = (16)	1	0
8	5 + 7 = (12)	1	0		18	8 + 9 = (17)	1	0
9	2 + 8 = (10)	1	0		19	9 + 2 = (11)	1	0
10	9 + 5 = (14)	1	0		20	8 + 5 = (13)	1	0

The child used:

	Fingers to count.
	Paper and pencil.
	Solved the question in his/her head.

Select all that apply.

When the timer reaches 0, say "stop."

If the child makes 4 successive errors at any point, say "thank you", discontinue this subtask, mark below and move to the next subtask.

➲If the child hesitates for 5 seconds, point to the next item and say "Go on". Mark the item that you provided the answer for as incorrect.

Thank you, let's move to the next task.

Subtask 4b. Addition (level 2)	Number Sheet 9	⏱ ✘

Paper and pencil

Here are some more addition questions [glide hand from top to bottom].

Tell me the answer to each question. If you do not know the answer, move to the next one.
If you want, you may use this paper and pencil. Are you ready? [wait until the child responds] **Start here** [point to the first problem]

(✓) 1 = Correct
(✓) 0 = Incorrect or without answer

1	13 + 6 = (19)	1	0
2	17 + 8 = (25)	1	0
3	14 + 13 = (27)	1	0
4	22 + 37 = (59)	1	0
5	38 + 26 = (64)	1	0
6	246+526= (772)	1	0

The child used:

	Fingers to count.
	Paper and pencil
	Solved the question in his/her head.

Select all answers that apply.

<u>Skip this subtask if the child scores zero in level 1 Addition questions.</u>

If the child makes 4 successive errors, say "thank you", discontinue this subtask, mark below and move to the next subtask.

If the child does not provide answer in 30 seconds, point to the next item and say "Go on". You may give additional 30 second if it looks like the child is still processing the question. Mark the item that you provided the answer for as incorrect.

Thank you, let's move to the next task.

Subtask 5a. Subtraction (level 1)	Number sheet 10 and 11	60 seconds

Paper and pencil

On these two pages there are some subtraction questions [glide hand from top to bottom on the two pages].

I will use the timer and will tell you when to stop.
Say the answer for each question. If you don't know an answer, move to the next question.
If you want, you can use this paper and pencil. Are you ready (wait until the child responds)**? Start here** [point to the first problem].

(✓) 1 = Correct
(✓) 0 = Incorrect or without answer

1	3 - 1 = (2)	1	0		11	15 - 7 = (8)	1	0
2	5 - 2 = (3)	1	0		12	11 - 4 = (7)	1	0
3	8 - 2 = (6)	1	0		13	13 - 7 = (6)	1	0
4	7 - 4 = (3)	1	0		14	13 - 9 = (4)	1	0
5	6 - 3 = (3)	1	0		15	14 - 6 = (8)	1	0
6	9 - 8 = (1)	1	0		16	16 - 8 = (8)	1	0
7	10 - 7 = (3)	1	0		17	16 - 7 = (9)	1	0
8	12 - 5= (7)	1	0		18	17 - 9 = (8)	1	0
9	10 - 2 = (8)	1	0		19	11 - 2 = (9)	1	0
10	14 - 9 = (5)	1	0		20	13 - 8 = (5)	1	0

The child used:

	Fingers to count.
	Paper and pencil.
	Solved the questions in his/her head.

Select all answers that apply.

When the timer reaches 0, say "stop."

If the child makes 4 successive errors at any point, say "thank you", discontinue this subtask, mark below and move to the next subtask.

If the child hesitates for 5 seconds, point to the next item and say "Go on". Mark the item as incorrect.

Thank you, let's move to the next task.

Subtask 5b. Subtraction (level 2)	Number Sheet 12	⏱ ✖
Paper and pencil		Skip this subtask if the child scores zero in level 1 Subtraction questions.

Here are some subtraction questions [glide hand from top to bottom]**.**

Tell me the answer to each subtraction question.

If you do not know an answer, move to the next one. If you want to, you may use this paper and pencil.

Are you ready? [wait until the child replies] **Start here** [point to the first problem]

(✓) 1 = Correct
(✓) 0 = Incorrect or without answer

1	19 - 6 = (13)	1	0
2	25 - 8 = (17)	1	0
3	27 - 14 = (13)	1	0
4	59 - 37 = (22)	1	0
5	64 - 26 = (38)	1	0
6	772 – 526= (246)	1	0

The child used:

	Fingers to count.
	Paper and pencil.
	Solved the questions in his/her head.

Select all answers that apply.

If the child makes 4 successive errors, say "thank you", discontinue this subtask, mark below and move to next task.

➲ If the child uses an inefficient strategy (e.g. tick marks), ask the child **"Do you know another way to solve the problem?"** If they say no and/or continue to use the inefficient strategy, then ask the child to move to the next item after 5 seconds.

Thank you, let's move to the next task.

Subtask 6. Word problems (PRACTICE)	×	×
Counters, paper and pencil.		×
**I am going to read some maths problems for you to solve.**<br><br>**If you want you can use these counters, paper and pencil. Listen carefully to each problem.**<br><br>**If you need, I can repeat once. Are you ready?** [wait until the child replies] **Let's start.**		
**There are 4 children in the classroom** [pause]<br>**1 child gets out of the classroom.** [pause]<br><br>**How many children stay in the classroom?**<br><br>✓ [If the child answers 3, say] **Well done, 3 children stayed in the classroom. Let's continue.**<br><br>× [If the child does not answer 3, Put 4 counters on top of the table and say] **Imagine that these counters are children.**<br><br>**One of the children gets out of the classroom.**<br><br>**Show me the child getting out of the classroom. How many children stayed in the classroom?**<br><br>**Well done, three children stayed in the classroom. Let's continue.**		

Subtask 6. Word Problems (TEST)	×	×
Counters, paper and pencil.		<u>[pause and check] at the end of each sentence</u> to make sure that the child understands what you have said before continuing.<br><br>You can ask "Do you understand?" when in doubt. <u>If the child requests, you may repeat the question ONCE only.</u><br><br>If the child makes 4 successive
**Now I will read some more problems to you.**		
(✓) 1 = Correct<br>(✓) 0 = Incorrect or no response		
Problem 1<br>**There are 3 children in a classroom.**<br>**Another 2 children go inside the classroom.**<br>**How many children are now in the classroom?**	**(5)** 1 | 0	
Problem 2<br>**There are 8 pieces of fruit in a bag.**<br>**2 are apples, the rest are oranges.**<br>**How many oranges are there (in the bag)?**	**(6)** 1 | 0	
Problem 3<br>**There are 4 children in Sunit's class and 7 children in Kamal's class.**	**(3)**	

<table>
<tr><td>How many children must join Sunit's class so there are the same number of children in both classes?</td><td>| 1 | 0 |</td><td rowspan="5">errors, say "thank you", discontinue this subtask and mark below.

➲If the child has worked on the problem for more than 60 seconds and not provided an answer, say "let us try another one" and move on to the next item and mark the item as incorrect.</td></tr>
<tr><td>Problem 4
5 birds land on a tree.
There are now 12 birds in the tree.
How many birds were in the tree to begin with?</td><td>(7)
| 1 | 0 |</td></tr>
<tr><td>Problem 5
5 children share 15 sweets. Each child gets the same number of sweets each.
How many sweets does each child get?</td><td>(3)
| 1 | 0 |</td></tr>
<tr><td>Problem 6
There are 5 tables in a classroom.
At each table there are two children seated.
How many children are in the classroom altogether?</td><td>(10)
| 1 | 0 |</td></tr>
<tr><td colspan="2">The child used:
[ ] Fingers to count.
[ ] Blocks to work
[ ] Paper and pencil.
[ ] Solved the questions in his/her head.
Select all answers that apply.</td></tr>
</table>

Thank you, you did a good job. Now please return to your own classroom/you can go home.
