# Supplementary material for: Support To Rural India’s Public Education System (STRIPES2) and impact on numeracy and literacy scores: A cluster randomized trial in rural villages of Madhya Pradesh, India
Source: PLoS One. 2025 Sep 12;20(9):e0330203. doi: 10.1371/journal.pone.0330203 (PMC12431668; doi:10.1371/journal.pone.0330203)
Supplement: S6 Appendix — (PDF) [file pone.0330203.s006.pdf]

Appendix 6 - STRIPES2 data collection process

GH Training & Consulting, Hyderabad, India, recruited and trained independent teams to collect the STRIPES2 data through various activities, including enumeration, caregiver surveys and child assessment tests. The data collection team was composed by:

- Village enumerators (VE): typically, a resident of the village who identified and mobilised children from the enumerated list for assessments and identified caregivers for the surveys. Usually, one VE was recruited per village.
- Surveyors and test administrators (TA): trained to conduct caregiver surveys and administer midline and endline tests for children. The caregiver surveys were household-level surveys that focused on the child's school enrolment and attendance, grades, engagement at the household level, and related costs.

Enumeration of children for STRIPES 2: The children and caregivers were assigned a unique ID (alphanumeric combination of village, cluster, household, parent and child IDs), which enabled quick identification of the participants at the village level for various data collection activities. GHTC developed operations manuals and translated them into Hindi to train the VEs, surveyors, and TAs to ensure uniformity in data collection.

Midline assessment of children

An independent expert in educational assessments designed tests to measure reading and mathematics skills. The tests were similar to the ASER (Annual Status of Education Report) tests, but adapted to the context of rural Madhya Pradesh. Two sets of language and mathematics questions were prepared and piloted among children from villages not enrolled in STRIPES2. The questions were finalised after modification based on the piloting experience. ASER, unlike EGRA/EGMA, is simpler and has fewer tasks for children. To reduce the potential for the passing of information between children, two sets of questions were used, these being randomly assigned to each child a priori.

GHTC trained TAs to carry out the midline assessment for one week through classroom interactions and mock sessions. Debrief sessions were organised to ensure that TAs adhered to the protocol in the test administration manual.

Implementation: All the enlisted children in each village were tested on a pre-determined day. VEs informed the caregivers in advance and mobilised the children on the test day to a central location in a staggered manner so that children attended the tests at different times, but in such a way that the confidentiality of the testing test was ensured. Both sets of questions were used, with these being randomly assigned to each child a priori, and TAs were not allowed to choose/change the assignment. Irrespective of the number of children in the village, the test was organised for only one day. Therefore, in large villages, multiple teams ensured that the completion of the test was within one day. Midline testing was done from December 2021 to January 2022.

Endline assessment of children

The primary outcome was the arithmetic mean of the child's scores on EGRA (Early Grade Reading Assessment) and EGMA (Early Grade Mathematics Assessment) tests. The National

Foundation of Educational Research (NFER), UK, coordinated with a panel of local experts and primary school teachers in Satna to develop the Hindi versions of EGRA and EGMA and to adapt them to the local context.

An independent consultant and the trial coordinator (Siddharudha Shivalli) were trained in the administration of EGRA and EGMA by NFER and the team for six days. The training focussed on EGRA and EGMA protocols and training TAs in administering EGRA and EGMA using tablets. The independent consultant coordinated with NFER and GHTC to translate the adapted versions of EGRA and EGMA into Hindi.

Validation workshops: GHTC organised a two-day workshop with primary school teachers in Satna, Madhya Pradesh, India, along with NFER, the trial coordinator and an independent consultant. The workshop focused on verifying the Hindi translation, determining if EGRA and EGMA subtasks were part of the student's curriculum, and determining whether students could understand the questions based on the Hindi translations that were used. NFER modified the questions based on the feedback.

Training of TAs: GHTC hired a team of TAs to administer EGRA and EGMA. We divided them into EGRA and EGMA teams and were trained accordingly. For six days, the independent consultant and The trial coordinator trained TA teams on EGRA and EGMA. The rigorous training included classroom interactions, practice sessions (on paper and using electronic tablets) and multiple mock sessions on children from non-trial villages.

Piloting of EGRA and EGMA tests: trained TAs piloted the tests on children in two non-trial villages using tablets. NFER made a few minor modifications to the questions based on the pilot results.

The final EGRA and EGMA tests were uploaded onto the Tangerine platform - https://www.tangerinecentral.org/tangerine to facilitate the application of EGRA and EGMA), which provided an opportunity for immediate online uploads of the test scores and monitoring of the data.

Implementation: Tests were conducted about one month after the intervention teams had stopped the main activities (between 24 July and 19 September 2022). To ensure the confidentiality of the EGRA and EGMA tests, testing was conducted only for one day in a trial village, and GHTC prepared a village-wise schedule accordingly. More teams worked simultaneously to ensure that the tests were performed quickly across 196 villages.

VEs informed the caregivers in advance and mobilised the children to a testing location on the test day. VEs gave admit cards to caregivers during the child mobilisation exercise. The admit cards indicated the test date, test location and child ID. The child mobilisation exercise was monitored by a team of supervisors, who accompanied the VE, ensuring that only enlisted children were mobilised and also increasing attendance.

On the test day, the children were mobilised at a central location at staggered timings so that during the day, the children attended for testing at different timings, ensuring the confidentiality of the testing test. Only children who carried admit cards were allowed to take the tests. The TAs cross-checked by asking the child to mention their details relating to key identifiers. The child was allowed to take the test if the details on the tablet and the child’s admit card were the same.

Pre-entered identifiers and IDs on the tablets minimised the risk of data entry errors by TAs. Pre-entered data aided immediate and error-free linking of the child's current scores to the forms/tests that had already been completed.

Most of the test locations identified for conducting the final tests were neutral points and did not have any indications/ambience of a school environment. On the test day, children were mobilised and waited at a distance from the testing place. Each child took the tests sequentially in two separate rooms (one for reading and one for mathematics). A list of warm-up measures was available to each TA to help the child relax before the test. A team of supervisors and field coordinators ensured that the test location was not accessed by any other person in the village, including the family members of the children. This enabled the teams to provide and maintain a quiet and enabling environment for the child to undertake the test. The EGRA and EGMA tests were administered orally and sequentially by two groups of TAs (one group each for EGRA and EGMA). They were otherwise not involved in the trial and unaware of randomisation. Tests were administered in one-on-one sessions to each participant child present in the village on the day of the assessment.

Quality assurance and control: the independent consultant and trial coordinator conducted random surprise checks at the test locations using NFER checklists while TAs were administering the EGRA and EGMA tests. At the end of each assessment day, the independent consultant and the trial coordinator checked the Tangerine data for quality and feedback to the TAs accordingly. We noticed improvements among the TAs post-feedback. This real-time data check and discussion was helpful for corrective actions and boosted the confidence of TAs during testing.

Caregiver surveys

Caregivers were interviewed twice during the midline and endline to assess child's enrolment details which included grades, type of school and attendance at the school. Questions were also asked about the level of engagement of the parents with the child's education and the costs involved.
