# Supplementary material for: Support To Rural India’s Public Education System (STRIPES2) and impact on numeracy and literacy scores: A cluster randomized trial in rural villages of Madhya Pradesh, India
Source: PLoS One. 2025 Sep 12;20(9):e0330203. doi: 10.1371/journal.pone.0330203 (PMC12431668; doi:10.1371/journal.pone.0330203)
Supplement: S7 Appendix — (PDF) [file pone.0330203.s007.pdf]

Appendix 7- Statistical Analysis - Full results

Adherence:

Summaries of adherence to intervention are given below in Table S1.

We defined adherence at child level for the *j*th child in the *i*th village in three ways:

a) Attended as a proportion of ideal $A_{ij}/360$,
b) Offered as a proportion of ideal $O_{ij}/360$,
c) Attended as a proportion of offered A_{ij}/O_{ij}.

Where more than 360 classes were offered or attended, we took the number(s) to be 360.

At village level and using N_i to denote the number of children in the *i*th village, we defined adherence in the same three ways:

a) Attended as a proportion of ideal $\left(\sum_j A_{ij}\right)/(360N_i)$,
b) Offered as a proportion of ideal $\left(\sum_j O_{ij}\right)/(360N_i)$,
c) Attended as a proportion of offered $\left(\sum_j A_{ij}\right)/\left(\sum_j O_{ij}\right)$.

Summaries of these six definitions of adherence can be found in Table S1. Note that for cluster-level measures, summaries of the continuous measures of adherence (i.e. the last three cells of the first row) are means (and standard deviations) of the cluster-level means and summaries of the categorical measures of adherence are means (and standard deviations) of the cluster-level proportions.

Table S1. Class adherence, intervention arm only.

	**Adherence measure**					
	**Child level measures**			**Cluster level measures**		
	**Attended as a proportion of ideal (N=3405)**	**Offered as a proportion of ideal (N=3405)**	**Attended as a proportion of offered (N=3405)**	**Attended as a proportion of ideal (N=98) mean (SD)**	**Offered as a proportion of ideal (N=98) mean (SD)**	**Attended as a proportion of offered (N=98) mean (SD)**
Mean (SD)	0.53 (0.36)	0.77 (0.36)	0.58 (0.24)	0.52 (0.13)	0.75 (0.13)	0.59 (0.08)
0	434 (13%)	412 (12%)	22 (1%)	0.13 (0.09)	0.13 (0.09)	0.00 (0.01)
>0 and ≤25%	616 (18%)	129 (4%)	326 (10%)	0.20 (0.13)	0.04 (0.09)	0.10 (0.09)
>25% and ≤50%	432 (13%)	205 (6%)	494 (15%)	0.12 (0.08)	0.06 (0.08)	0.15 (0.09)
>50% and ≤75%	664 (20%)	252 (7%)	757 (22%)	0.19 (0.11)	0.08 (0.09)	0.22 (0.12)
>75% and <100%	834 (24%)	512 (15%)	960 (28%)	0.24 (0.10)	0.15 (0.18)	0.28 (0.11)
100%	425 (12%)	1,895 (56%)	434 (13%)	0.13 (0.12)	0.54 (0.25)	0.13 (0.12)
Missing	0 (0%)	0 (0%)	412 (12%)	0 (0)	0 (0)	0.13 (0.09)

In addition, summaries of attendance and completion of activities at mothers' meetings are given below in Table S2.

Table S2. Mothers' group meetings attendance and activities, intervention arm only.

	Adherence measure					
	Child level measures			Cluster level measures		
	Attended as a proportion of ideal (N=3405)	Did language activity as a proportion of ideal (N=3405)	Did maths activity as a proportion of ideal (N=3405)	Attended as a proportion of ideal (N=98)	Did language activity as a proportion of ideal (N=98)	Did maths activity as a proportion of ideal (N=98)
Mean (SD)	0.50 (0.37)	0.45 (0.35)	0.45 (0.35)	0.49 (0.13)	0.44 (0.13)	0.44 (0.13)
0	838 (25%)	868 (25%)	868 (25%)	0.27 (0.15)	0.28 (0.15)	0.28 (0.15)
>0 and ≤25%	323 (9%)	411 (12%)	407 (12%)	0.09 (0.07)	0.12 (0.10)	0.11 (0.10)
>25% and ≤50%	419 (12%)	492 (14%)	513 (15%)	0.12 (0.09)	0.14 (0.11)	0.15 (0.11)
>50% and ≤75%	742 (22%)	788 (23%)	774 (23%)	0.20 (0.13)	0.22 (0.12)	0.21 (0.12)
>75% and <100%	886 (26%)	714 (21%)	718 (21%)	0.26 (0.13)	0.21 (0.12)	0.21 (0.13)
100%	197 (6%)	132 (4%)	125 (4%)	0.06 (0.09)	0.04 (0.07)	0.04 (0.07)
Missing	0 (0%)	0 (0%)	0 (0%)	0 (0%)	0 (0%)	0 (0%)

Table S3. Baseline characteristics of villages (randomisation stratification factors).

Variable	Intervention arm N=98	Control arm N=98
Village size (total population)		
Mean (SD)	1504.8 (491.6)	1459.1 (515.3)
Median (IQR)	1384.5 (1138.0-1967.0)	1371.0 (1063.0-1894.0)
Distance (km) to nearest Community Hospital/ Community Health Centre		
Mean (SD)	12.5 (5.5)	12.6 (5.5)
Median (IQR)	11.3 (8.2-15.6)	11.4 (8.5-16.7)

Summaries of the baseline characteristics of the children are given in Table S4. Note that for cluster-level measures, summaries of continuous measures are means (and standard deviations) of the cluster-level means and summaries of the categorical measures are means (and standard deviations) of the cluster-level proportions.

Table S4. Baseline characteristics of the children.

Variable	Intervention arm		Control arm	
	Child level N=3405	Cluster level N=98 mean (SD)	Child level N=3667	Cluster level N=98 mean (SD)
Female	1,641 (48%)	0.48 (0.10)	1,790 (49%)	0.49 (0.10)
Family Religion:				
Hindu	3,354 (99%)	0.99 (0.04)	3,615 (99%)	0.98 (0.03)
Muslim	26 (1%)	0.01 (0.03)	14 (0%)	0.00 (0.02)
Missing	25 (1%)	0.01 (0.02)	38 (1%)	0.01 (0.03)
Family Caste:				
Schedule caste	723 (21%)	0.21 (0.16)	821 (22%)	0.22 (0.17)
Schedule tribe	936 (27%)	0.27 (0.21)	1,102 (30%)	0.28 (0.26)
Other Backward caste	1,330 (39%)	0.39 (0.22)	1,263 (34%)	0.36 (0.21)
Forward caste	391 (11%)	0.12 (0.14)	443 (12%)	0.13 (0.12)
Missing	25 (1%)	0.01 (0.02)	38 (1%)	0.01 (0.03)
Child's main female caregiver:				
Biological mother	3,306 (97%)	0.97 (0.03)	3,561 (97%)	0.97 (0.03)
Step mother	8 (0%)	0.00 (0.01)	9 (0%)	0.00 (0.01)
Grandmother	31 (1%)	0.01 (0.02)	37 (1%)	0.01 (0.02)
Other female family member	10 (0%)	0.00 (0.01)	8 (0%)	0.00 (0.01)
Other	35 (1%)	0.01 (0.02)	45 (1%)	0.01 (0.02)
No female caregiver	15 (0%)	0.00 (0.01)	7 (0%)	0.00 (0.01)
Child's main male caregiver:				
Biological father	3,289 (97%)	0.96 (0.04)	3,528 (96%)	0.96 (0.04)
Step father	5 (0%)	0.00 (0.01)	7 (0%)	0.00 (0.01)
Grandfather	26 (1%)	0.01 (0.02)	33 (1%)	0.01 (0.02)
Other male family member	14 (0%)	0.00 (0.01)	16 (0%)	0.00 (0.01)
Other	43 (1%)	0.01 (0.02)	55 (1%)	0.01 (0.02)
No male caregiver	28 (1%)	0.01 (0.02)	28 (1%)	0.01 (0.02)
Mother's education:				
No schooling	1,004 (29%)	0.28 (0.15)	1,107 (30%)	0.28 (0.14)
Primary	765 (22%)	0.22 (0.09)	933 (25%)	0.26 (0.09)
Middle school	900 (26%)	0.27 (0.10)	939 (26%)	0.26 (0.11)
High school	497 (15%)	0.15 (0.10)	465 (13%)	0.13 (0.09)
Higher secondary	129 (4%)	0.04 (0.04)	116 (3%)	0.03 (0.04)
Graduate	48 (1%)	0.01 (0.03)	39 (1%)	0.01 (0.02)
Postgraduate	17 (0%)	0.01 (0.01)	15 (0%)	0.00 (0.02)
Missing	45 (1%)	0.01 (0.02)	53 (1%)	0.02 (0.03)

Father's education:				
No schooling	472 (14%)	0.13 (0.10)	606 (17%)	0.15 (0.12)
Primary	601 (18%)	0.17 (0.09)	645 (18%)	0.18 (0.09)
Middle school	987 (29%)	0.29 (0.11)	1,042 (28%)	0.29 (0.09)
High school	823 (24%)	0.25 (0.13)	795 (22%)	0.22 (0.11)
Higher secondary	280 (8%)	0.08 (0.07)	323 (9%)	0.09 (0.06)
Graduate	108 (3%)	0.03 (0.04)	118 (3%)	0.03 (0.04)
Postgraduate	55 (2%)	0.02 (0.02)	29 (1%)	0.01 (0.01)
Missing	79 (2%)	0.02 (0.04)	109 (3%)	0.03 (0.04)
Child's age	6.9 (0.7)	6.9 (0.2)	6.9 (0.7)	6.9 (0.2)
Mother alive at baseline	3,359 (99%)	0.99 (0.02)	3,629 (99%)	0.99 (0.02)
Father alive at baseline	3,352 (98%)	0.98 (0.03)	3,603 (98%)	0.98 (0.03)
Mother literacy:				
Can't read	1,486 (44%)	0.42 (0.16)	1,650 (45%)	0.44 (0.14)
Can read part of the sentence	582 (17%)	0.17 (0.09)	649 (18%)	0.18 (0.09)
Read entire sentence	1,038 (30%)	0.32 (0.15)	1,014 (28%)	0.29 (0.13)
Missing	299 (9%)	0.09 (0.07)	354 (10%)	0.10 (0.06)
Father literacy:				
Can't read	342 (10%)	0.10 (0.07)	421 (11%)	0.11 (0.08)
Can read part of the sentence	236 (7%)	0.07 (0.05)	266 (7%)	0.08 (0.06)
Read entire sentence	796 (23%)	0.24 (0.10)	856 (23%)	0.24 (0.11)
Missing*	2,031 (60%)	0.60 (0.11)	2,124 (58%)	0.58 (0.12)

*During enumeration father not present to read literacy card.

Table S4A. Baseline characteristics in the intervention arm according to subsequent adherence (post-hoc analysis)

**Variable**	**Adherence (%)**					
	0	>0 & ≤25	>25 & ≤50	>50 & ≤75	>75 & <100	100
Female	195 (44.9%)	274 (44.5%)	204 (47.2%)	328 (49.4%)	435 (52.2%)	205 (48.2%)
Family Religion:						
Hindu	428 (98.6%)	612 (99.4%)	424 (98.1%)	654 (98.5%)	819 (98.2%)	417 (98.1%)
Muslim	4 (0.9%)	2 (0.3%)	4 (0.9%)	4 (0.6%)	8 (1.0%)	4 (0.9%)
Missing	2 (0.5%)	2 (0.3%)	4 (0.9%)	6 (0.9%)	7 (0.8%)	4 (0.9%)
Family Caste:						
Schedule caste	86 (19.8%)	112 (18.2%)	116 (26.9%)	156 (23.5%)	191 (22.9%)	62 (14.6%)
Schedule tribe	111 (25.6%)	209 (33.9%)	140 (32.4%)	201 (30.3%)	193 (23.1%)	82 (19.3%)
Other Backward caste	150 (34.6%)	229 (37.2%)	135 (31.2%)	247 (37.2%)	357 (42.8%)	212 (49.9%)
Forward caste	85 (19.6%)	64 (10.4%)	37 (8.6%)	54 (8.1%)	86 (10.3%)	65 (15.3%)
Missing	2 (0.5%)	2 (0.3%)	4 (0.9%)	6 (0.9%)	7 (0.8%)	4 (0.9%)

Child's main female caregiver:						
Biological mother	407 (93.8%)	595 (96.6%)	417 (96.5%)	652 (98.2%)	815 (97.7%)	420 (98.8%)
Step mother	2 (0.5%)	0 (0.0%)	1 (0.2%)	3 (0.5%)	1 (0.1%)	1 (0.2%)
Grandmother	9 (2.1%)	7 (1.1%)	7 (1.6%)	4 (0.6%)	3 (0.4%)	1 (0.2%)
Other female family member	5 (1.2%)	2 (0.3%)	0 (0.0%)	1 (0.2%)	1 (0.1%)	1 (0.2%)
Other	8 (1.8%)	8 (1.3%)	6 (1.4%)	2 (0.3%)	9 (1.1%)	2 (0.5%)
No female caregiver	3 (0.7%)	4 (0.6%)	1 (0.2%)	2 (0.3%)	5 (0.6%)	0 (0.0%)
Child's main male caregiver:						
Biological father	403 (92.9%)	597 (96.9%)	417 (96.5%)	651 (98.0%)	807 (96.8%)	414 (97.4%)
Step father	2 (0.5%)	1 (0.2%)	0 (0.0%)	1 (0.2%)	1 (0.1%)	0 (0.0%)
Grandfather	11 (2.5%)	5 (0.8%)	4 (0.9%)	2 (0.3%)	1 (0.1%)	3 (0.7%)
Other male family member	4 (0.9%)	2 (0.3%)	0 (0.0%)	2 (0.3%)	5 (0.6%)	1 (0.2%)
Other	10 (2.3%)	8 (1.3%)	8 (1.9%)	2 (0.3%)	13 (1.6%)	2 (0.5%)
No male caregiver	4 (0.9%)	3 (0.5%)	3 (0.7%)	6 (0.9%)	7 (0.8%)	5 (1.2%)
Mother's education:						
No schooling	133 (30.6%)	197 (32.0%)	142 (32.9%)	216 (32.5%)	215 (25.8%)	101 (23.8%)
Primary	80 (18.4%)	123 (20.0%)	96 (22.2%)	168 (25.3%)	203 (24.3%)	95 (22.4%)
Middle school	96 (22.1%)	165 (26.8%)	102 (23.6%)	169 (25.5%)	245 (29.4%)	123 (28.9%)
High school	65 (15.0%)	84 (13.6%)	63 (14.6%)	82 (12.3%)	123 (14.7%)	80 (18.8%)
Higher secondary	33 (7.6%)	23 (3.7%)	17 (3.9%)	15 (2.3%)	26 (3.1%)	15 (3.5%)
Graduate	12 (2.8%)	16 (2.6%)	5 (1.2%)	4 (0.6%)	6 (0.7%)	5 (1.2%)
Postgraduate	7 (1.6%)	1 (0.2%)	2 (0.5%)	2 (0.3%)	3 (0.4%)	2 (0.5%)
Missing	8 (1.8%)	7 (1.1%)	5 (1.2%)	8 (1.2%)	13 (1.6%)	4 (0.9%)
Father's education:						
No schooling	70 (16.1%)	100 (16.2%)	61 (14.1%)	113 (17.0%)	97 (11.6%)	31 (7.3%)
Primary	82 (18.9%)	100 (16.2%)	86 (19.9%)	109 (16.4%)	158 (18.9%)	66 (15.5%)
Middle school	102 (23.5%)	178 (28.9%)	124 (28.7%)	196 (29.5%)	236 (28.3%)	151 (35.5%)
High school	95 (21.9%)	144 (23.4%)	98 (22.7%)	157 (23.6%)	213 (25.5%)	116 (27.3%)
Higher secondary	41 (9.4%)	52 (8.4%)	30 (6.9%)	49 (7.4%)	72 (8.6%)	36 (8.5%)
Graduate	23 (5.3%)	14 (2.3%)	13 (3.0%)	18 (2.7%)	32 (3.8%)	8 (1.9%)
Postgraduate	12 (2.8%)	17 (2.8%)	8 (1.9%)	5 (0.8%)	8 (1.0%)	5 (1.2%)
Missing	9 (2.1%)	11 (1.8%)	12 (2.8%)	17 (2.6%)	18 (2.2%)	12 (2.8%)
Child's age	6.9 (0.8)	6.9 (0.7)	6.9 (0.7)	6.9 (0.7)	6.9 (0.7)	6.9 (0.7)
Mother alive at baseline	425 (97.9%)	606 (98.4%)	427 (98.8%)	657 (98.9%)	823 (98.7%)	421 (99.1%)
Father alive at baseline	421 (97.0%)	610 (99.0%)	427 (98.8%)	657 (98.9%)	818 (98.1%)	419 (98.6%)
Mother literacy:						
Can't read	172 (39.6%)	291 (47.2%)	204 (47.2%)	312 (47.0%)	349 (41.8%)	158 (37.2%)
Can read part of the sentence	62 (14.3%)	92 (14.9%)	67 (15.5%)	113 (17.0%)	158 (18.9%)	90 (21.2%)
Read entire sentence	127 (29.3%)	187 (30.4%)	124 (28.7%)	192 (28.9%)	256 (30.7%)	152 (35.8%)
Missing	73 (16.8%)	46 (7.5%)	37 (8.6%)	47 (7.1%)	71 (8.5%)	25 (5.9%)

Father literacy:						
Can't read	49 (11.3%)	67 (10.9%)	53 (12.3%)	78 (11.7%)	70 (8.4%)	25 (5.9%)
Can read part of the sentence	26 (6.0%)	55 (8.9%)	26 (6.0%)	47 (7.1%)	60 (7.2%)	22 (5.2%)
Read entire sentence	113 (26.0%)	131 (21.3%)	96 (22.2%)	142 (21.4%)	199 (23.9%)	115 (27.1%)
Missing*	246 (56.7%)	363 (58.9%)	257 (59.5%)	397 (59.8%)	505 (60.6%)	263 (61.9%)

*During enumeration father not present to read literacy card.

Table S4B. Baseline characteristics of the children according to whether or not the primary outcome was observed (post-hoc analysis).

Variable	Intervention arm		Control arm	
	Outcome missing N=351	Outcome observed N=3,054	Outcome missing N=392	Outcome observed N=3,275
Female	166 (47.3%)	1,475 (48.3%)	183 (46.7%)	1,607 (49.1%)
Family Religion:				
Hindu	345 (98.3%)	3,009 (98.5%)	384 (98.0%)	3,231 (98.7%)
Muslim	3 (0.9%)	23 (0.8%)	3 (0.8%)	11 (0.3%)
Missing	3 (0.9%)	22 (0.7%)	5 (1.3%)	33 (1.0%)
Family Caste:				
Schedule caste	69 (19.7%)	654 (21.4%)	88 (22.4%)	733 (22.4%)
Schedule tribe	85 (24.2%)	851 (27.9%)	128 (32.7%)	974 (29.7%)
Other Backward caste	126 (35.9%)	1,204 (39.4%)	105 (26.8%)	1,158 (35.4%)
Forward caste	68 (19.4%)	323 (10.6%)	66 (16.8%)	377 (11.5%)
Missing	3 (0.9%)	22 (0.7%)	5 (1.3%)	33 (1.0%)
Child's main female caregiver:				
Biological mother	332 (94.6%)	2,974 (97.4%)	372 (94.9%)	3,189 (97.4%)
Step mother	0 (0.0%)	8 (0.3%)	0 (0.0%)	9 (0.3%)
Grandmother	6 (1.7%)	25 (0.8%)	5 (1.3%)	32 (1.0%)
Other female family member	3 (0.9%)	7 (0.2%)	2 (0.5%)	6 (0.2%)
Other	8 (2.3%)	27 (0.9%)	13 (3.3%)	32 (1.0%)
No female caregiver	2 (0.6%)	13 (0.4%)	0 (0.0%)	7 (0.2%)
Child's main male caregiver:				
Biological father	321 (91.5%)	2,968 (97.2%)	359 (91.6%)	3,169 (96.8%)
Step father	3 (0.9%)	2 (0.1%)	2 (0.5%)	5 (0.2%)
Grandfather	7 (2.0%)	19 (0.6%)	8 (2.0%)	25 (0.8%)
Other male family member	6 (1.7%)	8 (0.3%)	3 (0.8%)	13 (0.4%)
Other	11 (3.1%)	32 (1.0%)	17 (4.3%)	38 (1.2%)
No male caregiver	3 (0.9%)	25 (0.8%)	3 (0.8%)	25 (0.8%)

Mother's education:				
No schooling	82 (23.4%)	922 (30.2%)	106 (27.0%)	1,001 (30.6%)
Primary	65 (18.5%)	700 (22.9%)	80 (20.4%)	853 (26.0%)
Middle school	96 (27.4%)	804 (26.3%)	101 (25.8%)	838 (25.6%)
High school	58 (16.5%)	439 (14.4%)	69 (17.6%)	396 (12.1%)
Higher secondary	25 (7.1%)	104 (3.4%)	20 (5.1%)	96 (2.9%)
Graduate	12 (3.4%)	36 (1.2%)	5 (1.3%)	34 (1.0%)
Postgraduate	6 (1.7%)	11 (0.4%)	4 (1.0%)	11 (0.3%)
Missing	7 (2.0%)	38 (1.2%)	7 (1.8%)	46 (1.4%)
Father's education:				
No schooling	39 (11.1%)	433 (14.2%)	66 (16.8%)	540 (16.5%)
Primary	63 (17.9%)	538 (17.6%)	76 (19.4%)	569 (17.4%)
Middle school	99 (28.2%)	888 (29.1%)	96 (24.5%)	946 (28.9%)
High school	79 (22.5%)	744 (24.4%)	75 (19.1%)	720 (22.0%)
Higher secondary	34 (9.7%)	246 (8.1%)	47 (12.0%)	276 (8.4%)
Graduate	20 (5.7%)	88 (2.9%)	16 (4.1%)	102 (3.1%)
Postgraduate	8 (2.3%)	47 (1.5%)	3 (0.8%)	26 (0.8%)
Missing	9 (2.6%)	70 (2.3%)	13 (3.3%)	96 (2.9%)
Child's age	6.9 (0.7)	6.9 (0.7)	6.9 (0.7)	6.9 (0.7)
Mother alive at baseline	347 (98.9%)	3,012 (98.6%)	390 (99.5%)	3,239 (98.9%)
Father alive at baseline	337 (96.0%)	3,015 (98.7%)	379 (96.7%)	3,224 (98.4%)
Mother literacy:				
Can't read	116 (33.0%)	1,370 (44.9%)	152 (38.8%)	1,498 (45.7%)
Can read part of the sentence	56 (16.0%)	526 (17.2%)	55 (14.0%)	594 (18.1%)
Read entire sentence	121 (34.5%)	917 (30.0%)	137 (34.9%)	877 (26.8%)
Missing	58 (16.5%)	241 (7.9%)	48 (12.2%)	306 (9.3%)
Father literacy:				
Can't read	30 (8.5%)	312 (10.2%)	53 (13.5%)	368 (11.2%)
Can read part of the sentence	17 (4.8%)	219 (7.2%)	27 (6.9%)	239 (7.3%)
Read entire sentence	100 (28.5%)	696 (22.8%)	91 (23.2%)	765 (23.4%)
Missing*	204 (58.1%)	1,827 (59.8%)	221 (56.4%)	1,903 (58.1%)

*During enumeration father not present to read literacy card.

Table S5. EGRA and EGMA test results.

Variable	Intervention arm		Control arm		Difference* (95% CI) p-value
	Child level N: mean (SD)	Cluster level N=98 mean (SD)	Child level N: mean (SD)	Cluster level N=98 mean (SD)	
Composite test score	3,054 56.30 (25.03)	56.71 (8.41)	3,275 42.25 (25.74)	42.30 (11.18)	14.17 (11.36, 16.97) p<0.001
Composite test score -sensitivity analysis**	3,054 56.59 (25.17)	57.01 (8.46)	3,275 42.42 (25.91)	42.46 (11.26)	14.29 (11.47, 17.11) p<0.001
Mathematics test score, overall	3,058 55.52 (27.26)	55.69 (9.19)	3,282 41.75 (27.86)	41.75 (11.68)	13.90 (10.91, 16.88) p<0.001
Mathematics test, fluency score 1	3,058 23.11 (18.29)	23.78 (7.27)	3,282 16.38 (16.66)	16.68 (6.21)	
Mathematics test, fluency score 2	3,058 10.90 (6.51)	10.91 (1.87)	3,282 8.49 (6.68)	8.45 (2.58)	
Mathematics test, fluency score 3	3,058 7.81 (4.84)	7.79 (1.72)	3,282 5.92 (5.16)	5.91 (2.05)	
Mathematics 1	3,058 70.14 (29.94)	70.70 (9.87)	3,282 54.21 (32.54)	54.62 (13.24)	
Mathematics 2	3,058 68.84 (32.61)	69.27 (10.94)	3,282 51.73 (36.10)	52.25 (14.68)	
Mathematics 3	3,058 51.73 (28.07)	51.97 (8.84)	3,282 39.25 (26.80)	39.21 (10.30)	
Mathematics 4a	3,058 52.16 (30.50)	52.20 (8.90)	3,282 40.28 (31.59)	40.07 (12.65)	
Mathematics 4b	3,058 55.58 (38.32)	55.62 (12.34)	3,282 37.96 (37.15)	37.51 (15.53)	
Mathematics 5a	3,058 37.67 (23.83)	37.52 (8.55)	3,282 27.81 (24.40)	27.79 (10.06)	
Mathematics 5b	3,058 44.98 (37.52)	45.02 (12.87)	3,282 28.80 (34.88)	28.55 (13.94)	
Mathematics 6	3,058 47.20 (30.83)	47.01 (10.75)	3,282 37.88 (29.63)	37.49 (10.78)	
Language test score, overall	3,055 57.06 (25.25)	57.71 (8.51)	3,288 42.73 (25.95)	42.85 (11.32)	14.44 (11.54, 17.34) p<0.001
Language test score, overall - sensitivity analysis**	3,055 57.64 (25.57)	58.31 (8.60)	3,288 43.06 (26.30)	43.16 (11.47)	14.69 (11.76, 17.62) p<0.001
Language test, fluency score 1	3,055 51.89 (24.03)	52.69 (7.78)	3,288 40.12 (25.45)	40.60 (11.49)	
Language test, fluency score 2	3,055 20.28 (12.76)	20.36 (4.38)	3,288 14.30 (12.82)	14.21 (5.43)	
Language test, fluency score 3	3,055 32.01 (24.13)	32.60 (7.95)	3,288 19.92 (21.98)	19.91 (8.39)	
Language test, fluency score 4	3,055 46.95 (33.18)	47.89 (11.00)	3,288 29.45 (31.47)	29.59 (12.30)	
Language 1	3,055 51.80 (23.64)	52.57 (7.79)	3,288 40.11 (25.28)	40.60 (11.47)	

Language 2	3,055<br>51.27 (30.01)	52.33<br>(11.12)	3,288<br>41.93 (28.92)	42.34 (10.77)
Language 3	3,055<br>40.24 (24.57)	40.36<br>(8.62)	3,288<br>28.39 (24.81)	28.19 (10.76)
Language 4	3,055<br>55.64 (35.02)	56.39<br>(11.37)	3,288<br>35.73 (34.75)	35.69 (14.02)
Language 5a	3,055<br>63.10 (35.97)	63.92<br>(11.33)	3,288<br>41.06 (37.77)	41.21 (15.76)
Language 5b	3,055<br>51.11 (35.75)	52.01<br>(11.25)	3,288<br>31.61 (34.69)	31.79 (14.34)
Language 5b - sensitivity analysis	3,055<br>55.16 (37.90)	56.19<br>(11.91)	3,288<br>33.92 (37.22)	33.96 (15.33)
Language 6	3,055<br>86.26 (23.24)	86.39<br>(7.49)	3,288<br>80.27 (27.21)	80.16 (9.31)

*Differences estimated at child level and adjusted for randomisation stratification factors.

**Omitting the score from EGRA subtask 5b question

Intention-to-treat results

The intention-to-treat estimate of effectiveness on the primary outcome is 14.17 (95% CI 11.36, 16.97; p<0.001; note, this is shown in Table S5), adjusting for the randomisation stratification factors. The standardised intention-to-treat estimate is 0.58 (95% CI 0.47, 0.71). As a sensitivity analysis we used multiple imputation to address missing primary outcome data. The estimated intervention effect was 14.04 (95% CI 9.17, 18.90; p<0.001), adjusting for the randomisation stratification variables, very similar to that from the primary analysis.

Per protocol results

We defined the per-protocol population as those children who attended more than 75% of the ideal number of sessions (if allocated to the intervention arm). There were 1259 (37%) such children in the intervention arm. The mean composite test score was 66.19 (SD 19.42) for these children. The per-protocol estimate was 23.77 (95% CI 20.86, 26.68; p<0.001).

Subgroup analyses

The results of subgroup analyses are shown in Table S6. Numbers of observations, means and standard deviations are given by trial arm for each level of the moderators that we investigated. These summaries are given at child level and cluster level. Intervention effects are estimated at child level within levels of the moderators and adjusting for the randomisation stratification variables. The final column provides p-values of the tests of intervention interaction, i.e. tests of the null hypothesis that the effects of the intervention are the same across subgroups for a given moderator.

Table S6. Composite test scores by subgroup, with interaction tests.

Moderator variable<br>Subgroup	Intervention arm		Control arm		Difference* (95% CI)	p-value
	Child level N: mean (SD)	Cluster level N: mean (SD)	Child level N: mean (SD)	Cluster level N: mean (SD)		
Village population						
Below median	1213<br>54.15 (25.36)	49<br>54.73 (8.55)	1241<br>37.36 (25.41)	49<br>39.40 (12.68)	16.69 (11.66, 21.73)	p=0.174
Above median	1841<br>57.72 (24.72)	49<br>58.68 (7.86)	2034<br>45.24 (25.49)	49<br>45.21 (8.63)	12.56 (9.38, 15.75)	
Gender						
Male	1579<br>56.16 (25.58)	98<br>56.61 (9.22)	1668<br>43.76 (25.67)	98<br>44.08 (12.16)	12.61 (9.54, 15.67)	p=0.023
Female	1475<br>56.45 (24.44)	98<br>57.06 (10.02)	1607<br>40.69 (25.74)	98<br>40.64 (12.69)	15.79 (12.60, 18.97)	
Wealth index 1						
House made of all natural materials	155<br>55.78 (25.64)	36<br>53.83 (18.03)	116<br>39.13 (25.73)	33<br>40.55 (18.71)	17.21 (10.63, 23.79)	p<0.001
House made of natural/synthetic materials	2062<br>54.34 (24.84)	98<br>55.01 (9.10)	2282<br>38.20 (25.04)	98<br>39.29 (11.25)	16.08 (13.09, 19.07)	
House made of all synthetic materials	780<br>62.90 (23.60)	94<br>65.65 (12.85)	795<br>54.92 (23.52)	92<br>54.52 (13.19)	8.63 (5.12, 12.14)	
Wealth index 2 (items owned)**						
0	1156<br>50.05 (25.52)	97<br>49.94 (11.33)	1257<br>34.88 (24.14)	98<br>36.11 (12.56)	15.10 (11.66, 18.53)	p=0.583 (trend test)
1	908<br>57.31 (23.72)	97<br>57.43 (11.12)	1006<br>43.63 (24.89)	98<br>42.63 (12.45)	13.75 (10.44, 17.05)	
2	637<br>66.42 (20.79)	96<br>65.56 (12.55)	656<br>53.64 (24.43)	94<br>53.08 (15.61)	13.29 (10.21, 16.38)	
3 or 4	142<br>71.69 (19.56)	68<br>72.77 (15.12)	110<br>58.70 (23.83)	58<br>56.19 (19.23)	13.31 (7.70, 18.92)	
Caste						
Schedule caste	654<br>56.31 (24.41)	89<br>57.54 (15.11)	733<br>42.16 (23.75)	85<br>40.63 (16.71)	14.21 (10.24, 18.17)	p=0.411
Schedule tribe	851<br>43.11 (24.33)	85<br>42.40 (12.26)	974<br>27.38 (21.14)	84<br>30.75 (15.77)	15.56 (11.53, 19.60)	
Other backward caste	1204<br>61.57 (23.17)	97<br>61.54 (11.93)	1158<br>48.66 (24.61)	95<br>47.83 (13.21)	13.06 (9.81, 16.31)	
Forward caste	323<br>71.06 (18.56)	74<br>72.36 (12.12)	377<br>60.34 (23.35)	75<br>57.69 (17.65)	11.23 (7.09, 15.37)	

Female care-giver literacy						
Can't read	1370<br>48.53 (25.41)	98<br>49.04 (11.56)	1498<br>33.71 (23.48)	98<br>34.82 (11.21)	14.90 (11.53, 18.27)	
Can read part of the sentence	526<br>57.79 (22.82)	96<br>57.80 (14.16)	594<br>41.43 (24.60)	97<br>41.57 (15.49)	16.32 (13.01, 19.63)	p=0.010
Read entire sentence	917<br>67.87 (20.45)	97<br>67.64 (7.29)	877<br>57.23 (23.33)	96<br>53.94 (13.28)	10.88 (8.33, 13.44)	
Male care-giver education						
No schooling	433<br>41.49 (25.63)	90<br>41.19 (18.29)	540<br>28.01 (21.73)	92<br>30.98 (15.67)	13.16 (8.82, 17.51)	
Primary school	538<br>47.72 (24.47)	94<br>48.48 (14.62)	569<br>32.73 (22.86)	97<br>34.93 (15.58)	15.16 (11.15, 19.17)	
Middle school	888<br>55.12 (23.73)	98<br>54.99 (11.31)	946<br>40.96 (24.03)	98<br>38.67 (12.93)	14.29 (11.26, 17.33)	p=0.263
High school	744<br>65.16 (21.03)	97<br>65.25 (10.29)	720<br>51.35 (24.29)	94<br>49.89 (13.02)	14.03 (10.80, 17.26)	
Higher secondary school	246<br>71.09 (19.08)	81<br>69.55 (14.35)	276<br>60.09 (22.59)	81<br>57.66 (17.81)	11.28 (7.20, 15.36)	
Graduate	88<br>73.87 (19.08)	55<br>73.89 (17.70)	102<br>66.78 (20.19)	59<br>67.14 (17.91)	7.05 (1.11, 12.99)	
Postgraduate	47<br>76.19 (15.54)	32<br>74.90 (14.30)	26<br>70.04 (20.96)	20<br>73.38 (15.93)	6.50 (-5.54, 18.54)	
Pregnancy in Household (post-hoc analysis)						
No	2495<br>56.71 (25.03)	98<br>56.72 (8.41)	2707<br>42.99 (25.56)	98<br>42.85 (11.08)	13.86 (11.12, 16.59)	p=0.287
Yes	559<br>54.49 (25.00)	95<br>56.21 (16.40)	568<br>38.73 (26.36)	98<br>39.81 (16.11)	15.76 (11.35, 20.17)	

*Differences estimated at child level and adjusted for randomisation stratification factors.

**Wealth index 2. Number of Items (television, radio, motorbike, 4-wheeled vehicle) owned by the household members.

Table S7. Midline test results (using ASER-like exam).

Variable	Intervention arm		Control arm		Difference (95% CI) p-value
	Child level N: mean (SD) or N (%)	Cluster level N=98 mean (SD)	Child level N: mean (SD) or N (%)	Cluster level N=98 mean (SD)	
Mathematics test score	2961<br>2.63 (1.30)	2.68 (0.47)	3171<br>2.04 (1.36)	2.08 (0.56)	0.60 (0.45, 0.74)<br>p<0.001
Beginner level	157 (5%)	0.04 (0.05)	374 (10%)	0.09 (0.08)	
Numbers 1-9	595 (17%)	0.16 (0.10)	1,036 (28%)	0.27 (0.13)	
Numbers 10-99	522 (15%)	0.15 (0.09)	559 (15%)	0.15 (0.08)	
Addition	611 (18%)	0.18 (0.10)	503 (14%)	0.14 (0.09)	
Subtraction	1,076 (32%)	0.33 (0.14)	699 (19%)	0.19 (0.12)	
Missing	444 (13%)	0.14 (0.08)	496 (14%)	0.14 (0.08)	

Language test score	2961<br>2.67 (1.43)	2.72 (0.49)	3171<br>1.86 (1.51)	1.91 (0.63)	0.81 (0.65, 0.97)<br>p<0.001
Beginner level	255 (7%)	0.07 (0.07)	643 (18%)	0.16 (0.14)	
Letters	619 (18%)	0.18 (0.09)	1,118 (30%)	0.31 (0.11)	
Words	304 (9%)	0.09 (0.06)	274 (7%)	0.08 (0.05)	
Paragraph	455 (13%)	0.13 (0.08)	301 (8%)	0.09 (0.06)	
Story	1,328 (39%)	0.40 (0.14)	835 (23%)	0.23 (0.13)	
Missing	444 (13%)	0.14 (0.08)	496 (14%)	0.14 (0.08)	

Table S8. Children enrolled in school.

Variable	Intervention arm		Control arm		Odds ratio* (95% CI) p-value
	Child level N=3405	Cluster level N=98 mean (SD)	Child level N=3667	Cluster level N=98 mean (SD)	
Midline – pre lockdown					
Yes	2,992 (88%)	0.88 (0.08)	3,115 (85%)	0.85 (0.10)	
No	59 (2%)	0.02 (0.04)	148 (4%)	0.04 (0.07)	
Missing	354 (10%)	0.11 (0.07)	404 (11%)	0.11 (0.07)	
Midline – post lockdown					
Yes	2,955 (87%)	0.87 (0.07)	3,104 (85%)	0.85 (0.09)	
No	96 (3%)	0.03 (0.04)	159 (4%)	0.04 (0.06)	
Missing	354 (10%)	0.11 (0.07)	404 (11%)	0.11 (0.07)	
Endline					
Yes	3,076 (90%)	0.91 (0.06)	3,234 (88%)	0.89 (0.08)	1.38 (1.04, 1.83)
No	84 (2%)	0.02 (0.03)	121 (3%)	0.03 (0.05)	p=0.025
Missing	245 (7%)	0.07 (0.05)	312 (9%)	0.08 (0.05)	

*Yes v No ignoring missing

Table S9. Learning support (endline).

Variable	Intervention arm		Control arm		Difference (95% CI) p-value
	Child level N=3405	Cluster level N=98 mean (SD)	Child level N=3667	Cluster level N=98 mean (SD)	
Help with schoolwork					
No	884 (26%)	0.24 (0.20)	895 (24%)	0.24 (0.22)	
Yes	2,188 (64%)	0.66 (0.21)	2,319 (63%)	0.64 (0.24)	
Missing	333 (10%)	0.10 (0.06)	453 (12%)	0.12 (0.09)	
Help with reading or counting activities to promote learning					
No	2,073 (61%)	0.61 (0.16)	2,274 (62%)	0.62 (0.18)	
Yes	999 (29%)	0.30 (0.16)	940 (26%)	0.26 (0.19)	
Missing	333 (10%)	0.10 (0.06)	453 (12%)	0.12 (0.09)	
Hours per week (ignoring missing) engaging with child in reading or counting activities to promote learning	1.25 (2.12)	1.24 (0.69)	1.19 (2.20)	1.13 (0.96)	0.08 (-0.17, 0.32)<br>p=0.530

Table S10. Reported attendance in school, among those enrolled (endline).

	Intervention arm		Control arm		Difference (95% CI) p-value
	Child level N=3405	Cluster level N=98 mean (SD)	Child level N=3667	Cluster level N=98 mean (SD)	
Number of days of school missed in the last two weeks					
0	918 (27%)	0.28 (0.14)	869 (24%)	0.24 (0.12)	
1	416 (12%)	0.12 (0.07)	423 (12%)	0.12 (0.08)	
2	519 (15%)	0.15 (0.07)	562 (15%)	0.15 (0.08)	
3	356 (10%)	0.11 (0.06)	435 (12%)	0.12 (0.07)	
4	216 (6%)	0.06 (0.05)	264 (7%)	0.08 (0.06)	
5	124 (4%)	0.03 (0.03)	138 (4%)	0.04 (0.03)	
6	106 (3%)	0.03 (0.03)	115 (3%)	0.03 (0.04)	
7	92 (3%)	0.03 (0.04)	97 (3%)	0.03 (0.03)	
8	55 (2%)	0.01 (0.02)	69 (2%)	0.02 (0.03)	
9	21 (1%)	0.01 (0.01)	16 (0%)	0.00 (0.01)	
10	72 (2%)	0.02 (0.03)	62 (2%)	0.02 (0.02)	
11	10 (0%)	0.00 (0.01)	7 (0%)	0.00 (0.01)	
12	153 (4%)	0.04 (0.05)	163 (4%)	0.04 (0.05)	
Missing	347 (10%)	0.10 (0.06)	447 (12%)	0.11 (0.08)	
mean (SD)	2.81 (3.24)	2.72 (1.00)	2.89 (3.17)	2.91 (1.08)	-0.10 (-0.40, 0.20) p=0.492

Table S11. Children resident in study village.

Variable	Intervention arm		Control arm	
	Child level N=3405	Cluster level N=98 mean (SD)	Child level N=3667	Cluster level N=98 mean (SD)
Midline survey				
Yes	3,046 (89%)	0.89 (0.07)	3,257 (89%)	0.89 (0.07)
No	5 (0%)	0.00 (0.01)	6 (0%)	0.00 (0.01)
Dead	15 (0%)	0.01 (0.01)	21 (0%)	0.01 (0.01)
Missing	339 (10%)	0.10 (0.07)	383 (10%)	0.11 (0.07)
Endline survey				
Yes	3,150 (93%)	0.92 (0.05)	3,339 (91%)	0.91 (0.05)
No	13 (0%)	0.00 (0.02)	19 (0%)	0.01 (0.02)
Dead	16 (0%)	0.01 (0.01)	24 (1%)	0.01 (0.02)
Missing	226 (7%)	0.07 (0.05)	285 (8%)	0.07 (0.05)

Table S12. School grade of child.

Variable	Intervention arm		Control arm	
	Child level N=3405	Cluster level N=98 mean (SD)	Child level N=3667	Cluster level N=98 mean (SD)
Midline – pre lockdown				
Anganwadi	119 (3%)	0.03 (0.04)	129 (4%)	0.03 (0.05)
Pre-primary/LKG/UKG	138 (4%)	0.04 (0.04)	129 (4%)	0.04 (0.04)
1	886 (26%)	0.26 (0.09)	942 (26%)	0.25 (0.09)
2	1,584 (47%)	0.47 (0.11)	1,658 (45%)	0.46 (0.13)
3	228 (7%)	0.07 (0.06)	225 (6%)	0.07 (0.07)
4	27 (1%)	0.01 (0.02)	22 (0%)	0.01 (0.01)
5	6 (0%)	0.00 (0.01)	6 (0%)	0.00 (0.01)
6	0 (0%)	0.00 (0.00)	1 (0%)	0.00 (0.00)
Don't know	4 (0%)	0.00 (0.01)	3 (0%)	0.00 (0.01)
Missing	413 (12%)	0.12 (0.08)	552 (15%)	0.15 (0.10)
Midline – post lockdown				
Anganwadi	7 (0%)	0.00 (0.01)	8 (0%)	0.00 (0.01)
Pre-primary/LKG/UKG	1 (0%)	0.00 (0.00)	1 (0%)	0.00 (0.00)
1	86 (3%)	0.02 (0.03)	80 (2%)	0.02 (0.04)
2	174 (5%)	0.05 (0.05)	209 (6%)	0.06 (0.04)
3	872 (26%)	0.26 (0.09)	920 (25%)	0.25 (0.10)
4	1,559 (46%)	0.46 (0.11)	1,637 (45%)	0.45 (0.13)
5	223 (7%)	0.06 (0.06)	220 (6%)	0.06 (0.07)
6	26 (1%)	0.01 (0.02)	21 (0%)	0.01 (0.01)
7	5 (0%)	0.00 (0.01)	5 (0%)	0.00 (0.00)
8	0 (0%)	0.00 (0.00)	1 (0%)	0.00 (0.00)
Don't know	2 (0%)	0.00 (0.00)	2 (0%)	0.00 (0.01)
Missing	450 (13%)	0.13 (0.07)	563 (15%)	0.15 (0.09)
Endline				
Anganwadi	0 (0%)	0.00 (0.00)	0 (0%)	0.00 (0.00)
Pre-primary/LKG/UKG	0 (0%)	0.00 (0.00)	0 (0%)	0.00 (0.00)
1	21 (1%)	0.00 (0.01)	39 (1%)	0.01 (0.02)
2	91 (3%)	0.02 (0.03)	104 (3%)	0.03 (0.04)
3	214 (6%)	0.06 (0.06)	263 (7%)	0.07 (0.06)
4	887 (26%)	0.26 (0.09)	940 (26%)	0.25 (0.11)
5	1,608 (47%)	0.48 (0.11)	1,664 (45%)	0.46 (0.13)
6	220 (6%)	0.06 (0.07)	187 (5%)	0.06 (0.07)
7	27 (1%)	0.01 (0.02)	22 (0%)	0.01 (0.01)
8	5 (0%)	0.00 (0.01)	4 (0%)	0.00 (0.00)
Don't know	6 (0%)	0.00 (0.01)	14 (0%)	0.00 (0.02)
Missing	326 (10%)	0.09 (0.06)	430 (12%)	0.11 (0.08)

Table S13. Covid-19 challenges faced (midline).

Variable	Intervention arm		Control arm	
	Child level N=3405 mean (SD)	Cluster level N=98 mean (SD)	Child level N=3667 mean (SD)	Cluster level N=98 mean (SD)
Any challenges faced?				
No	987 (29%)	0.28 (0.21)	926 (25%)	0.26 (0.20)
Yes	2,064 (61%)	0.61 (0.21)	2,337 (64%)	0.63 (0.21)
Missing	354 (10%)	0.11 (0.07)	404 (11%)	0.11 (0.07)
Specific challenges				
No smartphone	208 (6%)	0.06 (0.09)	230 (6%)	0.05 (0.10)
Limited access to smartphone	26 (1%)	0.01 (0.02)	27 (1%)	0.01 (0.02)
Internet connectivity issues	9 (0%)	0.00 (0.01)	11 (0%)	0.00 (0.01)
Internet costs too expensive	12 (0%)	0.00 (0.01)	8 (0%)	0.00 (0.01)
Electricity issues	15 (0%)	0.01 (0.02)	12 (0%)	0.00 (0.02)
Lack of schoolteacher support	1,031 (30%)	0.31 (0.20)	1,255 (34%)	0.34 (0.19)
Lack of time to help child	585 (17%)	0.16 (0.20)	613 (17%)	0.15 (0.19)
Low knowledge of technology	197 (6%)	0.05 (0.09)	83 (2%)	0.02 (0.05)
Child not interested	1,459 (43%)	0.45 (0.21)	1,722 (47%)	0.46 (0.19)
No money for a private tutor	228 (7%)	0.06 (0.08)	250 (7%)	0.07 (0.08)

Table S14. Learning support when schools were closed (midline).

Variable	Intervention arm		Control arm	
	Child level N=3405 mean (SD)	Cluster level N=98 mean (SD)	Child level N=3667 mean (SD)	Cluster level N=98 mean (SD)
Help for home study				
No	116 (3%)	0.03 (0.05)	339 (9%)	0.08 (0.10)
Yes	2,933 (86%)	0.86 (0.08)	2,923 (80%)	0.80 (0.12)
Don’t know	2 (0%)	0.00 (0.00)	1 (0%)	0.00 (0.00)
Missing	354 (10%)	0.11 (0.07)	404 (11%)	0.11 (0.07)
Home devices				
Regular phone bought	81 (2%)	0.03 (0.04)	59 (2%)	0.02 (0.03)
Smartphone bought	221 (6%)	0.07 (0.07)	179 (5%)	0.05 (0.06)
Tablet/computer bought	3 (0%)	0.00 (0.01)	2 (0%)	0.00 (0.00)
Access at home to				
Regular phone	445 (13%)	0.13 (0.11)	198 (5%)	0.06 (0.07)
Smartphone	804 (24%)	0.25 (0.13)	619 (17%)	0.17 (0.11)
Tablet/computer	13 (0%)	0.00 (0.01)	3 (0%)	0.00 (0.00)
Educational videos etc.	1,134 (33%)	0.35 (0.17)	768 (21%)	0.22 (0.12)
Textbooks or worksheets	2,974 (87%)	0.87 (0.07)	3,017 (82%)	0.83 (0.11)
Support from schools				
Learning materials/activities	2,553 (75%)	0.74 (0.17)	2,461 (67%)	0.66 (0.21)
Child’s progress/well-being	1,784 (52%)	0.51 (0.27)	1,418 (39%)	0.38 (0.18)
Administrative information	1,395 (41%)	0.40 (0.30)	1,120 (31%)	0.30 (0.26)

Table S15. Spending (midline) in RS. Adult caregiver spending on education between July 2020 and June 2021 (1st academic year that schools were closed).

	Intervention arm		Control arm	
	**Child level N=3405 mean (SD)**	**Cluster level N=98 mean (SD)**	**Child level N=3667 mean (SD)**	**Cluster level N=98 mean (SD)**
School materials	637.5 (935.5)	675.2 (414.6)	655.9 (998.9)	658.9 (434.3)
School fees	1093.7 (2702.8)	1136.2 (863.3)	1120.1 (3221.6)	1113.0 (955.5)
Out of school tuition	311.3 (959.5)	320.2 (303.4)	412.6 (1150.5)	413.2 (330.7)
Other	351.3 (1696.1)	351.4 (381.4)	348.2 (1820.1)	332.0 (485.8)
Total	2393.8 (4234.7)	2483.1 (1392.5)	2536.8 (5057.4)	2517.1 (1636.4)
